# Trefoil polymers from a knotted synthon

**DOI:** 10.1038/s41557-026-02197-4

**Published:** 2026-06-29

**Authors:** Chenchen Du, Zhi Chen, Changqing Xu, Zhanhu Sun, Zhi-Hui Zhang, Xian-Guang Liu, Binhua Chen, Chenxin Zhang, Zhikai Li, Yin Rao, Deao Xu, Ningjin Zhang, Qiaowei Li, Heng Wang, Hai Qian, Liang Dai, Xiaopeng Li, David A. Leigh, Liang Zhang

**Affiliations:** 1 School of Chemistry and Molecular Engineering, East China Normal University, Shanghai, People’s Republic of China; 2 State Key Laboratory of Radio Frequency Heterogeneous Integration, College of Chemistry and Environmental Engineering, Shenzhen University, Shenzhen, Guangdong People’s Republic of China; 3 State Key Laboratory of Organometallic Chemistry, Shanghai Institute of Organic Chemistry, University of Chinese Academy of Sciences, Chinese Academy of Sciences, Shanghai, People’s Republic of China; 4 Department of Chemistry, Fudan University, Shanghai, People’s Republic of China; 5 Department of Macromolecular Sciences, State Key Laboratory of Molecular Engineering of Polymers, Fudan University, Shanghai, People’s Republic of China; 6 Instrumental Analytical Center of Shanghai Jiao Tong University, Shanghai, People’s Republic of China; 7 Department of Physics, City University of Hong Kong, Kowloon, Hong Kong People’s Republic of China; 8 Department of Chemistry, University of Manchester, Manchester, UK

**Keywords:** Interlocked molecules, Polymer synthesis

## Abstract

Precise control over the presence and position of knots in polymers remains a long-standing synthetic challenge, with theory indicating broad implications for properties and function. Here we report on a topological synthon approach—extension and macrocyclization of an overhand knot—that enables the high yielding synthesis of closed-loop trefoiled polymers with narrow polydispersity and tunable topological parameters. From the same set of building blocks we prepared linear, cyclic and trefoiled topoisomers of matched chemical composition, allowing topology–property relationships to be examined directly. Low-temperature ultrahigh-vacuum scanning tunnelling microscopy resolved three conformers of trefoiled polystyrene, with high-, medium- and low-symmetry forms whose populations depend on chain length, local flexibility and solvation. Coarse‑grained Langevin dynamics reproduce these distributions and indicate that bending energy and conformational entropy govern conformer stability. Metal binding localizes the knotted region, reminiscent of protein/DNA knot translocation. The synthon also affords trefoiled poly(ethylene glycol) and trefoiled polystyrene–poly(ethylene glycol) diblock copolymers, demonstrating the generality of the approach across polymer backbones.

**Figure:**
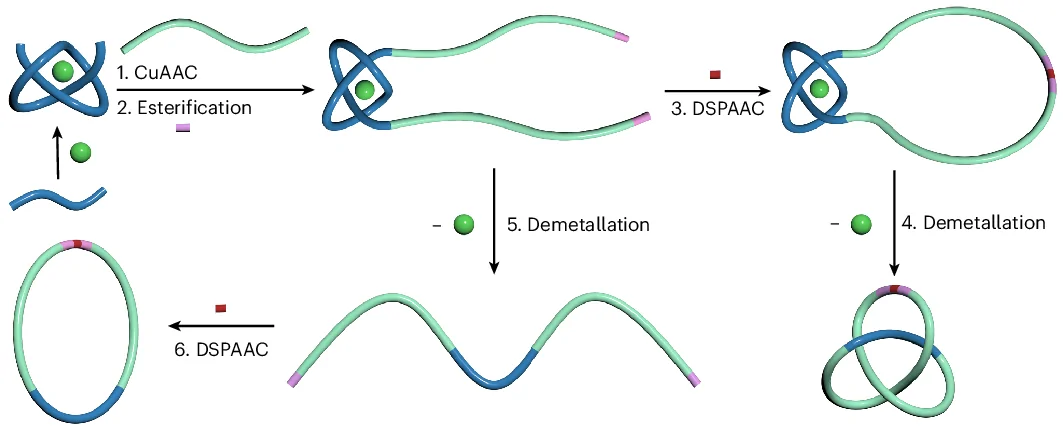

## Abstract (web summary)

An overhead knot that serves as a topological synthon enables the high-yield synthesis of trefoiled polymers with precise control over knotting. Topology–property relationships are then investigated across different polymer backbones, and the knotting influence on these properties revealed.

## Main

Theory indicates that polymer knots, arising from stochastic^1–8^ or designed^9^ entanglements, can substantially influence physical, mechanical and chemical properties^10^. However, whether knotting affects polymers differently if they have dissimilar chemical compositions remains experimentally unexplored. Computational approaches that model polymer chains as chemically undefined segments have established general principles underpinning random polymer knotting probabilities^11,12^, geometrical measures of entanglement complexity^13^, dynamics^14^, entropy^15^, dimensional scaling^16^ and the localization of knots within large rings^17^. Rare experimental studies on specific types of polymer have shown that knotting can enhance chemical reactivity and alter the mechanical response under tension^2–5^, suggesting there are opportunities to modulate properties through designing or modifying entanglements^18^.

**Fig. 1:**
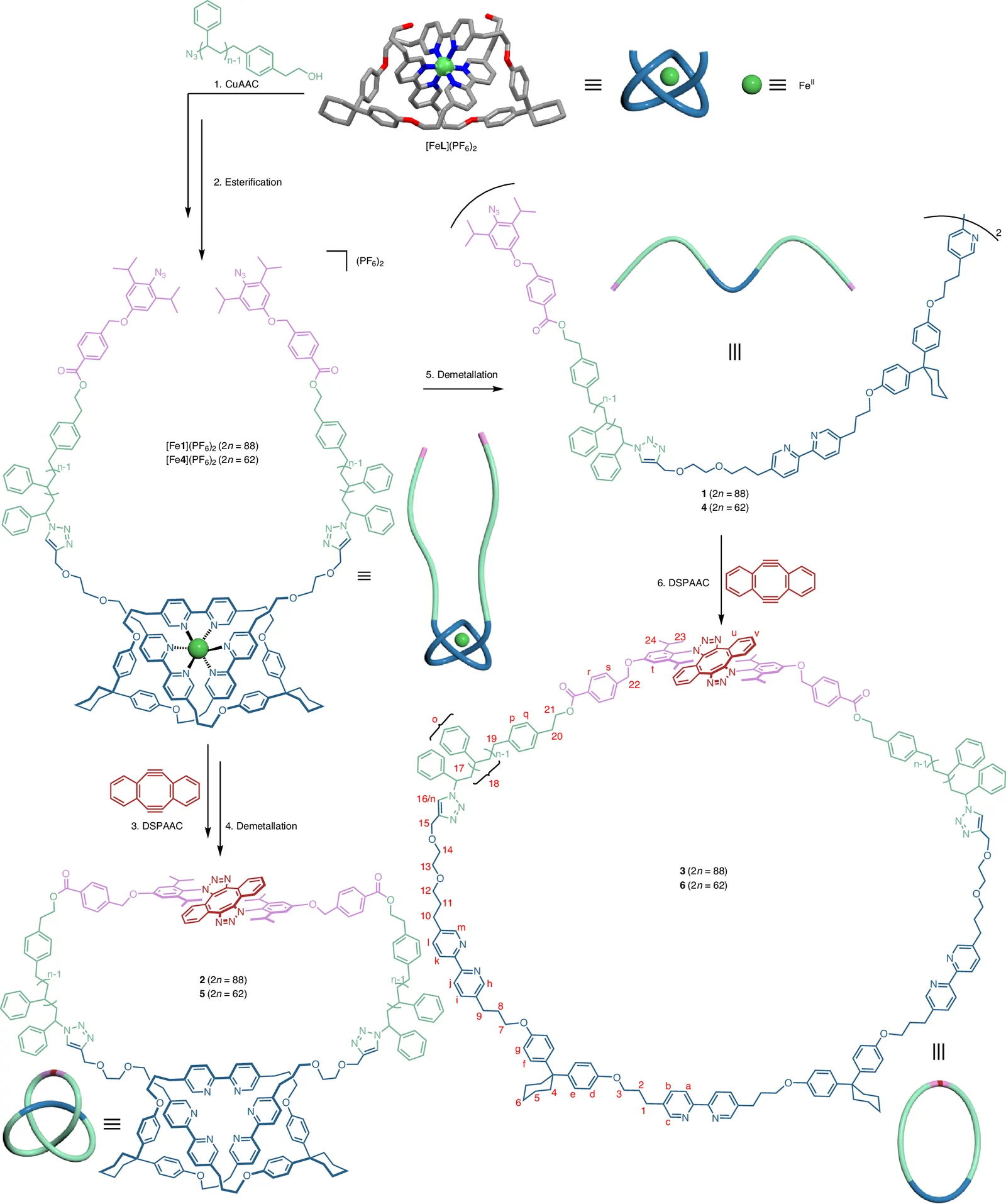
Synthesis of trefoil PS and linear and unknotted topoisomers using knot synthon [FeL1](PF_6_)_2_. Preparation of linear (**1**, **4**), trefoiled (**2**, **5**) and cyclic (**3**, **6**) PS topoisomers. 2*n* indicates the total number of PS monomers in the polymer chains. Experimental procedures and characterization data are provided in Supplementary Section 5.

The limitation in exploring how entanglements affect polymers differently depending on their chemical composition is that the synthesis of polymers with well-defined knotted topologies remains challenging^19–21^. Early approaches, such as ‘end-to-end ring closure’^19,20^ and ‘ring-expansion’ strategies^21^, are limited in scope, generally requiring extensive purification procedures^22^ and/or achieving modest polydispersity^23,24^. Metal template synthesis^25^ has proved amongst the most effective synthetic strategies to molecular knots to date, by encoding crossing information into open tangle^26^ scaffolds. We envisioned that such motifs could serve as general topological synthons by joining together appended telechelic chains to produce closed-loop knotted polymers of precise size and topology. This could allow the effects of knotting within different chemical types of polymers (for example, polyolefins, polyesters, polyethers, block copolymers) to be assessed. Here we introduce this topological synthon approach for the preparation and characterization of three different chemical classes of trefoiled polymer—polystyrene (PS), poly(ethylene glycol) (PEG) and a PS–PEG diblock copolymer (Fig. 1 and Supplementary Section 4).

## A knotted synthon

A metal-coordinated overhand knot, based on a motif previously used by Hunter to prepare a small-molecule trefoil knot^27,28^, was chosen as a general synthon for assembling trefoiled closed-loop polymers of different chemical compositions. Ligand **L1** was prepared following established procedures^27,28^. Chelation with iron(II) kinetically locked the strand as an open knot, [Fe**L1**](PF_6_)_2_, making the tangle^26^ suitable for subsequent macrocyclization. The composition and strand entanglement of the synthon was confirmed by ^1^H NMR, electrospray ionization mass spectrometry and single-crystal X-ray diffraction (Supplementary Sections 5.1 and 5.2).

## Synthesis of topological isomeric linear, cyclic and trefoiled PSs

Using the knot synthon, we constructed a series of PSs with three different topologies (**1**–**6**; Fig. 1). A PS chain (**S17**) with hydroxyl and azide end groups (*M*_n_ = 4,412 Da, *Đ* = 1.10) was synthesized via atom-transfer radical polymerization. Two PS chains were attached to each [Fe**L1**](PF_6_)_2_ synthon through copper(I)-catalysed alkyne–azide cycloaddition (CuAAC) click reactions (Fig. 1, steps 1 and 2). Macrocyclization through self-accelerating double-strain-promoted azide–alkyne cycloaddition (DSPAAC)^29,30^ with *sym*-dibenzo-1,5-cyclooctadiene-3,7-diyne (DIBOD) afforded [Fe**2**](PF_6_)_2_ in 98% yield. The structure of the closed-loop metallated trefoiled polymer, [Fe**2**](PF_6_)_2_, was confirmed by Fourier transform infrared spectroscopy, mass spectrometry and ^1^H NMR (Supplementary Sections 5.3–5.5). The high macrocyclization efficiency can be rationalized by the self-accelerating DSPAAC kinetics, in which the second cycloaddition is ∼180-fold faster than the first, biasing the reaction toward intramolecular ring closure under high dilution conditions^29,30^.

**Fig. 2:**
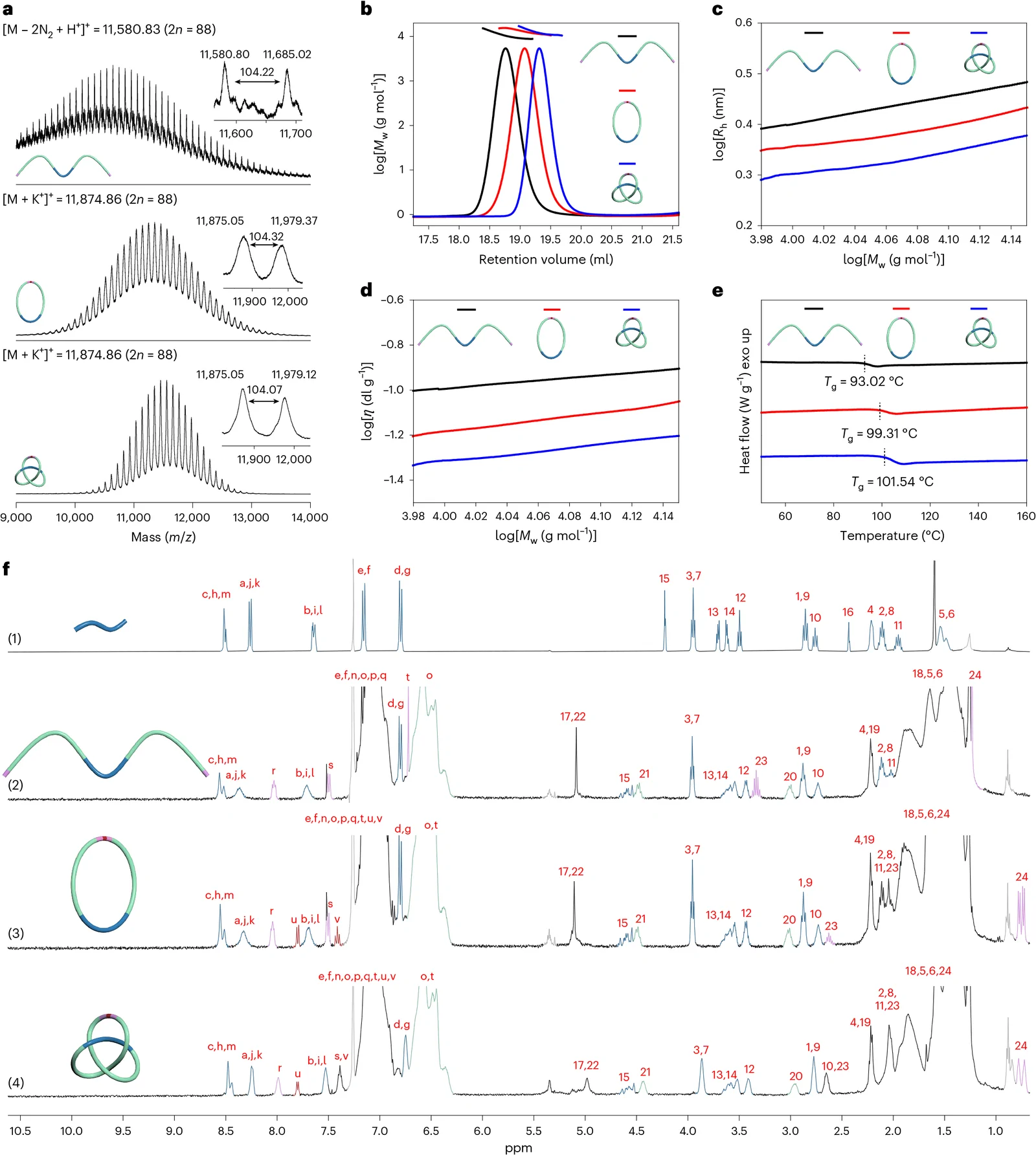
Structural determination of linear (1), cyclic (3) and trefoiled (2) PS topoisomers. **a**, MALDI-TOF MS spectra of **1**–**3**. **b**, GPC curves and logarithm of absolute weight-average molecular weight (*M*_w_) versus elution volume. **c**, Logarithm of [*R*_h_] versus the logarithm of *M*_w_. **d**, Logarithm of [*η*] versus the logarithm of *M*_w_ (Mark–Houwink–Sakurada plots). **e**, DSC curves of **1**–**3** (*T*_g_ given as the onset temperatures). **f**, ^1^H NMR spectra (400 MHz, 298 K, CDCl_3_) of (1) **L1**, (2) **1**, (3) **3** and (4) **2**. The signal assignments correspond to the labelling shown in Fig. 1.

Demetallation of [Fe**2**](PF_6_)_2_ with sodium azide (NaN_3_) in *N*,*N*-dimethylformamide (DMF) at 120 °C, followed by size-exclusion chromatography, afforded the wholly organic closed-loop trefoiled polystyrene (trefoil PS) (**2**) in 44% yield (Fig. 1, step 4, and Supplementary Section 5.6). The removal of iron(II) was accompanied by a change in colour (from brown to colourless) and was confirmed by matrix-assisted laser desorption/ionization time-of-flight mass spectrometry (MALDI-TOF MS, Fig. 2a). The observed ions correspond to [**2** + K]^+^, with a mass interval of 104 Da corresponding to the PS repeat unit.

The same building blocks were used to form two other topological isomers of **2** (Fig. 1): linear (**1**) and unknotted cyclic PS (**3**). The linear polymer (**1**) was obtained by demetallation of [Fe**1**](PF_6_)_2_ (Fig. 1, step 5). The mass difference of 200 Da between the ions of **1** and **2** with the same number of monomer units is due to the absence of DIBOD (*M*_w_ = 200.06 Da) in **1**. Macrocyclization of **1** with DIBOD afforded unknotted cyclic PS (**3**) in 44% yield (Fig. 1, step 6). The MALDI-TOF MS profile of **3** is virtually identical to that of **2** (Fig. 2a), confirming their topoisomeric chemical composition.

Although theory suggests that the lengths of flexible and stiff segments can influence knot conformational properties^31,32^, such effects have not previously been explored experimentally. In our system, PS constitutes a relatively flexible segment, whereas the bipyridine units in the knot synthon form a comparatively stiffer region. We therefore define the flexible–stiff length ratio as ${\chi }_{\mathrm{FS}}=(2{L}_{\mathrm{PS}})/{L}_{{\rm{L}}1}$, where *L*_PS_ is the length of the PS chain and *L*_L1_ is the length of ligand **L1**. To probe the effect of ${\chi }_{\mathrm{FS}}$, we prepared a second set of linear (**4**), trefoiled (**5**) and cyclic (**6**) PSs using a PS chain with a lower molecular weight (**S18**, *M*_n_ = 2,886 Da, *Đ* = 1.14; Supplementary Sections 5.5–5.7), which should produce a tighter tangle in the trefoiled polymer. The contour lengths of the PS strands are 10.35 nm (**S17**) and 7.54 nm (**S18**) (Supplementary Section 5.8), giving corresponding ${\chi }_{\mathrm{FS}}\,$ values of 3.9 (**2**) and 2.8 (**5**), respectively. Although knot localization occurs in the long-chain limit, where the most probable knot-core length becomes insensitive to the overall chain length^17,33,34^, the polymer lengths studied here do not reach this regime (Supplementary Section 11).

## Characterization of the different PS topoisomers

By comparing macromolecules of similar molecular weights and polydispersities, the variations in physical properties can be attributed to differences in topology. Gel permeation chromatography (GPC) showed that trefoiled PS **2** has a smaller hydrodynamic volume (*R*_h_) and lower intrinsic viscosity ([*h*]) than linear PS **1** (Fig. 2b–d). The *g*′ factor (the ratio of the intrinsic viscosity, [*h*]_cyclic_/[*h*]_linear_) is 0.66 across the various molecular weights^21,22^, whereas the [*h*]_knotted_/[*h*]_linear_ and [*h*]_knotted_/[*h*]_cyclic_ ratios were found to be 0.48 and 0.73, respectively. Differential scanning calorimetry (DSC) analysis (Fig. 2e) showed a gradual increase in glass transition temperature (*T*_g_) in going from linear (93.0 °C; **1**) to cyclic (99.3 °C; **3**) to trefoil knot (101.5 °C; **2**), attributable to the progressively tighter structures and reduced free volumes. Similar trends were observed for **4**–**6** (Supplementary Section 5.7). The *T*_g_ values increased with molecular weight for both the linear (93.0 °C for **1** versus 77.8 °C for **4**) and cyclic polymers (99.3 °C for **3** versus 93.3 °C for **6**), but the lower molecular weight knot (**5**) exhibited a higher *T*_g_ (106.8 °C) than the higher molecular weight analogue **2** (101.5 °C). This may be due to a tighter conformation of **5** resulting in a further reduction of free volume. Thermogravimetric analysis (TGA) showed that the topology of **1**–**6** has a negligible effect on thermal stability (Supplementary Section 6), probably because thermal decomposition is largely a consequence of chemical composition rather than the spatial arrangement of the chain.

The influence of polymer topology in solution was probed by ^1^H NMR (Fig. 2f). In CDCl_3_ the proton resonances corresponding to the tris-bipyridine units in PS **1*–*3** have similar chemical shifts to those in ligand **L1**, suggesting that the knot is diffused along the polymer backbone rather than restricted to the synthon section. A slight upfield shift (0.02*–*0.11 ppm) and broadening of the bipyridine proton signals were observed in **2**, probably due to increased C−H···*π* interactions within the knotted loop. Variable-temperature NMR of **2** showed little change over the range 223–338 K (Supplementary Section 5.9), indicating that the knot does not adopt a single well-defined conformation but rather undergoes knot ‘breathing’ (fluctuations in the knot-core length) and/or sliding along the polymer backbone. The UV–visible spectra of all six polymers have similar absorbance bands, consistent with the chromophores being in chemically similar environments across the different topologies (Supplementary Section 7).

The diffusion coefficient (*D*) of cyclic PS **3** was higher than that of the linear analogue **1** across a range of solvents with varying polarities (CDCl_3_, tetrahydrofuran-*d*_8_, dimethylsulfoxide-*d*_6_, Cl_2_CDCDCl_2_), indicating a smaller effective hydrodynamic radius (*R*_h_) as derived from diffusion-ordered spectroscopy measurements and supported by GPC analysis. In contrast, trefoil PS **2**, the topology with the smallest apparent *R*_h_, showed similar or smaller *D* values in these solvents compared to **3**. This can be attributed to a non-free draining effect^35^, by which the knotted structure creates multiple smaller rigid segments within the polymer chain, hindering solvent molecules from freely diffusing through the solvated rings. A similar trend was observed for the lower-molecular-weight polymers **4**–**6** (Supplementary Section 8).

## Visualization of PS topoisomers via scanning tunnelling microscopy

**Fig. 3:**
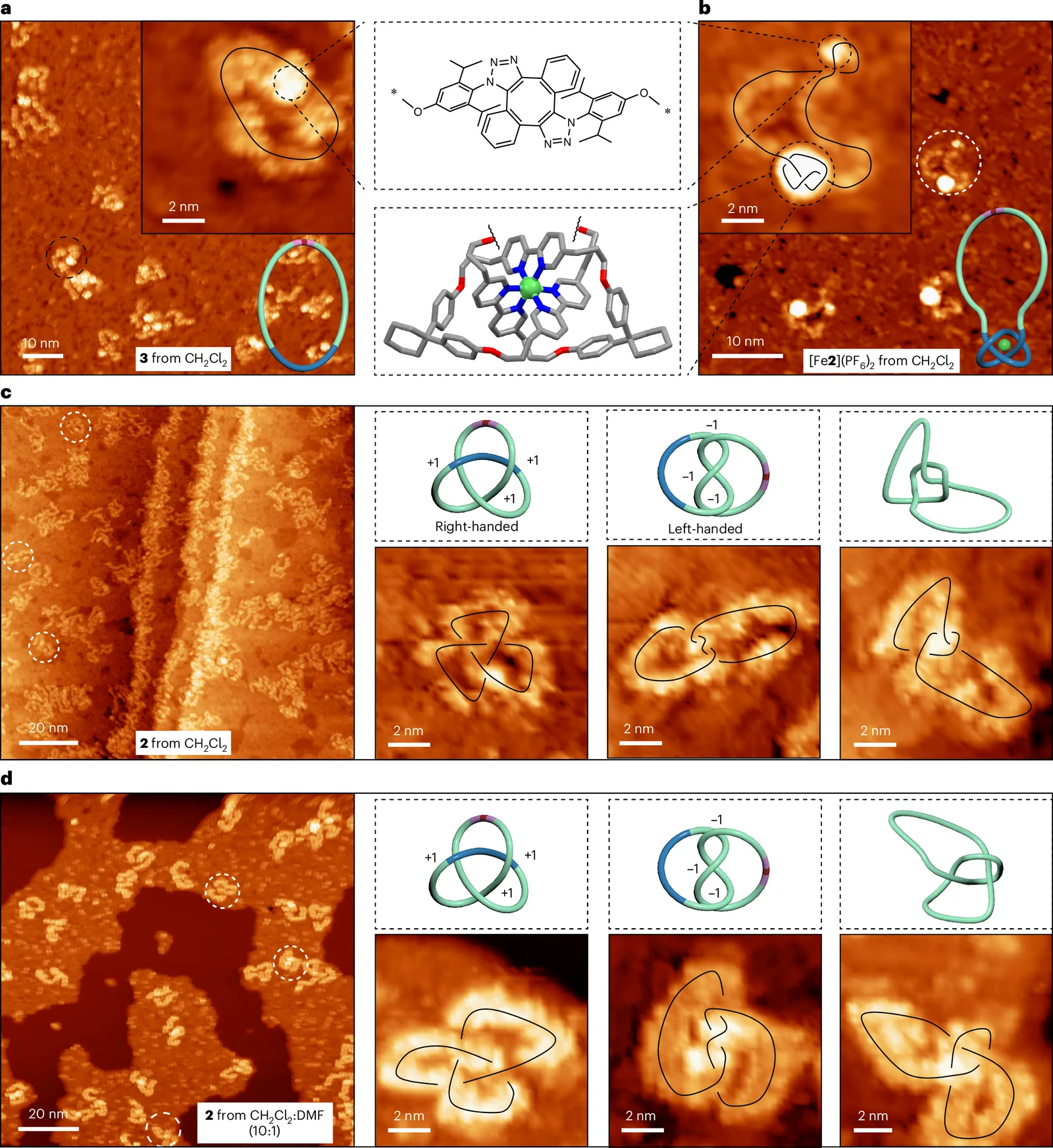
Direct observation of PS topoisomers via STM. **a**, STM images of cyclic PS **3** showing a collapsed, ring-like morphology including a bright lobe attributable to DIBOD. **b**, STM images of [Fe**2**](PF_6_)_2_ show two bright lobes, the smaller corresponding to the DIBOD units and the larger to the iron(II)-tris-bipyridine coordination centre. **c**, STM images of **2** show three types of conformers: high-, medium- and low-symmetry trefoil. The three crossings in the trefoil knot have the same handedness, so identifying one also gives the knot chirality. **a**–**c** were recorded from samples deposited from CH_2_Cl_2_. **d**, STM images of **2** (deposited from CH_2_Cl_2_:DMF (10:1)) show that the conformer ratios are altered by the use of different solvents for deposition. Scanning parameters for **a**–**d**: *U* = 2.7 V and *I*_t_ = 20 pA. *U*, bias between sample and tip; *I*_t_, tunnelling current.

During reptation the whole of the polymer chain migrates through the region of entanglement, potentially generating different structures that differ in size, shape and tightness of loops and tangles in the strand. Theory suggests there will be influenced by molecular weight, backbone rigidity, solvation conditions, temperature and the presence of external forces such as shear, but again the significance of these different factors for a polymer of particular chemical composition has previously been unexplored^7,8,31^. Atomic force microscopy has previously provided only limited information on the structure of knotted polymers, mainly from height profile differences (Supplementary Section 9). To probe the conformations and spatial arrangement of entanglements, we instead used ultrahigh-vacuum low-temperature scanning tunnelling microscopy (STM). Very dilute dichloromethane (CH_2_Cl_2_) solutions (*c* = 5 × 10^−7^ M) of the various polymers, with and without coordination to iron(II), were drop-cast onto a freshly cleaned and annealed Ag(111) surface for imaging (Supplementary Section 10). This yielded high-resolution STM images of knotted polymers and revealed previously elusive evidence for different types of well-defined conformation being present in a knotted polymer of particular chemical composition (Fig. 3).

The STM images of linear PS (**1**) showed linear or kinked chains on the Ag(111) surface (Supplementary Fig. 70a). A distinct small, bright lobe is present in all of the STM images of knotted (**2**) or cyclic (**3**) PS (Fig. 3), attributable to the DIBOD moiety (the pronounced electron density contrast is due to its conjugation and the nature of its interaction with the Ag(111) surface). Cyclic PS **3** principally adopts a single type of conformation on the surface: a collapsed, roughly oval, shape (Fig. 3a and Supplementary Fig. 70b). The metallated trefoiled polymer, [Fe**2**](PF_6_)_2_, with the tangled portion of the strand localized to the metal-coordinated region, also adopts a single ring-like configuration (Fig. 3b and Supplementary Fig. 77), with a second bright lobe composed of three smaller spots in a triangular arrangement due to the iron(II)-tris-bipyridine unit. In contrast, in metal-free trefoiled PS (**2**) the STM images show a much looser knotted region that is no longer rigidly anchored at the synthon but instead appears distributed along the PS chains (Fig. 3c,d and Supplementary Figs. 71–74). Unlike previous atomic force microscopy studies that could only identify^20,21^ the conformation of knotted PS as being roughly symmetric, the high-resolution STM images show three distinct trefoil conformations: high, medium and low symmetry. In the high-symmetry conformation, the knotted loop has approximate *C*_3_-symmetry with a triangular arrangement of strand crossings (Fig. 3c,d, left single-knot images). In the low-symmetry conformation, one of the three loops merges into the knotted region (Fig. 3c,d, right single-knot images), whereas the medium-symmetry conformer has a double-helical structure with two loops, in which the three crossings are arranged in a nearly linear fashion (Fig. 3c,d, central single-knot images). As the tangle synthon is racemic, both left-handed (Λ) and right-handed (Δ) knot enantiomers are present in the STM images (Fig. 3c,d).

The three-dimensional nature of knots lessens the intensity of tunnelling current, reducing the ultrahigh-resolution imaging achievable for planar structures^36–39^. Nevertheless, analysis of STM images of 69 individual molecules of **2** (deposited from CH_2_Cl_2_) shows 33% (23 molecules) in the high-symmetry trefoil conformation, a proportion that decreases to 4% (2 out of 54 molecules) when the solvent polarity was increased (CH_2_Cl_2_:DMF (10:1)) for sample deposition (Supplementary Figs. 71–74). This suggests that the arrangement of the entanglements in the PS chain is particularly sensitive to solvation. The results also show that, in addition to solvation, *χ*_FS_ and knot tightness also play an important role in the distribution of conformers. For the shorter strand trefoil PS, **5**, which has a more rigid backbone (*χ*_FS_ = 2.8), the proportion of the high-symmetry conformer deposited from CH_2_Cl_2_ was substantially lower (10%, 4 out of 39 molecules, Supplementary Fig. 75) than for **2** (33%; Supplementary Fig. 73). These findings show that the conformations of knotted PS are governed by a delicate interplay between solvation and chain flexibility. The use of a metal chaperone^40^ to relocate a diffuse entanglement to a ‘tight’ knot in a specific region of the strand (Fig. 3b) is reminiscent of translocation processes that occur in knotted proteins and DNA^41^.

## Mechanistic insights from coarse-grained simulations

**Fig. 4:**
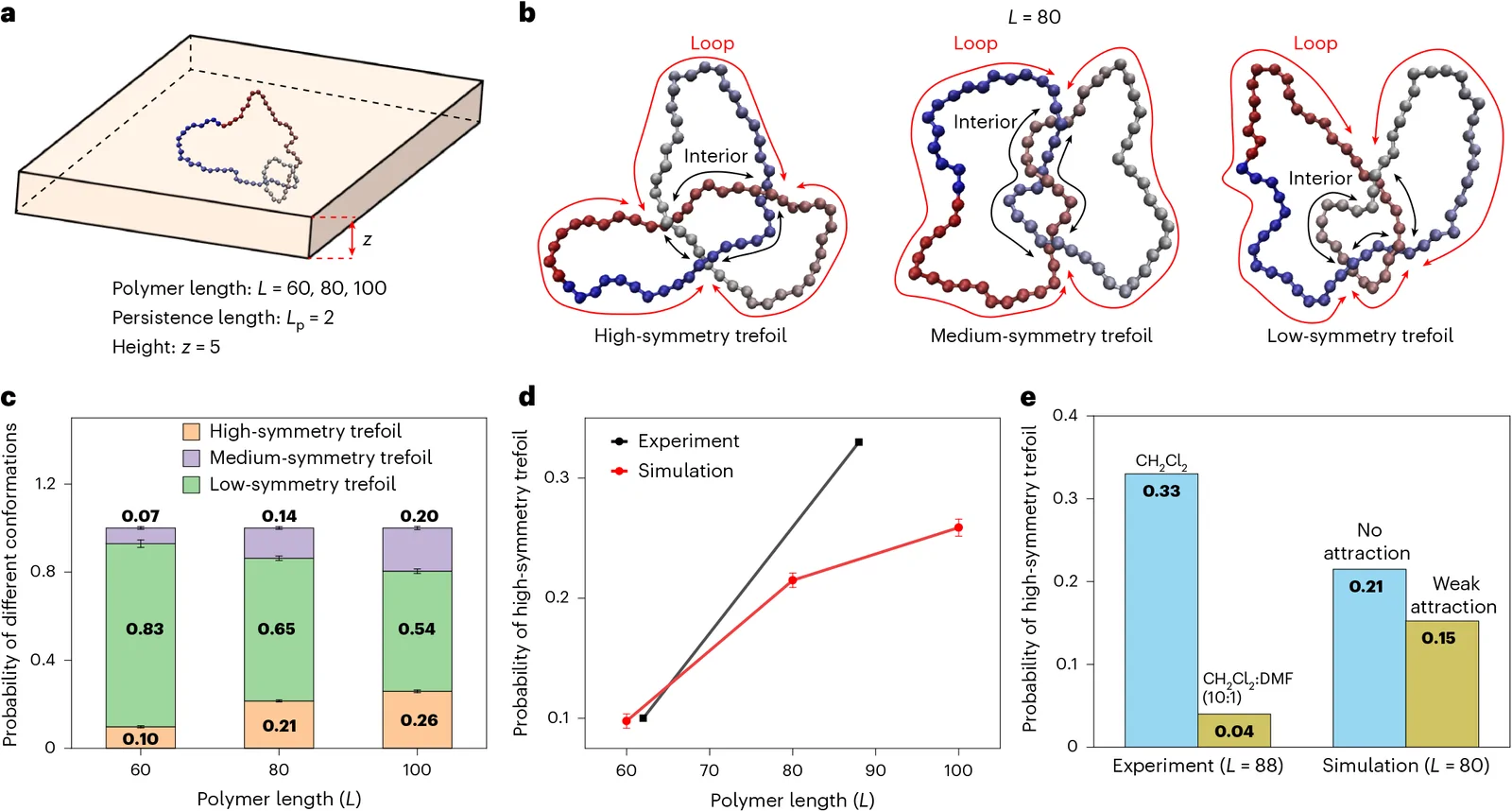
Conformational diversity and transitions in a trefoil knotted polymer. **a**, Snapshot of simulation of a trefoil knotted polymer. Spatial confinement in the *z* direction (height) was used to mimic the polymer on a surface. **b**, Schematic diagram of three conformers: high-symmetry trefoil (three loops; the shortest loop >11 monomers between crossings), low-asymmetry trefoil (three loops; the shortest distance between crossings less than or equal to 11 monomers), and medium-symmetry trefoil (two loops). The cut-off value *L*_short_ = 11 is chosen based on the spatial resolution of the STM measurements. In our simulation, each bead represents approximately one styrene repeat unit with a backbone length of ∼0.25 nm, thus, 11 beads correspond to a counter length of ∼2.8 nm. The red and black arrow lines indicate the loops and interior, respectively. **c**, Fraction of the three conformers present for different polymer lengths. **d**, Fraction of high-symmetry trefoil conformer in STM experiments (*L* = 62, 88) and simulations. **e**, Effects of solvent or monomer–monomer attraction on the probability of high-symmetry trefoil in experiment and simulation.

To further understand the conformational behaviour of trefoiled PS, we performed coarse-grained (CG) Langevin dynamics simulations^42^ using the LAMMPS package (Supplementary Section 11)^43^. In the model, the polymer was represented as a bead-spring chain and confined between two parallel planes^44,45^ to emulate surface adsorption (Fig. 4a). Our simulations used three polymer lengths: 60, 80 and 100. Other parameters can be found in Supplementary Section 11. Under this confinement, two dominant conformations emerged: two-loop and three-loop conformers (Fig. 4b). Transition between the conformers requires a flipping event with an energy barrier of several ${k}_{{\rm{B}}}T$ (Supplementary Fig. 78a). Typically, tens of such transitions occur over 10^9^ simulation steps, which allowed the calculation of the occurrence probabilities for the different conformers. In addition to the confined knots, we also performed simulations of adsorbed knots^46^, which exhibit similar results as the confined knots (Supplementary Section 11).

To facilitate comparison of the simulations with the STM data, the three-loop conformations were further classified as high or low symmetry based on the shortest length between crossings in the major loops (*L*_short_ > 11 or *L*_short_ ≤ 11, respectively), while the two-loop conformer was treated as medium symmetry (Fig. 4b). Simulations of different polymer lengths revealed two particular trends consistent with the experimental observations: the high-symmetry trefoil conformation accounts for less than half of the total population, and its frequency increases with polymer length (Fig. 4c,d).

We focus on the high-symmetry probability rather than the three-loop probability, because identification of high-symmetry states, defined as conformations in which all loop lengths exceed a threshold, is more reliable in STM images. In contrast, identification of all three-loop states includes conformations with one or more subresolution loops, which cannot be unambiguously resolved experimentally.

The tightest trefoil knots are expected to adopt the three-loop state^47^. Consistent with this expectation, our simulations show that the three-loop probability increases for shorter polymers (Supplementary Section 11). Notably, however, for shorter polymers the three-loop probability increases while the high-symmetry probability decreases. This opposite trend arises because, for shorter three-loop knots, the loop lengths are more likely to fall below the experimental resolution and therefore are not classified as high-symmetry states.

Of the three different conformers, the high-symmetry trefoil showed the smallest root mean square displacement, indicating a lower conformational entropy. Bending energy differences between the high- and low-symmetry conformers changes from positive to negative with increasing chain length, favouring high-symmetry conformations in longer polymers (Supplementary Fig. 78b).

To probe the influence of solvent, we introduced weak monomer–monomer attractions in simulations to understand the experimental results under conditions of poor solvation. This reduced the population of high-symmetry conformers, in agreement with the STM results (Fig. 4e), a finding that can be rationalized by the fact that high-symmetry trefoils have fewer short-distance contacts: on average, the high-symmetry trefoil conformation contained 10.45 short-distance pairs, compared with 11.15 in the medium-symmetry ones.

Finally, analysis of bending curvature across different conformational regions revealed consistently higher curvature in the outer loop segments relative to the interior (Supplementary Fig. 78e,f), supporting the interpretation that more rigid elements, such as the tris-bipyridine synthon section, preferentially localize near the interior crossing points of the knot.

## Synthesis of trefoiled PEG and trefoiled PS–PEG block copolymers

**Fig. 5:**
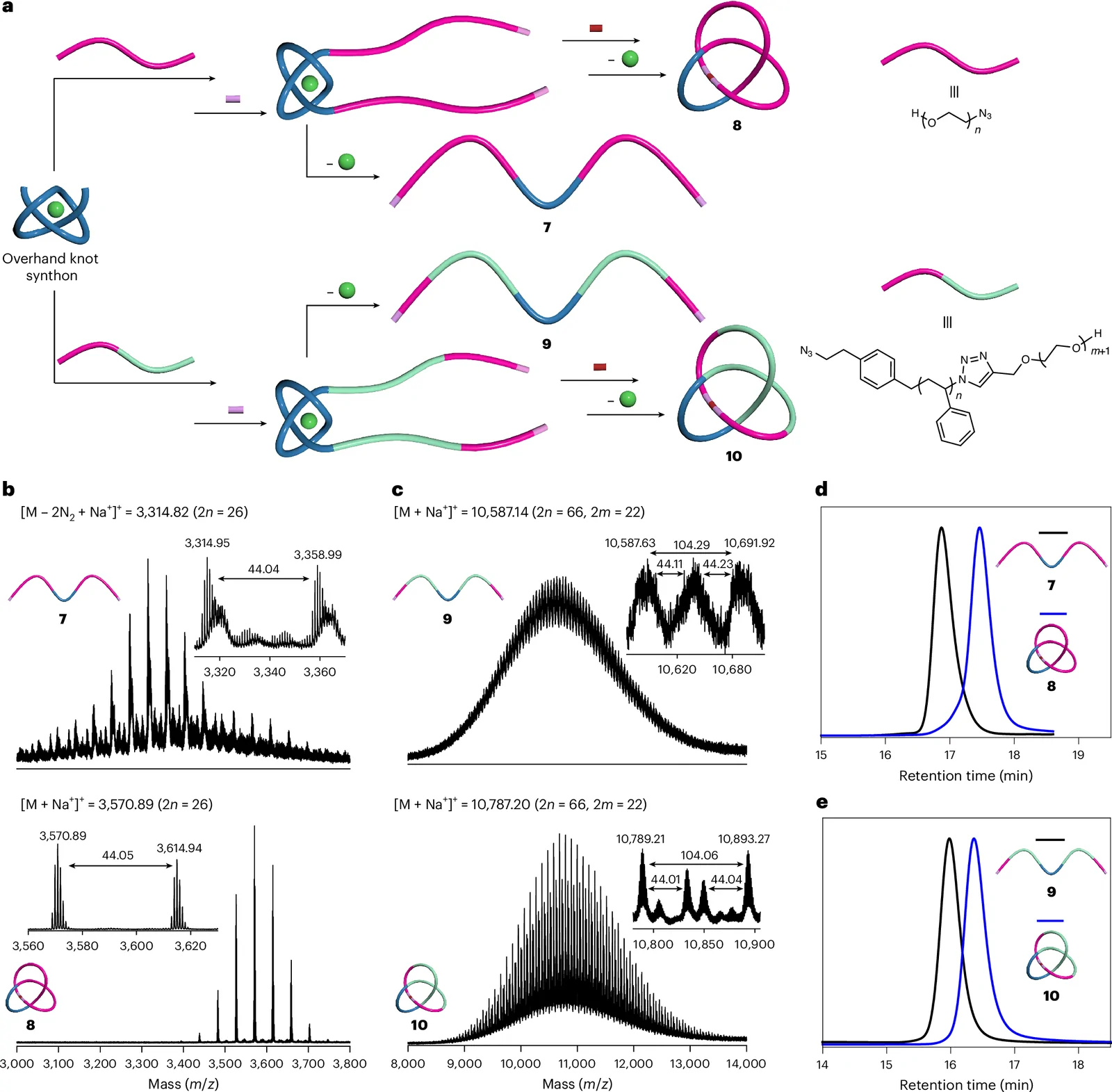
Synthesis of trefoiled PEG 8 and trefoiled PS–PEG diblock copolymer 10 and their linear analogues. **a**, Overhand knot synthon [Fe**L1**](PF_6_)_2_ enables the synthesis of precise trefoil knotted closed-loop polymers with otherwise inaccessible backbones, such as PEG **8**, and PS–PEG diblock copolymer **10**. **b**, MALDI-TOF MS spectra of linear (**7**) and trefoiled (**8**) PEG polymers. 2*n* indicates the total number of PEG monomers in the polymer chains. **c**, MALDI-TOF MS spectra of linear (**9**) and trefoiled (**10**) PS–PEG diblock copolymers. 2*n* indicates the total number of PS monomers and 2*m* indicates the total number of PEG monomers in the co-polymer chains. **d**, GPC curves of **7** and **8**. **e**, GPC curves of **9** and **10**.

Importantly, the knot synthon approach should be readily extendable to the synthesis of precisely knotted polymers with other chemical compositions. To demonstrate this we used [Fe**L1**](PF_6_)_2_ to prepare both trefoiled PEG (**8**; 60% yield, *Đ* = 1.06;) and a trefoiled PS–PEG diblock copolymer (**10**; 69% yield, *Đ* = 1.07), along with their linear analogues (**7** and **9**, respectively) (Fig. 5a and Supplementary Section 12). As with the PS polymers **1**–**6**, the topologies of the knotted and linear PEG and PS–PEG macromolecules was confirmed through ^1^H NMR, MALDI-TOF MS (Fig. 5b,c) and GPC (Fig. 5d,e). GPC indicates that trefoiled polymers exhibit systematically smaller hydrodynamic volumes and lower intrinsic viscosities than their linear analogues for both PEG and PS–PEG backbones (Supplementary Figs. 93 and 99). DSC further shows pronounced increases in *T*_g_ upon trefoil formation (for example, −36.15 °C to 2.18 °C for PEG and 31.02 °C to 61.92 °C for PS–PEG) (Supplementary Fig. 94 and 100). These results demonstrate that topology alone, independent of chemical composition, has an important influence on bulk polymer properties.

## Conclusions

The experimental investigation of the mechanical, thermal, dynamic and conformational properties of knots in polymers has previously been limited by the lack of suitable routes to well-defined knotted macromolecules. The topological synthon approach enables the synthesis of trefoiled polystyrenes of precise size, along with linear and cyclic topoisomers of similar chemical composition. Their preparation allowed STM to reveal the existence of three distinct conformational isomers of trefoil PS, with the ratios of the different conformations determined by the solvent of deposition and the length and rigidity of the knotted PS chain. Coarse-grained simulations indicate that the relative abundance of the different conformations is governed by bending energy, conformational entropy and strand length, consistent with the experimental results. The knot synthon approach was extendable to the synthesis of trefoiled polymers with other backbones. We anticipate that combining together multiple trefoil synthons should generate composite knots (for example, granny/square knots), and that other synthons *(*for example, based on 5_1_, 5_2_ or 8_19_ small-molecule knots) should allow the precise synthesis of polymers with higher-order topologies^40,48,49^.

## Methods

### Synthesis of trefoiled PS 2

NaN_3_ (34.0 mg, 523 μmol) was added to a solution of [Fe**2**](PF_6_)_2_ (200 mg, 17.4 μmol) in DMF (60 ml). The reaction mixture was stirred at 120 °C for 0.5 h. After completion, the crude product was precipitated from water (100 ml) and filtrated on celite, followed by washing the solid with water and methanol. The solid was redissolved in CH_2_Cl_2_ and evaporated to dryness under vacuum. The crude product was purified by size-exclusion chromatography to give pure **2** as a white solid (86 mg, yield 44%, *M*_n(absolute)_ = 11,852 Da, *Đ* = 1.02).

### STM measurement of trefoiled PS 2

Ultrahigh-vacuum low-temperature STM images were conducted on a CreaTec Fischer LT-STM-AFM-BO scanning tunnelling microscope at 77 K. The Ag(111) substrate was cleaned by repeated cycles of Ar^+^ sputtering (1.5 kV) followed by annealing at 700 K. An electrochemically etched polycrystalline tungsten wire was used for the STM tip. **2** was dissolved in CH_2_Cl_2_ at a concentration of 5.0 × 10^−7^ M. **2** was also dissolved in the mixture of CH_2_Cl_2_ and DMF (10:1 v/v) at a concentration of 5.0 × 10^−7^ M to research the influence of polarity on the morphology of the knot. Onto the freshly annealed Ag(111) surface, 0.02 ml of the solution was deposited by drop casting. The obtained STM images were analysed using WSxM5.0 software.

## Online content

Any methods, additional references, Nature Portfolio reporting summaries, source data, extended data, supplementary information, acknowledgements, peer review information; details of author contributions and competing interests; and statements of data and code availability are available at https://doi.org/10.1038/s41557-026-02197-4.

## Supplementary information

Supplementary Information Supplementary Sections 1–14, including Figs. 1–101 and Tables 1–3.

Peer Review File

## Data Availability

Crystallographic data have been deposited at the Cambridge Crystallographic Data Centre (http://www.ccdc.cam.ac.uk/) under CCDC number 2423451. These data can be obtained free of charge via http://www.ccdc.cam.ac.uk/structures/. All other data supporting the finding of this study are available within the article and its Supplementary Information.
