# Supplementary material for: Trefoil polymers from a knotted synthon
Source: Nat Chem. 2026 Jun 29;18(10):1708–16. doi: 10.1038/s41557-026-02197-4 (PMC13623649; doi:10.1038/s41557-026-02197-4)
Supplement: Supplementary file 1 [file 41557_2026_2197_MOESM1_ESM.pdf]

nature chemistry

Supplementary information https://doi.org/10.1038/s41557-026-02197-4

Trefoil polymers from a knotted synthon

In the format provided by the authors and unedited

Table of contents

1. General methods and information 2

2. Abbreviations 3

3. Comparison between knot synthon with previous reported synthetic strategies 4

4. Synthetic overview 6

5. Synthetic procedures and characterization details 6

5.1 Synthesis of [Fe**L1**](PF_6)$_2$ 7

5.2 X-ray crystal structure information of [Fe**L1**](PF_6)$_2$ 19

5.3 Synthesis of **S17** and **S18** 21

5.4 Synthesis of **S23** 25

5.5 Synthesis of [Fe**2**](PF_6)$_2$ and [Fe**5**](PF_6)$_2$ 26

5.6 Synthesis of linear (**1**, **4**), trefoiled (**2**, **5**) and cyclic (**3**, **6**) polymers 33

5.7 ^{1}H NMR, MALDI-TOF-MS spectra, GPC analysis and DSC curves of **4**, **5**, **6** 37

5.8 Molecular models of **L1**, **S17** and **S18** 40

5.9 Variable-temperature NMR (VT-NMR) spectra of linear (**1**, **4**), trefoiled (**2**, **5**) and cyclic (**3**, **6**) polymers 41

6. Thermal gravimetric analysis (TGA) of linear (**1**, **4**), trefoiled (**2**, **5**) and cyclic (**3**, **6**) polymers 46

7. UV-Vis and fluorescence emission spectra of linear (**1**, **4**), trefoiled (**2**, **5**) and cyclic (**3**, **6**) polymers 47

8. Dosy spectra of **1**, **2**, **3**, **4**, **5**, **6** in different solvents 48

9. AFM iamges of linear (**1**), trfoiled (**2**) and cyclic (**3**) polymers 61

10. STM iamges of **1**, **2**, **3**, **5** and [Fe**2**](PF_6)$_2$ 63

11. Coarse-graining (CG) simulation 70

12. Synthesis of linear (**7**, **9**) and trefoiled (**8**, **10**) polymers 77

12.1 Synthesis of **S30** and **S33** 77

12.2 Synthesis of linear (**7**) and trefoiled (**8**) PEG polymers 83

12.3 Synthesis of linear (**9**) and trefoiled (**10**) diblock PS-PEG polymers 90

13. Potentials 98

14. References 99

1. General methods and information

All reagents and solvents were purchased from Energy Chemical and used without further purification unless otherwise specified. **S24** was synthesized according to the literature report.[1] CuBr was purified by stirring overnight in acetic acid at room temperature, followed by washing with ethanol and acetone prior to dry under vacuum at 40 °C for 12 hours. Styrene was washed with the aqueous solution of NaOH (5%, repeated three times) and water (repeated three times), followed by filtrating though basic alumina. Nuclear magnetic resonance (NMR) spectra were recorded on Bruker Avance III 400 MHz (^{1}H: 400 MHz; ^{13}C: 101 MHz) or 500 MHz (^{1}H: 500 MHz; ^{13}C: 126 MHz). Chemical shifts are reported in parts per million (ppm) from high to low frequency and referenced to the residual solvent resonance. Coupling constants (J) are reported in hertz (Hz). Standard abbreviations indicating multiplicity were used as follows: s = singlet, d = doublet, t = triplet, q = quartet, dd = double doublet, ddd = doublet of double doublets, dt = double triplet, m = multiplet, br = broad. ^{1}H assignments were made using 2D NMR methods (COSY and HSQC). Diffusion ordered spectroscopy (DOSY) measurements were performed using the standard Bruker pulse program dstebpgp1s. The gradient recovery delay was 200 µs, and diffusion time Δ = 45, 55 or 60 ms were used for the experiments. Eight gradient increments were acquired, ranging from 0.96–45.74 G·cm^{-1} in equal steps of gradient squared. High-resolution electrospray ionization mass spectrometry (HR ESI-MS) was performed with a Waters XEVO G2-XS TOF Mass Spectrometer. Low-resolution electrospray ionization mass spectrometry (LR ESI-MS) was performed with a Thermo Scientific LTQ XL Fleet Ion Trap Mass Spectrometer equipped with an Advion Expression single quadrupole MS detector. Matrix-assisted laser desorption/ionization time-of-flight mass spectrometry (MALDI-TOF MS) was conducted on a Bruker UltrafleXtreme TOF/TOF mass spectrometer (Bruker Daltonics, Inc., Billerica, MA) equipped with a Nd: YAG laser (355 nm). Trans-2-[3-(4-tert-butylphenyl)-2-methyl-2-propenylidene] malononitrile (DCTB, purchased from TCI) was used as matrix. The sample was measured in linear mode for knotted polymers (**2** and **5**) and macrocycles (**3** and **6**) and reflection mode for linear polymers (**1** and **4**). Fourier transform infrared (FT-IR) spectroscopy was performed on a Shimadzu IRtracer-100 spectrometer at room temperature in the range of 500–4000 cm^{-1}. Thermal gravimetric analysis (TGA) was conducted using a Netzsch STA 449 F3 Jupiter® instrument under nitrogen atmosphere. Each sample was heated from 30 °C to 720 °C with a rate of 10 °C min^{-1}. Differential scanning calorimetry (DSC) was recorded on a TA Instruments DSC Q2000 with a temperature ramp of 10.0 °C min^{-1} to 200 °C under nitrogen atmosphere. Plotted data represents the second heating scan. The absolute molecular weight (M_n, M_w), intrinsic viscosity (η), hydrodynamic radius (R_h) and molecular weight distributions ($Đ$) of the polymers were determined by a Malvern Panalytical Viscotek TDAmax instrument which equipped with a differential viscometer (DV) detector, a right-angle laser-light scattering (RALLS, Viscotek) detector, a low-angle laser-light scattering (LALLS, Viscotek) detector, and a refractive index (RI) detector. The columns consist of one PL 10 mm guard column (50 × 7.5 mm^2) followed by one Viscotek T6000 M column (8.0 × 300 mm, 10 mm bead size; 104 Å pore size) and one Viscotek T4000 column (8.0 × 300 mm, 6 mm bead size; 1.5 × 103 Å pore size). The analysis was performed at 35 °C using THF as eluent with a flow rate of 1.0 mL/min. The relative molecular weights of the polymers were calibrated using monodisperse polystyrene standards. Gel permeation chromatography (GPC) measurements were also conducted using an Agilent 1260 Infinity II series equipped with a G7110B isocratic Pump, a G7162A refractive index detector, and Agilent GPC/SEC Columns connected in series (PLgel 10 µm MIXED-B, Serial No. 0006721934-40; PLgel 5 µm MIXED-C, Serial No. 0006717522-199) in THF at 35 °C with an elution rate of 1.0 mL/min.

2. Abbreviations

THF: Tetrahydrofuran
DMF: Dimethyl formamide
DMSO: Dimethyl sulfoxide
MeCN: Acetonitrile
MeOH: Methanol
DCM: Dichloromethane
EA: Ethyl acetate
PE: Petroleum ether
TBTA: Tris(benzyltriazolylmethyl)amine
DMAP: 4-dimethylaminopyridine
LDA: Lithium diisopropylamide
TBAF: Tetrabutylammonium fluoride
TsCl: 4-toluene sulfonyl chloride
TEA: Triethylamine
DNBPY: 4,4'-Dinonyl-2,2'-bipyridine
DCTB: Trans-2-[3-(4-tert-butylphenyl)-2-methyl-2-propenylidene] malononitrile
NMR: Nuclear magnetic resonance
COSY: Correlated Spectroscopy
HSQC: Heteronuclear Single Quantum Coherence
HR ESI-MS: High-resolution electrospray ionization mass spectrometry
LR ESI-MS: Low-resolution electrospray ionization mass spectrometry
MALDI-TOF MS: Matrix-assisted laser desorption/ionization time-of-flight mass spectrometry
GPC: Gel permeation chromatography
SEC: Size-exclusion chromatography
FT-IR: Fourier transform infrared
DSC: Differential scanning calorimetry
DV: Differential viscometer
RI: Refractive index
LALLS: Right-angle laser-light scattering
LALLS: Low-angle laser-light scattering
STM: Scanning tunneling microscopy

3. Comparison between knot synthon with previous reported synthetic strategies

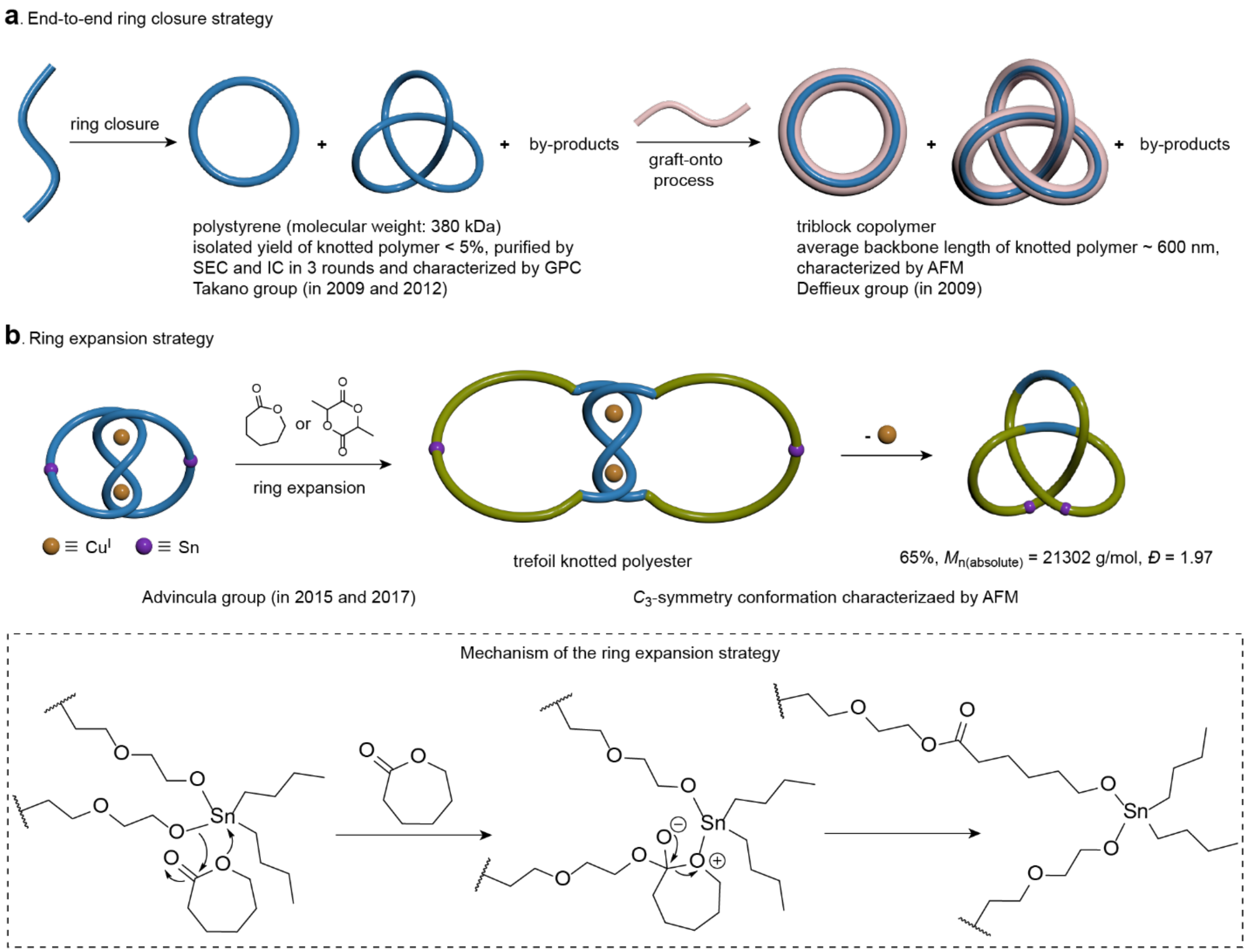

Figure 1. (a) Tanaka and co-workers pioneered the isolation of trefoiled PS via an end-to-end ring closure strategy; however, the inherent stochasticity of this method results in low yields (<5%) and difficult separations. Concurrently, Deffieux and colleagues employed AFM imaging following a grafting-onto process, which, due to backbone rigidification, predominantly reveals the trefoil conformation. The corresponding trefoiled polymer was also synthesised through end-to-end cyclisation, accompanied by the formation of oligomeric, linear, cyclic, and [2]catenated polymers. (b) Advincula's ring-expansion strategy enabled the synthesis of polymer chains features topological configurations, including [2]catenane (Hopf link) and trefoil knot, but is limited by monomer scope (lactones or lactides) and control over dispersity, with AFM again predominantly showing C_3-symmetric trefoil structures.

Table 1. Comparison of knot synthon with previous reported synthetic strategies.

Strategies	Yield	Molecular weight distributions (*Đ*)	Backbones	Topoisomers	Characterization
End-to-end ring closure	< 5%	-	PS and Triblock copolymer	Yes	AFM High-symmetry trefoil
Ring expansion	65%	1.97	lactones and lactides	No	AFM High-symmetry trefoil
Knot synthon	44–69%	1.02–1.07	Various backbones: PS, PEG, PS-PEG copolymer etc.	Yes	STM High-, medium- and low-symmetry trefoil

4. Synthetic overview

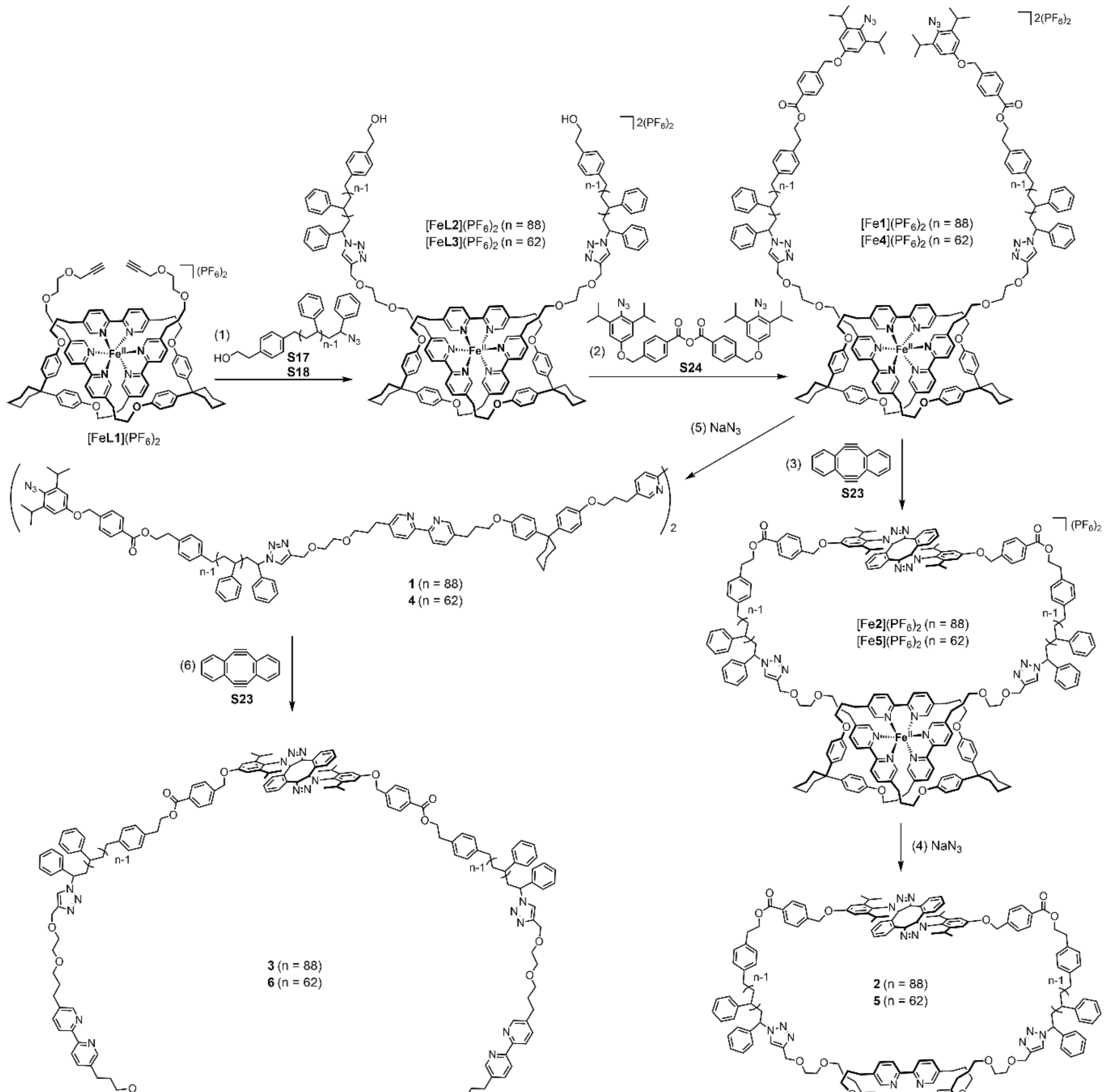

Figure 2. **Synthesis of linear (1, 4), trefoiled (2, 5) and cyclic (3, 6) polymers**. Reaction conditions: (1) [Fe**L1**](PF_6)$_2$ (1 equiv.), **S17** or **S18** (3 equiv.), Cu(MeCN)$_4$PF$_6$ (3 equiv.), TBTA (3 equiv.), MeCN:MeOH:THF = 1:1:2 (v:v:v), 50 °C, 48 h, 93% for [Fe**L2**](PF_6)$_2$, 97% for [Fe**L3**](PF_6)$_2$; (2) [Fe**L2**](PF_6)$_2$ or [Fe**L3**](PF_6)$_2$ (1 equiv.), **S24** (6 equiv.), DMAP (3 equiv.), DCM, r.t., 4 d, 95% for [Fe**1**](PF_6)$_2$, 85% for [Fe**4**](PF_6)$_2$; (3) [Fe**1**](PF_6)$_2$ or [Fe**4**](PF_6)$_2$ (1 equiv.), **S23** (20 equiv.), THF, r.t., 29 h, 98% for [Fe**2**](PF_6)$_2$, 96% for [Fe**5**](PF_6)$_2$; (4) [Fe**2**](PF_6)$_2$ or [Fe**5**](PF_6)$_2$ (1 equiv.), NaN_3 (30 equiv.), DMF, 120 °C, 0.5 h, 44% for **2**, 68% for **5**; (5) [Fe**1**](PF_6)$_2$ or [Fe**4**](PF_6)$_2$ (1 equiv.), NaN_3 (30 equiv.), DMF, 120 °C, 0.5 h, 78% for **1**, 71% for **4**; (6) **1** or **4** (1 equiv.), **S23** (20 equiv.), THF, r.t., 29 h, 44% for **3**, 36% for **6**.

5. Synthetic procedures and characterization details

5.1 Synthesis of [FeL1]$(PF_6)_2$

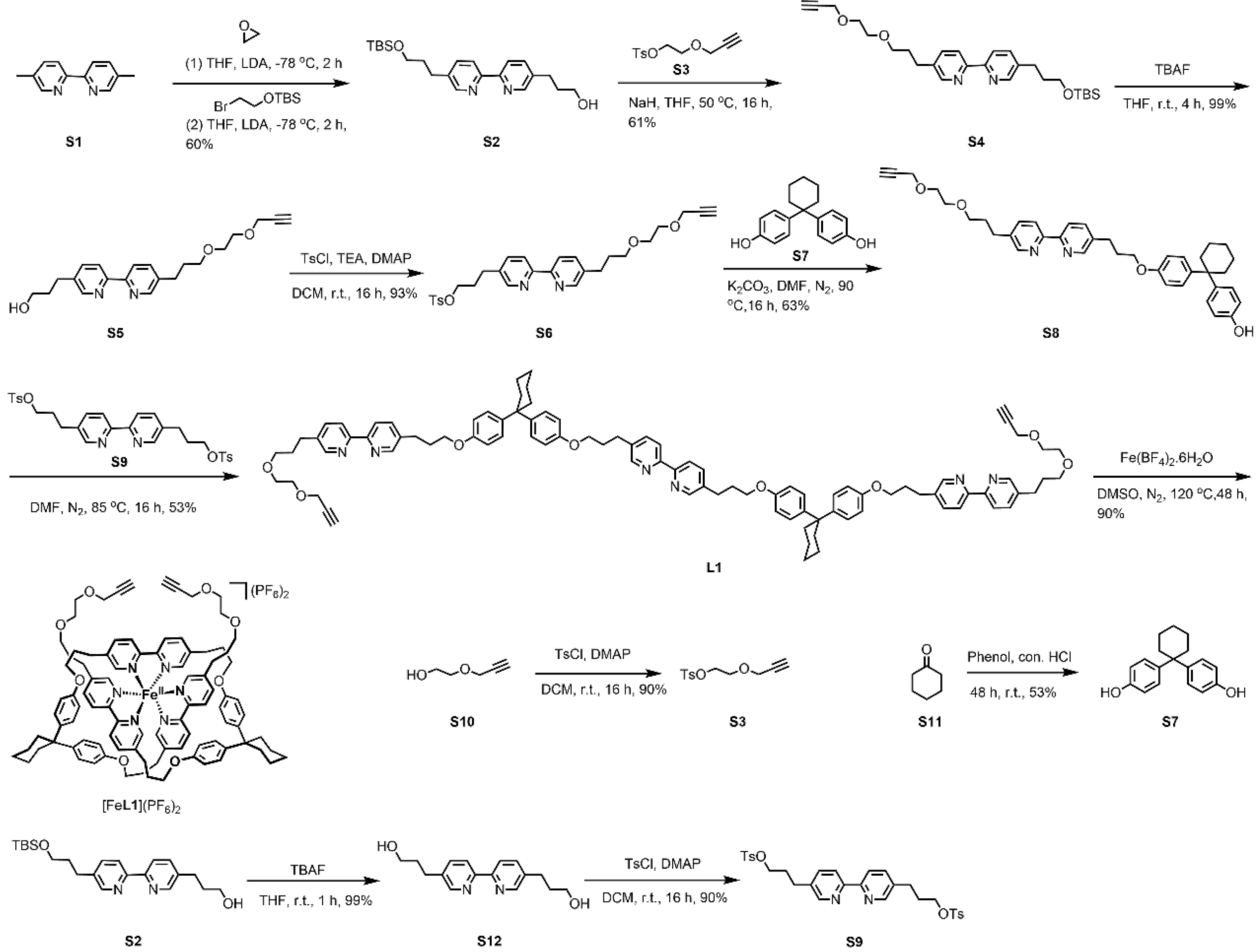

Figure 3. Synthesis of [Fe**L1**]$(PF_6)_2$.

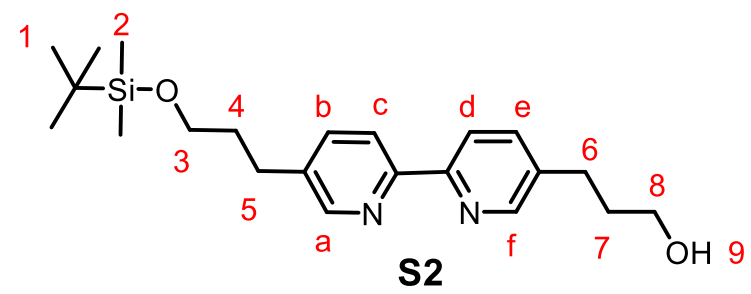

S2

To a stirred solution of **S1** (10.00 g, 35.18 mmol) in dry THF (100 mL) was added LDA (21.1 mL, 2.0 M in THF) dropwise at -78 °C in N_2 atmosphere and stirred the mixture at -78 °C for 1 hour. Then oxirane (12.9 mL, 38.7 mmol) was added to the reaction mixture in one portion at -78 °C. The reaction mixture was stirred at -78 °C for 0.5 h and then wlooped to room temperature and stirred for another 0.5 h. The reaction mixture was cooled to -78 °C and added LDA (21.1 mL, 2.0 M in THF) dropwise and then stirred for 1 hour in N_2 atmosphere. (2-bromoethoxy)(tert-butyl)dimethylsilane (9.26 g, 38.7 mmol) was added to the reaction mixture and stirred at -78 °C for 0.5 h and then wlooped to room temperature and stirred for another 0.5 h. After completion, the reaction was quenched with water (10 mL). The solvent was removed in vacuum and the crude product was purified by silica gel column using DCM and MeOH as eluent (DCM: MeOH = 100: 1) to give **S2** as white solid (8.20 g, yield 60%). mp: 96.1–98.5 °C. ^{1}H NMR (400 MHz, Chloroform-*d*) δ 8.50 (ddd, *J* = 4.3, 2.3, 0.8 Hz, 2H, $H_{a, f}$), 8.26 (ddd, *J* = 8.1, 5.0, 0.9 Hz, 2H, $H_{c, d}$), 7.64 (dt, *J* = 8.1, 2.5 Hz, 2H, $H_{b, e}$), 3.70 (dt, *J* = 6.3, 4.7 Hz, 2H, H_8), 3.65 (t, *J* = 6.1 Hz, 2H, H_3), 2.76 (m, 4H, $H_{5, 6}$), 1.97–1.83 (m, 4H, $H_{4, 7}$), 1.61 (t, *J* = 5.1 Hz, 1H, H_9), 0.91 (s, 9H, H_1), 0.05 (s, 6H, H_2). ^{13}C NMR (101 MHz, Chloroform-*d*) δ 154.23, 153.96, 149.35, 149.24, 137.46, 136.98, 136.90, 136.84, 120.56, 120.48, 61.91, 61.80, 33.99, 33.78, 29.05, 29.01, 25.95, 18.33, -5.29. LR ESI-MS: m/z = 387.29 $[M+H^+]^+$ requires 387.25.

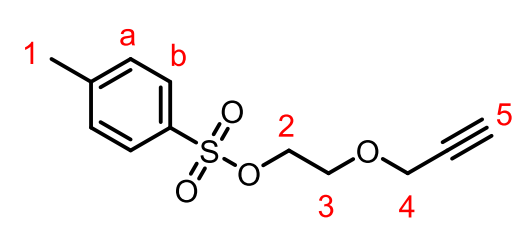

3

To a stirred solution of **S10** (5.00 g, 49.98 mmol), TEA (10.11 g, 99.95 mmol) and DMAP (0.31 g, 2.5 mmol) in dry DCM was added the solution of TsCl (11.43 g, 59.97 mmol) in dry DCM (10 mL) dropwise at 0 °C for 0.5 hour. Then the mixture was wlooped to room temperature and stirred for 16 hours at room temperature. After completion, the reaction was quenched with water (100 mL) and extracted with DCM. The organic phase was dry with anhydrous sodium sulfate and evaporated to dryness. The crude product was purified by silica gel column using EA and PE (EA: PE = 1: 4) as eluent to give **S3** as colorless oily liquid (10.53 g, yield 90%). ^{1}H NMR (400 MHz, Chloroform-*d*) δ 7.80 (d, *J* = 8.3 Hz, 2H, H_b), 7.40–7.30 (m, 2H, H_a), 4.25–4.17 (m, 2H, H_2), 4.12 (d, *J* = 2.4 Hz, 2H, H_4), 3.79–3.69 (m, 2H, H_3), 2.45 (s, 3H, H_1), 2.42 (t, *J* = 2.4 Hz, 1H, H_5). ^{13}C NMR (101 MHz, Chloroform-*d*) δ 144.85, 132.97, 129.83, 127.98, 78.91, 75.04, 68.86, 67.13, 58.36, 21.64.

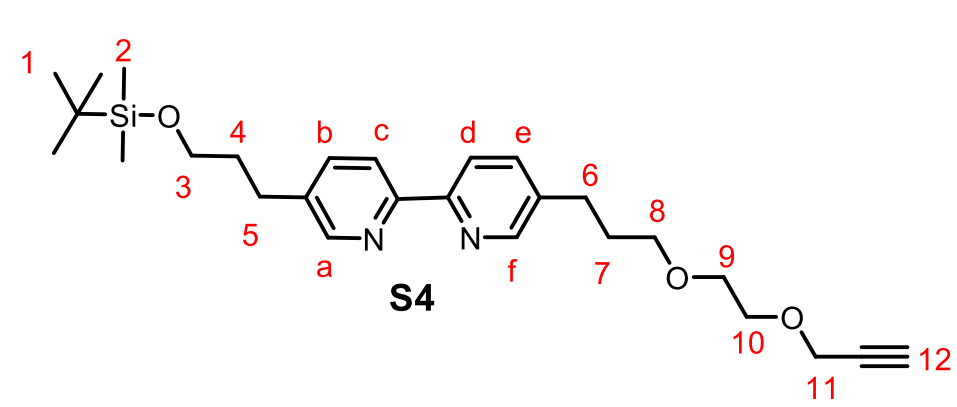

S4

S2 (5.00 g, 12.95 mmol) and NaH (1.56 g, 38.84 mmol) in 250 mL two-neck round bottom bottle was added dry THF (80 mL) at 0 °C in N_2 atmosphere. The reaction mixture was stirred at 0 °C for 10 mins and then **S3** (4.93 g, 19.42 mmol) in dry THF (10 mL) was added to the mixture in one portion. The reaction mixture was wlooped to room temperature and stirred at 70 °C for 16 hours. After completion, the reaction mixture was quenched with water (10 mL) and extracted with dichloride methane (100 mL × 3). The organic phase was dry with anhydrous sodium sulfate and evaporated to dryness. The crude product was purified by silica gel column using DCM and MeOH (DCM: MeOH = 100: 1) as eluent to give **S4** as white solid (3.70 g, yield 61%). mp: 41.7–44.1 °C. ^{1}H NMR (400 MHz, Chloroform-*d*) δ 8.52–8.47 (m, 2H, $H_{a, f}$), 8.26 (ddd, *J* = 8.1, 2.5, 0.8 Hz, 2H, $H_{c, d}$), 7.63 (dt, *J* = 8.2, 2.4 Hz, 2H, $H_{b, e}$), 4.21 (d, *J* = 2.4 Hz, 2H, H_{11}), 3.75–3.68 (m, 2H, H_9), 3.68–3.58 (m, 4H, $H_{3, 10}$), 3.49 (t, *J* = 6.3 Hz, 2H, H_8), 2.74 (q, *J* = 7.7 Hz, 4H, $H_{5, 6}$), 2.43 (t, *J* = 2.4 Hz, 1H, H_{12}), 1.98–1.91 (m, 2H, H_7), 1.90–1.81 (m, 2H, H_4), 0.90 (s, 9H, H_1), 0.05 (s, 6H, H_2). ^{13}C NMR (101 MHz, Chloroform-*d*) δ 154.14, 154.00, 149.33, 149.30, 137.40, 137.05, 136.89, 136.87, 120.49, 120.45, 79.66, 74.53, 70.05, 69.10, 61.89, 58.44, 33.98, 30.81, 29.23, 29.04, 25.95, 18.32, -5.29. LR ESI-MS: *m*/*z* = 469.35 $[M+H^+]^+$ requires 469.29.

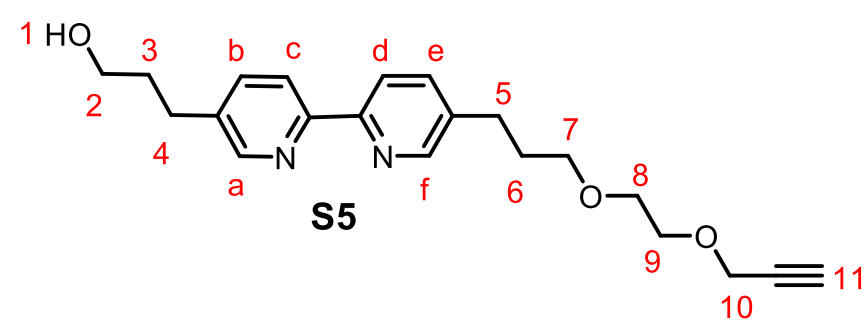

S4 (5.23 g, 11.17 mmol) and tetrabutylammonium fluoride (1 mol/L in THF, 22.34 mL, 22.34 mmol) were dissolved in THF (50 mL) and stirred at room temperature for 2 hours. After completion, the solvent was evaporated under reduced pressure, and the crude product was extracted with DCM and water. The organic phase was washed four times with water and one time with saturated sodium chloride aqueous and dry with anhydrous sodium sulfate. The organic solvent was evaporated under vacuum to give **S5** as waxy white solid. The crude product was used directly without further purification. ^{1}H NMR (400 MHz, Chloroform-*d*) δ 8.57–8.46 (m, 2H, $H_{a, f}$), 8.26 (ddd, *J* = 8.1, 2.6, 0.8 Hz, 2H, $H_{c, d}$), 7.64 (ddd, *J* = 8.1, 2.3, 0.9 Hz, 2H, $H_{b, e}$), 4.22 (d, *J* = 2.4 Hz, 2H, H_{10}), 3.75–3.66 (m, 4H, $H_{2, 8}$), 3.65–3.59 (m, 2H, H_9), 3.50 (t, *J* = 6.3 Hz, 2H, H_7), 2.83–2.71 (m, 4H, $H_{4, 5}$), 2.43 (t, *J* = 2.4 Hz, 1H, H_{11}), 2.01–1.87 (m, 4H, $H_{3, 6}$). ^{13}C NMR (101 MHz, Chloroform-*d*) δ 154.17, 154.05, 149.32, 149.25, 137.15, 137.03, 136.93, 136.86, 120.58, 120.54, 74.55, 70.06, 70.04, 69.11, 61.80, 58.45, 33.77, 30.80, 29.24, 29.00. LR ESI-MS: *m*/*z* = 355.28 $[M+H^+]^+$ requires 355.20.

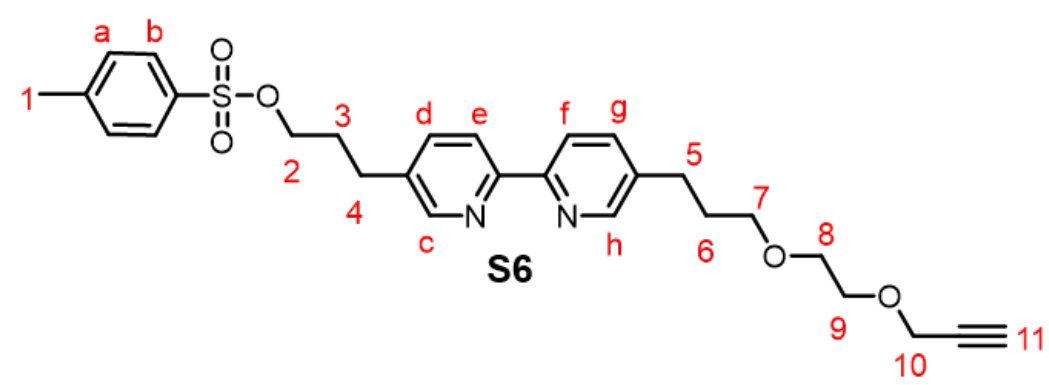

To a stirred solution of **S5** (4.00 g, 11.29 mmol), TEA (2.28 g, 22.59 mmol), DMAP (0.14 g, 1.13 mmol) in dry DCM was added the solution of TsCl (3.23 g, 16.94 mmol) in dry DCM (10 mL)

dropwise at 0 °C and stirred for 0.5 hour. Then the mixture was wlooped to room temperature and stirred for 16 hours at room temperature. After completion, the reaction was quenched with water (50 mL) and extracted with ethyl acetate. The organic phase was dry with anhydrous sodium sulfate and evaporated to dryness. The crude product was purified by silica gel column using EA and PE (EA: PE = 1.5: 1) as eluent to give **S6** as brown solid (5.30 g, yield 93%). ^{1}H NMR (400 MHz, Chloroform-*d*) δ 8.52–8.48 (m, 1H, H_c), 8.42–8.37 (m, 1H, H_h), 8.24 (ddd, *J* = 9.1, 8.1, 0.8 Hz, 2H, $H_{e,f}$), 7.81 –7.76 (m, 2H, H_b), 7.64 (dd, *J* = 8.1, 2.3 Hz, 1H, H_g), 7.52 (dd, *J* = 8.1, 2.3 Hz, 1H, H_d), 7.37–7.31 (m, 2H, H_a), 4.22 (d, *J* = 2.4 Hz, 2H, H_{10}), 4.06 (t, *J* = 6.1 Hz, 2H, H_2), 3.74–3.68 (m, 2H, H_8), 3.65 –3.60 (m, 2H, H_9), 3.50 (t, *J* = 6.3 Hz, 2H, H_7), 2.74 (ddd, *J* = 17.2, 8.5, 6.7 Hz, 4H, $H_{4,5}$), 2.43 (s, 4H, $H_{1,11}$), 2.05–1.90 (m, 4H, $H_{3,6}$). ^{13}C NMR (101 MHz, Chloroform-*d*) δ 154.46, 153.84, 149.33, 149.14, 144.91, 137.30, 136.95, 136.80, 135.56, 132.97, 129.91, 127.89, 120.58, 120.55, 74.55, 70.05, 69.15, 69.11, 58.45, 30.81, 30.11, 29.24, 28.55, 21.64. LR ESI-MS: *m*/*z* = 509.30 $[M+H^+]^+$ requires 509.21.

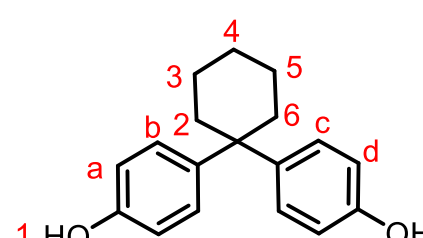

S7

S11 (10.00 g, 0.1019 mol) was added to a 250 mL round bottom flash followed by the addition of concentrated HCl (50 mL). Then phenol (21.1 g, 0.2242 mol) was added to the mixture and stirred at room temperature for 24 hours. After completion, the reaction mixture was neutralized with saturated aqueous solution of $NaHCO_3$ and extracted with DCM. The organic phase was dry with anhydrous Na_2SO_4 and evaporated to dryness. The crude product was purified by silica gel column using ethyl acetate and petroleum ether (1:2) as eluent to give **S7** as white solid (14.50 g, yield 53%). ^{1}H NMR (400 MHz, DMSO-*d*$_6$) δ 9.09 (s, 2H, H_1), 7.07–6.99 (m, 4H, $H_{b,c}$), 6.69–6.59 (m, 4H, $H_{a,d}$), 2.12 (br, 4H, $H_{2,6}$), 1.41 (br, 6H, $H_{3,4,5}$). ^{13}C NMR (101 MHz, DMSO-*d*$_6$) δ 155.07, 139.51, 128.02, 115.26, 44.60, 37.05, 26.36, 23.02. LR ESI-MS: *m*/*z* = 268.27 $[M]^+$ requires 268.15.

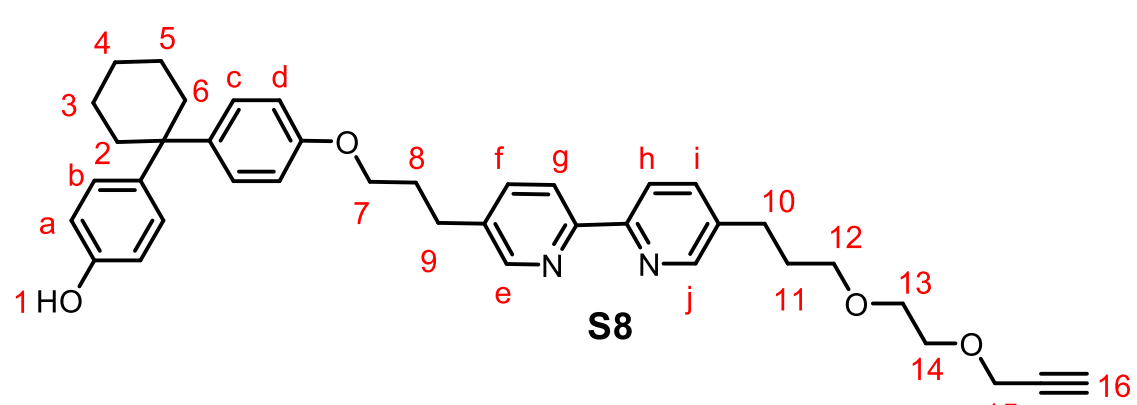

S8

S7 (5.38 g, 20.07 mmol) and K_2CO_3 (4.16 g, 30.11 mmol) were dissolved in anhydrous DMF (100 mL) under nitrogen atmosphere in a 250 mL two-necked round bottom flask and stirred at 90 °C for 0.5 h. The solution of **S6** (5.10 g, 10.04 mmol) in dry DMF (20 mL) was added dropwise to the reaction mixture for 5 hours at 90 °C. The reaction mixture was stirred at 90 °C for another 16 hours. After completion, the reaction mixture was diluted with water (150 mL) and extracted with DCM (3 × 150 mL). The organic layer was combined and washed with water (3 × 150 mL) and brine. The organic phase was dried with anhydrous sodium sulfate and evaporated to dryness. The

crude product was purified by normal phase column using EA and PE (EA: PE = 2: 1) as eluent to give **S8** as pale-brown oil (3.81 g, yield 63%). ^{1}H NMR (400 MHz, Chloroform-*d*) δ 8.50 (dd, *J* = 4.4, 2.2 Hz, 2H, $H_{e,j}$), 8.29–8.22 (m, 2H, $H_{g,h}$), 7.63 (ddd, *J* = 8.4, 6.4, 2.3 Hz, 2H, $H_{f,i}$), 7.19–7.06 (m, 4H, $H_{b,c}$), 6.82–6.70 (m, 4H, $H_{a,d}$), 4.22 (d, *J* = 2.4 Hz, 2H, H_{13}), 3.94 (t, *J* = 6.0 Hz, 2H, H_5), 3.74–3.68 (m, 2H, H_{11}), 3.64–3.59 (m, 2H, H_{12}), 3.49 (t, *J* = 6.3 Hz, 2H, H_{10}), 2.88–2.81 (m, 2H, H_7), 2.81–2.72 (m, 2H, H_8), 2.43 (t, *J* = 2.4 Hz, 1H, H_{14}), 2.20 (t, *J* = 5.4 Hz, 4H, H_2), 2.06–2,13 (m, 2H, H_6), 1.99–1.90 (m, 2H, H_9), 1.46–1.56 (m, 6H, $H_{3,4}$). ^{13}C NMR (101 MHz, Chloroform-*d*) δ 156.38, 154.09, 153.95, 153.48, 149.27, 141.21, 140.92, 137.24, 137.07, 136.90, 128.21, 128.09, 120.70, 115.03, 114.12, 74.57, 70.04, 69.11, 66.43, 58.45, 45.03, 37.39, 30.78, 30.57, 29.22, 26.44, 22.93. LR ESI-MS: *m*/*z* = 605.95 $[M]^+$ requires 605.34.

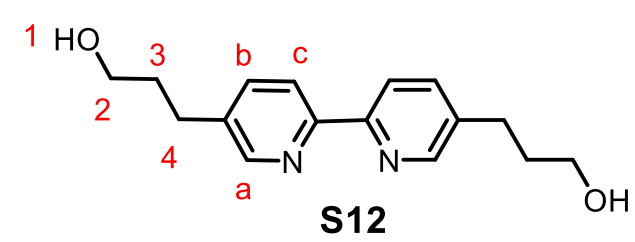

S12

S2 (1.00 g, 2.59 mmol) and tetrabutylammonium fluoride (1 mol/L in THF, 2.6 mL, 2.59 mmol) were dissolved in THF (30 mL) and stirred at room temperature for 2 hours. After completion, the solvent was evaporated under reduced pressure, and the crude product was extracted with DCM and water. The organic phase was washed four times with water and one time with saturated sodium chloride aqueous and dry with anhydrous sodium sulfate. The organic solvent was evaporated under vacuum to give **S12** as waxy white solid. The crude product was used directly without further purification.

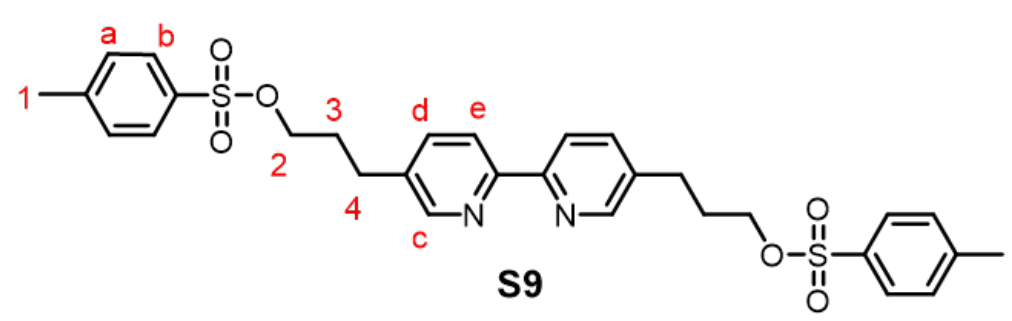

S9

To a stirred solution of **S12** (1.00 g, 3.67 mmol), TEA (1.12 g, 11.02 mmol) and DMAP (44.89 mg, 0.37 mmol) in dry DCM was added the solution of TsCl (1.75 g, 9.19 mmol) in dry DCM (5 mL) dropwise at 0 °C for 0.5 hour. Then the mixture was wlooped to room temperature and stirred for 16 hours at room temperature. After completion, the reaction was quenched with water (50 mL) and extracted with DCM. The organic phase was dry with anhydrous sodium sulfate and evaporated to dryness. The crude product was purified by silica gel column using EA and PE (EA: PE = 1.5: 1) as eluent to give **S9** as white solid (1.80 g, 91%). mp: 166.7–168.1 °C. ^{1}H NMR (300 MHz, Chloroform-*d*) δ 8.44 (d, *J* = 2.2 Hz, 2H, H_d), 8.25 (d, *J* = 8.1 Hz, 2H, H_e), 7.88–7.77 (m, 4H, H_b), 7.56 (dd, *J* = 8.1, 2.3 Hz, 2H, H_c), 7.37 (d, *J* = 8.0 Hz, 4H, H_2), 4.09 (t, *J* = 6.1 Hz, 4H, H_2), 2.84–2.70 (m, 4H, H_4), 2.47 (s, 6H, H_1), 2.10–1.98 (m, 4H, H_3). ^{13}C NMR (101 MHz, Chloroform-*d*) δ 154.28, 149.18, 144.91, 136.79, 135.72, 132.98, 129.91, 127.89, 120.58, 69.13, 30.12, 28.57, 21.64. LR ESI-MS: *m*/*z* = 581.32 $[M+H^+]^+$ requires 581.18.

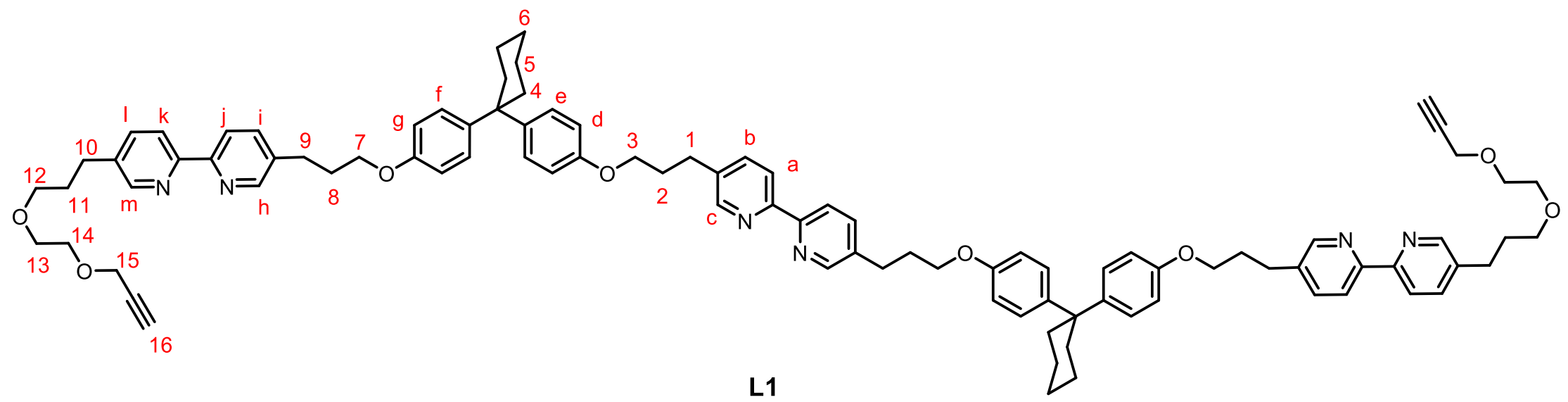

L1

S9 (1.82 g, 3.14 mmol) was added to a solution of **S8** (3.80 g, 6.28 mmol) and K_2CO_3 (2.60 g, 18.84 mmol) in anhydrous DMF (30 mL) under nitrogen atmosphere in a 50 mL round bottom flask. The reaction mixture was stirred at 90 °C for 16 hours. After completion, the mixture was diluted with water (50 mL) and extracted with DCM (3 × 100 mL). The organic layer was combined and wash with water (3 × 100 mL) and brine. The organic phase was dried over sodium sulfate and evaporated to dryness. The crude product was dissolved in DCM and precipitated from MeOH. Filtration and wash the filter cake with MeOH and redissolved the filter cake in DCM. This process was repeated three times to give **L1** as white solid (2.40 g, yield 53%). mp: 159.5–161.2 °C. ^{1}H NMR (500 MHz, Chloroform-d) δ 8.57–8.47 (m, 6H, $H_{c, h, m}$), 8.35–8.25 (m, 6H, $H_{a, j, k}$), 7.66 (dt, J = 8.2, 1.9 Hz, 6H, $H_{b, i, l}$), 7.22–7.11 (m, 8H, $H_{e, f}$), 6.86–6.76 (m, 8H, $H_{d, g}$), 4.22 (d, J = 2.3 Hz, 4H, H_{15}), 3.94 (t, J = 6.0 Hz, 8H, $H_{3, 7}$), 3.76–3.67 (m, 4H, H_{13}), 3.65–3.58 (m, 4H, 4H, H_{14}), 3.49 (t, J = 6.3 Hz, 4H, 4H, H_{12}), 2.86 (t, J = 7.6 Hz, 8H, $H_{1, 9}$), 2.76 (t, J = 7.6 Hz, 4H, H_{10}), 2.44 (t, J = 2.4 Hz, 2H, H_{16}), 2.21 (t, J = 5.5 Hz, 8H, H_4), 2.11 (dt, J = 8.5, 6.2 Hz, 8H, $H_{2, 8}$), 2.00–1.90 (m, 4H, H_{11}), 1.59–1.44 (m, 12H, $H_{5, 6}$). ^{13}C NMR (126 MHz, Chloroform-d) δ 156.41, 153.89, 153.85, 153.71, 149.20, 149.14, 141.16, 137.31, 137.17, 137.14, 137.12, 136.96, 128.11, 120.68, 114.05, 79.64, 74.57, 70.03, 70.01, 69.09, 66.36, 66.34, 58.44, 45.04, 37.39, 30.77, 30.56, 29.22, 26.42, 22.91. HR ESI-MS: m/z = 1445.8004 $[M+H^+]^+$ requires 1445.7994.

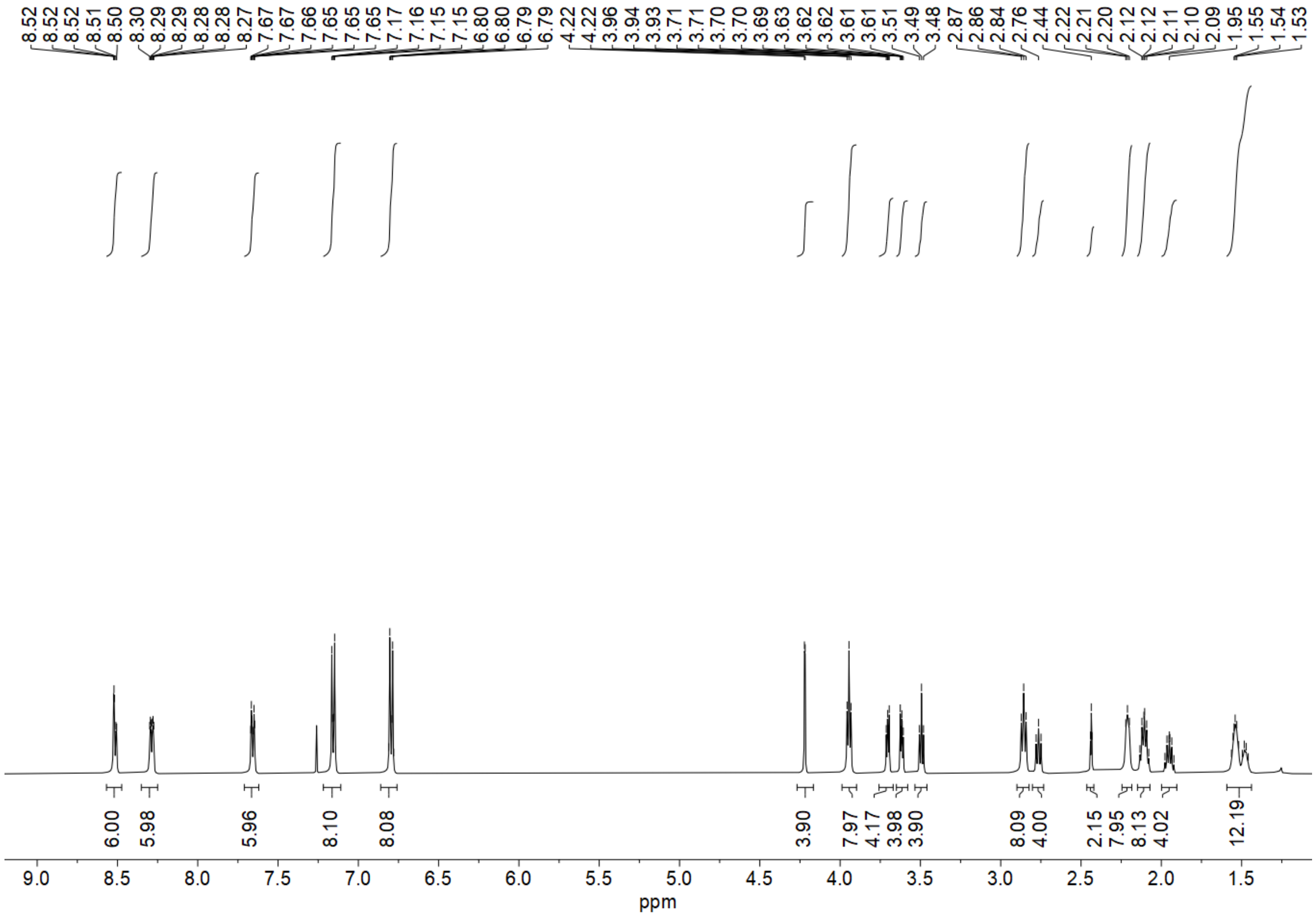

Figure 4. ^{1}H NMR spectra (500 MHz, $CDCl_3$, 298 K) of **L1**.

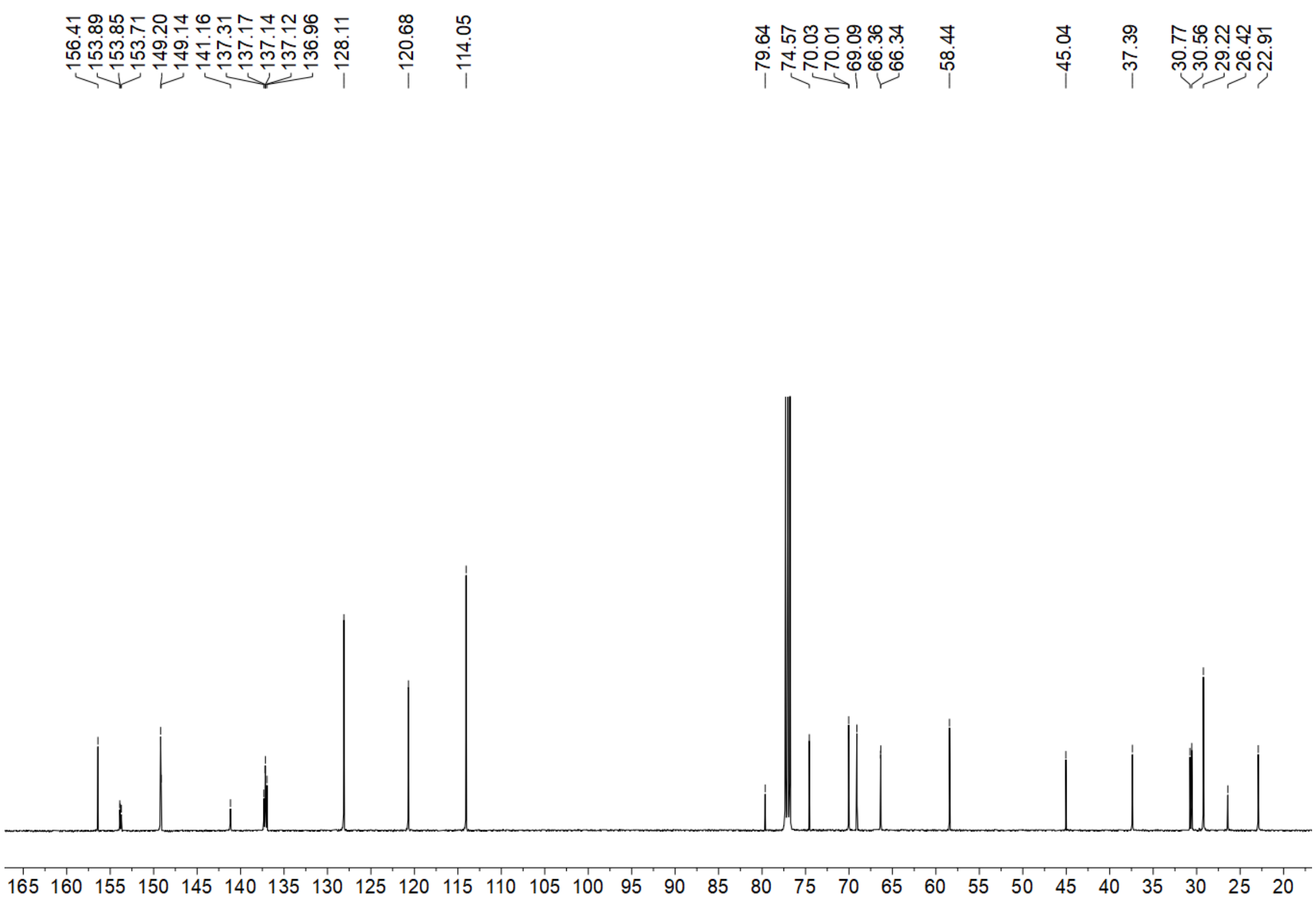

Figure 5. ^{13}C NMR spectra (126 MHz, $CDCl_3$, 298 K) of **L1**.

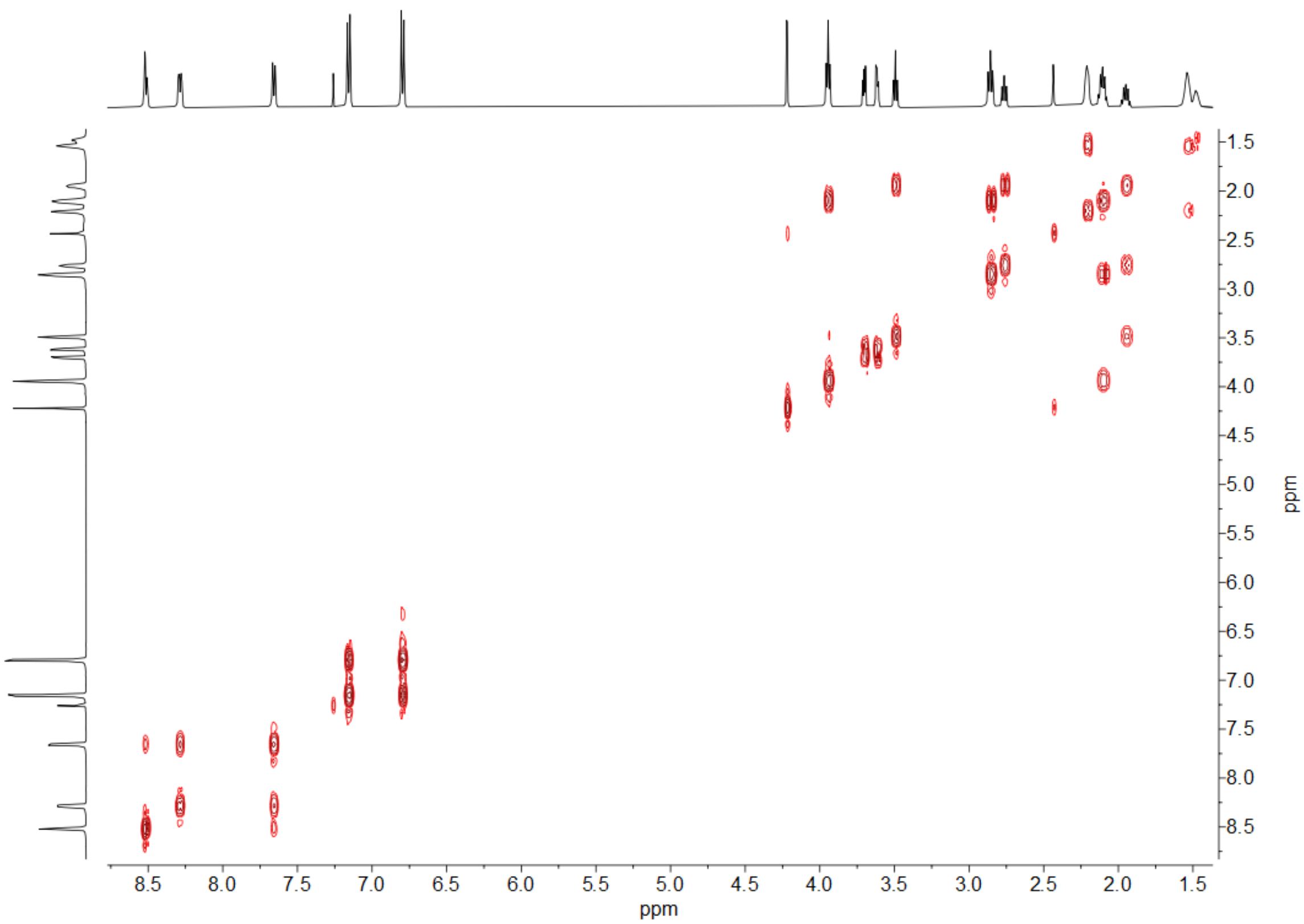

Figure 6. ^{1}H-^{1}H COSY spectrum (500 MHz, $CDCl_3$, 298 K) of **L1**.

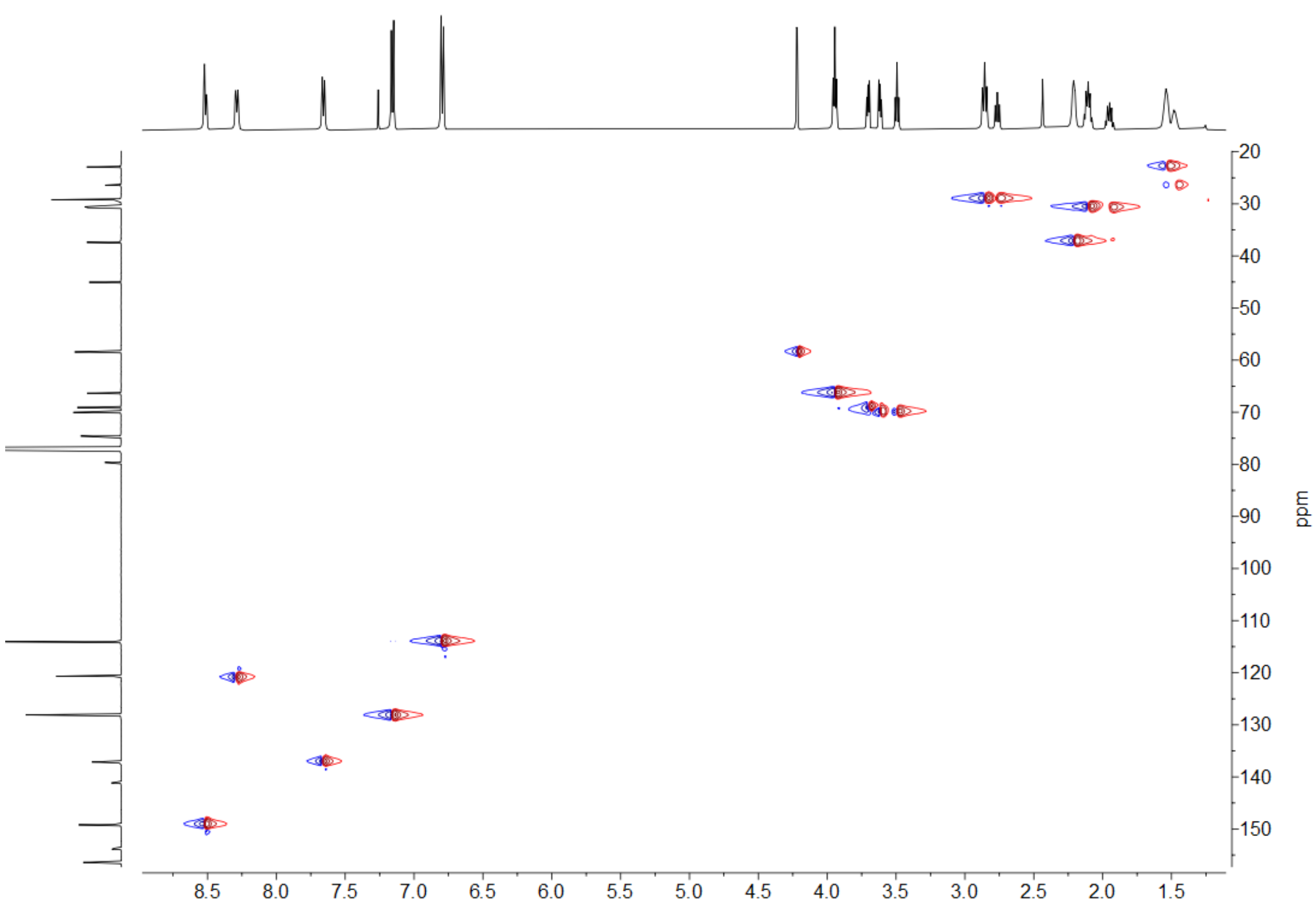

Figure 7. ^{1}H-^{13}C HSQC spectrum (500 MHz, $CDCl_3$, 298 K) of **L1**.

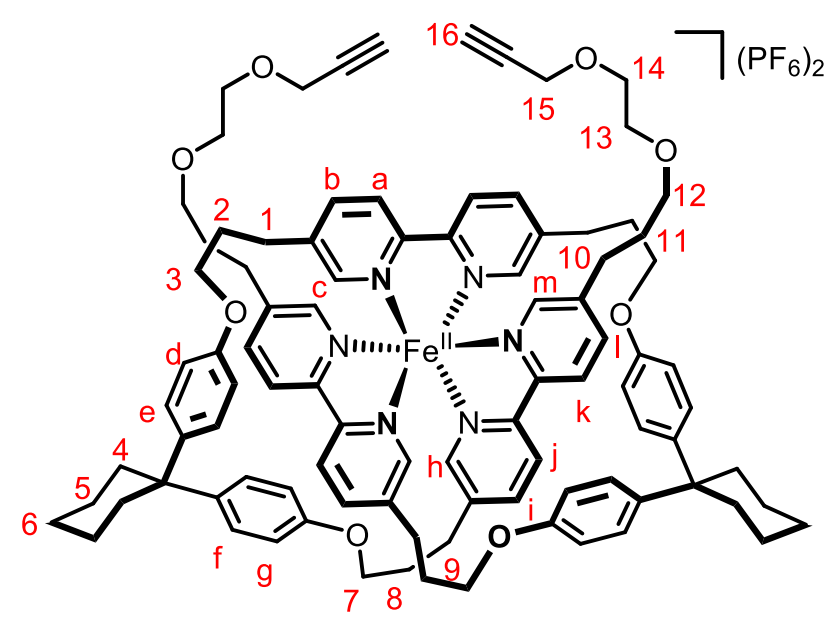

[Fe**L1**]$(PF_6)_2$

L1 (50 mg, 34.61 μmol) and $Fe(BF_4)_2\cdot 6H_2O$(4.39 mg, 34.61 μmol) in DMSO (6 mL) was heated to 120 °C in a sealed vial for 24 h in nitrogen atmosphere with stirring. After completion, the resulting purple solution was cooled to room temperature, and diluted with saturated aqueous solution of KPF_6 until a precipitate formed. The solid was isolated by filtration onto celite, and washed with water in excess. The remaining solid was dissolved in acetonitrile, the solvent was removed under vacuum to give [Fe**L1**]$(PF_6)_2$ as red-purple solid (56 mg, yield 90%). mp > 300 °C. ^{1}H NMR (500 MHz, Acetonitrile-d_3) δ 8.33 (d, J = 8.3 Hz, 2H, H_a), 7.92 (dd, J = 8.3, 1.9 Hz, 2H, H_b), 7.71–7.62 (m, 10H, $H_{i, e, f}$), 7.50 (dd, J = 8.2, 1.9 Hz, 2H, H_l), 7.01 (d, J = 1.8 Hz, 2H, H_m), 6.76 (d, J = 1.8 Hz, 2H, H_c), 6.60–6.49 (m, 8H, $H_{d, g}$), 6.38 (d, J = 1.7 Hz, 2H, H_h), 5.96 (d, J = 8.3 Hz, 2H, H_j), 5.64 (d, J = 8.2 Hz, 2H, H_k), 4.15 (d, J = 2.4 Hz, 4H, H_{15}), 3.64–3.53 (m, 8H, $H_{7, 13}$), 3.42–3.39 (m, 4H, H_{14}), 3.18–3.07 (m, 4H, H_3), 2.77–2.68 (m, 6H, $H_{16, 1}$), 2.64–2.32 (m, 20H, $H_{4, 8, 10, 12}$), 2.11–1.98 (m, 4H, H_{11}), 1.71–1.55 (m, 20H, $H_{2, 5, 6, 9}$). ^{13}C NMR (126 MHz, Acetonitrile-d_3) δ 156.47, 155.53, 155.49, 155.07, 154.20, 152.48, 151.96, 151.57, 141.78, 140.65, 139.68, 139.36, 137.76, 137.50, 137.45, 126.40, 122.93, 122.66, 122.57, 114.12, 79.13, 73.97, 68.75, 68.09, 67.87, 62.73, 62.65, 56.94, 43.55, 33.45, 33.41, 29.05, 28.40, 27.48, 26.60, 26.44, 25.28, 21.88. HR ESI-MS: m/z = 1645.6895 $[M-PF_6]^+$ requires 1645.6907.

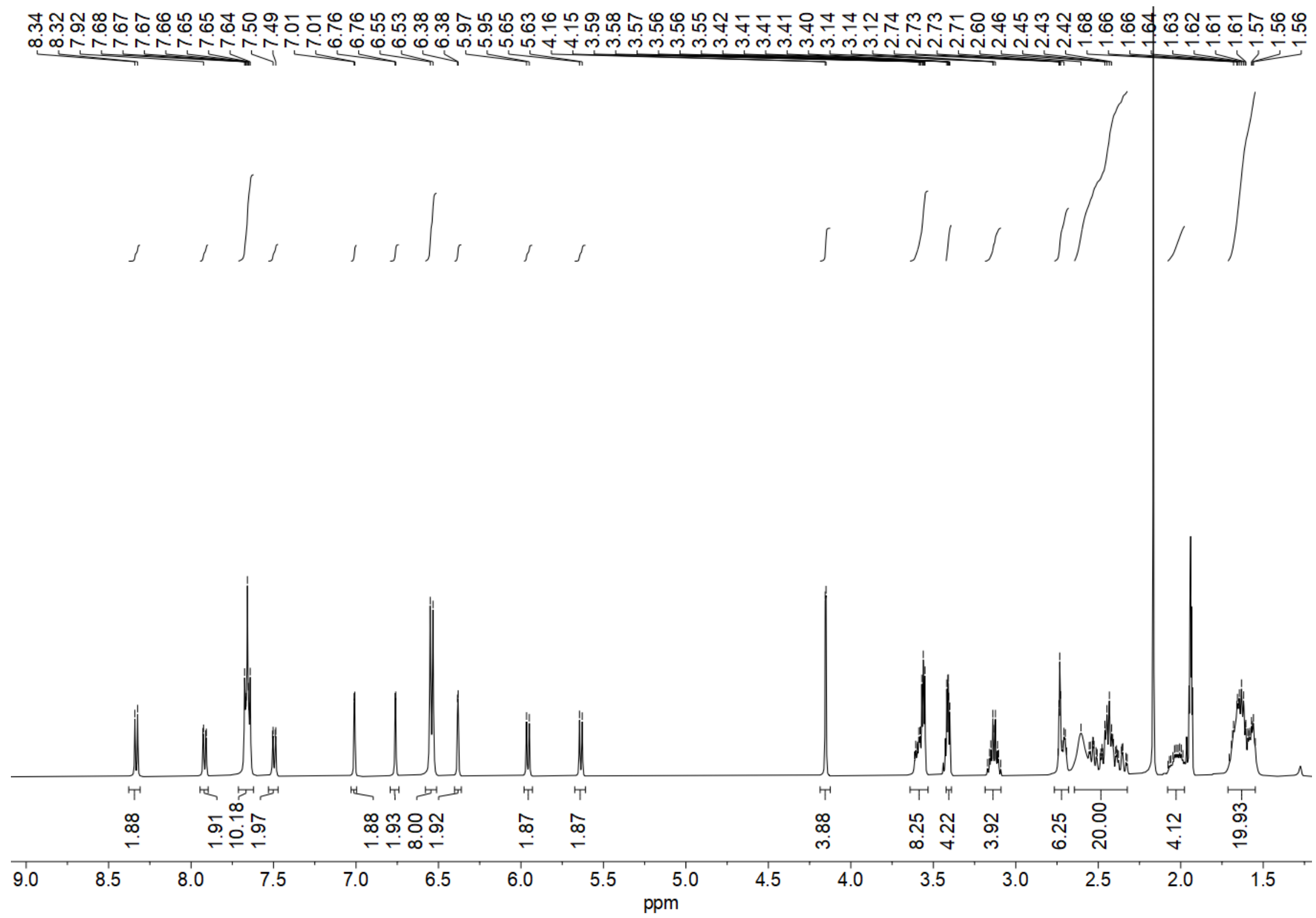

Figure 8. ^{1}H NMR spectra (500 MHz, MeCN-d_3, 298 K) of [Fe**L1**]$(PF_6)_2$.

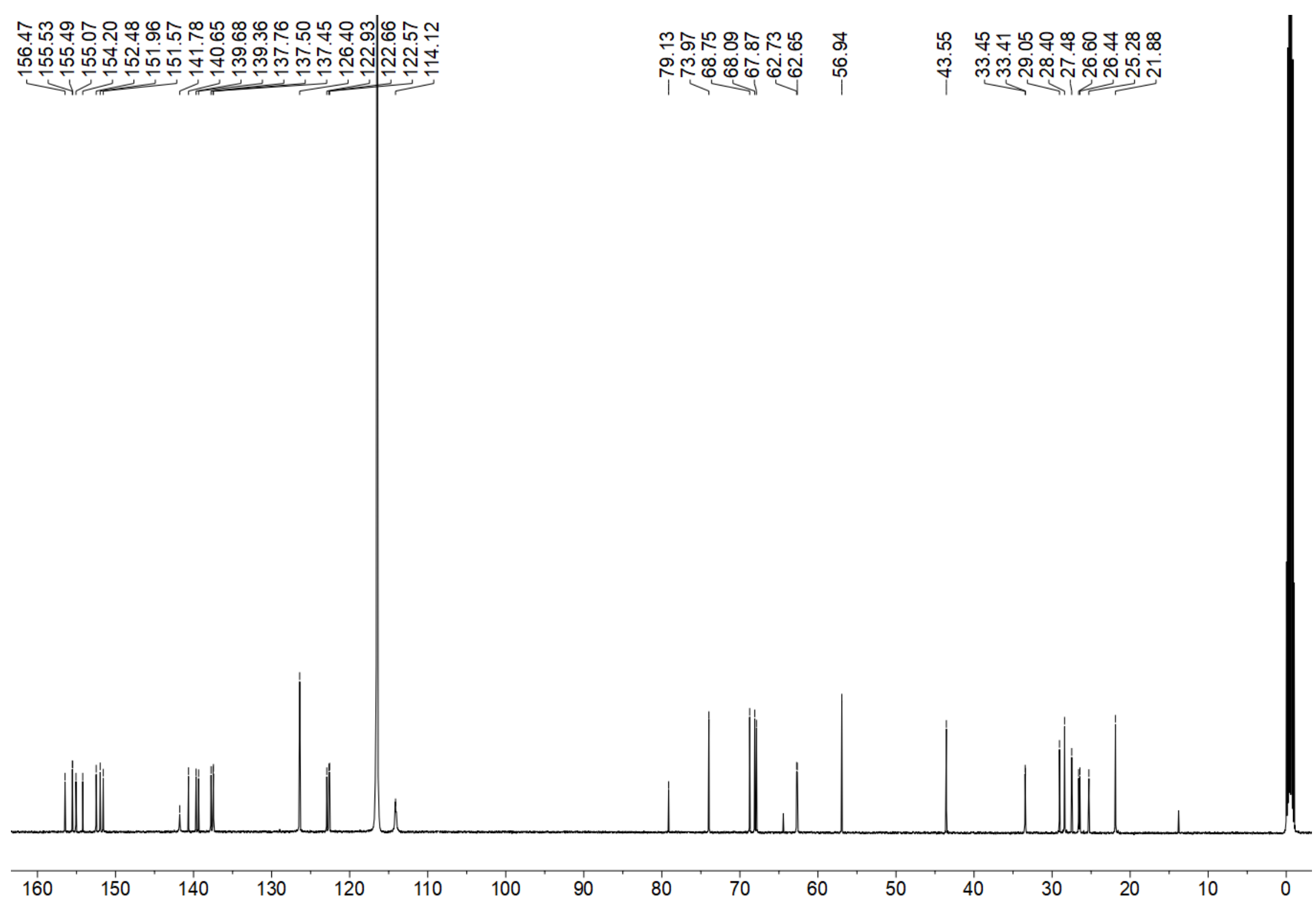

Figure 9. ^{13}C NMR spectra (126 MHz, MeCN-d_3, 298 K) of [Fe**L1**]$(PF_6)_2$.

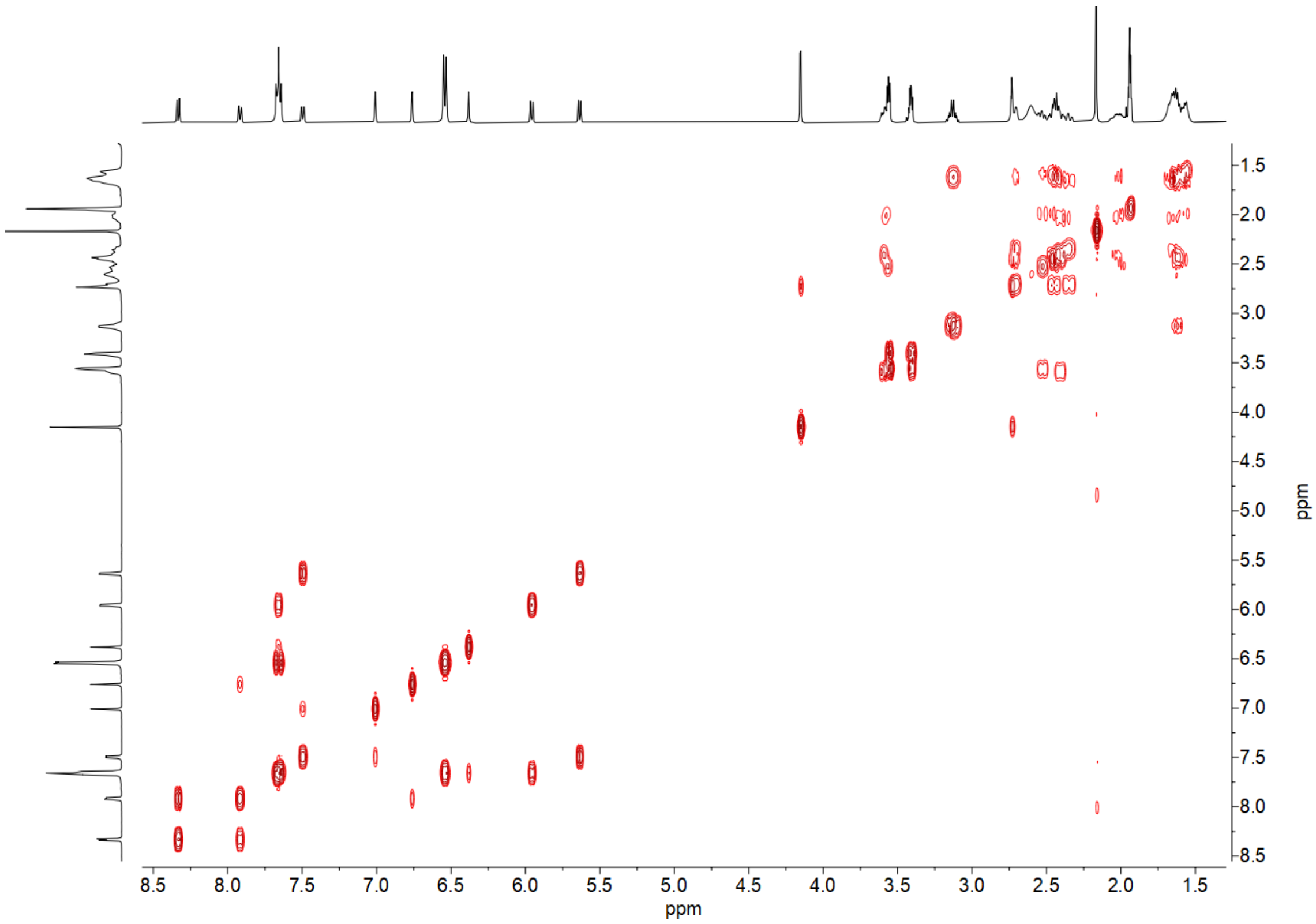

Figure 10. ^{1}H-^{1}H COSY spectrum (500 MHz, MeCN-d_3, 298 K) of [Fe**L1**](PF_6)$_2$.

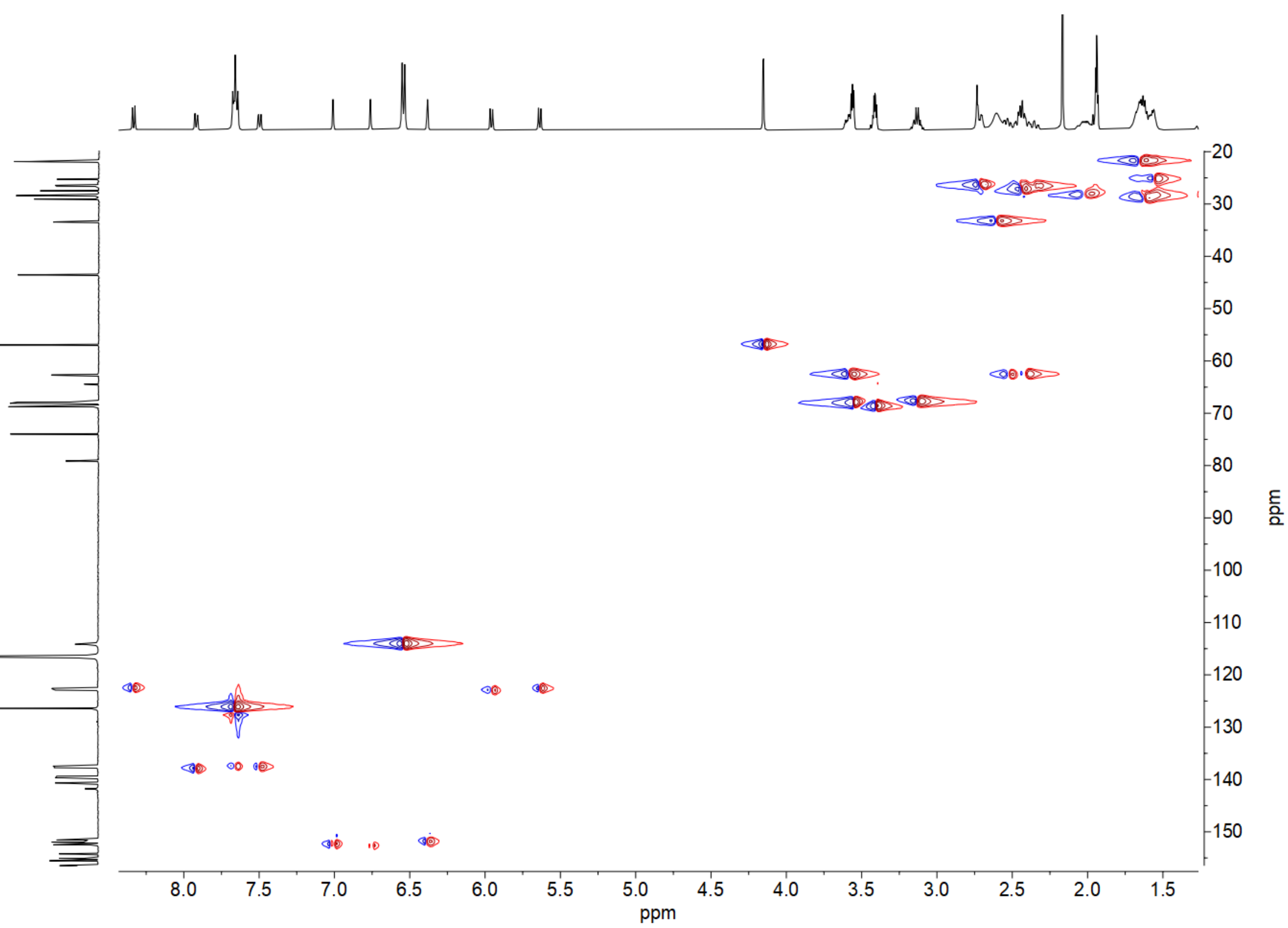

Figure 11. ^{1}H-^{13}C HSQC spectrum (500 MHz, MeCN-d_3, 298 K) of [Fe**L1**](PF_6)$_2$.

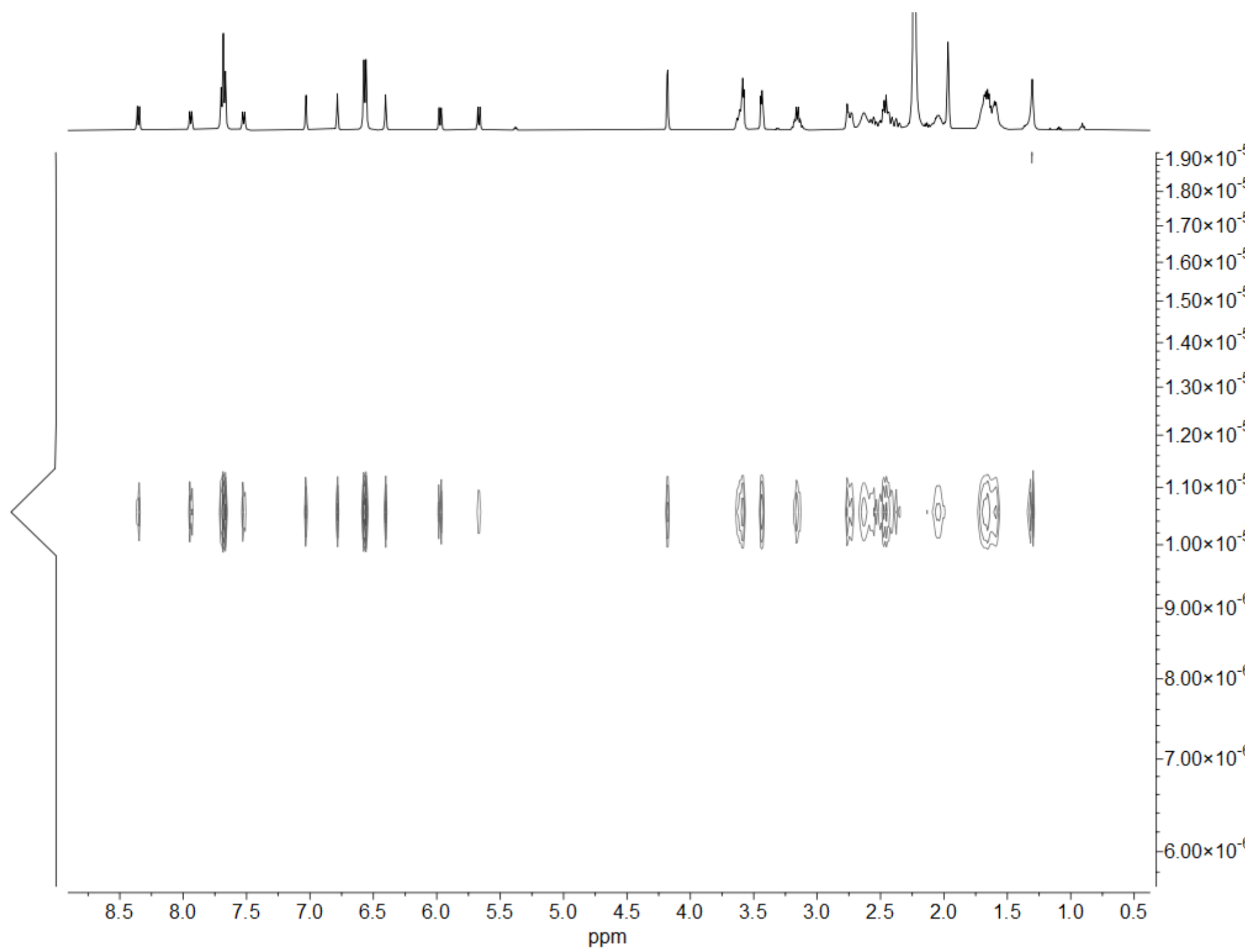

Figure 12. DOSY experiment spectrum (500 MHz, MeCN-d_3, 298 K) of [Fe**L1**]$(PF_6)_2$. $D = 1.06 \times 10^{-6}$ $cm^2 \cdot s^{-1}$.

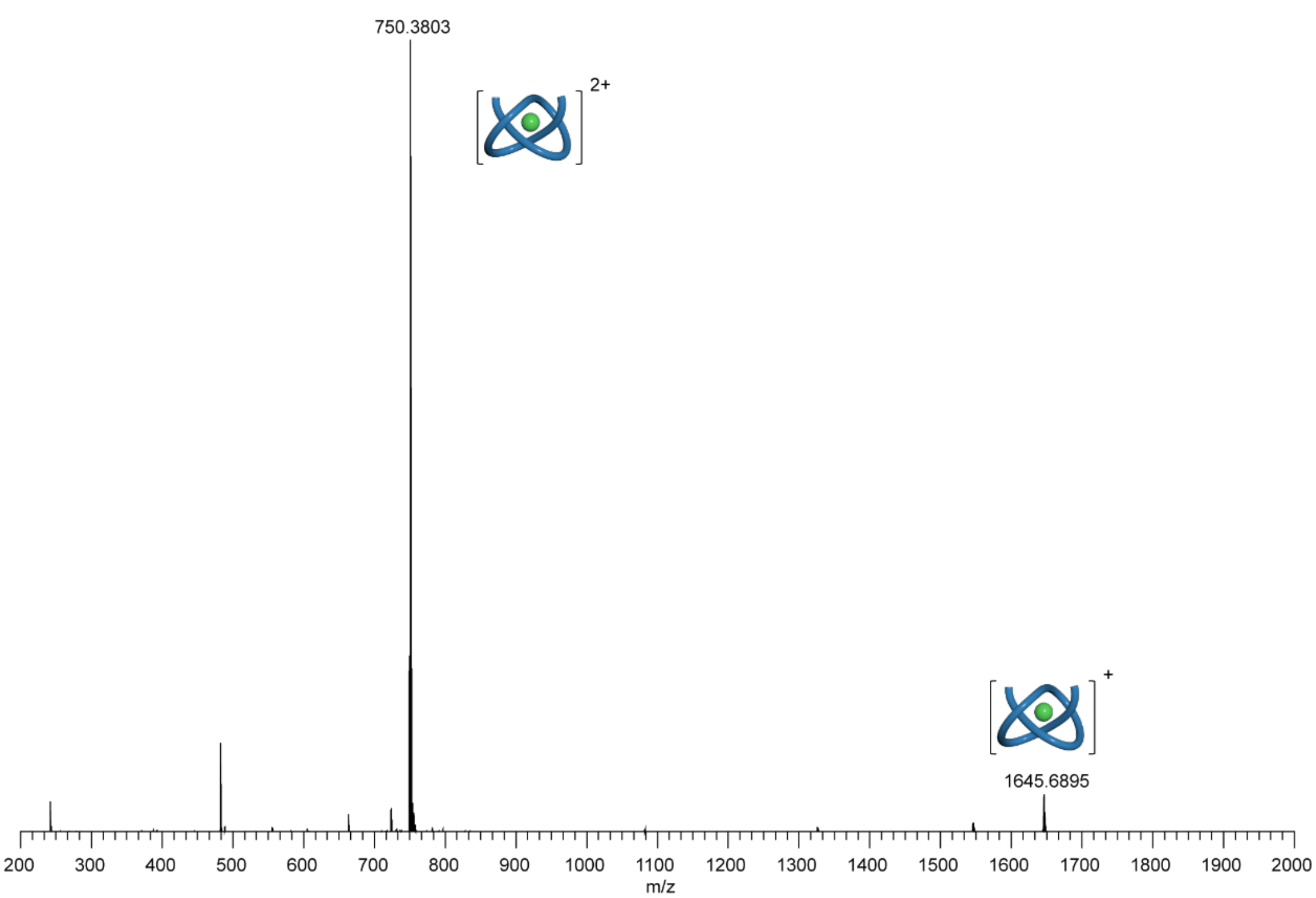

Figure 13. HR ESI-MS of [Fe**L1**]$(PF_6)_2$. Calculated peaks (*m*/*z*): 750.3633 $[M\text{-}2PF_6]^{2+}$; 1645.6907 $[M\text{-}PF_6]^{2+}$.

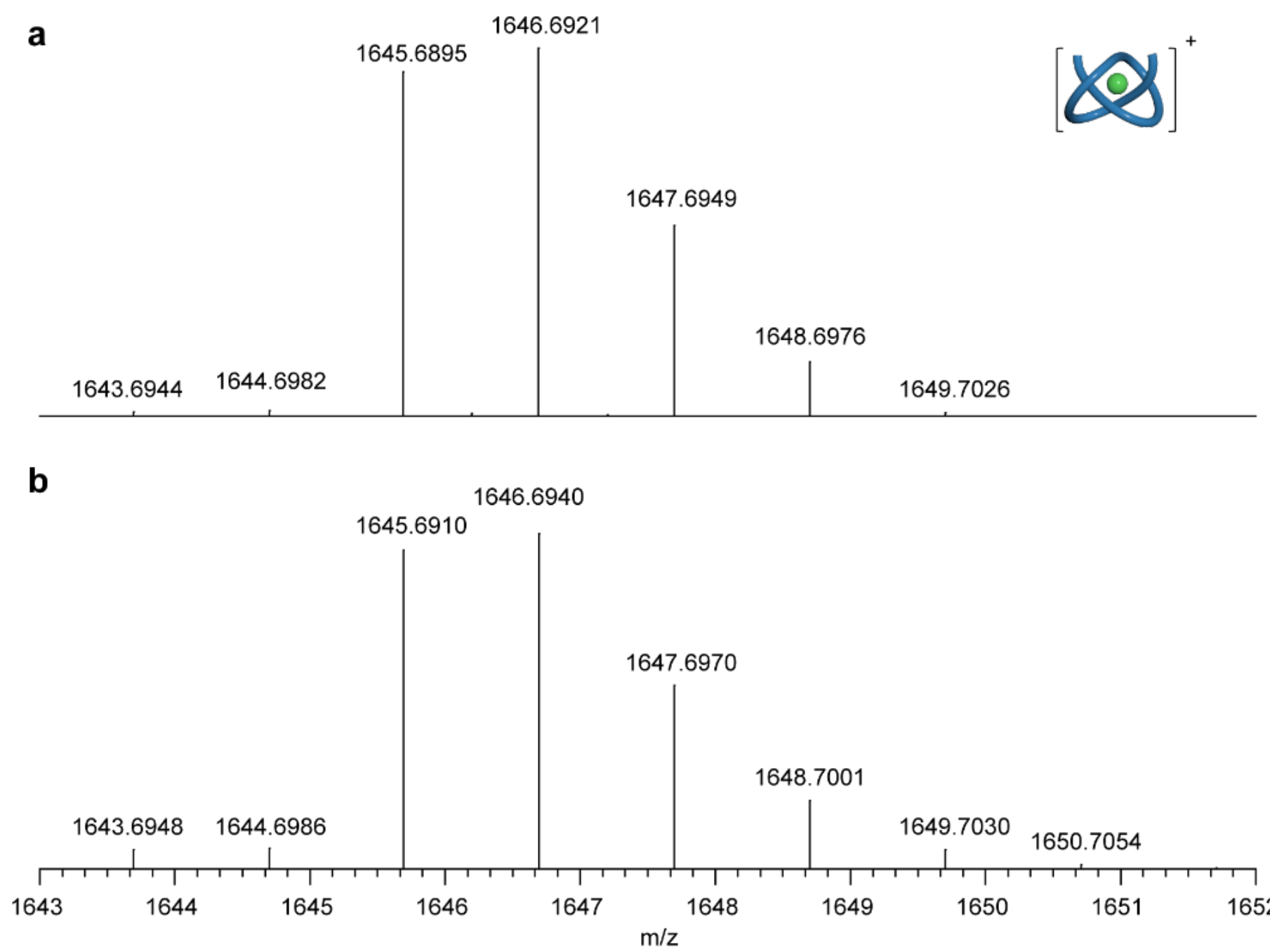

Figure 14. HR ESI-MS of $[M-PF_6]^+$ peak of [Fe**L1**]$(PF_6)_2$. (a) experimental spectrum; (b) calculated spectrum.

5.2 X-ray crystal structure information of [FeL1]$(PF_6)_2$

Slow diffusion of ethyl ether into an acetonitrile solution of [Fe**L1**]$(PF_6)_2$ produced red crystals suitable for X-ray crystallography. X-Ray data for compound [Fe**L1**]$(PF_6)_2$ were collected at a temperature of 173 K using Bruker D8 Venture MetalJet diffractometer equipped with a photon II CCD area detector and operated at 200 W (70 kV, 2.86 mA) to generate gallium Kα radiation (λ = 1.34139 Å). The crystal structures are solved by Direct Method and refined on F^2 by full-matrix least-squares using the Shelxtl-2018 program in the Olex2 GUI platform.

Discussion of the B Alert in the CheckCIF report:

In the CheckCIF report of [Fe**L**$_1$]$(PF_6)_2$, an alert level B exist (*PLAT220_ALERT_2_B Non Solvent Resd 1 C Ueq(max)/Ueq(min) Range 6.2 Ratio*). This alert is due to substantial disorder in the side chains (C2-C8 in the .cif file) attached to the rigid bipyridine units within the structure. Dynamically disordered side-chains tethered to a relatively rigid backbone often exhibit such unusual ratios. Consequently, high thermal motion of these atoms is expected.

Table 2. Crystallographic information of [Fe**L1**]$(PF_6)_2$

	[Fe**L1**]$(PF_6)_2$
Empirical formula	$C_{94}H_{104}F_{12}FeN_6O_8P_2$
Formula weight	1791.62
Temperature/K	173
Wavelength/Å	1.34139
Crystal system	Monoclinic
Space group	*C*2/*c*
a/Å	42.559 (2)
b/Å	18.2765(9)
c/Å	30.2728(15)
α/°	90
β/°	128.850(2)
γ/°	90
Volume/Å^3	18338.2(16)
Z	8
Density (calculated) g/cm^3	1.298
μ/mm^{-1}	1.571
F(000)	7504.0
Crystal size/mm^3	0.1 × 0.05 × 0.02
Radiation	GaKα (λ = 1.34139)
2θ range for data collection/°	6.83 to 104.436
Index ranges	$-50 \leq h \leq 49$, $-21 \leq k \leq 21$, $-34 \leq l \leq 35$
Reflections collected	86199
Independent reflections	15474 [R_{int} = 0.1213, R_{sigma} = 0.0901]
Data/restraints/parameters	15474/968/1189
Goodness-of-fit on F^2	1.023
Final *R* indexes [$I >= 2\sigma(I)$]	R_1 = 0.0849, wR_2 = 0.2207
Final *R* indexes [all data]	R_1 = 0.1544, wR_2 = 0.2691
Largest diff. peak/hole / e Å^{-3}	0.83/-0.66

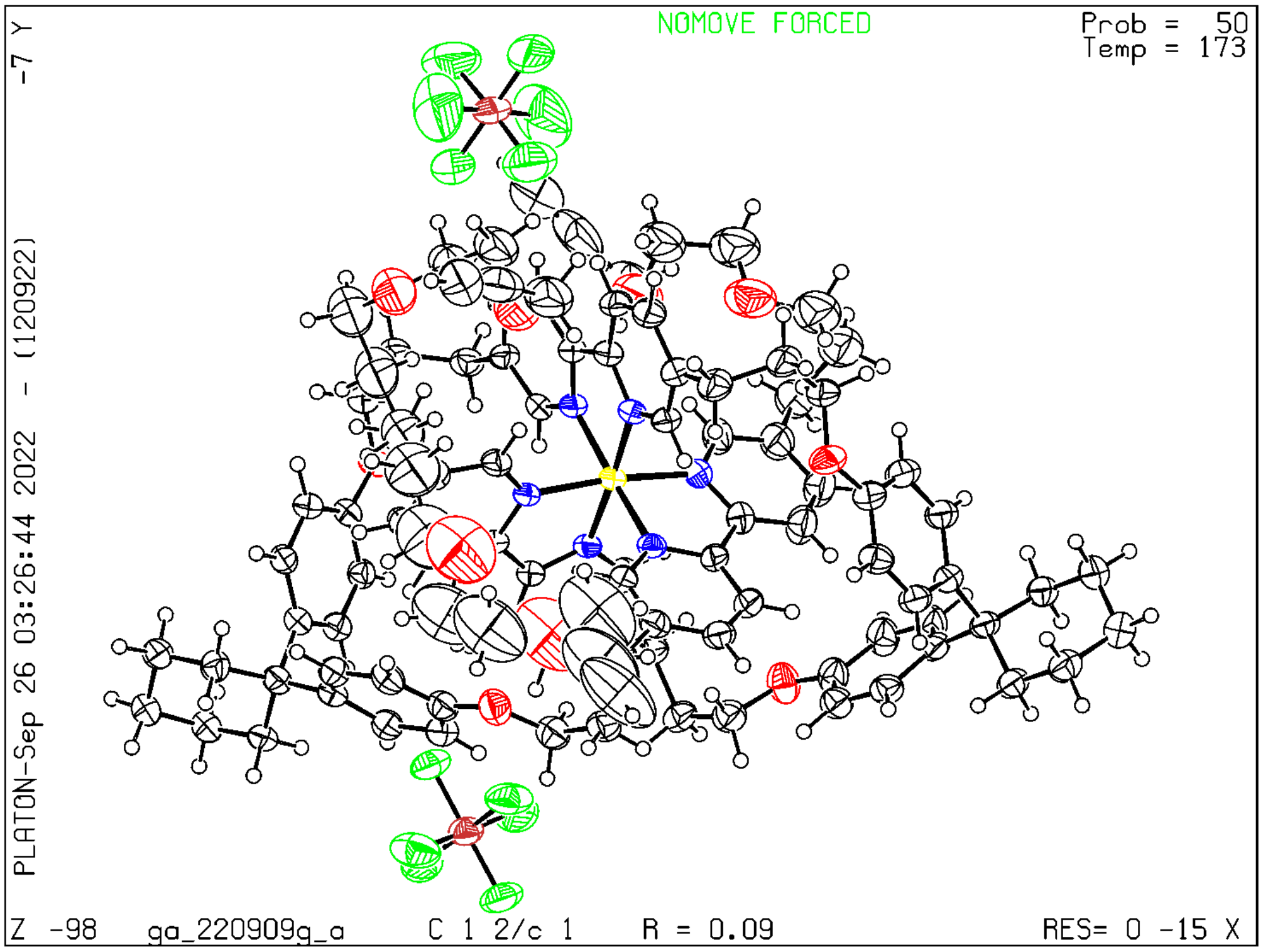

Figure 15. X-ray crystal structure of [Fe**L1**]$(PF_6)_2$ shown with 50% probability ellipsoids.

5.3 Synthesis of S17 and S18

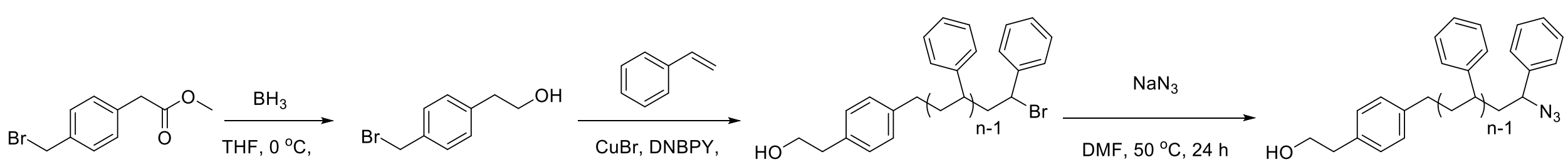

S13 **S14** **S15** **S16** **S17** **S18**

Figure 16. Synthesis of **S17** and **S18**.

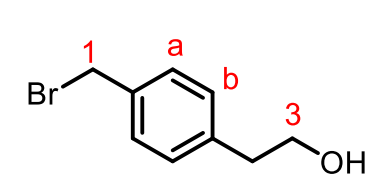

S14

S13 (2.00 g, 8.26 mmol) was dissolved in anhydrous THF (30 mL) in a 100 mL two-necked round bottom flash and stirred at 0 °C. The solution of BH_3 in THF (10 mL, 10 mmol, 1 mol/L in THF) was added slowly to the reaction mixture at 0 °C and then the mixture was stirred for another 4 h at 0 °C. After completion, the reaction was quenched with water (10 mL) and extracted with EA. The organic phase was dry with anhydrous sodium sulfate and evaporated to dryness. The crude product was purified by silica gel column using EA and PE (EA: PE = 1: 8) as eluent to give **S14** as white solid (1.70 g, yield 98%). mp: 95.1–98.0 °C. ^{1}H NMR (400 MHz, Chloroform-*d*) δ 7.34 (d, J = 8.1 Hz, 2H, H_a), 7.21 (d, J = 8.1 Hz, 2H, H_b), 4.49 (s, 2H, H_1), 3.86 (t, J = 6.5 Hz, 2H, H_3),

2.87 (t, $J = 6.5$ Hz, 2H, H_4). ^{13}C NMR (101 MHz, Chloroform-*d*) δ 139.00, 135.99, 129.49, 129.28, 63.49, 38.88, 33.44. LR ESI-MS: $m/z = 581.32$ $[M]^+$ requires 581.18.

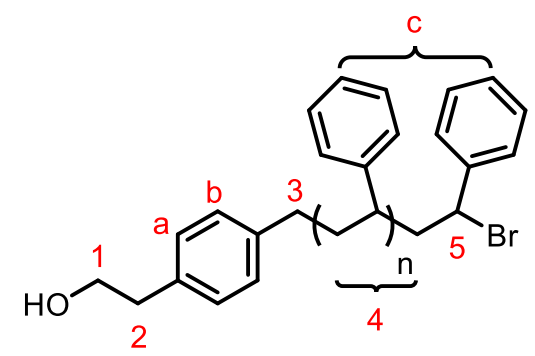

S15

S14 (0.22 g ,1.03 mmol), styrene (16.04 g, 154.21 mmol) and 4,4'-dinonyl-2,2-bipyridine (0.84 g, 2.06 mmol) were added to a two-necked round bottom flash in N_2 atmosphere. The mixture was degassed by three freeze-pump-thaw cycles and backfilled with nitrogen. The mixture was added CuBr (145.83 mg, 1.03 mmol) in N_2 atmosphere and degassed again by freeze-pump-thaw cycle. The reaction mixture was stirred at 100 °C for 4 hours. After completion, the reaction mixture was wlooped to room temperature and diluted with THF. The copper complex was removed by neutral alumina column. The product was precipitated from MeOH for three times and dry at 60 °C for 6 hours to give **S15** as white solid (1.81 g, $M_n = 4412$ Da, $Đ = 1.10$).

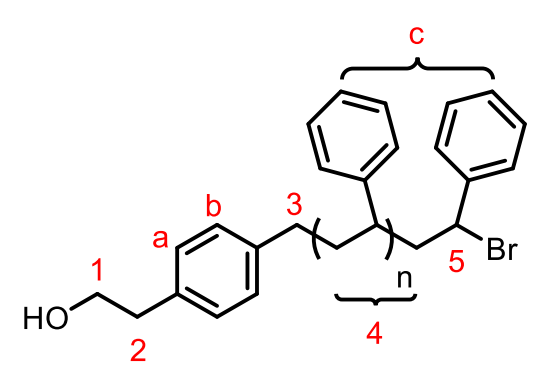

S16

S14 (0.10 g ,0.47 mmol), styrene (7.29 g, 70.09 mmol) and 4,4'-dinonyl-2,2-bipyridine (0.38 g, 0.94 mmol) were added to a two-necked round bottom flash in N_2 atmosphere. The mixture was degassed by three freeze-pump-thaw cycles and backfilled with nitrogen. The mixture was added CuBr (145.8 mg, 1.03 mmol) in N_2 atmosphere and degassed again by freeze-pump-thaw cycle. The reaction mixture was stirred at 100 °C for 1 hour and 40 mins. After completion, the reaction mixture was wlooped to room temperature and diluted with THF. The copper complex was removed by neutral alumina column. The product was precipitated from MeOH for three times and dry at 60 °C for 6 hours to give **S16** as white solid (2.45 g, $M_n = 2862$ Da, $Đ = 1.15$).

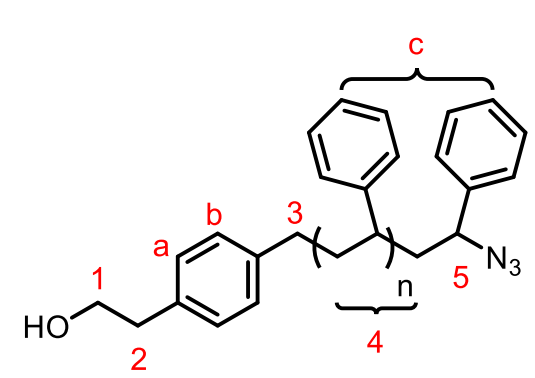

S17

To a stirred solution of **S15** (1.70 g) in DMF was added NaN_3 (0.25 g). The reaction mixture was stirred at 50 °C for 24 h. After completion, the reaction mixture was wlooped to room temperature and diluted with water. White solid precipitated from the mixture, filtration and wash the filter cake with water and MeOH. The solid was redissolved in DCM and concentrated under vacuum.

The product was precipitated from MeOH and dry at 60 °C for 6 hours to give **S17** as white solid (1.68 g, yield 99%, M_n = 4401 Da, *Đ* = 1.10).

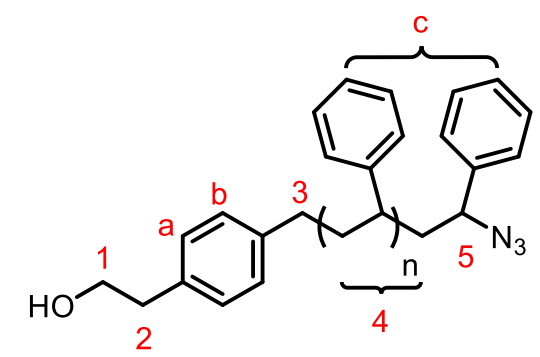

S18

To a stirred solution of **S16** (2.40 g) in DMF was added NaN_3 (0.50 g). The reaction mixture was stirred at 50 °C for 24 h. After completion, the reaction mixture was wlooped to room temperature and diluted with water. White solid precipitated from the mixture, filtration and wash the filter cake with water and MeOH. The solid was redissolved in DCM and concentrated under vacuum. The product was precipitated from MeOH and dry at 60 °C for 6 hours to give **S18** as white solid (2.30 g, yield 96%, M_n = 2886 Da, *Đ* = 1.14).

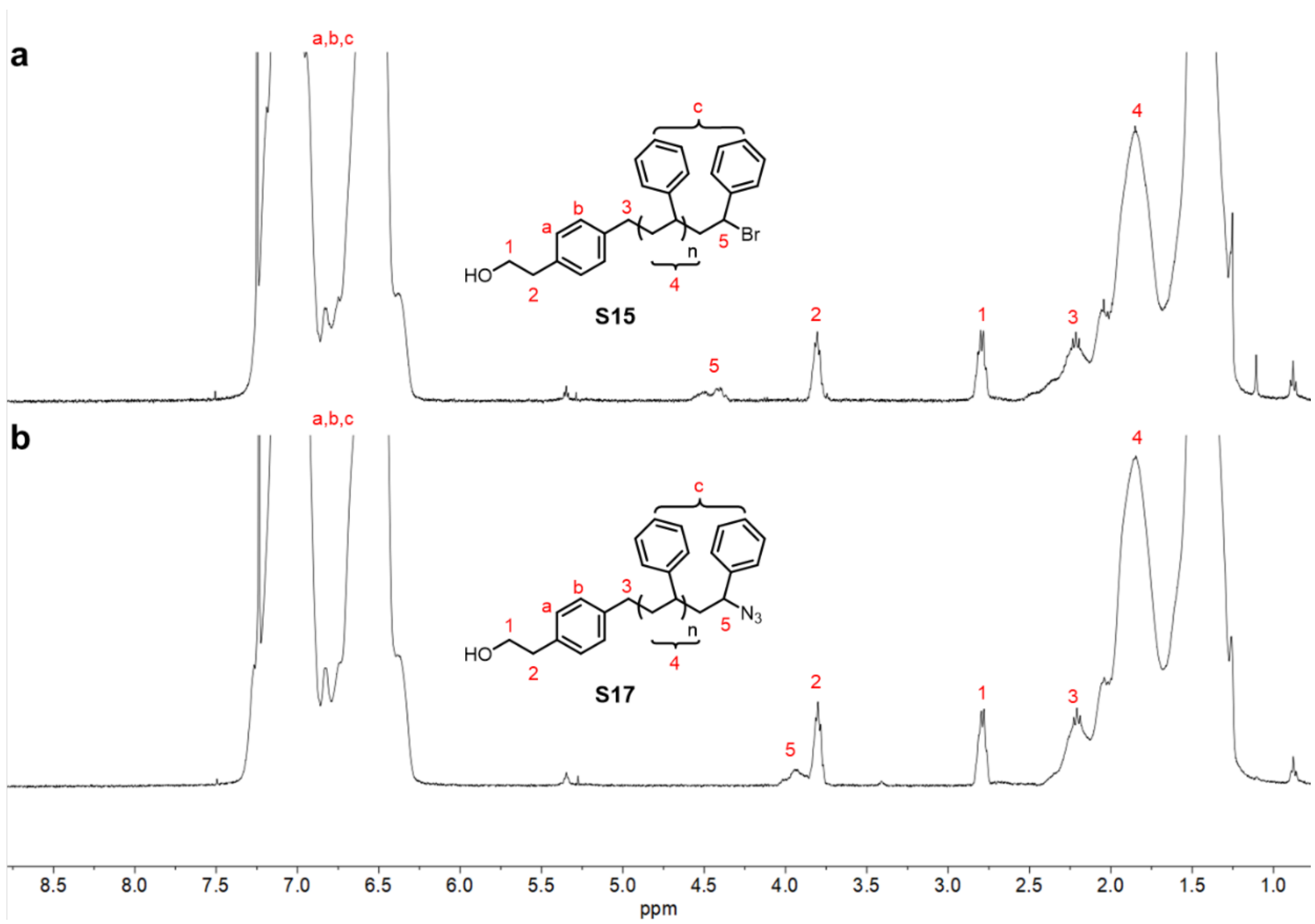

Figure 17. ^1H NMR spectra (400 MHz, $CDCl_3$, 298 K) of (a) **S15** and (b) **S17**. The obvious and thorough down-field shift of H_5 signal indicate the successful replacement of $-Br$ by $-N_3$.

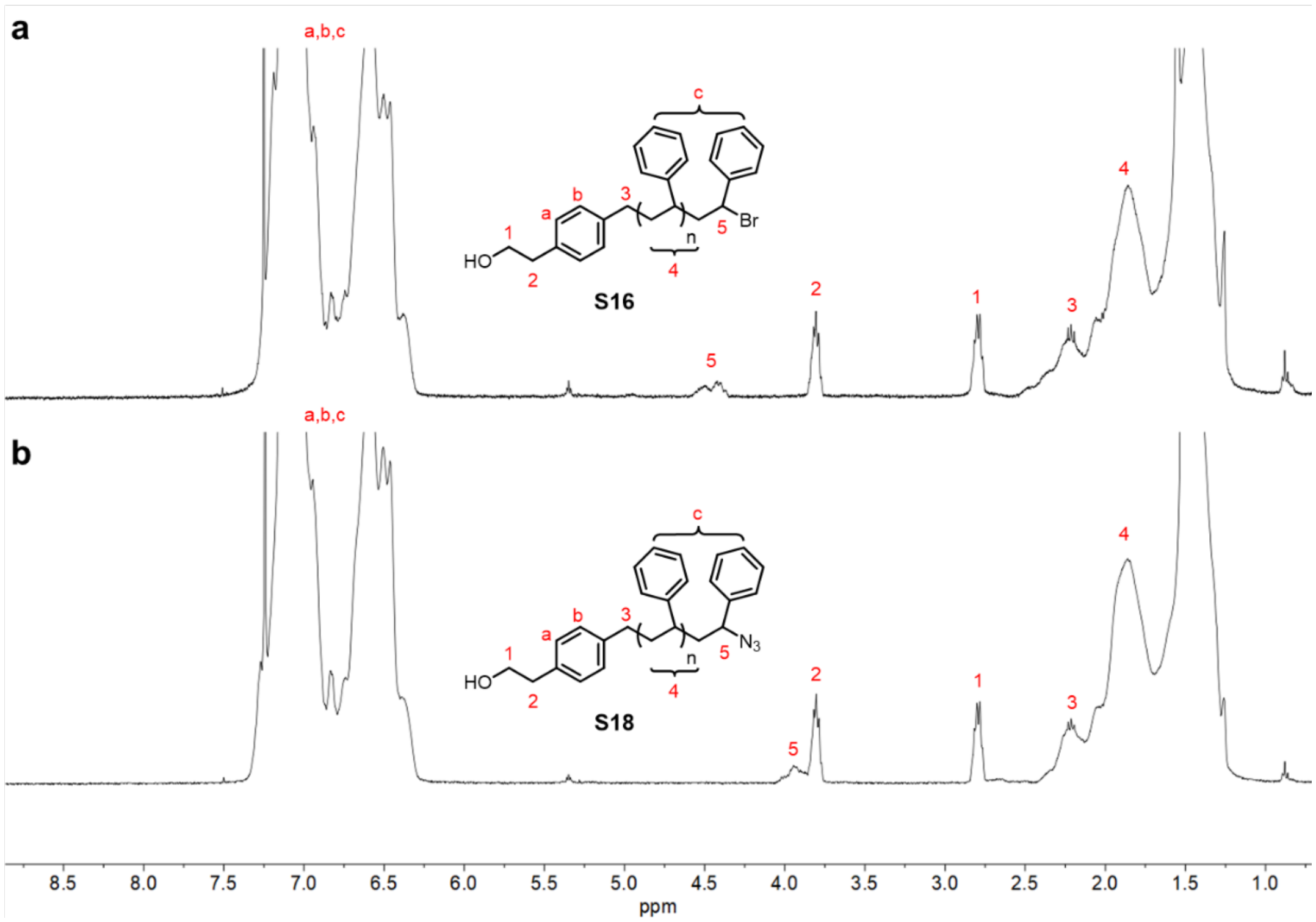

Figure 18. ^{1}H NMR spectra (400 MHz, $CDCl_3$, 298 K) of (a) **S16** and (b) **S18**. The obvious and thorough down-field shift of H_5 signal indicate the successful replacement of -Br by $-N_3$.

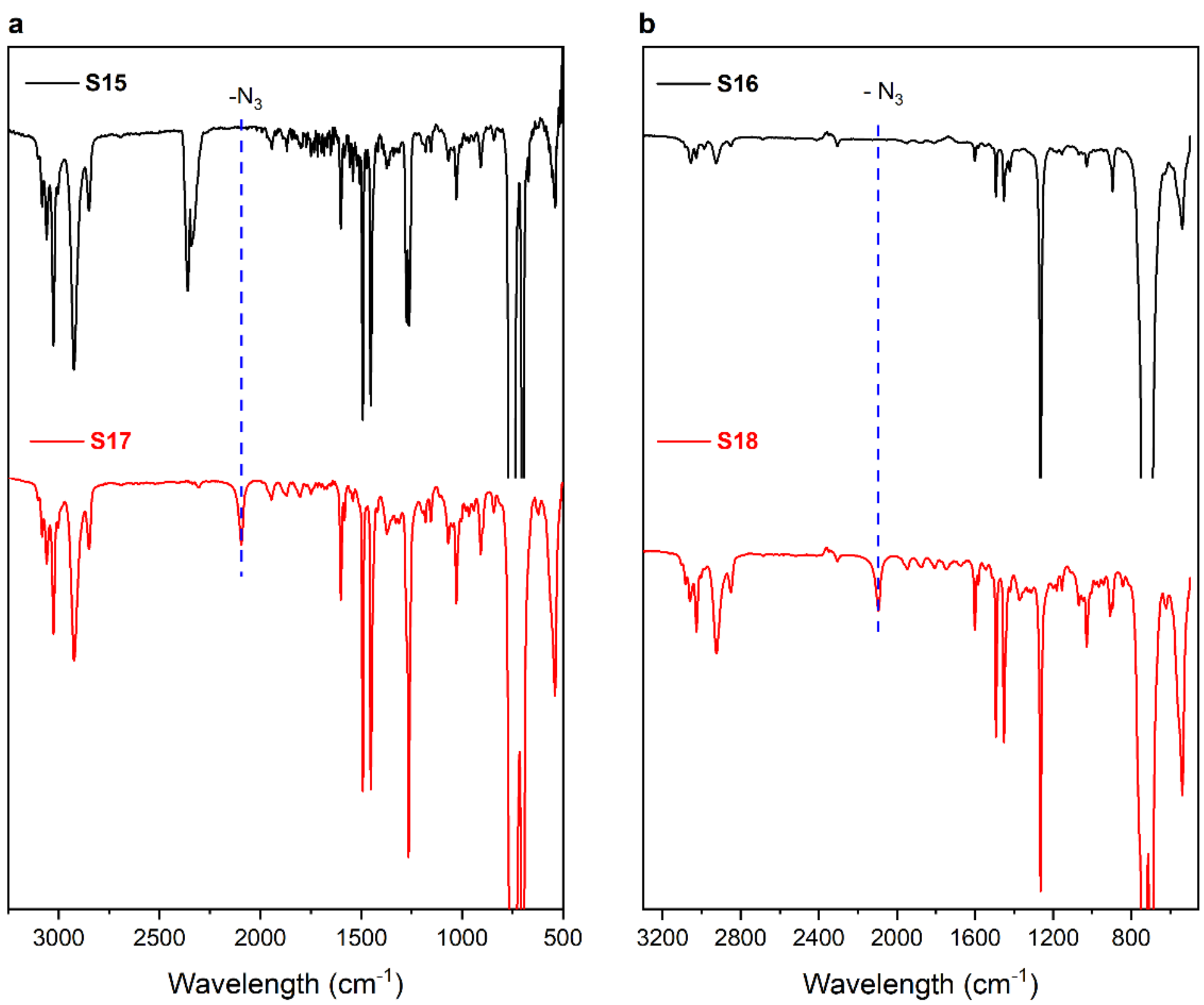

Figure 19. IR spectra of (a) **S15**, **S17** and (b) **S16**, **S18**. The characteristic peak of $-N_3$ at 2095 cm^{-1} prove the successful introduce of $-N_3$.

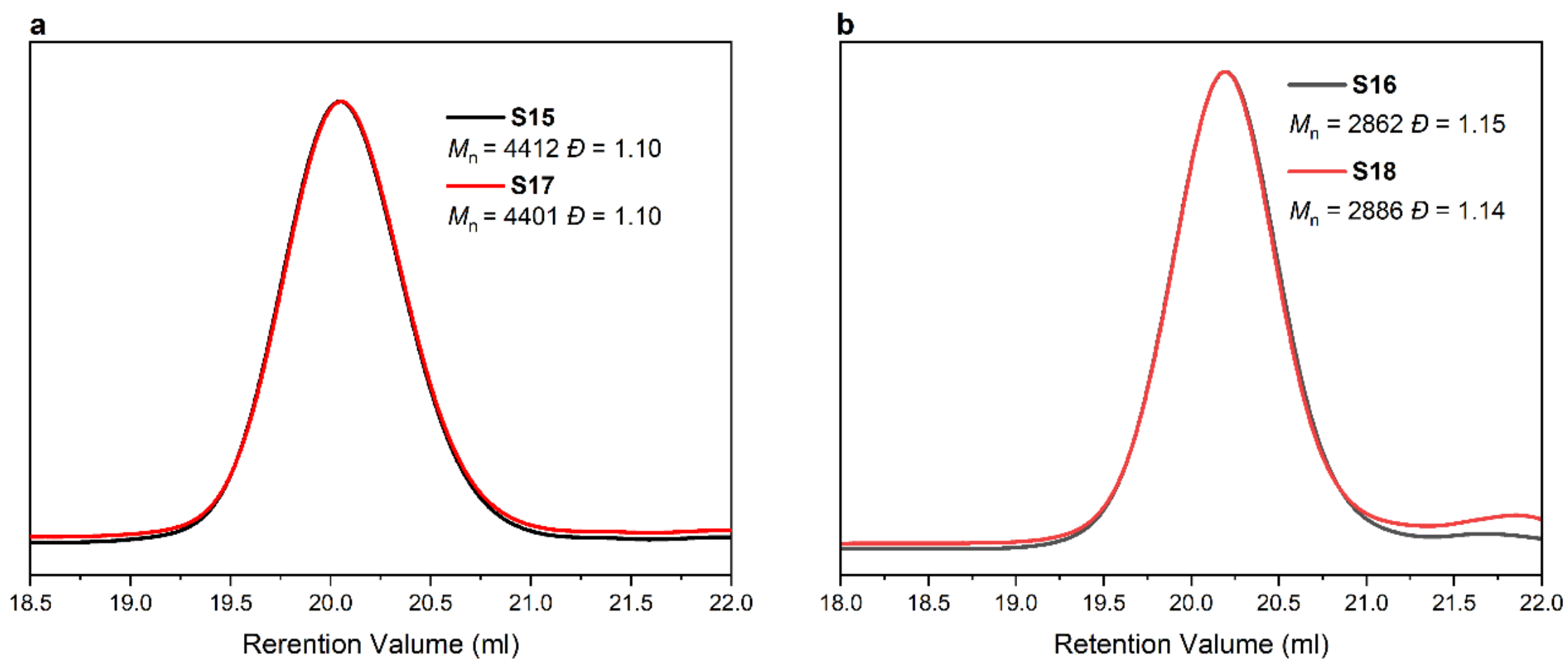

Figure 20. GPC curves of (a) **S15**, **S17** and (b) **S16**, **S18**.

5.4 Synthesis of S23

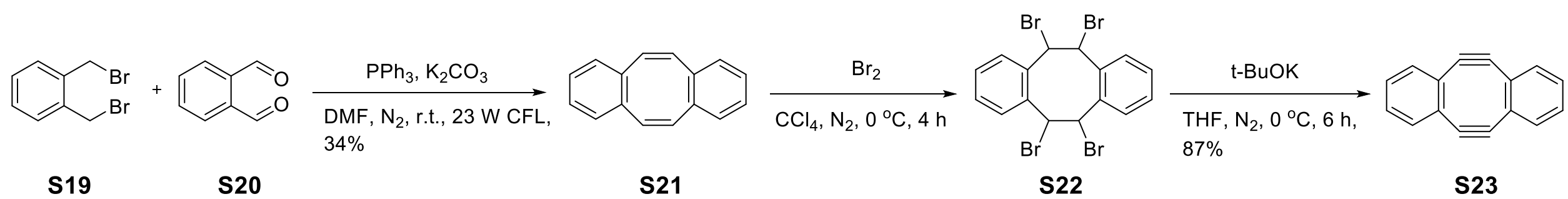

Figure 21. Synthesis of compound **S23**.

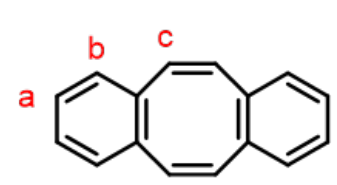

S21

S19 (5.00 g, 19.09 mmol), **S20** (3.07 g, 22.91 mmol), PPh_3 (12.02 g, 45.81 mmol), K_2CO_3 (6.23 g, 45.05 mmol) and $Ru(bpy)_3 \cdot 6H_2O$ (71.45 mg, 0.0955 mmol) were added into an oven-dried round bottom flask with dry DMSO in N_2 atmosphere. Catalyzing the reaction with white light at room temperature and stirring overnight. After completion, extract the mixture with water and EA, the organic phase dry with anhydrous Na_2SO_4 and evaporated to dryness. The crude product was purified by silica gel column with PE as eluent to give **S21** as pale-yellow solid (0.64 g, yield 34%). Mp: 104.8–107.6 °C. ^{1}H NMR (400 MHz, Chloroform-*d*) δ 7.16 (dd, J = 5.8, 3.3 Hz, 4H, H_b), 7.06 (dd, J = 5.8, 3.4 Hz, 4H, H_a), 6.76 (s, 4H, H_c). ^{13}C NMR (101 MHz, Chloroform-*d*) δ 137.08, 133.24, 129.11, 126.83. LR ESI-MS: *m/z* = 204.24 $[M]^+$ requires 204.09.

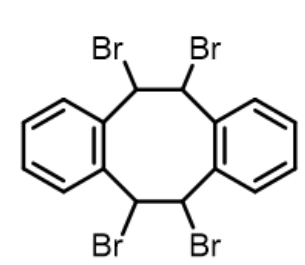

S22

S21 (1.10 g, 5.39 mmol) was dissolved in CCl_4 in N_2 atmosphere, Br_2 (1.89 g in CCl_4, 11.31 mmol) was added dropwise to the mixture at 0 °C under stirring. Then the reaction mixture was stirred at 0 °C for 4 hours. After completion, quenching the reaction with aqueous solution of sodium sulfite and extracting the mixture with water and EA, drying the organic phase with anhydrous Na_2SO_4. The organic phase was evaporated to dryness to give crude **S22** as brown solid. The crude product

was used directly without further purification.

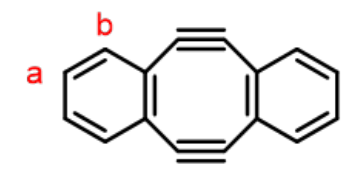

S23

Potassium tert-butoxide (54 mL, 54 mmol, 1 mol·L^{-1} in THF) was stirred in an oven-dried two-necked round bottom flask. The solution of **S22** (2.82 g, 5.38 mmol) in 25 mL dry THF was injected into the mixture at 0 °C and the reaction mixture was stirred for 6 hours at 0 °C. After completion, extraction of the mixture with water and EA, the organic phase dry with anhydrous Na_2SO_4 and evaporated to dryness. The crude product was purified by silica gel column with PE as eluent to give **S23** as yellow solid. (0.94 g, yield 87%). ^{1}H NMR (400 MHz, Chloroform-*d*) δ 6.93 (dd, J = 5.6, 3.3 Hz, 4H, H_b), 6.74 (dd, J = 5.6, 3.3 Hz, 4H, H_a). ^{13}C NMR (101 MHz, Chloroform-*d*) δ 132.91, 129.06, 126.91, 109.33. The chemical shifts are consistent with that reported in the literature.[2]

5.5 Synthesis of [Fe2]$(PF_6)_2$ and [Fe5]$(PF_6)_2$

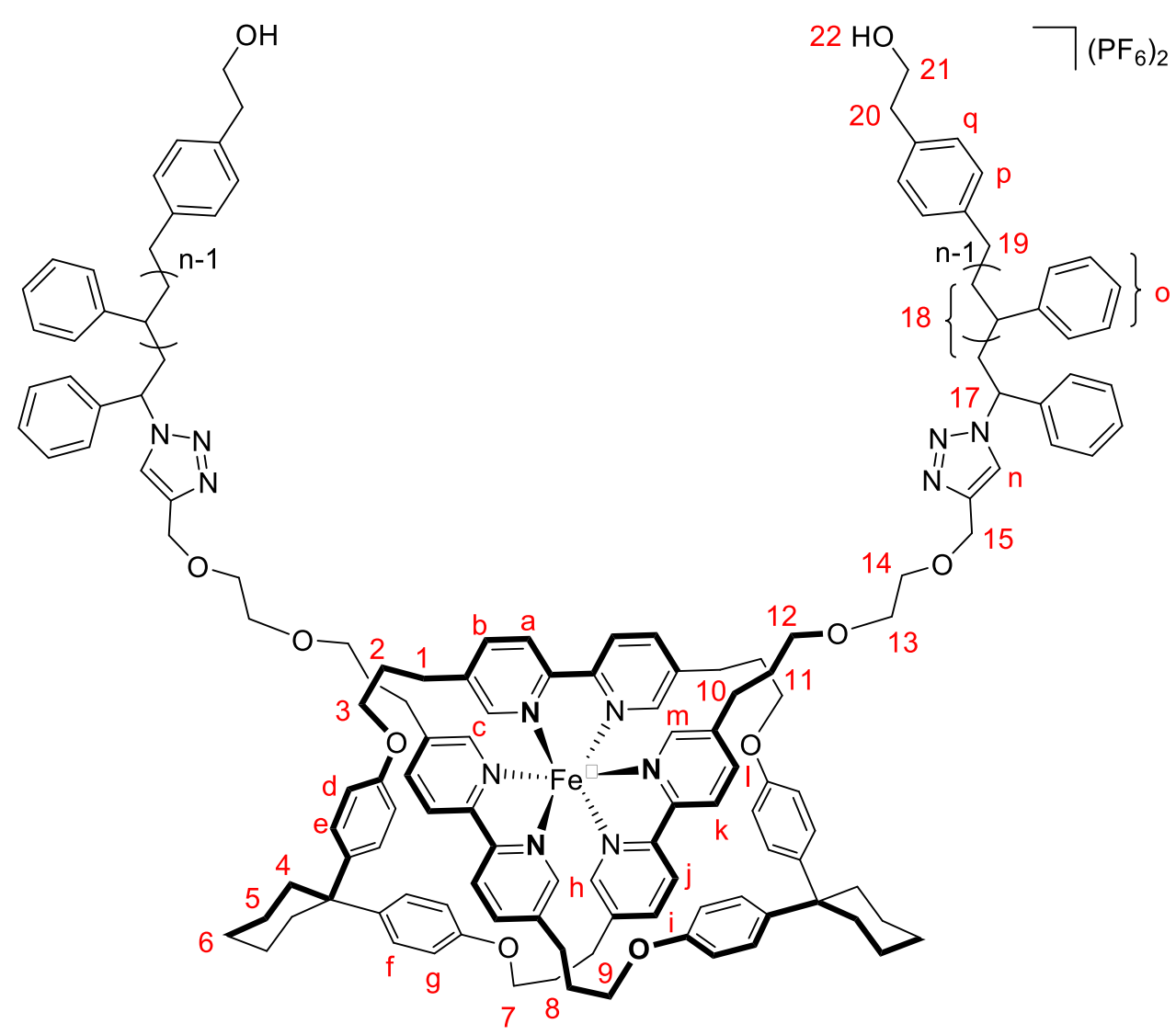

[Fe**L2**]$(PF_6)_2$

[Fe**L1**]$(PF_6)_2$ (0.20 g, 111.7 µmol), **S17** (1.47 g, 335.1 µmol), $Cu(MeCN)_4·PF_6$ (0.125 g, 335.1 µmol) and TBTA (0.18 g, 335.1 µmol) were dissolved in the mixture solvent of MeCN (15 mL), MeOH (15 mL), THF (30 mL) in a sealed vial in N_2 atmosphere. The reaction mixture was stirred at 50 °C for 48 hours. After completion, the crude product and excess linear polymer were precipitated from MeOH. Filtration and wash the filter cake with MeOH. The filter cake was redissolved in DCM and the solvent was evaporated under vacuum. The crude product was purified by silica gel column with DCM and MeOH (DCM: MeOH = 50: 1) as eluent to give [Fe**L2**]$(PF_6)_2$ as red solid (1.10 g, yield 93%).

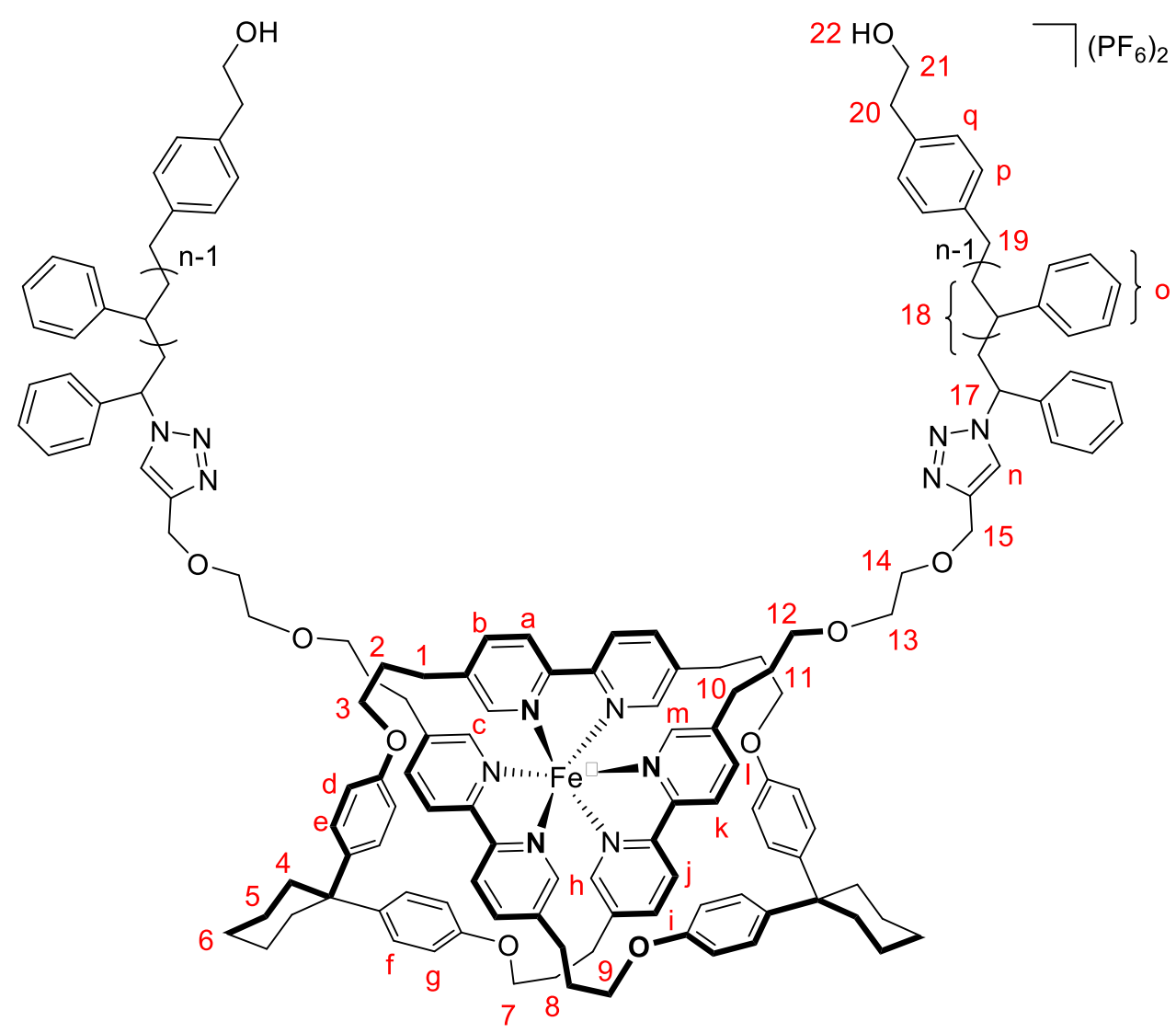

[FeL3](PF_6)$_2$

[Fe**L1**](PF_6)$_2$ (0.194 g, 108.34 μmol), **S18** (0.94 g, 325.02 μmol), Cu(MeCN)$_4$·PF_6 (0.12 g, 325.02 μmol) and TBTA (0.17 g, 325.02 μmol) were dissolved in the mixture solvent of MeCN (15 mL), MeOH (15 mL), THF (30 mL) in a sealed vial in N_2 atmosphere. The reaction mixture was stirred at 50 °C for 48 hours. After completion, the crude product and excess linear polymer were precipitated from MeOH. Filtration and wash the filter cake with MeOH. The filter cake was redissolved in DCM and the solvent was evaporated under vacuum. The crude product was purified by silica gel column with DCM and MeOH (DCM: MeOH = 50 :1) as eluent to give [Fe**L3**](PF_6)$_2$ as red solid (0.80 g, yield 97%).

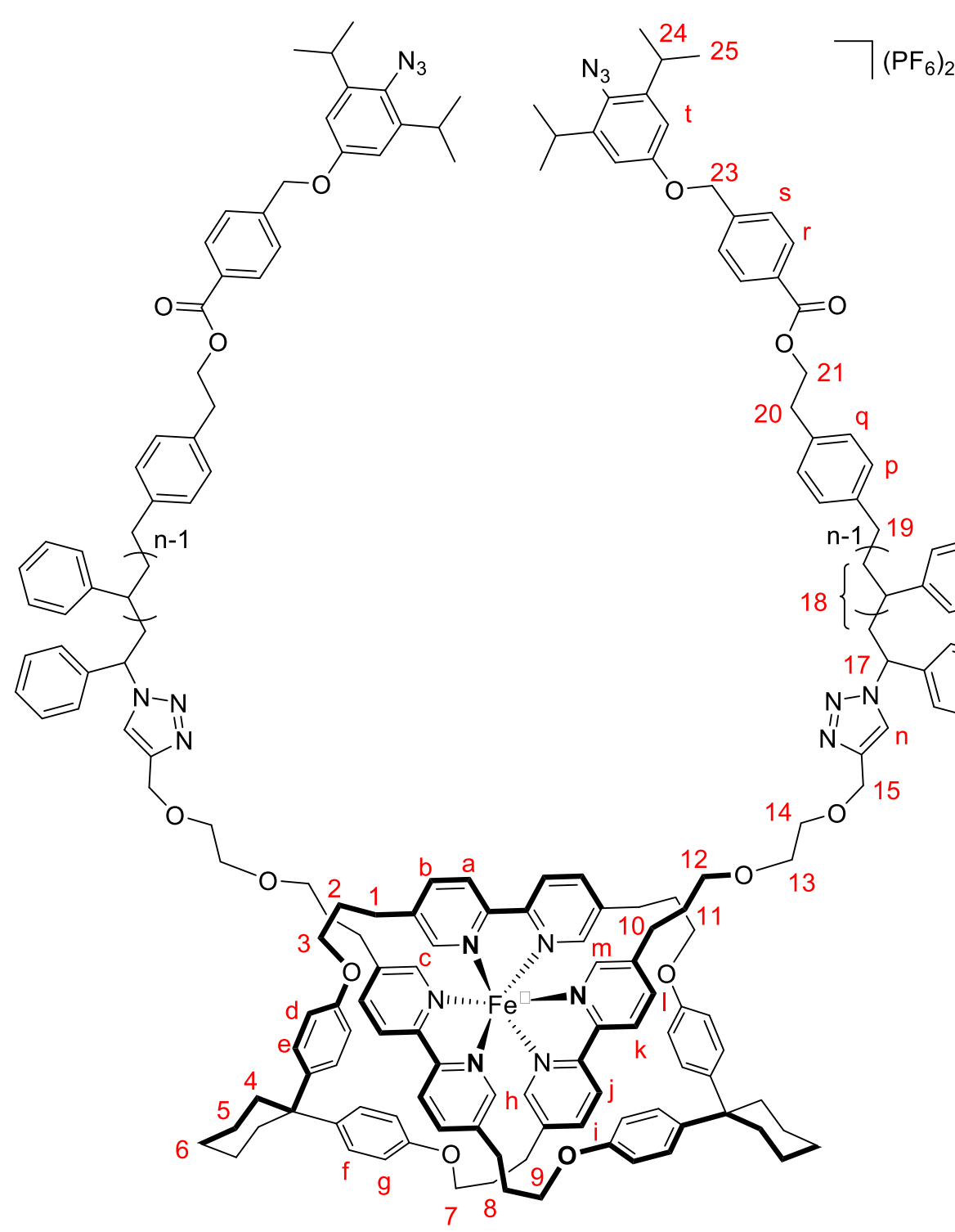

[Fe1](PF_6)$_2$

[Fe**L2**]$(PF_6)_2$ (1.05 g, 99.1 μmol), **S24** (0.41 g, 594.7 μmol), and DMAP (36.3 mg, 297.3 μmol) were dissolved in anhydrous DCM (30 mL) in a 100 mL round bottom flask in N_2 atmosphere. The reaction mixture was stirred at room temperature for 4 days. After completion, the mixture was concentrated under vacuum and the crude product was precipitated from MeOH. Filtration and wash the filter cake with MeOH and redissolved the filter cake in DCM. This process was repeated three times to give [Fe**1**]$(PF_6)_2$ as pink solid (1.06 g, yield 95%).

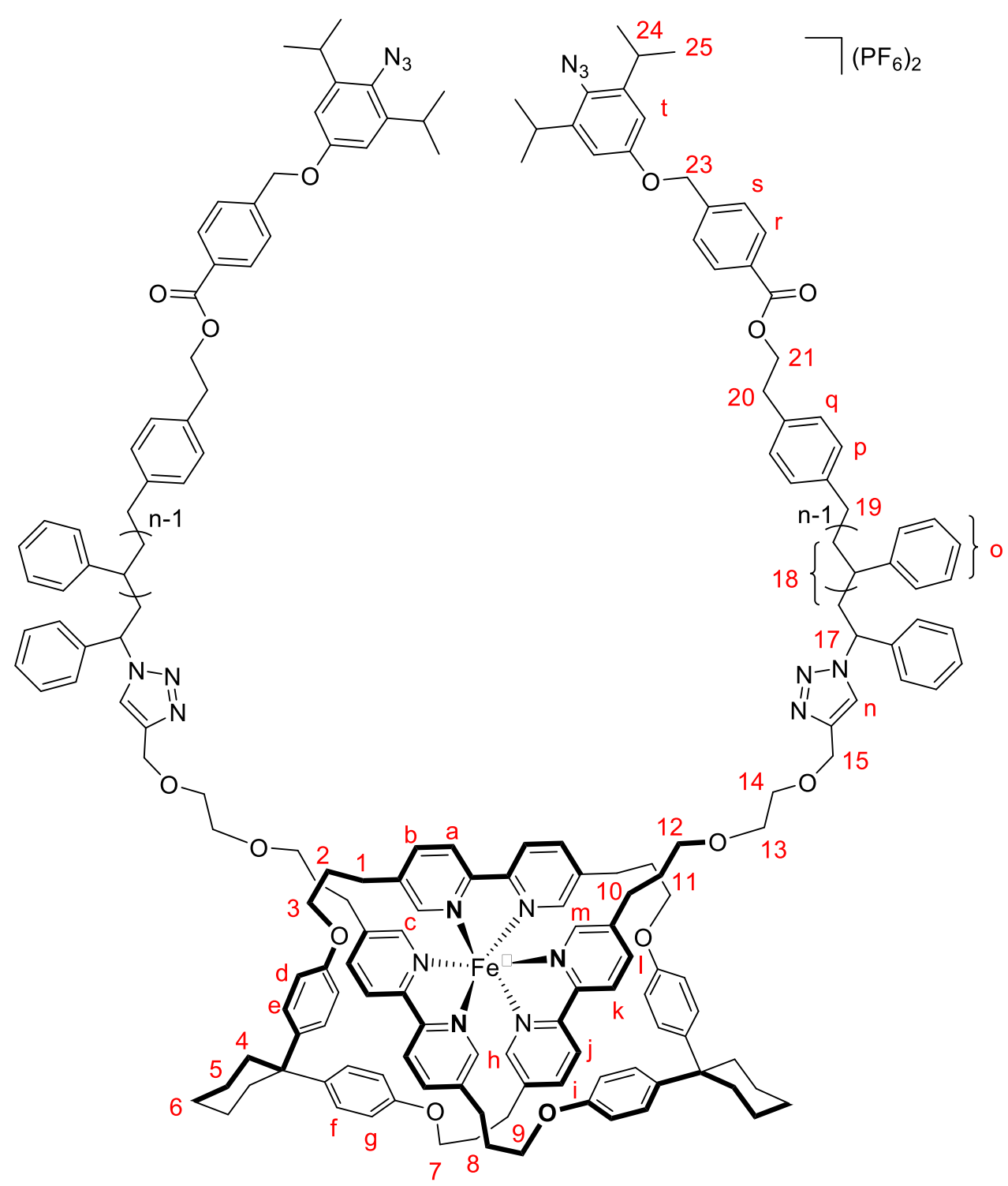

[Fe**4**]$(PF_6)_2$

[Fe**L3**]$(PF_6)_2$ (0.75 g, 100.5 μmol), **S24** (0.42 g, 602.9 μmol), and DMAP (37.5 mg, 301.5 μmol) were dissolved in anhydrous DCM (30 mL) in a 100 mL round bottom flask in N_2 atmosphere. The reaction mixture was stirred at room temperature for 4 days. After completion, the mixture was concentrated under vacuum and the crude product was precipitated from MeOH. Filtration and wash the filter cake with MeOH and redissolved the filter cake in DCM. This process was repeated three times to give [Fe**4**]$(PF_6)_2$ as pink solid (0.69 g, yield 85%).

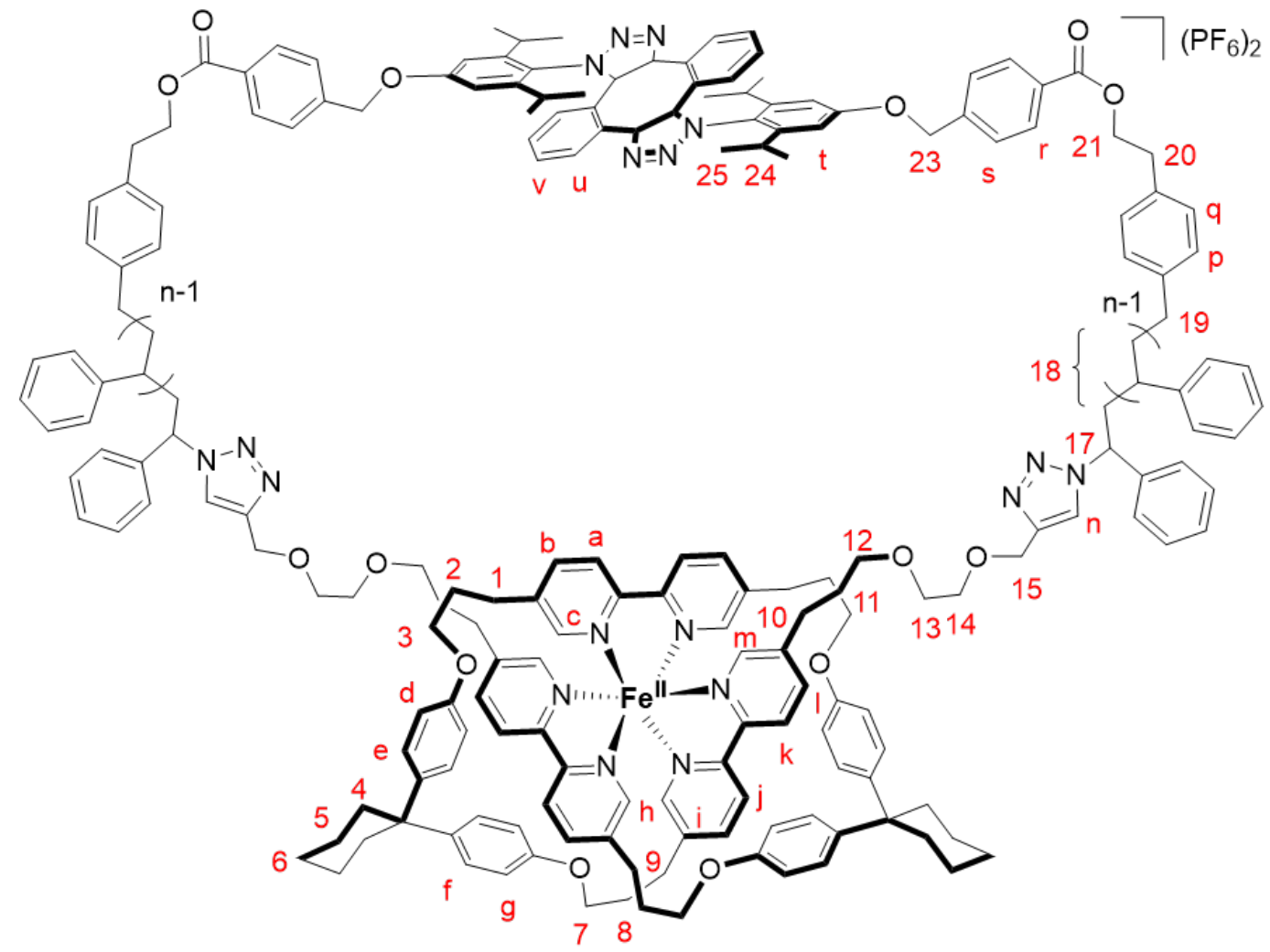

[Fe**2**](PF_6)$_2$

To a stirred solution of **S23** (88.8 mg, 443.9 µmol) in THF (125 mL) was added the solution of [Fe**1**](PF_6)$_2$ (0.25 g, 22.2 µmol) in THF (42 mL) dropwise for 24 hours and then the reaction mixture was stirred for another 5 hours. After completion, the solvent was evaporated to dryness. The crude product was dissolved in DCM and precipitated from MeOH. Filtration and wash the filter cake with MeOH and redissolved the filter cake in DCM. This process was repeated three times to give [Fe**2**](PF_6)$_2$ as pink solid (250 mg, crude yield: 98%, could contain some higher molecular weight by-product which formation by the oligomerization of [Fe**1**](PF_6)$_2$ and **S23**).

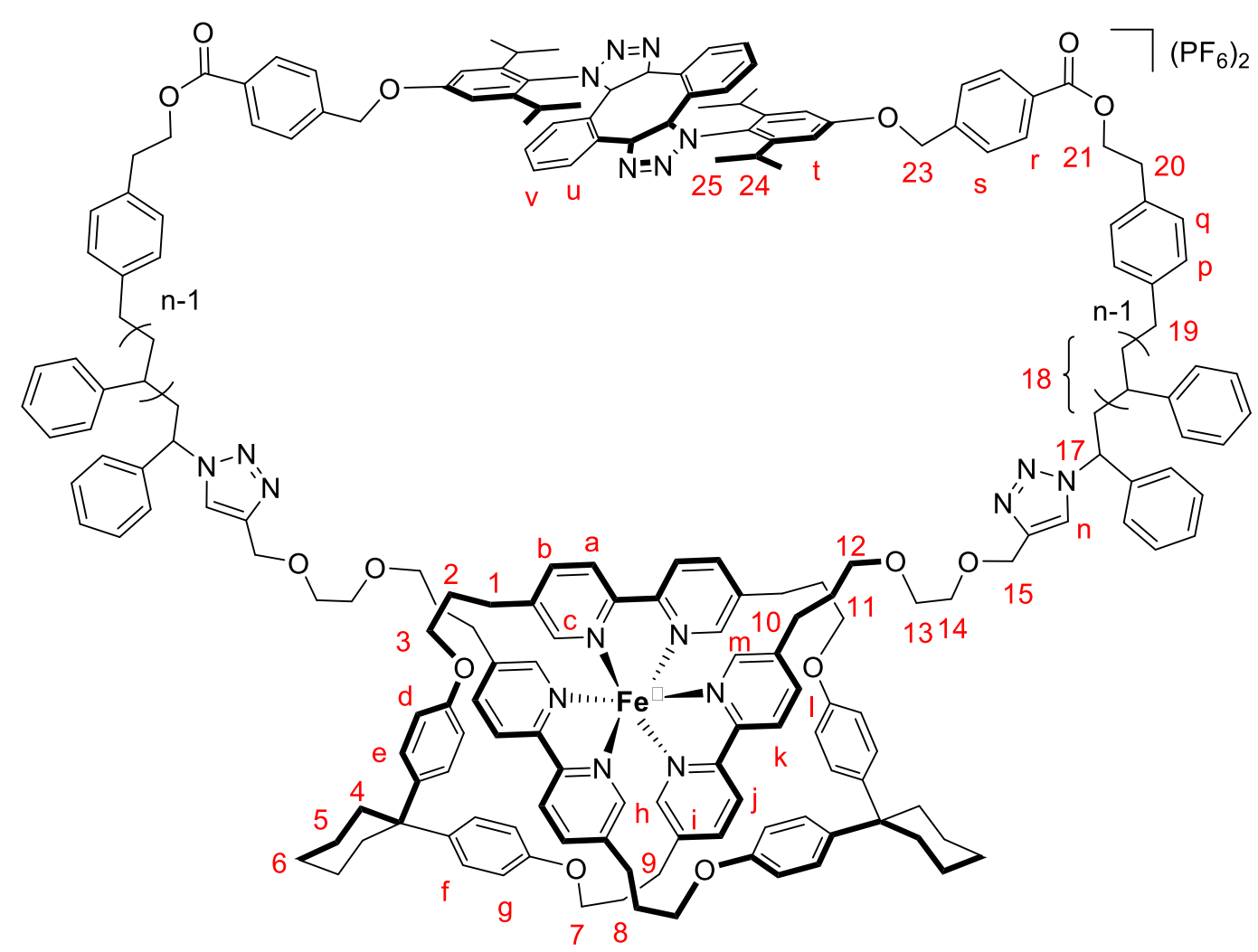

[Fe**5**](PF_6)$_2$

To a stirred solution of **S23** (121.5 mg, 607.2 µmol) in THF (190 mL) was added the solution of [Fe**4**](PF_6)$_2$ (0.25 g, 30.4 µmol) in THF (50 mL) dropwise for 24 hours and then the reaction mixture was stirred for another 5 hours. After completion, the solvent was evaporated to dryness. The crude product was dissolved in DCM and precipitated from MeOH. Filtration and wash the filter cake with MeOH and redissolved the filter cake in DCM. This process was repeated three times to give [Fe**5**](PF_6)$_2$ as pink solid (0.245 g, crude yield: 96%, contain some higher molecular weight by-products which formation by the oligomerization of [Fe**4**](PF_6)$_2$ and **S23**).

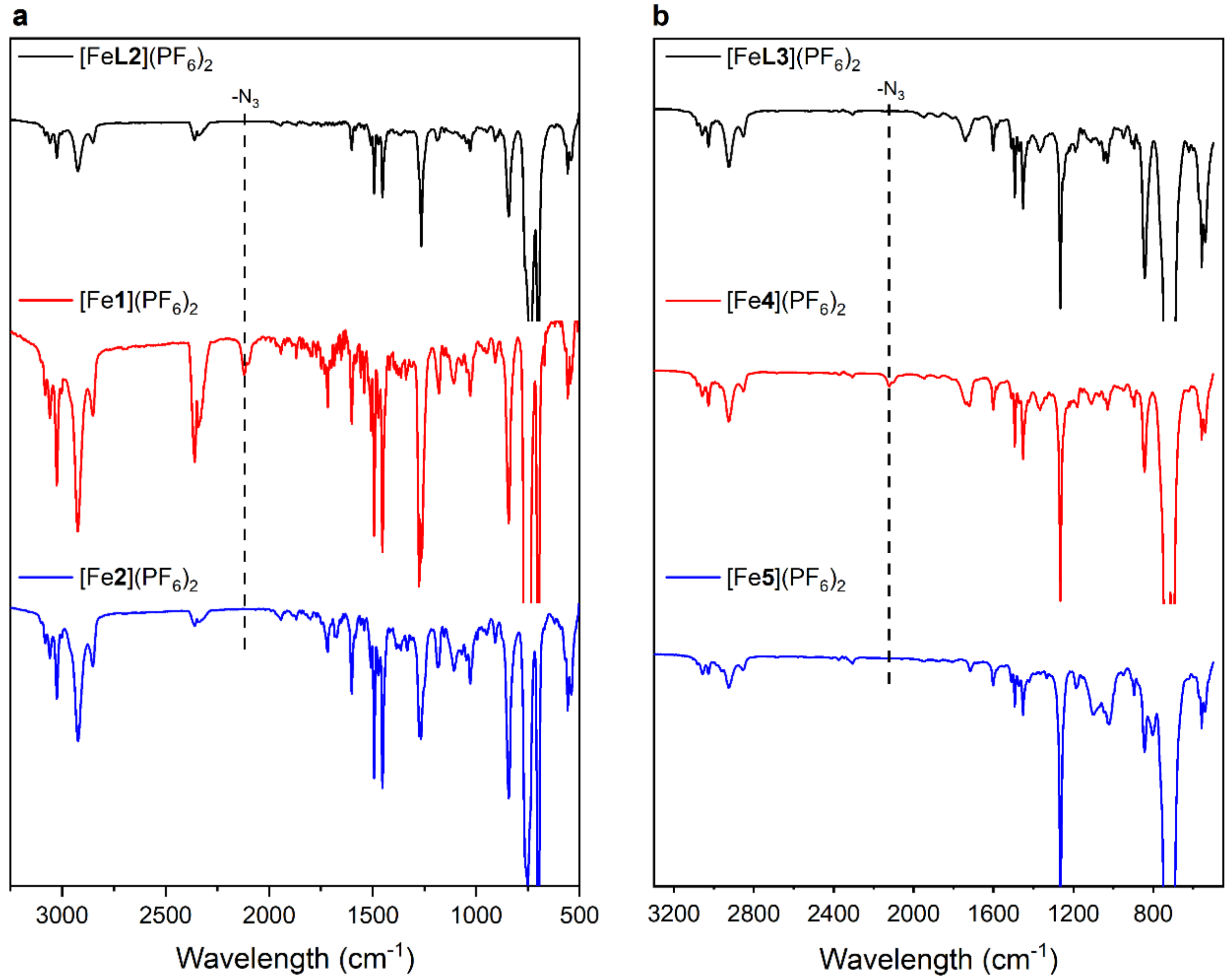

Figure 22. IR spectra of (a) [Fe**L2**]$(PF_6)_2$ [Fe**1**]$(PF_6)_2$, [Fe**2**]$(PF_6)_2$; (b) [Fe**L3**]$(PF_6)_2$, [Fe**4**]$(PF_6)_2$, [Fe**5**]$(PF_6)_2$. The appearance and disappearance of the characteristic peak of $-N_3$ at 2122 cm^{-1} prove the successful introduce of terminal $-N_3$ in [Fe**1**]$(PF_6)_2$, [Fe**4**]$(PF_6)_2$ and thoroughly consumed the $-N_3$ group when prepared [Fe**2**]$(PF_6)_2$ and [Fe**5**]$(PF_6)_2$.

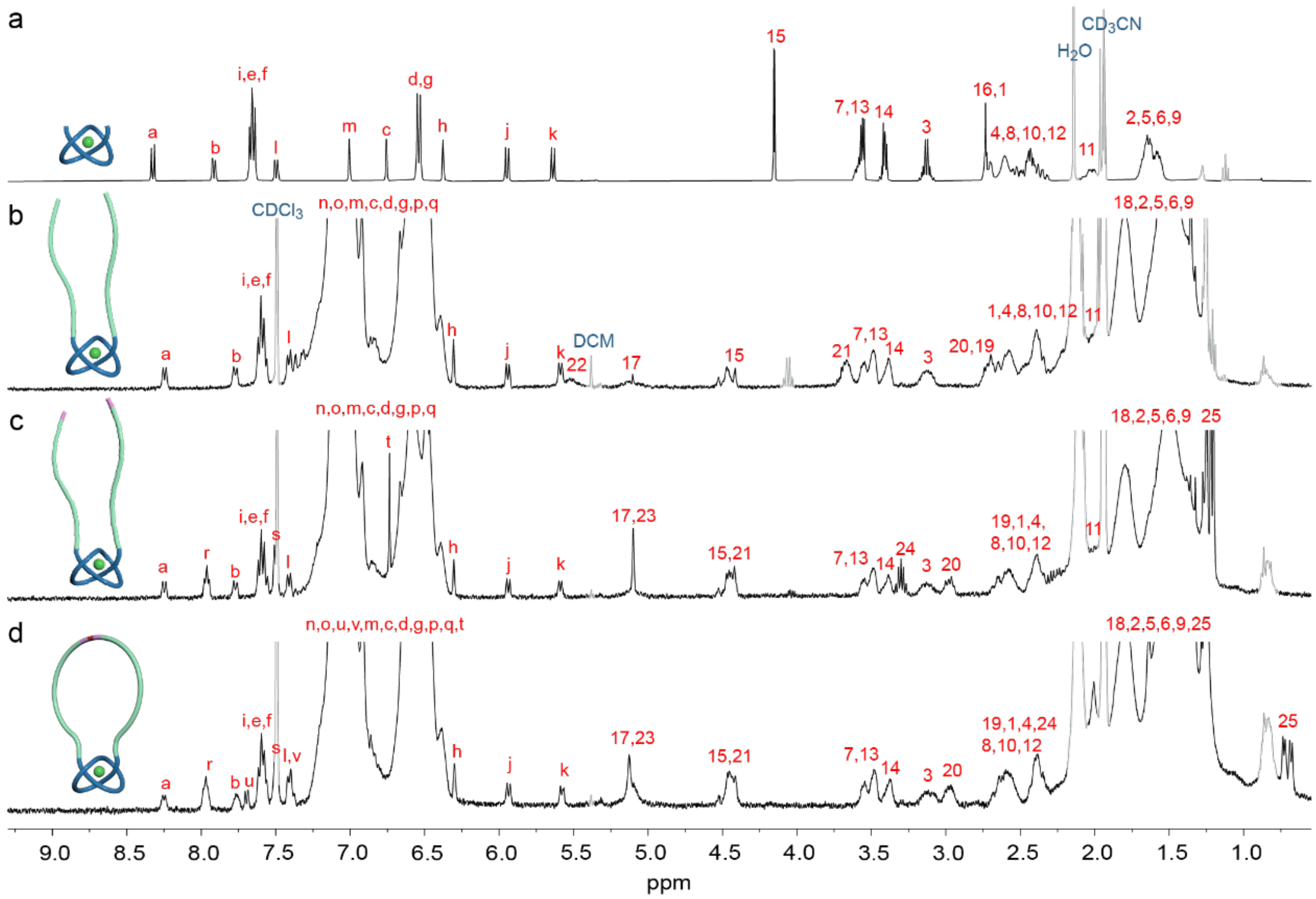

Figure 23. ^{1}H NMR spectra (400 MHz, MeCN-d_3 or MeCN-d_3:$CDCl_3$ = 5 : 1, 298 K) of (a) [Fe**L1**]$(PF_6)_2$; (b) [Fe**L2**]$(PF_6)_2$; (c) [Fe**1**]$(PF_6)_2$; (d) [Fe**2**]$(PF_6)_2$. $CDCl_3$ was added to increase the solubility of the polymers.

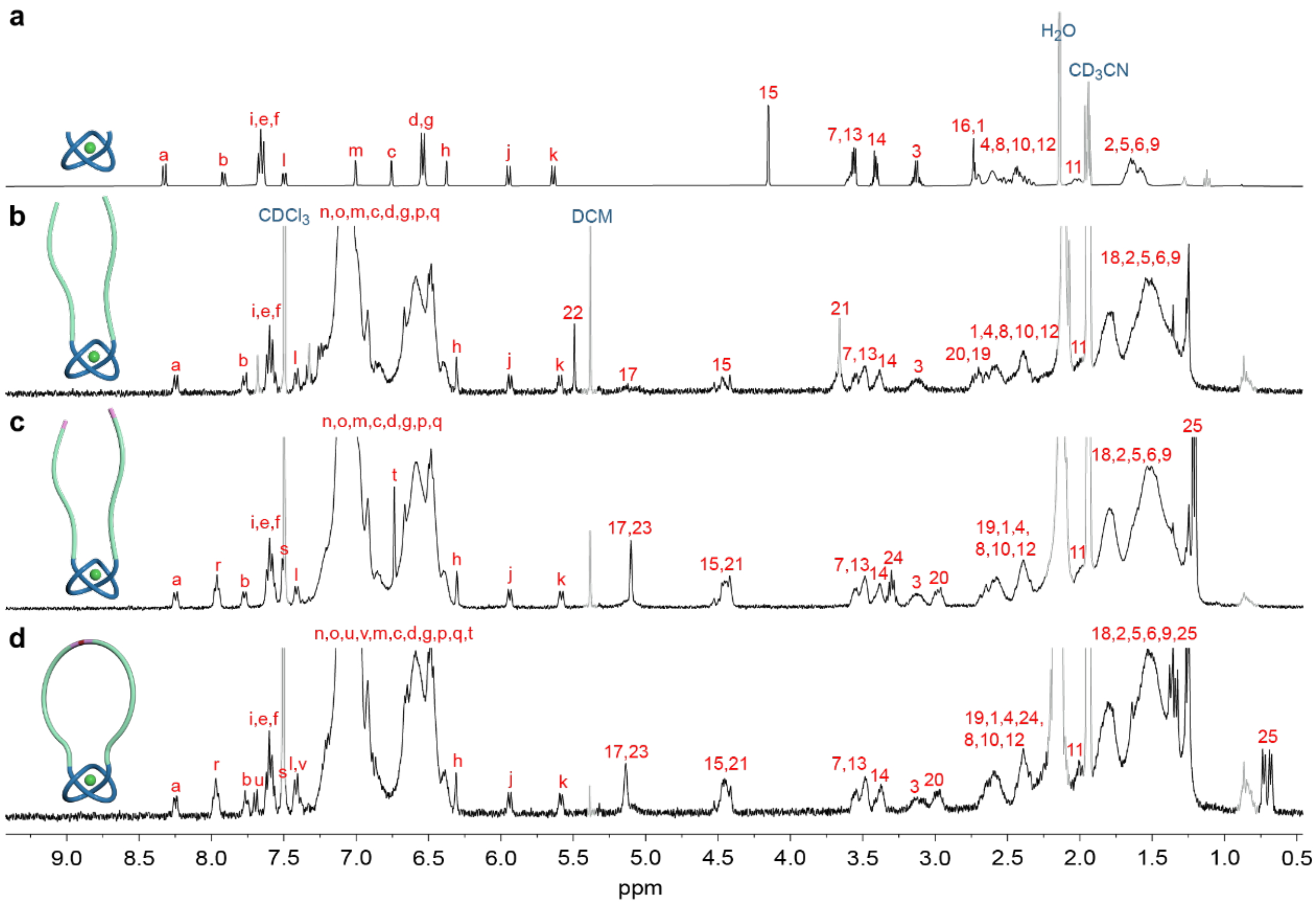

Figure 24. ^{1}H NMR spectra (400 MHz, MeCN-d_3 or MeCN-d_3:$CDCl_3$ = 5 : 1, 298 K) of (a) [Fe**L1**]$(PF_6)_2$; (b) [Fe**L3**]$(PF_6)_2$; (c) [Fe**4**]$(PF_6)_2$; (d) [Fe**5**]$(PF_6)_2$. $CDCl_3$ was added to increase the solubility of the polymers.

5.6 Synthesis of linear (1, 4), trefoiled (2, 5) and cyclic (3, 6) polymers

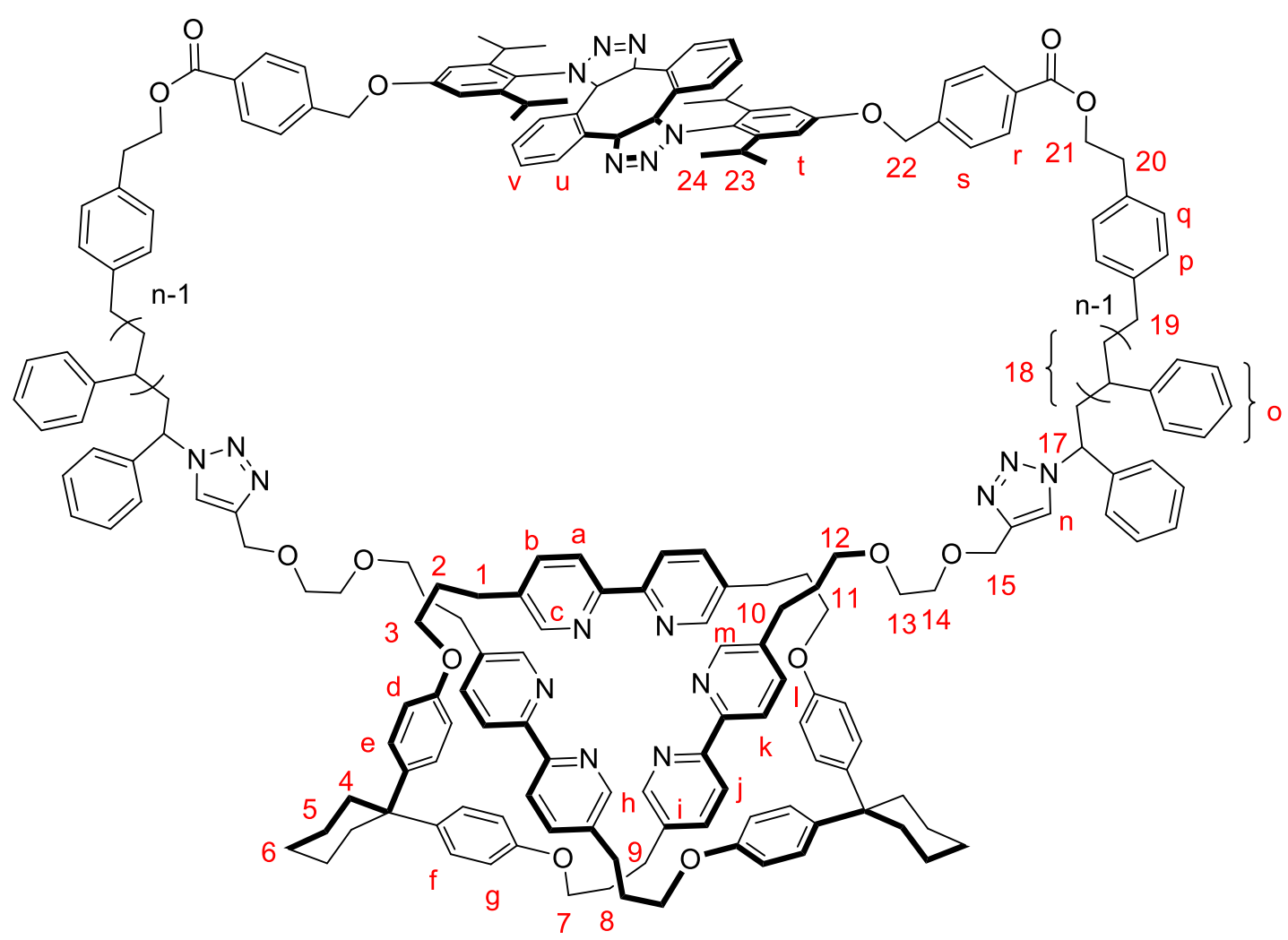

2

NaN_3 (34.0 mg, 523 µmol) was added to the solution of [Fe**2**](PF_6)$_2$ (200 mg, 17.4 µmol) in DMF (60 mL). The reaction mixture was stirred at 120 °C for 0.5 h. After completion, the crude product was precipitated from water (100 mL) and filtrated on celite, followed by washing the solid with water and MeOH. The solid was redissolved in DCM and evaporated to dryness under vacuum. The crude product was purified by SEC to give pure **2** as white solid (86 mg, yield 44%, $M_{n(absolute)}$ = 11852 Da, $Đ$ = 1.02).

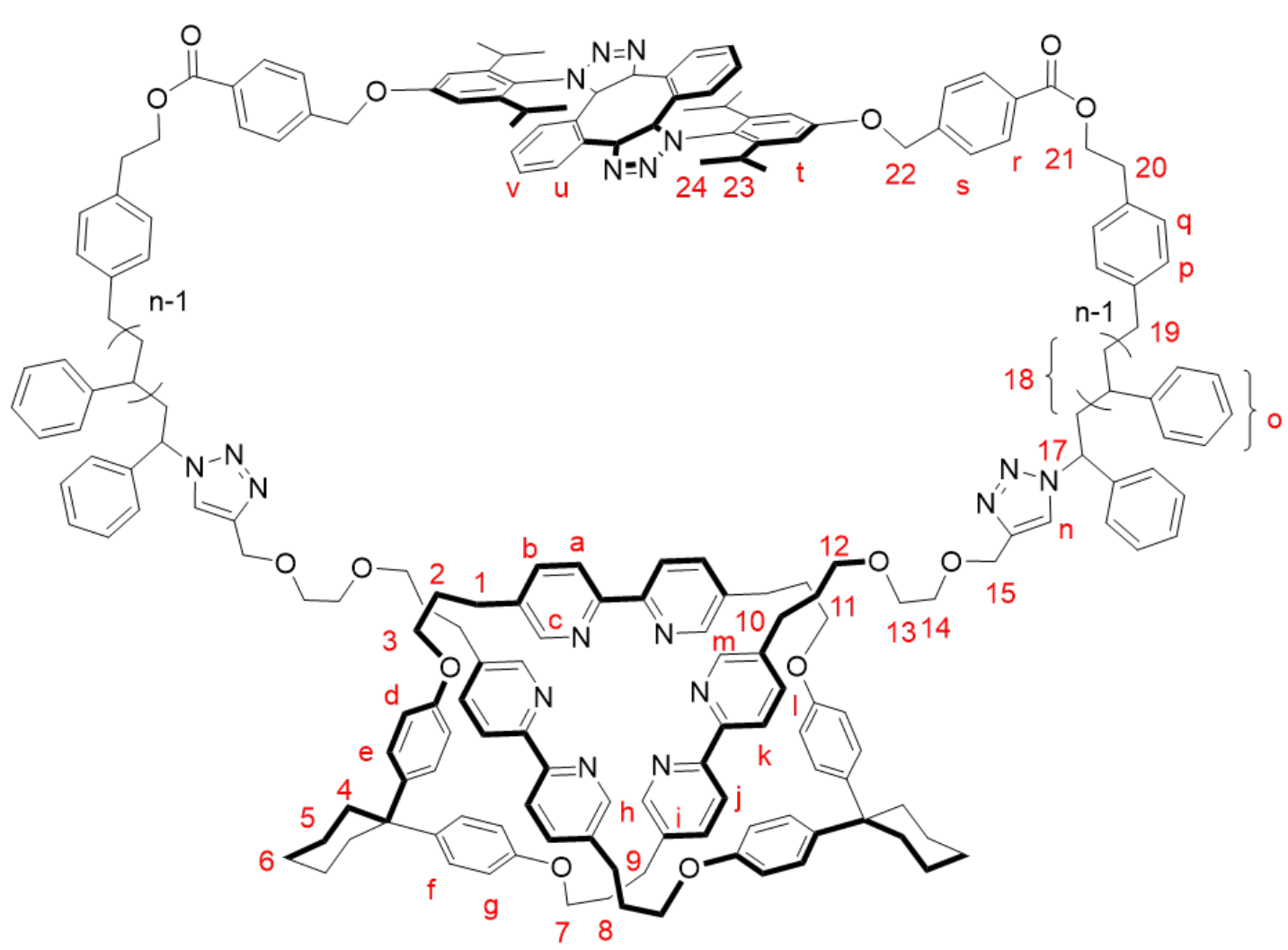

5

NaN_3 (46.2 mg, 711 µmol) was added to the solution of [Fe**5**](PF_6)$_2$ (200 mg, 23.7 µmol) in DMF (60 mL). The reaction mixture was stirred at 120 °C for 0.5 h. After completion, the crude product was precipitated from water (100 mL) and filtrated on celite and wash the solid with water and MeOH. The solid was redissolved in DCM and evaporated to dryness under vacuum. The crude

product was purified by SEC to give **5** as white solid (131 mg, yield 68%, $M_{n(absolute)}$ = 9125 Da, $Đ$ = 1.02).

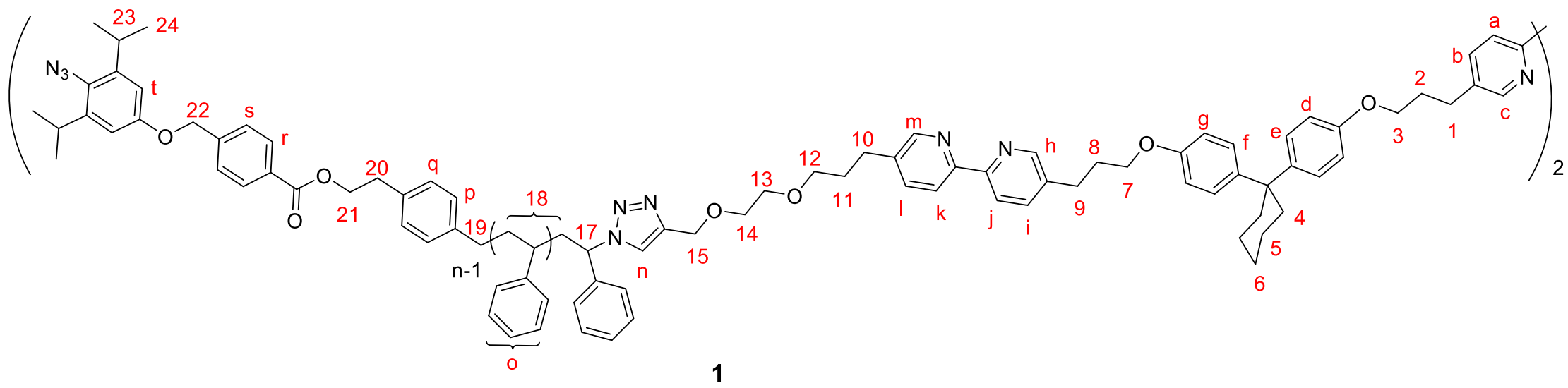

1

NaN_3 (44.2 mg, 680 µmol) was added to the solution of [Fe**1**]$(PF_6)_2$ (260 mg, 22.7 µmol) in DMF (60 mL). The reaction mixture was stirred at 120 °C for 0.5 h. After completion, the crude product was precipitated from water (100 mL) and filtrated on celite and wash the solid with water and MeOH. The solid was redissolved in DCM and evaporated to dryness under vacuum (245 mg, yield 97%). 100 mg of the crude product was purified by SEC to give pure **1** as white solid (80 mg, yield 78%, $M_{n(absolute)}$ = 10436 Da, $Đ$ = 1.03).

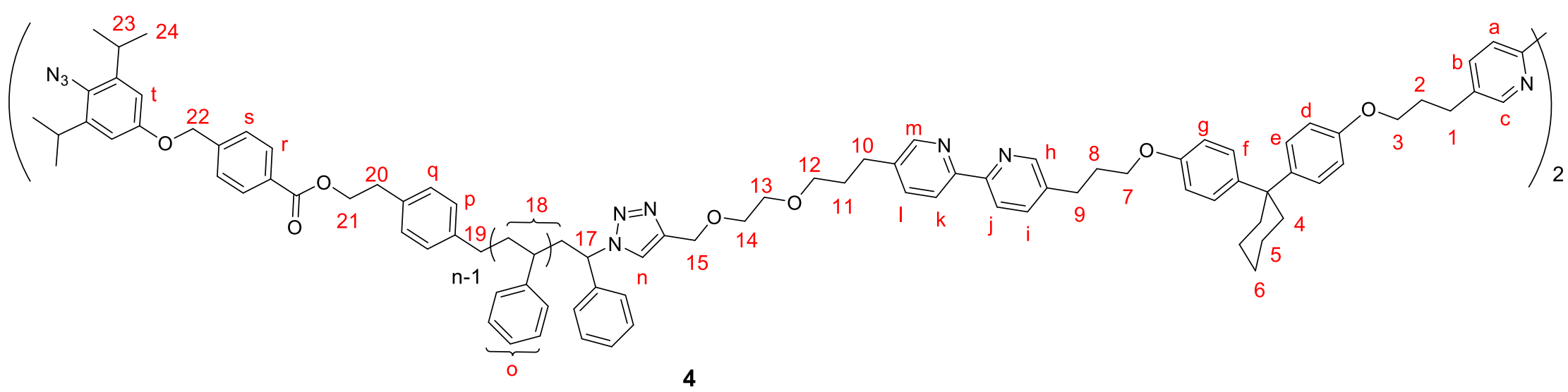

4

NaN_3 (40.3 mg, 619 µmol) was added to the solution of [Fe**4**]$(PF_6)_2$ (170 mg, 20.6 µmol) in DMF (30 mL). The reaction mixture was stirred at 120 °C for 0.5 h. After completion, the crude product was precipitated from water (100 mL) and filtrated on celite and wash the solid with water and MeOH. The solid was redissolved in DCM and evaporated to dryness under vacuum to give crude product as pale brown solid (160 mg, yield 98%). 55 mg of the crude product was purified by SEC to give pure **4** as white solid (40 mg, yield 71%, $M_{n(absolute)}$ = 8624 Da, $Đ$ = 1.04).

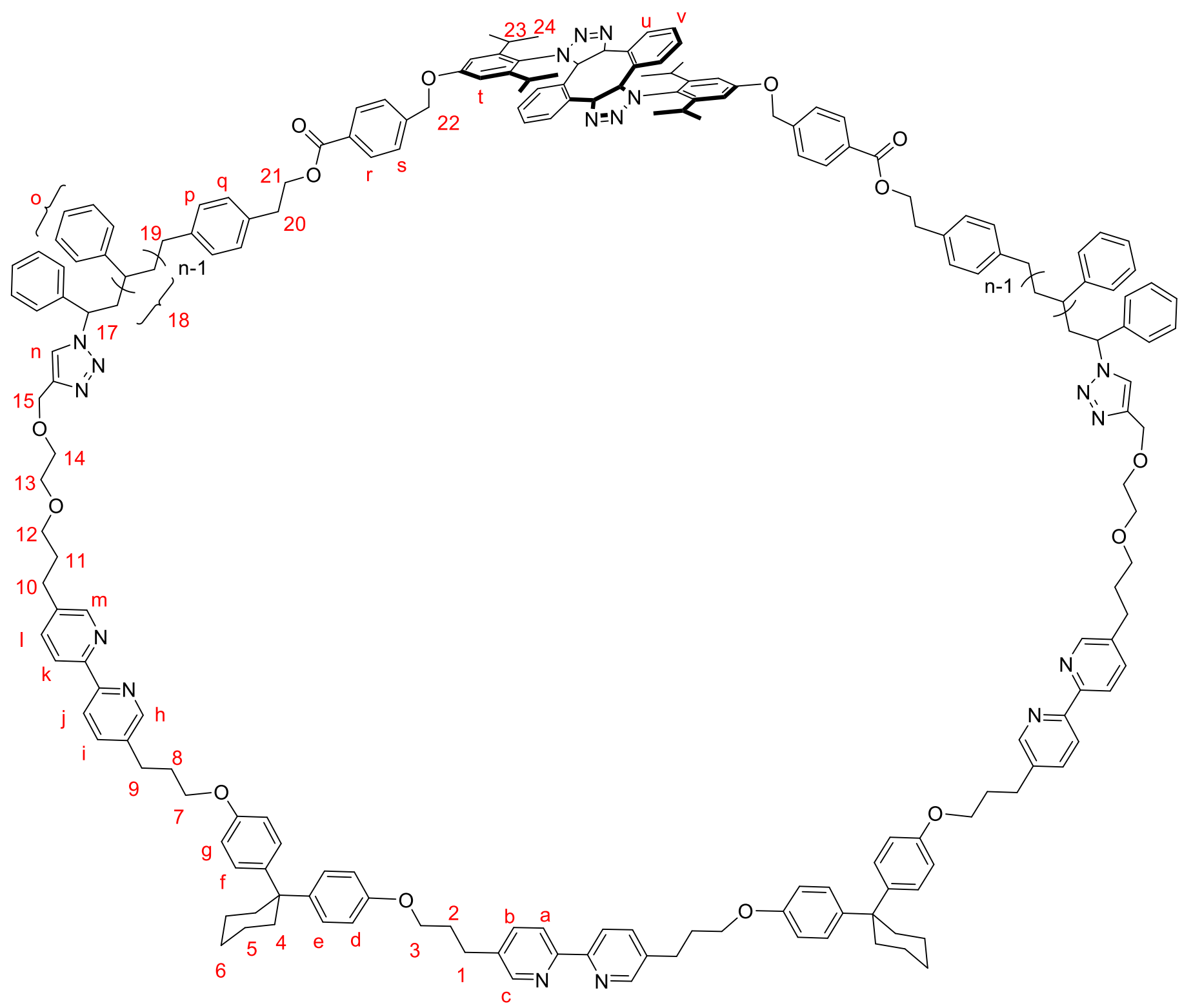

3

To a stirred solution of **S23** (49.1 mg, 245 μmol) in THF (50 mL) was added the solution of **1** (145 mg, 12.3 μmol) in THF (20 mL) dropwise for 24 hours and then the reaction mixture was stirred for another 5 hours. After completion, the solvent was evaporated to dryness. The crude product was purified by SEC to give pure **3** as white solid (65 mg, yield 44%, $M_{n(absolute)}$ = 11869 Da, $Đ$ = 1.05).

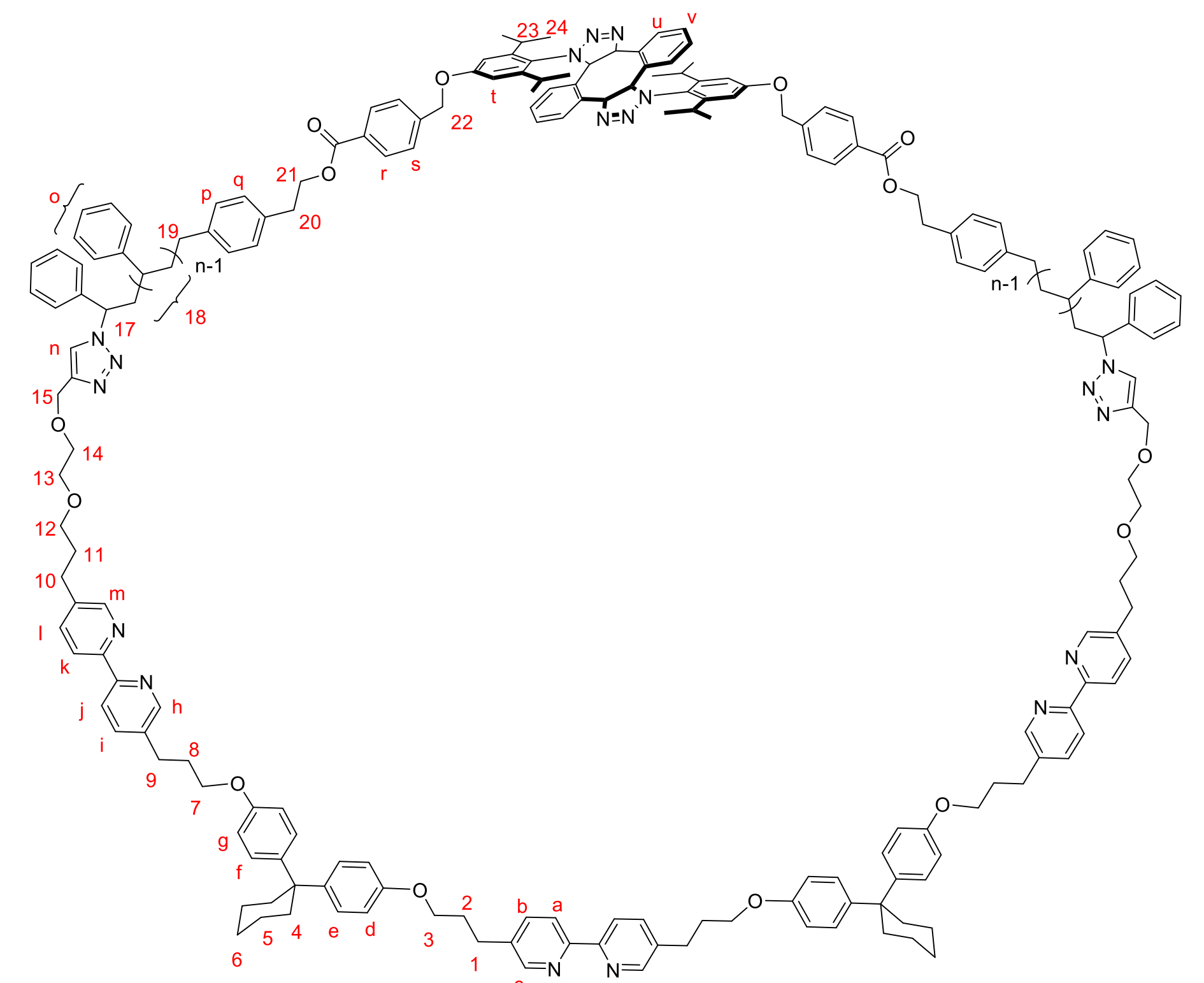

6

To a stirred solution of **S23** (53.1 mg, 265 µmol) in THF (80 mL) was added the solution of **4** (105 mg, 13.3 µmol) in THF (30 mL) dropwise for 24 hours and then the reaction mixture was stirred for another 5 hours. After completion, the solvent was evaporated to dryness. The crude product was purified by SEC to give pure **6** as white solid (39 mg, yield 36%, $M_{n(absolute)}$ = 9076 Da, *Đ* = 1.02).

5.7 ^{1}H NMR, MALDI-TOF-MS spectra, GPC analysis and DSC curves of 4, 5, 6

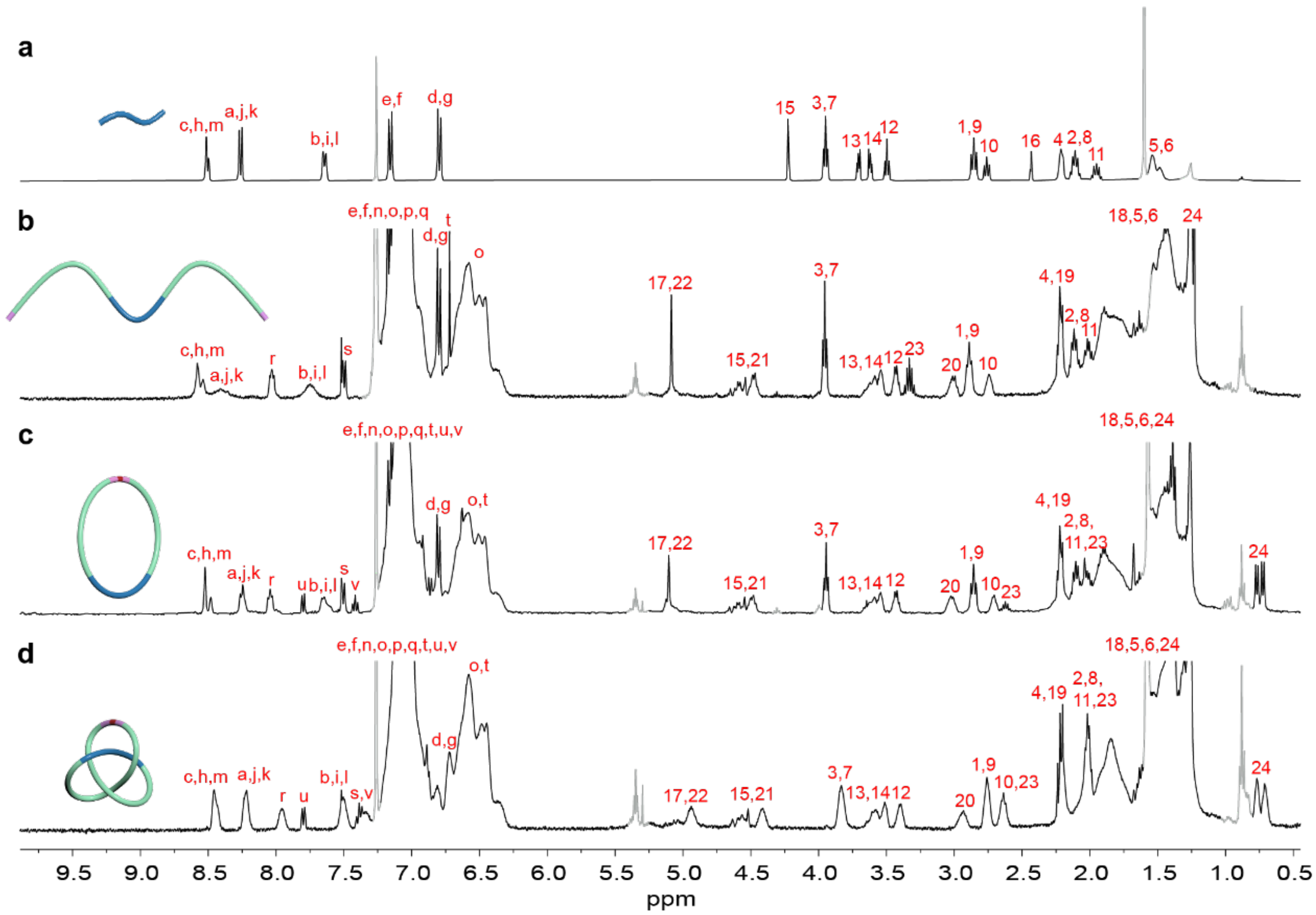

Figure 25. ^{1}H NMR spectra (400 MHz, $CDCl_3$, 298 K) of (a) **L1**; (b) **4**; (c) **6**; (d) **5**.

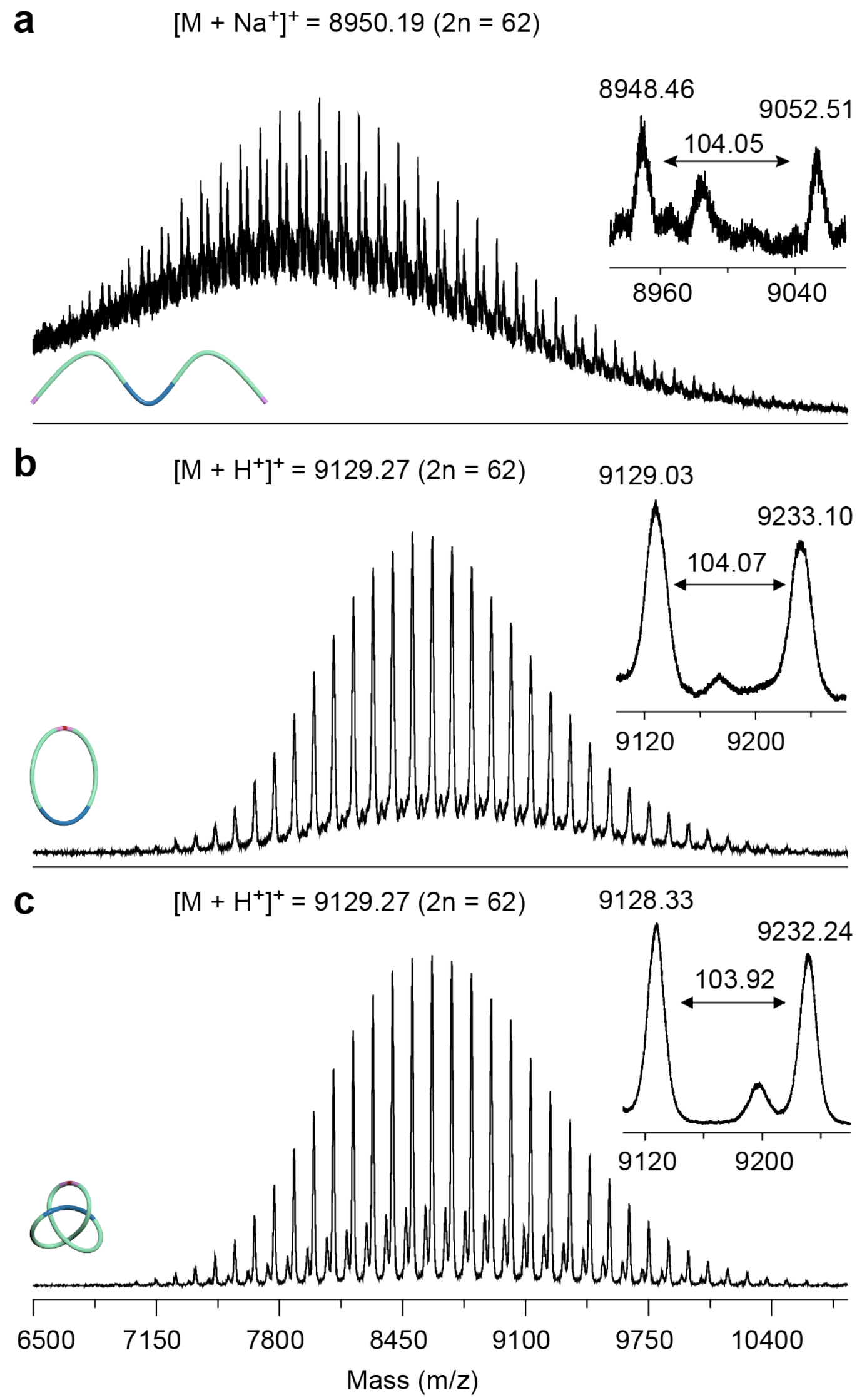

Figure 26. MALDI-TOF-MS spectra of (a) **4**; (b) **6**; (c) **5**.

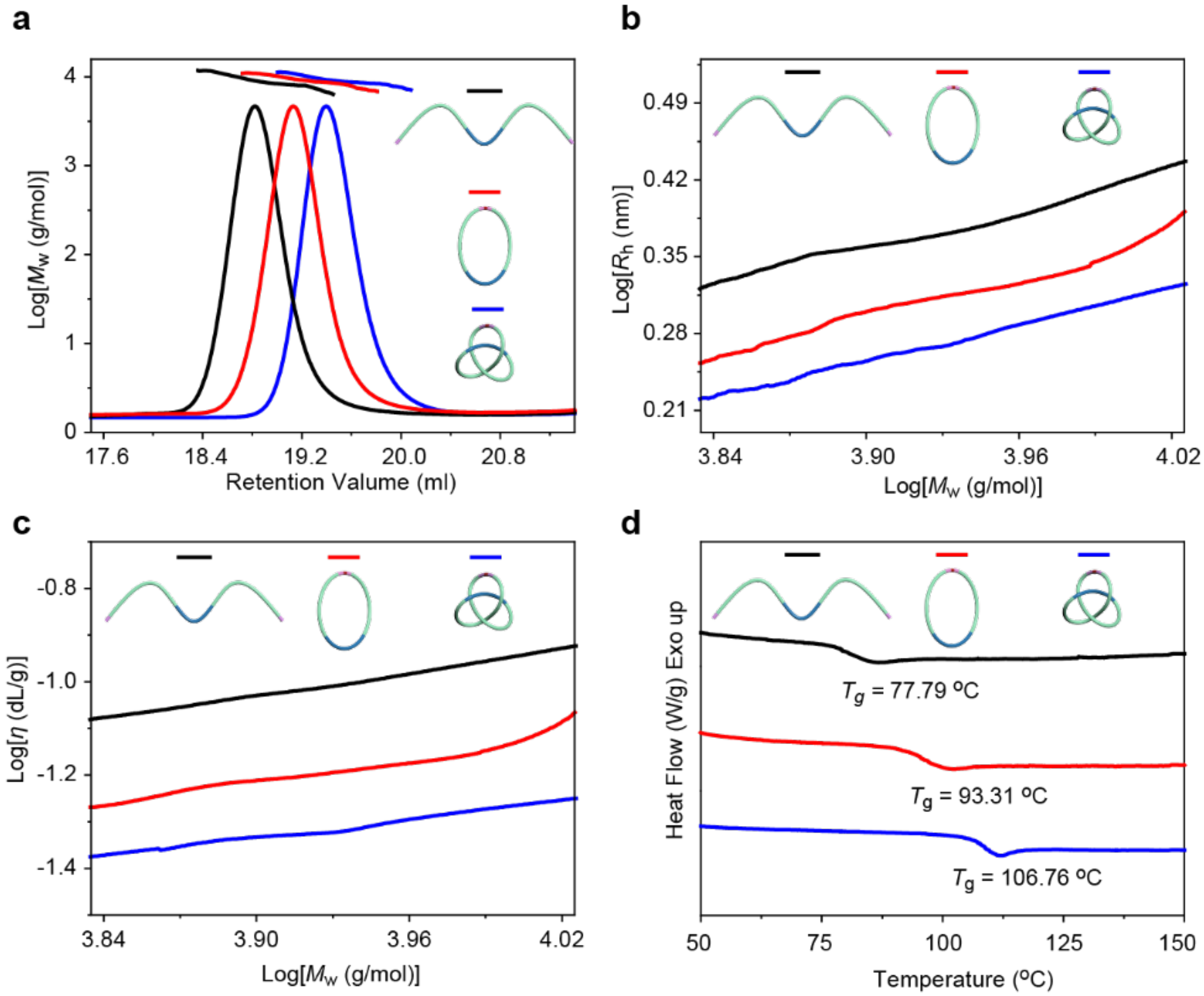

Figure 27. (a) GPC curves and Logarithm of molar mass versus elution time; (b) Logarithm of [R_h] versus the logarithm of molar mass; (c) Logarithm of [η] versus the logarithm of molar mass (Mark-Houwink-Sakurada plots, $[\eta]_{cyclic}/[\eta]_{linear} = 0.64$, $[\eta]_{knotted}/[\eta]_{linear} = 0.49$, $[\eta]_{knotted}/[\eta]_{cyclic} = 0.76$); (d) Differential scanning calorimetry (DSC) curves (T_g were determined by onset temperatures) of **4**, **5**, and **6**.

Because direct determination of the macrocyclization yield in the metallated intermediate is not straightforward, the efficiency of ring closure was evaluated after demetallation by size-exclusion chromatography (SEC). Preparative SEC of the crude organic knotted polymers reveals a distinct higher-molecular-weight fraction attributable to intermolecular oligomerization. Integration of SEC peak areas indicates that this fraction accounts for ~43% of the material for the longer PS knot **2**, whereas the proportion of oligomeric by-products decreases and the isolated yield increases to ~72% for the shorter PS knot **5**, consistent with chain-length-dependent macrocyclization efficiency. The disappearance of the azide stretching band in the IR spectra further supports high end-group conversion. Representative SEC traces and integrations are provided in Figure 28.

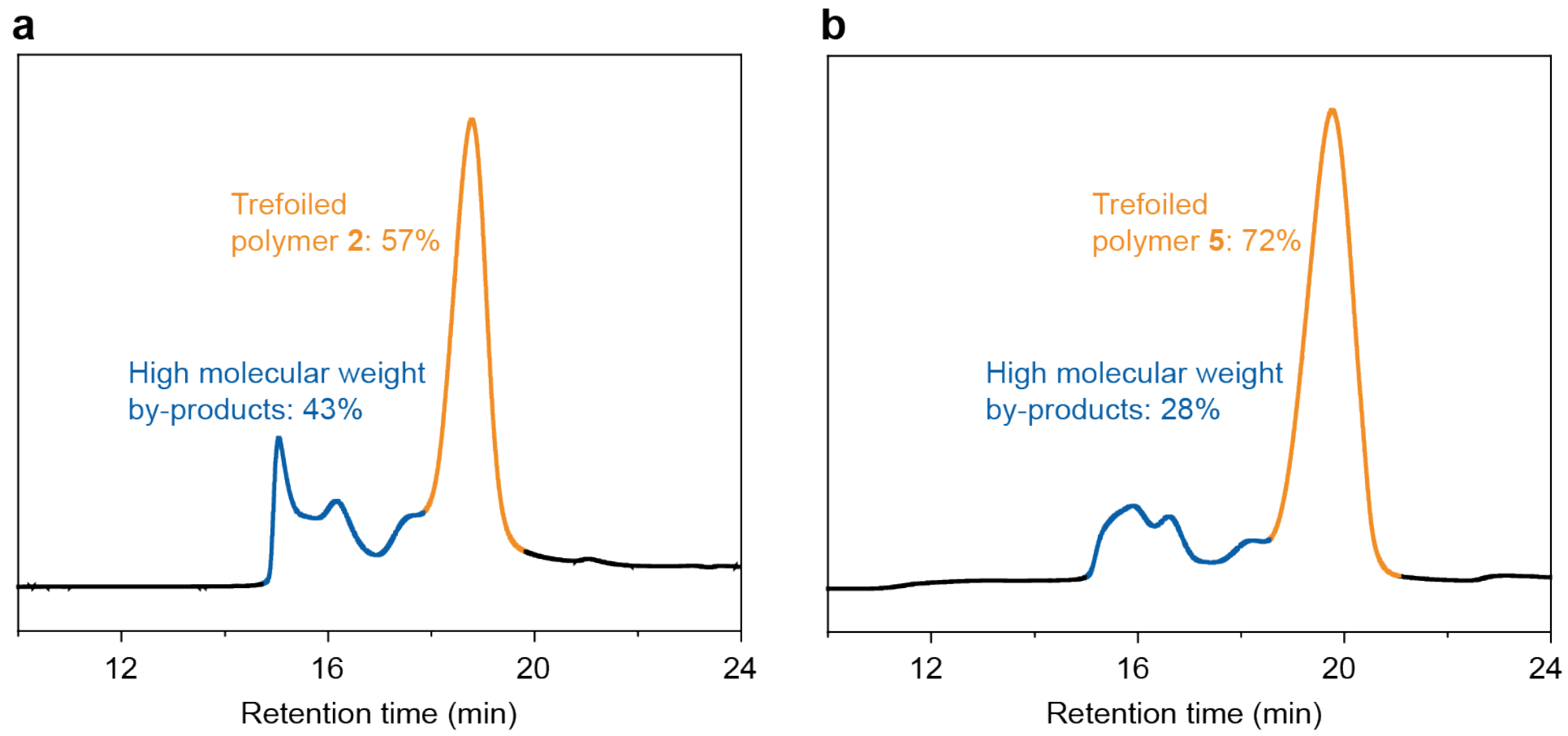

Figure 28. SEC traces of crude trefoil PS **2** and PS **5**, showing that the fraction of knotted polymer increases for the shorter-chain sample, consistent with chain-length-dependent macrocyclization efficiency.

5.8 Molecular models of L1, S17 and S18

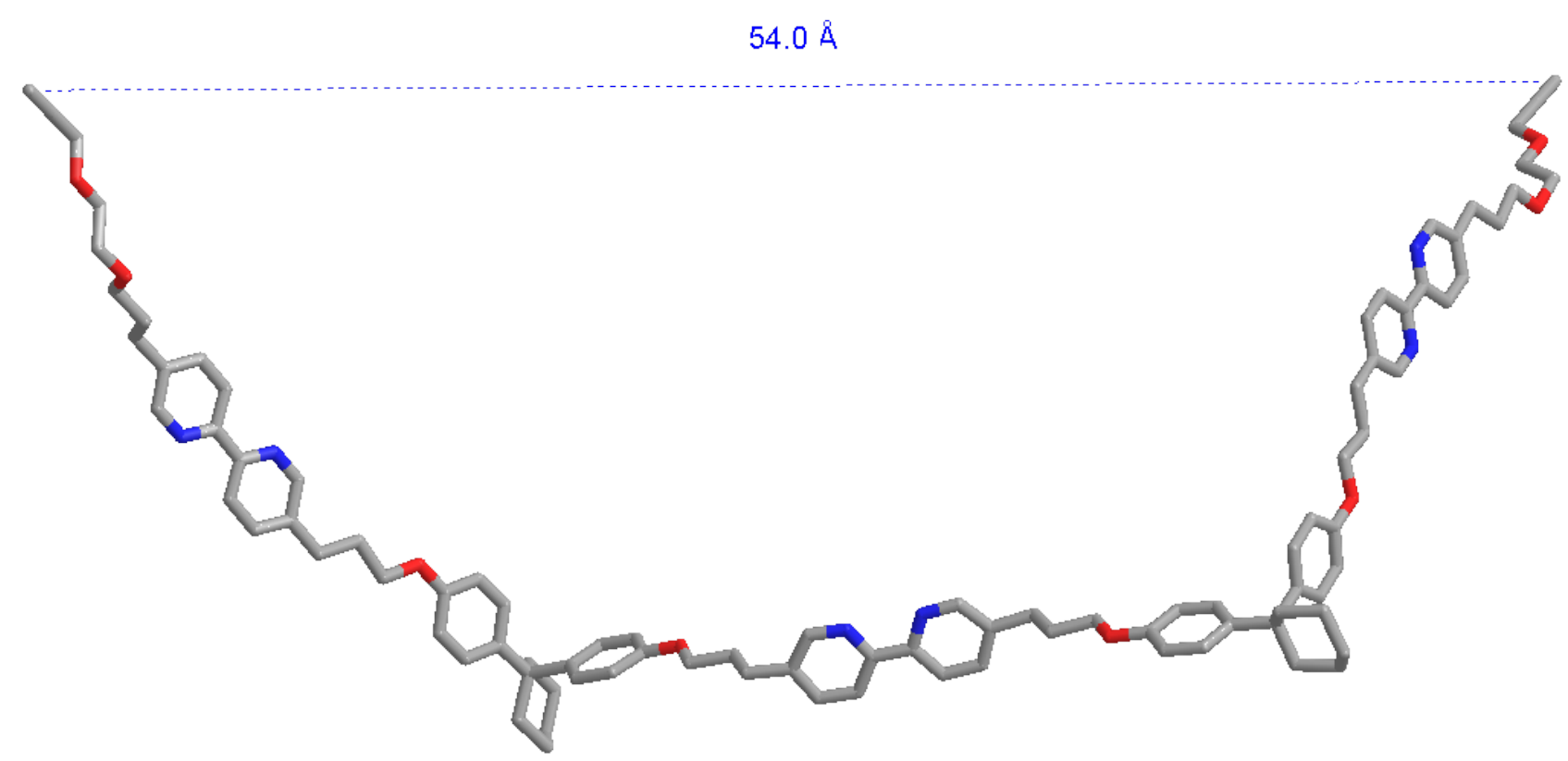

Figure 29. Molecular model of **L1**, which was established by Chem 3D 19.0.0.22 and minimized by MM2.

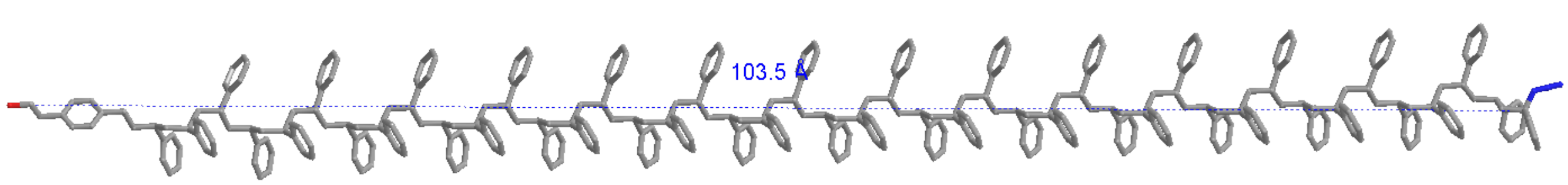

Figure 30. Molecular model of **S17**, which was established by Chem 3D 19.0.0.22 and minimized by MM2.

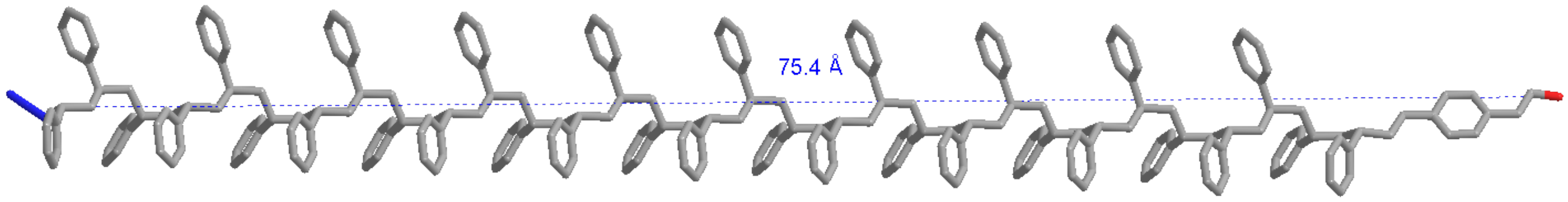

Figure 31. Molecular model of **S18**, which was established by Chem 3D 19.0.0.22 and minimized by MM2.

5.9 Variable-temperature NMR (VT-NMR) spectra of linear (1, 4), trefoiled (2, 5) and cyclic (3, 6) polymers

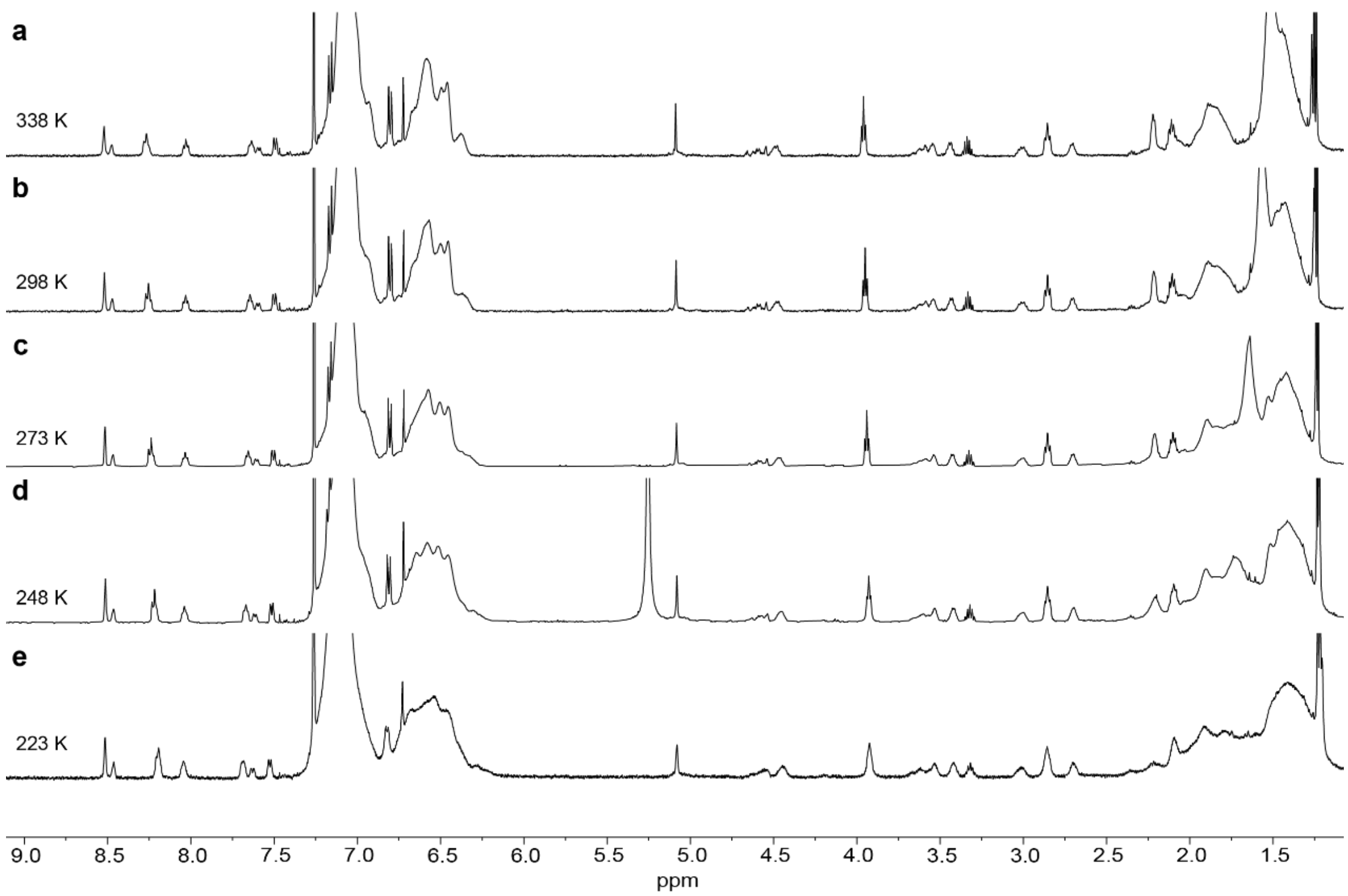

Figure 32. ¹H NMR spectra (500 MHz, $CDCl_3$) of **1** under different temperature (223 K, 248 K, 273 K, 298 K, and 338 K).

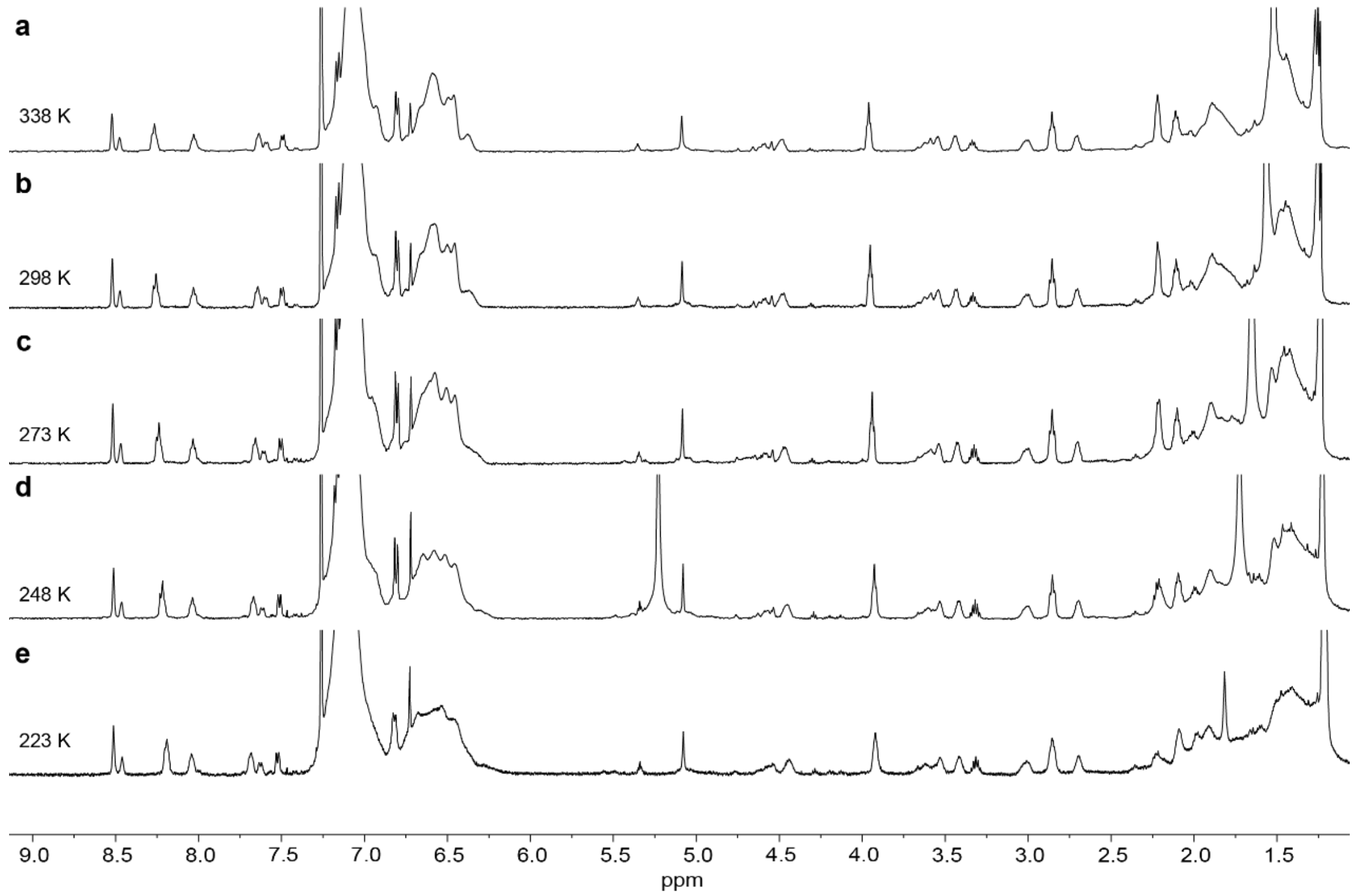

Figure 33. ^{1}H NMR spectra (500 MHz, $CDCl_3$) of **4** under different temperature (223 K, 248 K, 273 K, 298 K, and 338 K).

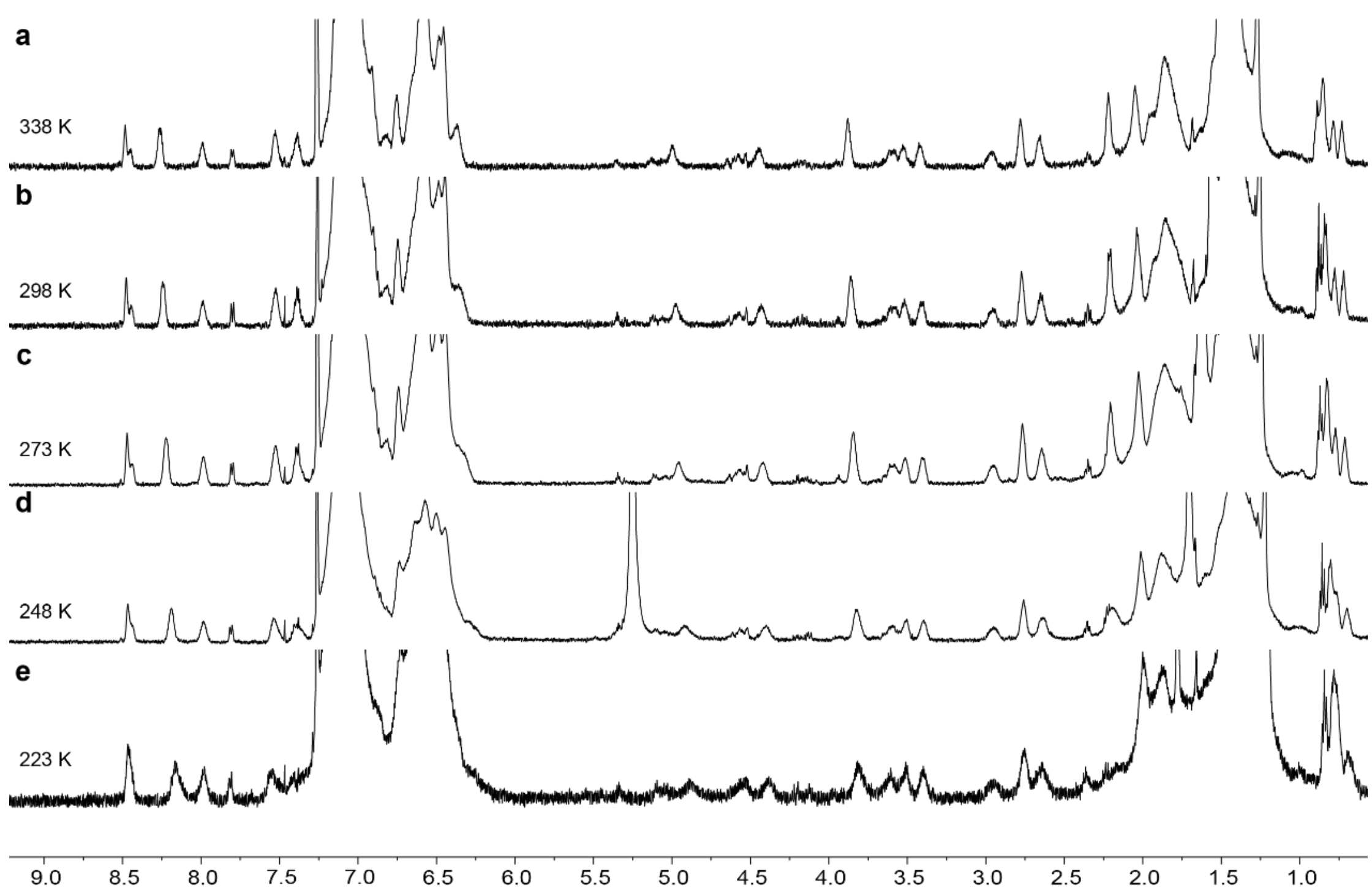

Figure 34. ^{1}H NMR spectra (500 MHz, $CDCl_3$) of **2** under different temperature (223 K, 248 K, 273 K, 298 K, and 338 K).

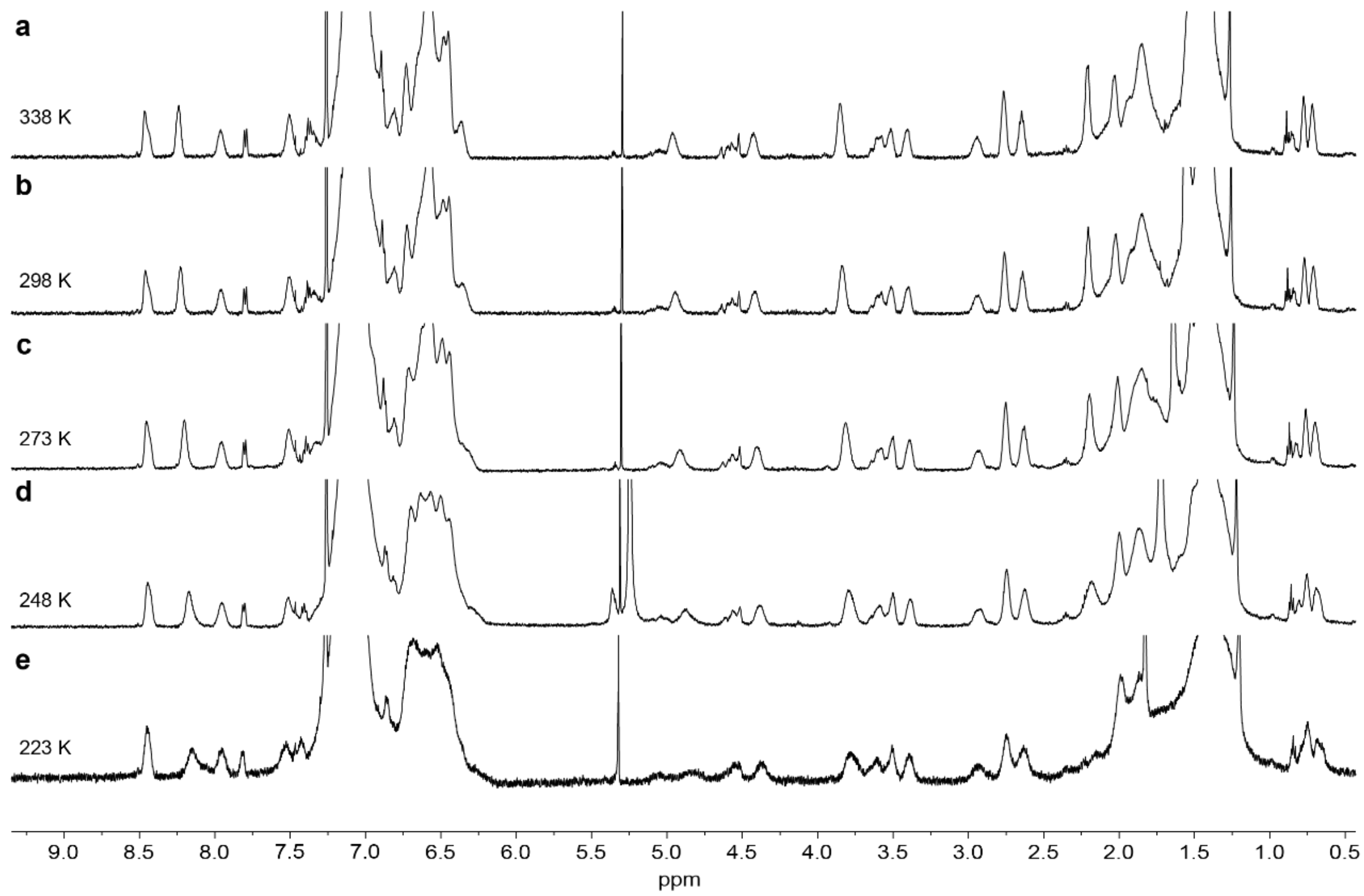

Figure 35. ^{1}H NMR spectra (500 MHz, $CDCl_3$) of **5** under different temperature (223 K, 248 K, 273 K, 298 K, and 338 K).

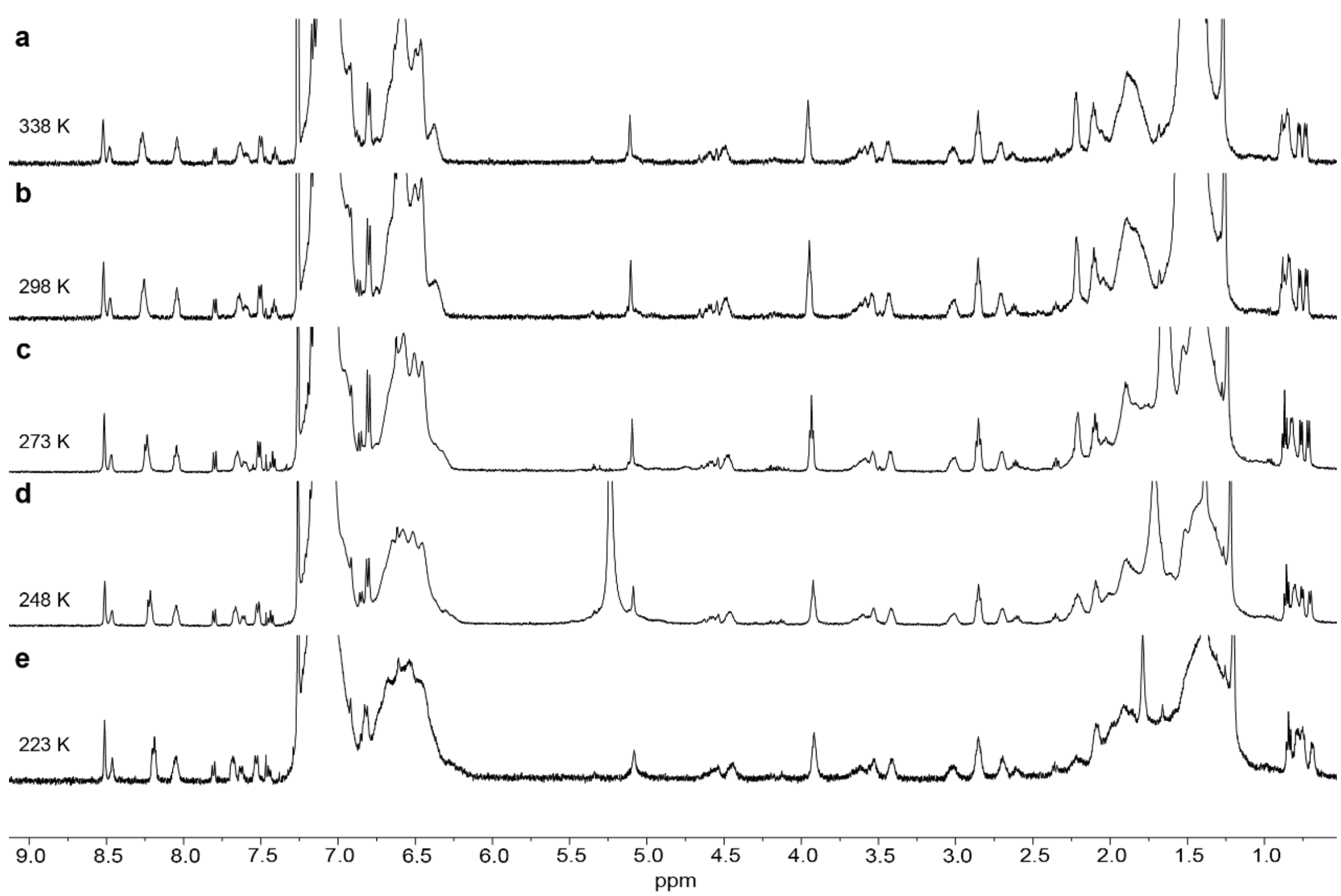

Figure 36. ^{1}H NMR spectra (500 MHz, $CDCl_3$) of **3** under different temperature (223 K, 248 K, 273 K, 298 K, and 338 K).

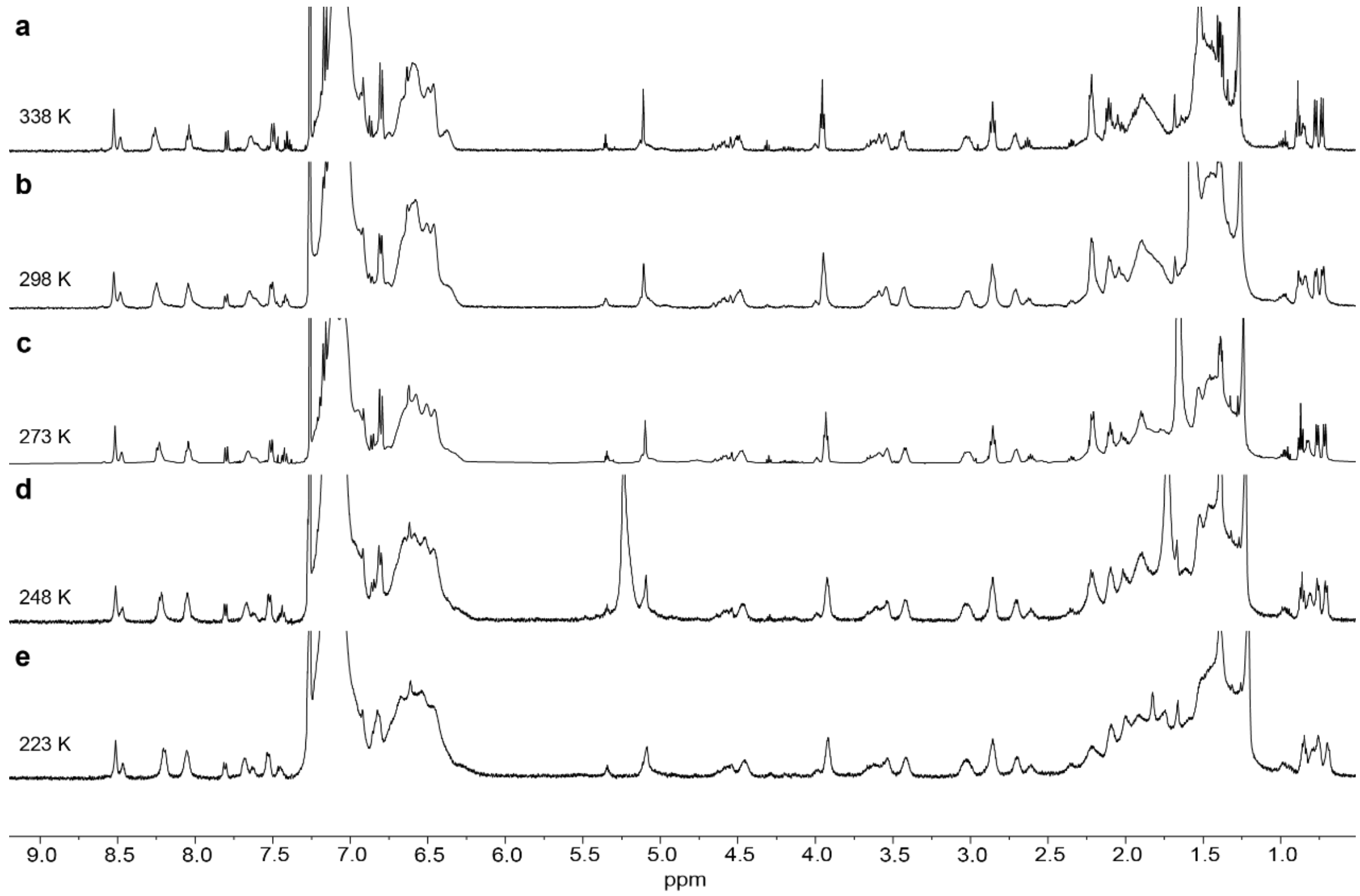

Figure 37. ^{1}H NMR spectra (500 MHz, $CDCl_3$) of **6** under different temperature (223 K, 248 K, 273 K, 298 K, and 338 K).

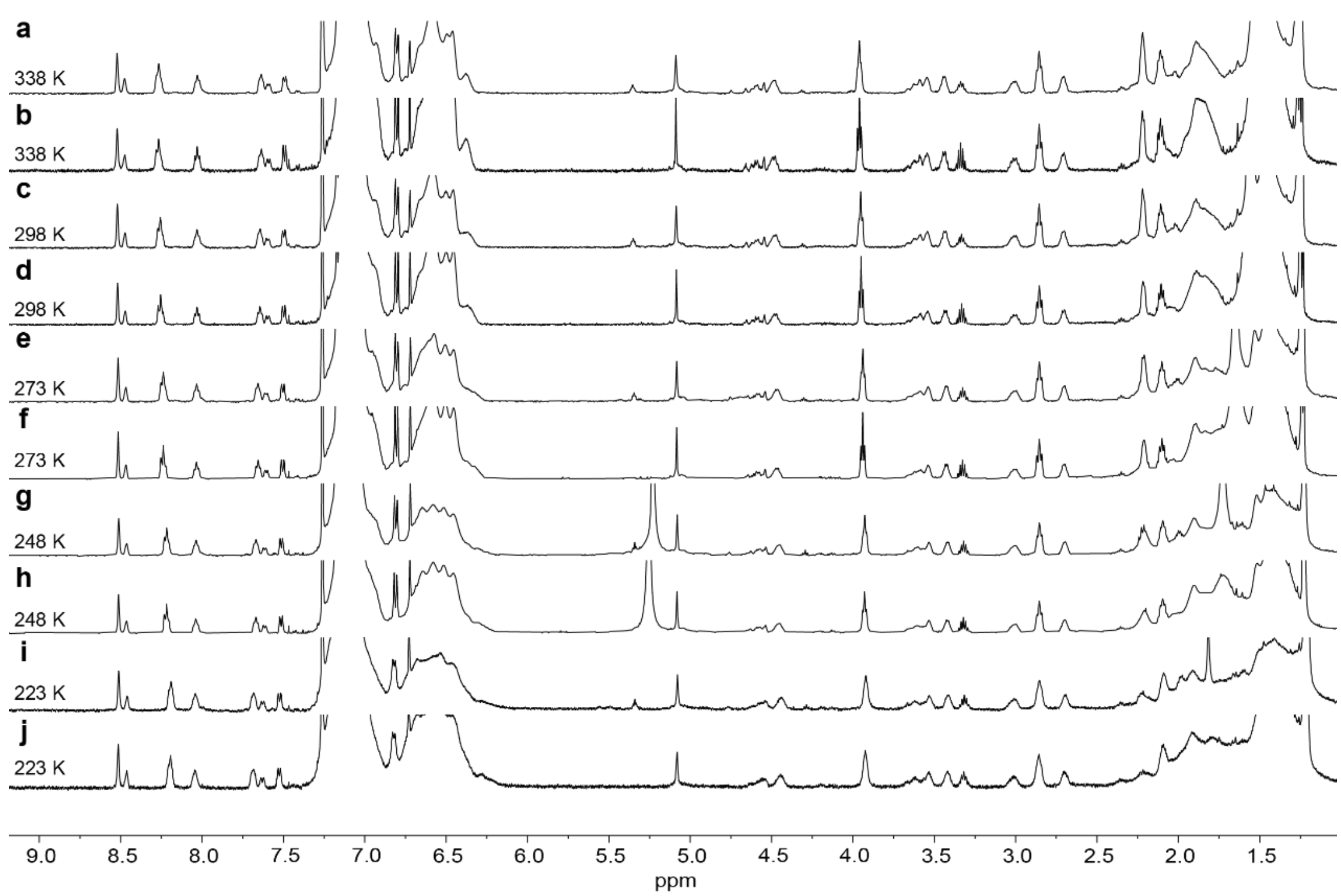

Figure 38. Comparison of the ^{1}H NMR spectra (500 MHz, $CDCl_3$) of **1** (b, d, f, h, j) and **4** (a, c, e, g, i) under different temperature (223 K, 248 K, 273 K, 298 K, and 338 K).

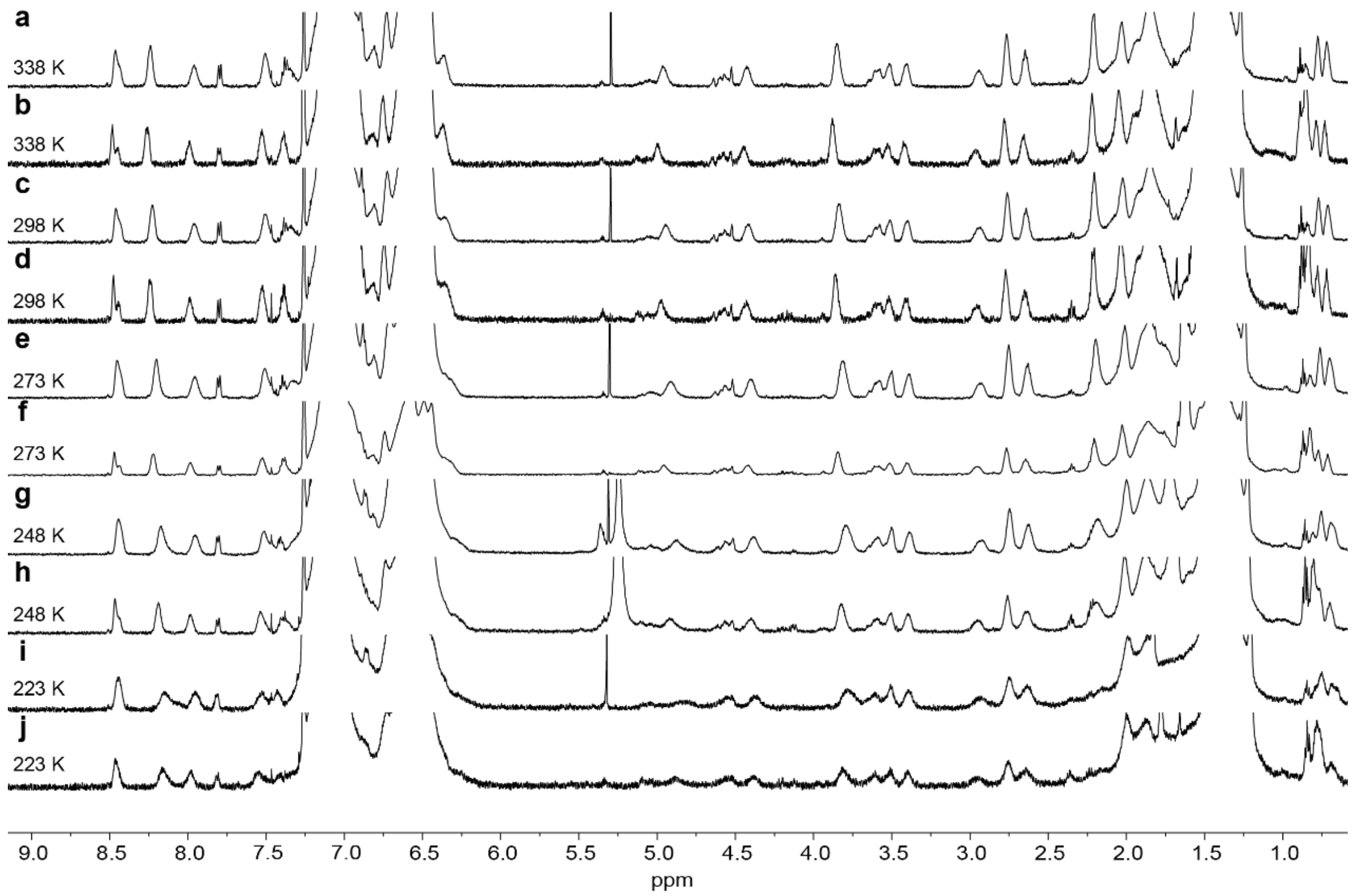

Figure 39. Comparison of the ^{1}H NMR spectra (500 MHz, $CDCl_3$) of **2** (b, d, f, h, j) and **5** (a, c, e, g, i) under different temperature (223 K, 248 K, 273 K, 298 K, and 338 K).

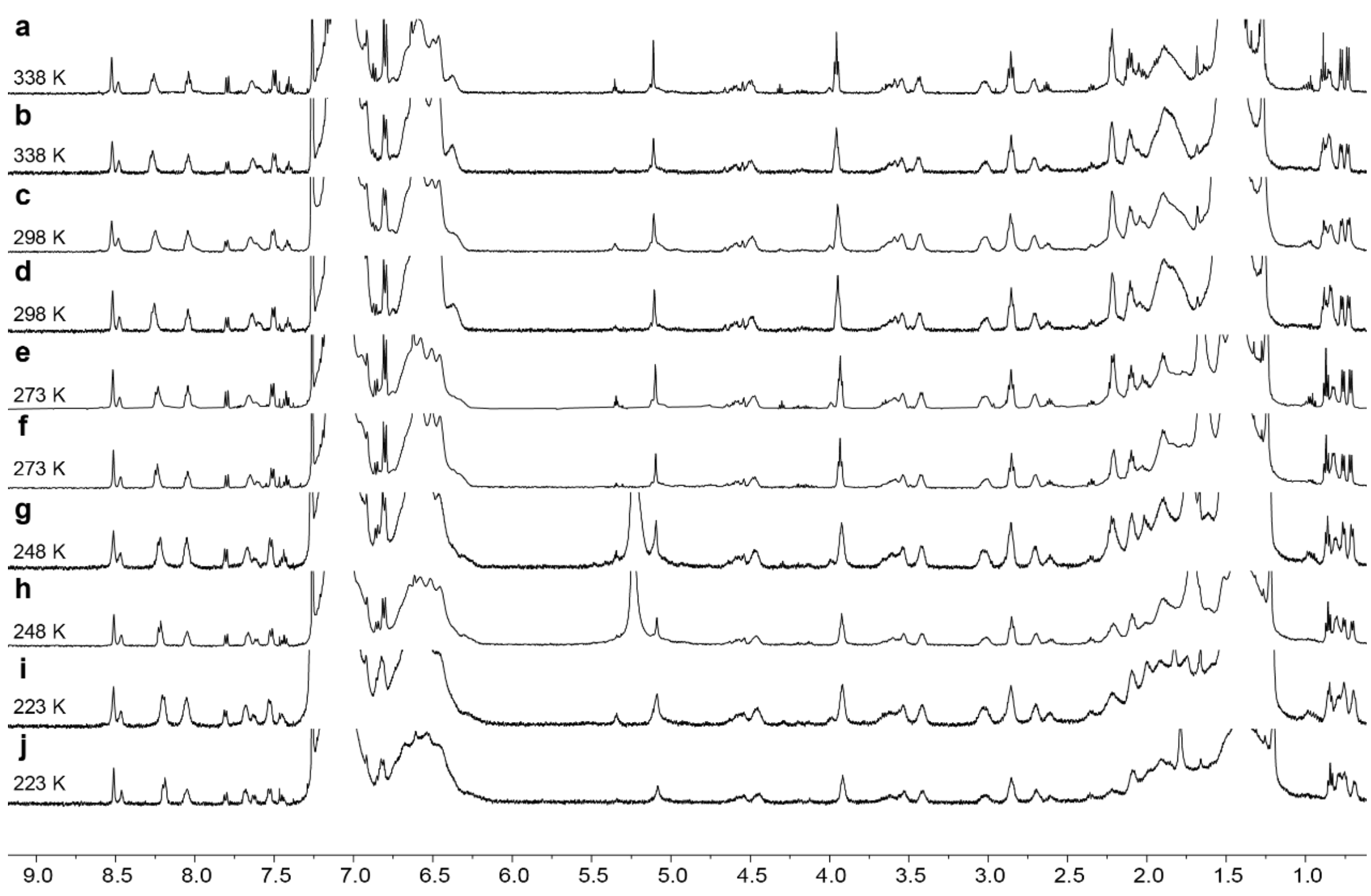

Figure 40. Comparison of the ^{1}H NMR spectra (500 MHz, $CDCl_3$) of **3** (b, d, f, h, j) and **6** (a, c, e, g, i) under different temperature (223 K, 248 K, 273 K, 298 K, and 338 K).

6. Thermal gravimetric analysis (TGA) of linear (1, 4), trefoiled (2, 5) and cyclic (3, 6) polymers

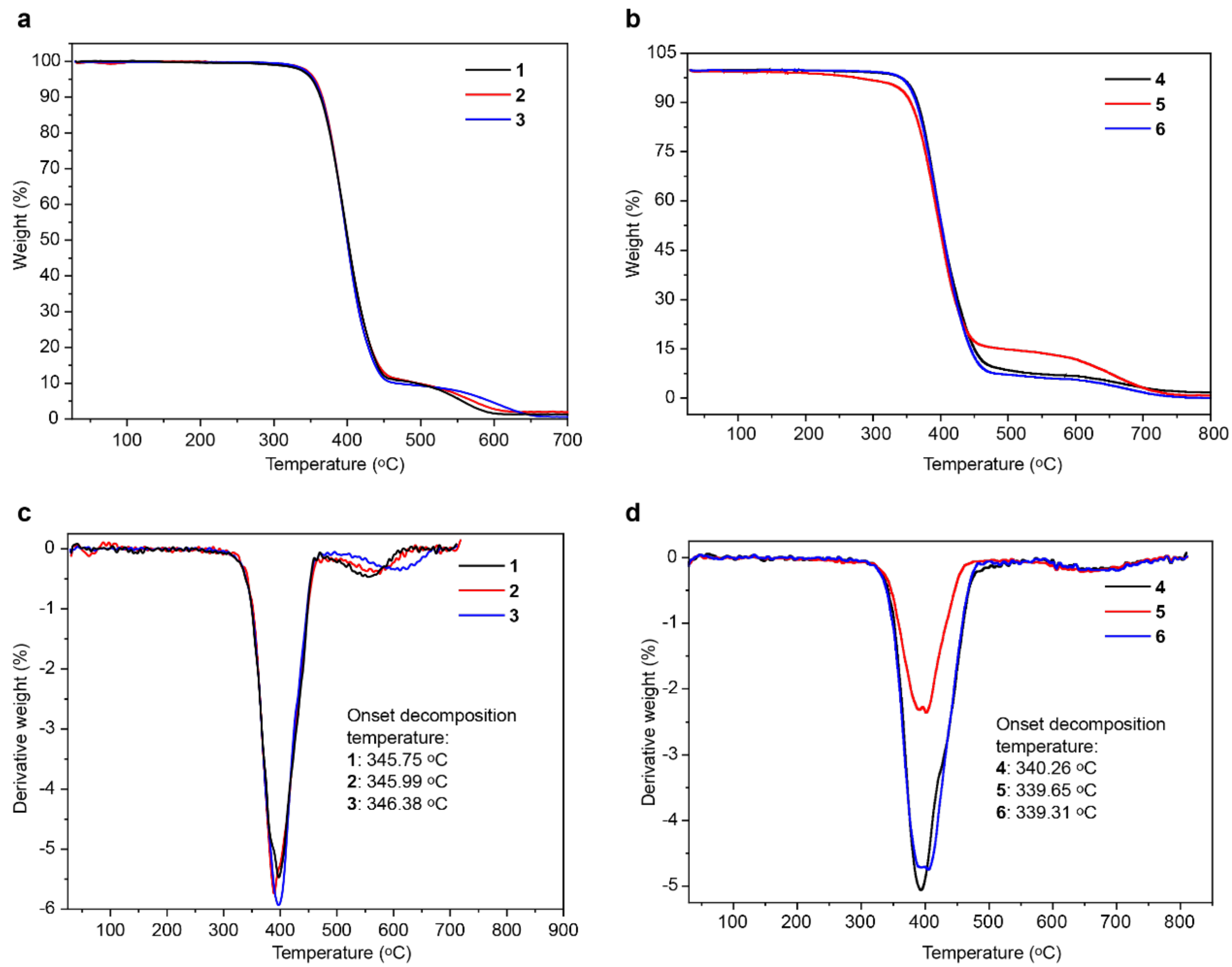

Figure 41. Thermogravimetric analysis (TGA) curves and corresponding derivative curves of (a, c) **1**, **2**, **3**; (b, d) **4**, **5**, **6**.

7. UV-Vis and fluorescence emission spectra of linear (1, 4), trefoiled (2, 5) and cyclic (3, 6) polymers

Absorbance spectra of the polymers (concentration: c ≈ 1.0 × 10^{-6} mol/L, with THF as solvent) were performed on a Shimadzu UV-1800 UV-Vis spectrophotometer. Fluorescence emission spectra of the polymers (concentration: $c \approx 2.0 \times 10^{-7}$ mol/L, with THF as solvent) were performed on a Shimadzu RF-6000 spectrofluorophotometer with 260 nm excitation wavelength.

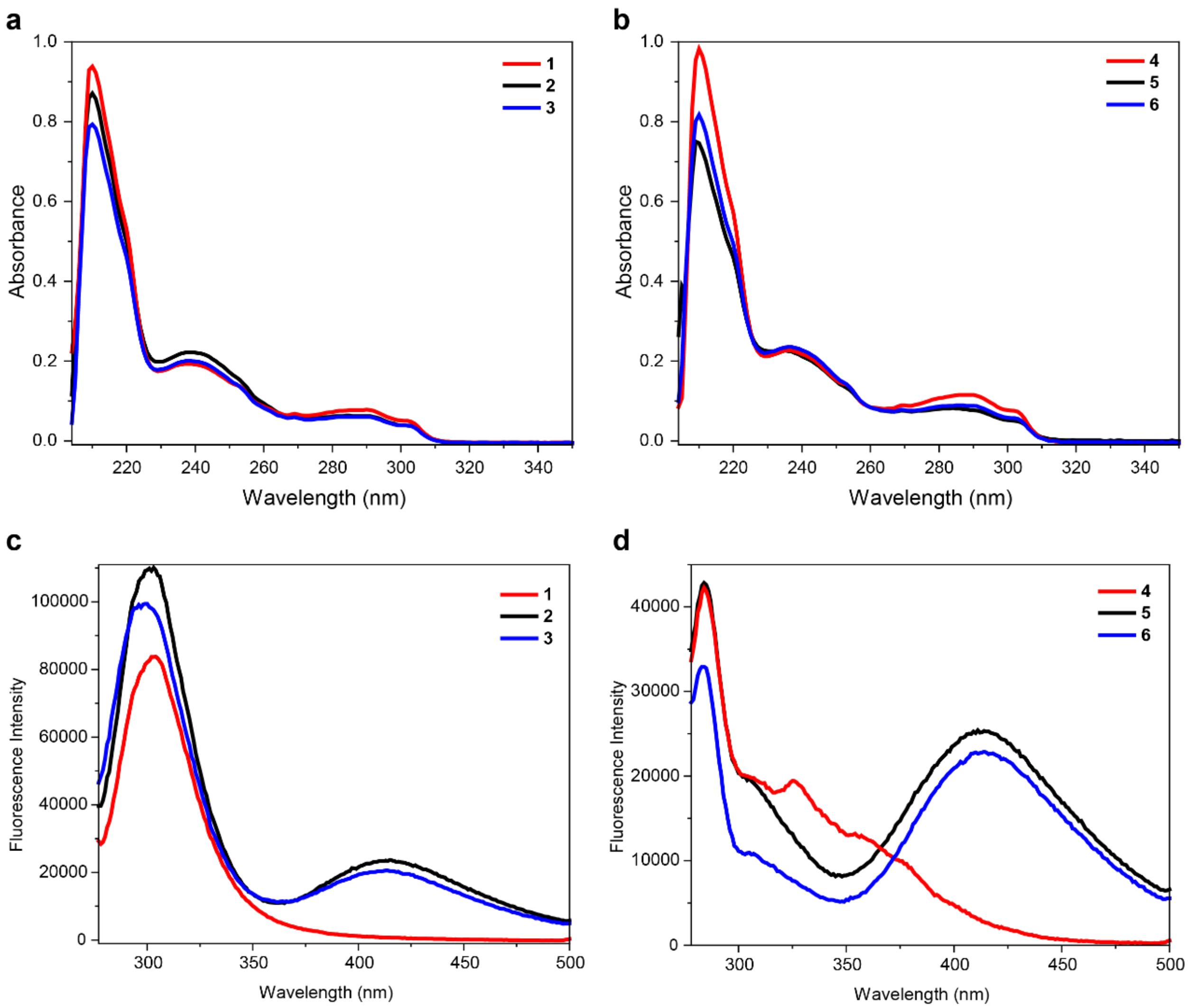

Figure 42. (a) UV-vis spectra of **1**, **2**, **3** and (b) **4**, **5**, **6**; (c) Fluorescence emission spectra of **1**, **2**, **3** and (d) **4**, **5**, **6**.

8. DOSY spectra of 1, 2, 3, 4, 5, 6 in different solvents

Table 3. Diffusion coefficients of **1**, **2**, **3, 4, 5, 6** in different solvents.

Polymer	*D* (cm²/s) in $CDCl_3$ at 298 K	*D* (cm²/s) in THF-d_4 at 298 K	[a]*D* (cm²/s) in DMSO-d_6 at 298 K	*D* (cm²/s) in TCE-d_2 at 298 K
**1**	1.20×10^{-6}	1.39×10^{-6}	3.76×10^{-7}	4.51×10^{-7}
**2**	3.08×10^{-6}	2.14×10^{-6}	4.67×10^{-7}	5.40×10^{-7}
**3**	3.08×10^{-6}	2.14×10^{-6}	4.34×10^{-7}	5.80×10^{-7}
**4**	3.08×10^{-6}	2.48×10^{-6}	6.24×10^{-7}	6.71×10^{-7}
**5**	3.31×10^{-6}	3.08×10^{-6}	7.76×10^{-7}	8.34×10^{-7}
**6**	3.08×10^{-6}	3.31×10^{-6}	6.71×10^{-7}	7.22×10^{-7}

a: 15% $CDCl_3$ by volume was added to facilitate the dissolution of the polymers.

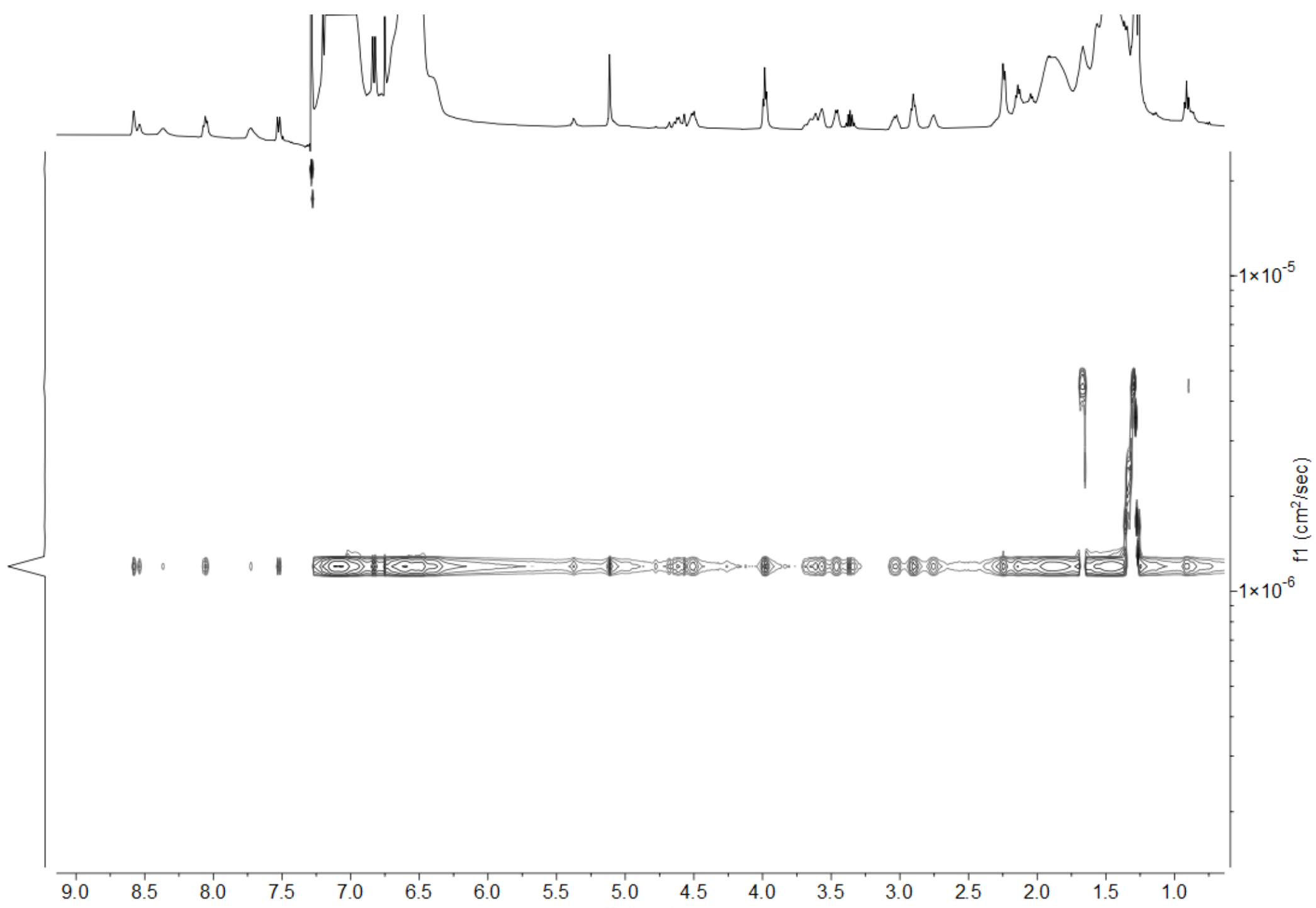

Figure 43. DOSY spectrum (500 MHz, $CDCl_3$, 298 K, gradient duration (δ) = 6.4 ms, diffusion time Δ = 45 ms) of **1**.

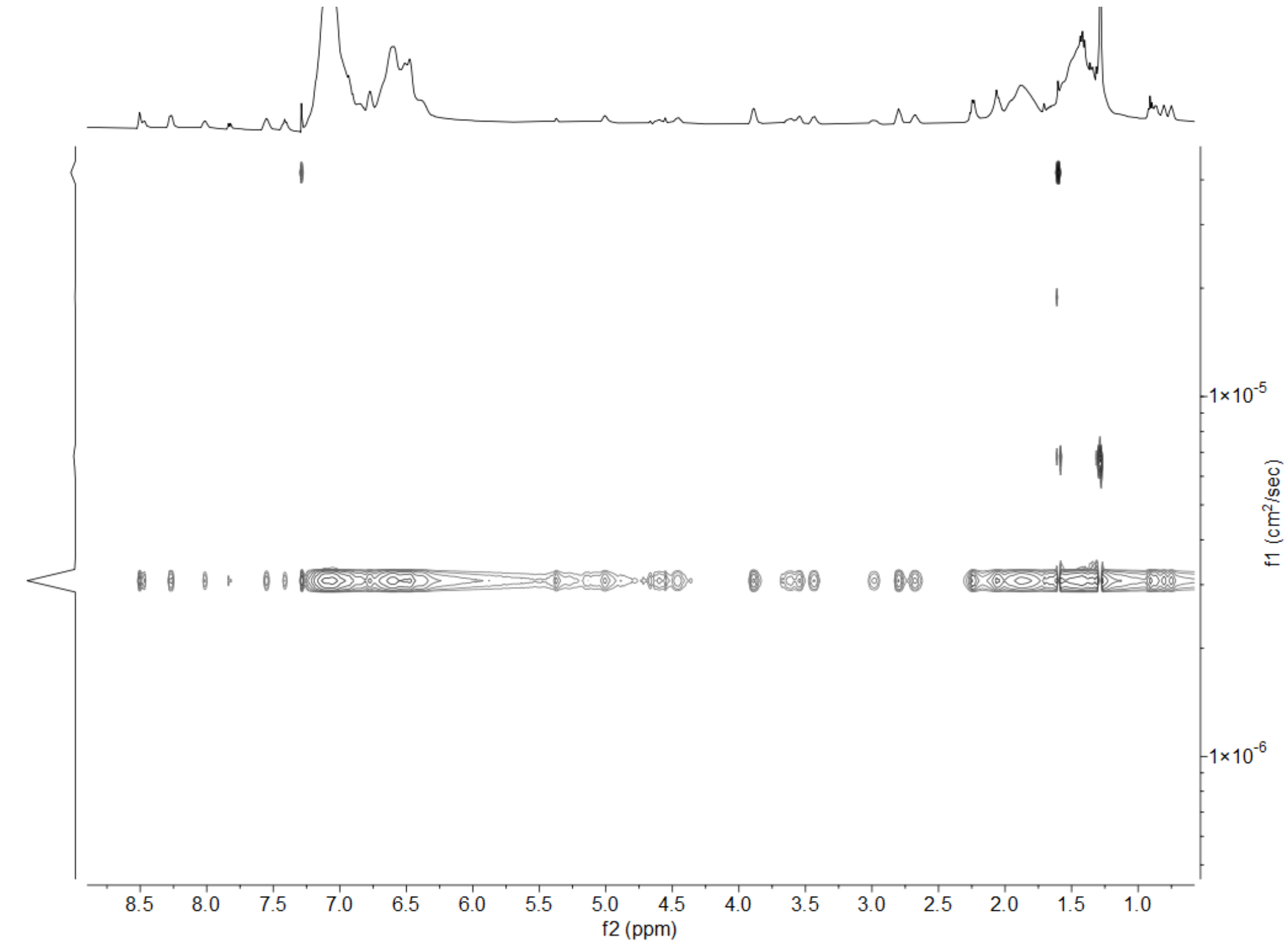

Figure 44. DOSY spectrum (500 MHz, $CDCl_3$, 298 K, gradient duration (δ) = 4 ms, diffusion time Δ = 45 ms) of **2**.

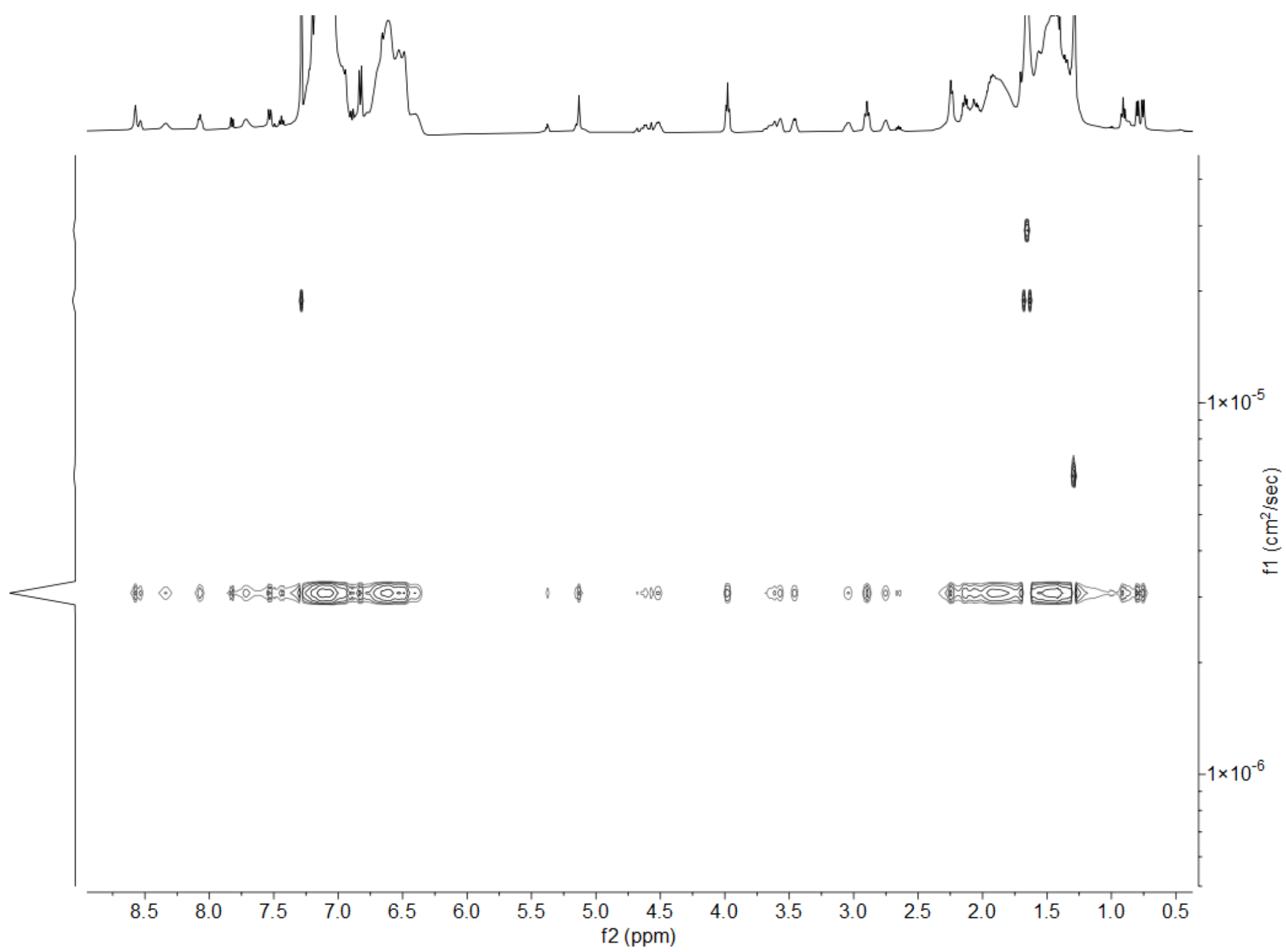

Figure 45. DOSY spectrum (500 MHz, $CDCl_3$, 298 K, gradient duration (δ) = 4.44 ms, diffusion time Δ = 45 ms) of **3**.

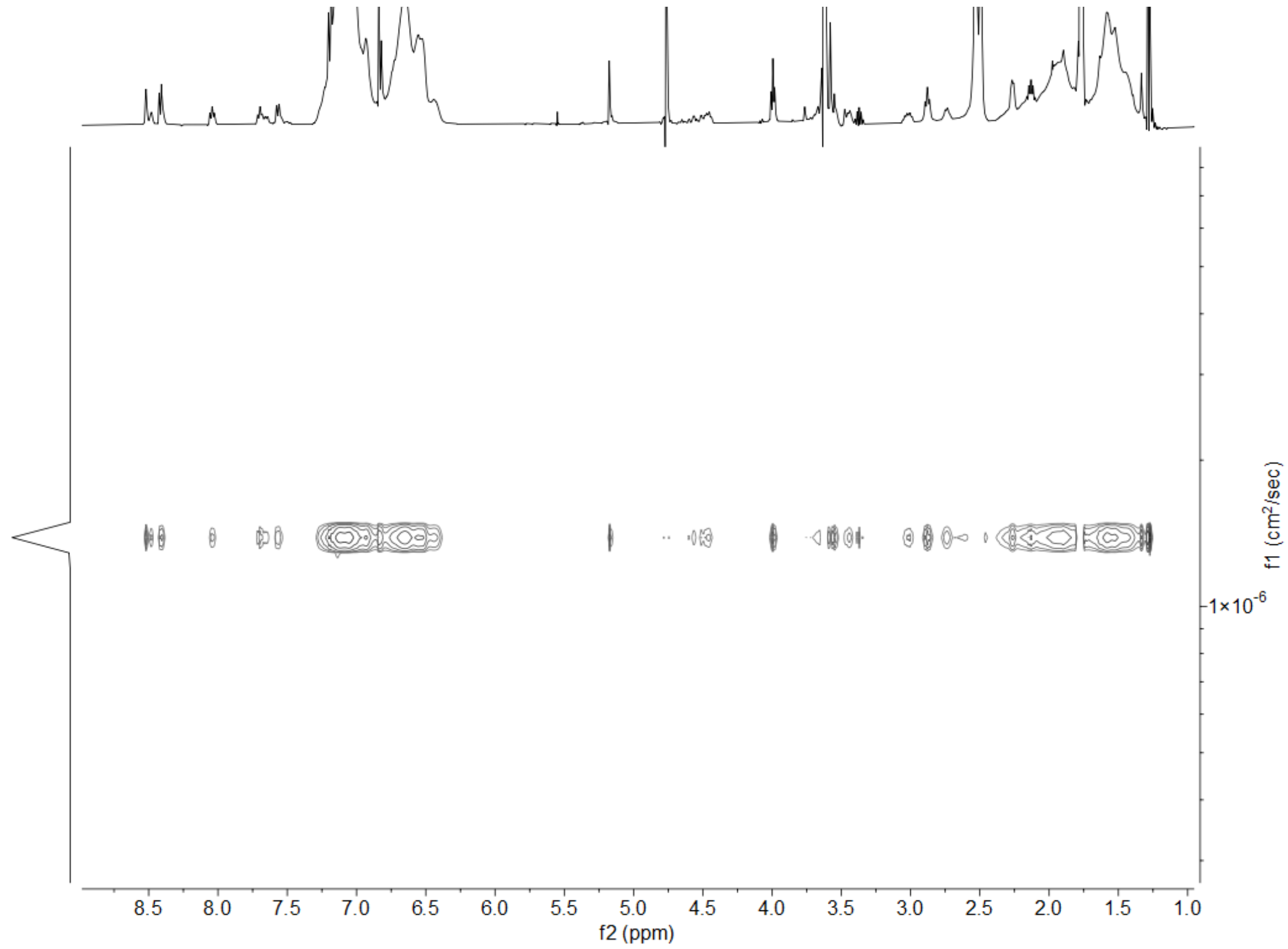

Figure 46. DOSY spectrum (500 MHz, THF-d_4, 298 K, gradient duration (δ) = 6.2 ms, diffusion time Δ = 45 ms) of **1**.

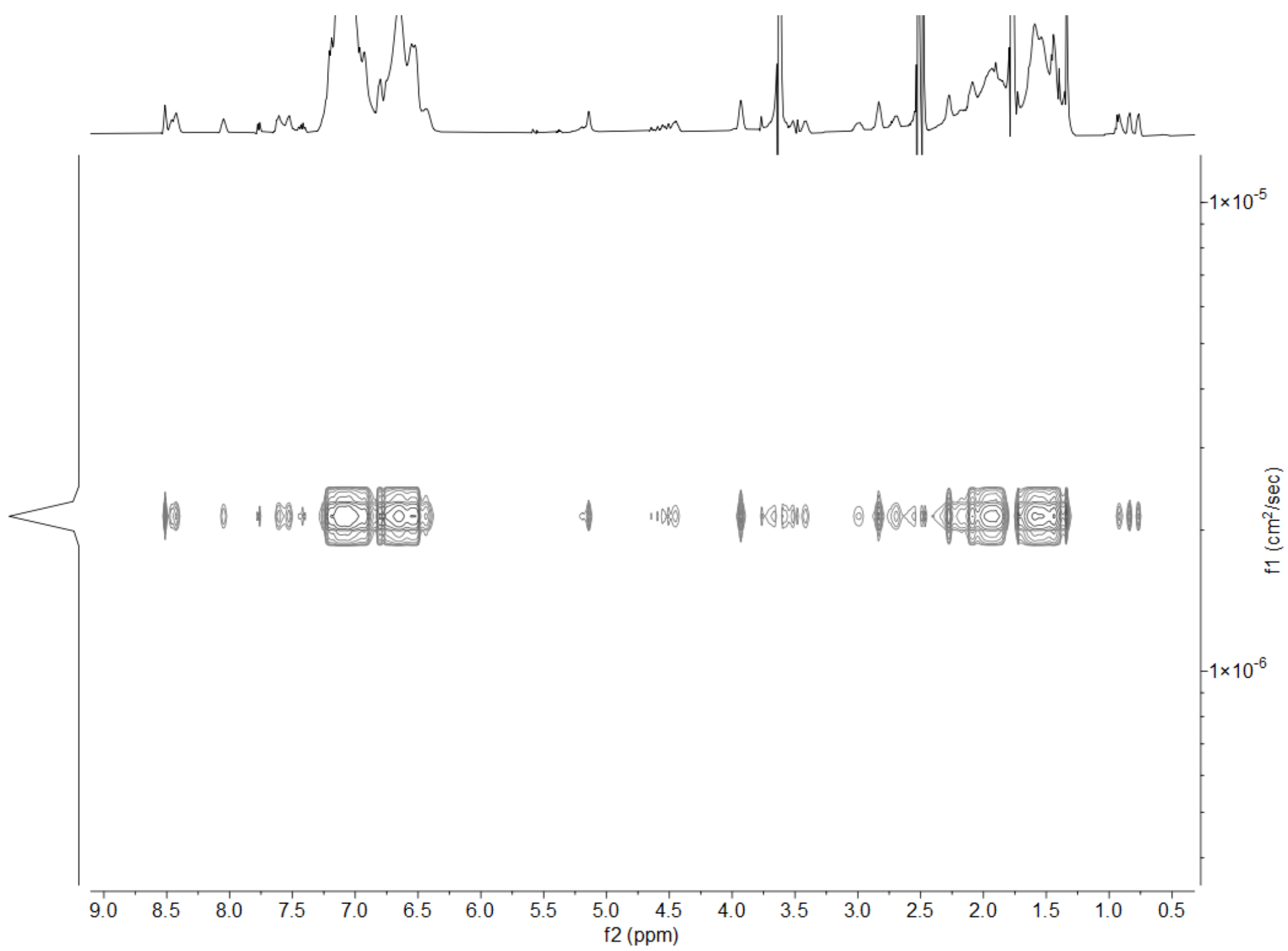

Figure 47. DOSY spectrum (500 MHz, THF-d_4, 298 K, gradient duration (δ) = 5 ms, diffusion time Δ = 45 ms) of **2**.

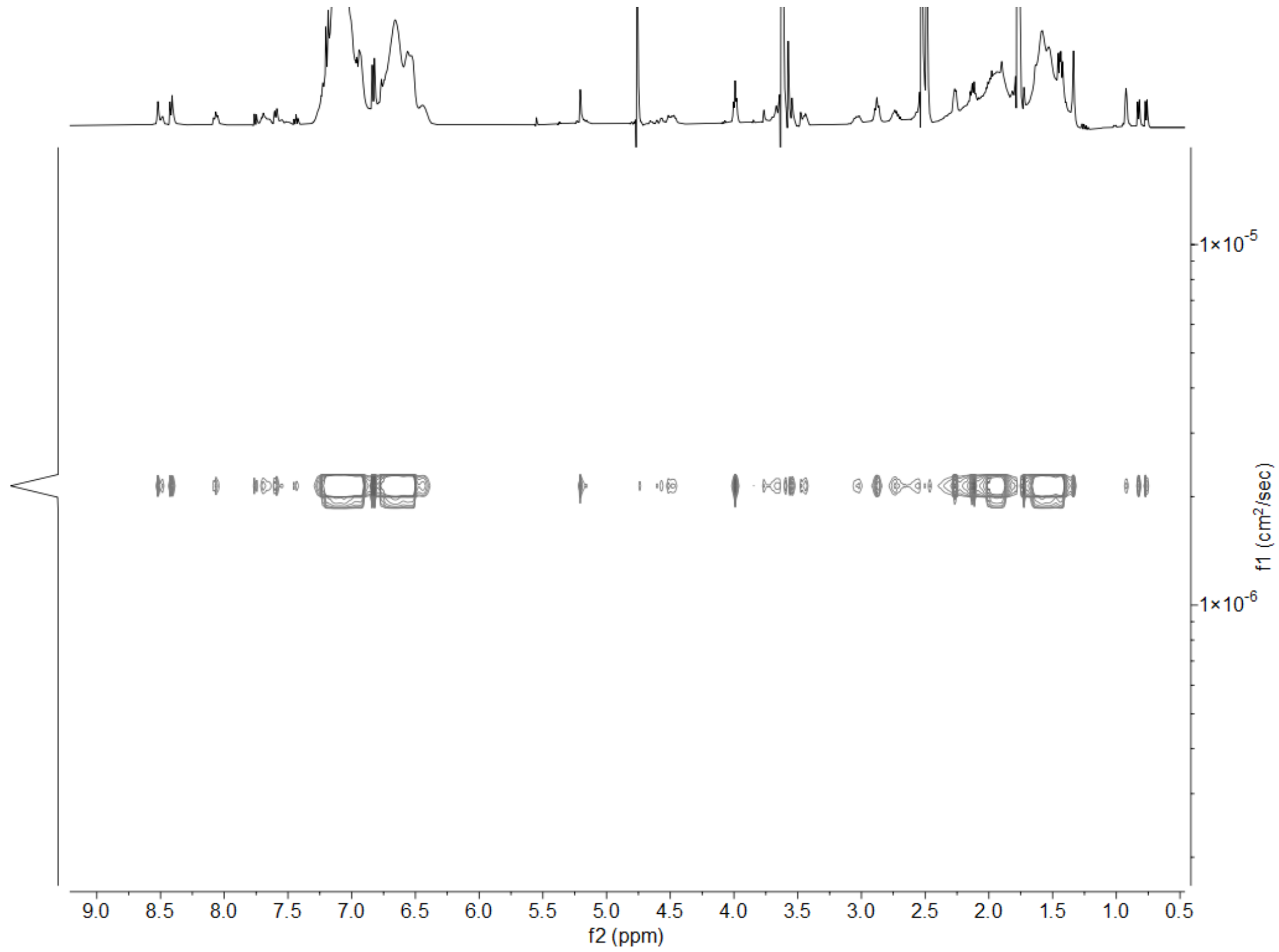

Figure 48. DOSY spectrum (500 MHz, THF-d_4, 298 K, gradient duration (δ) = 5 ms, diffusion time Δ = 45 ms) of **3**.

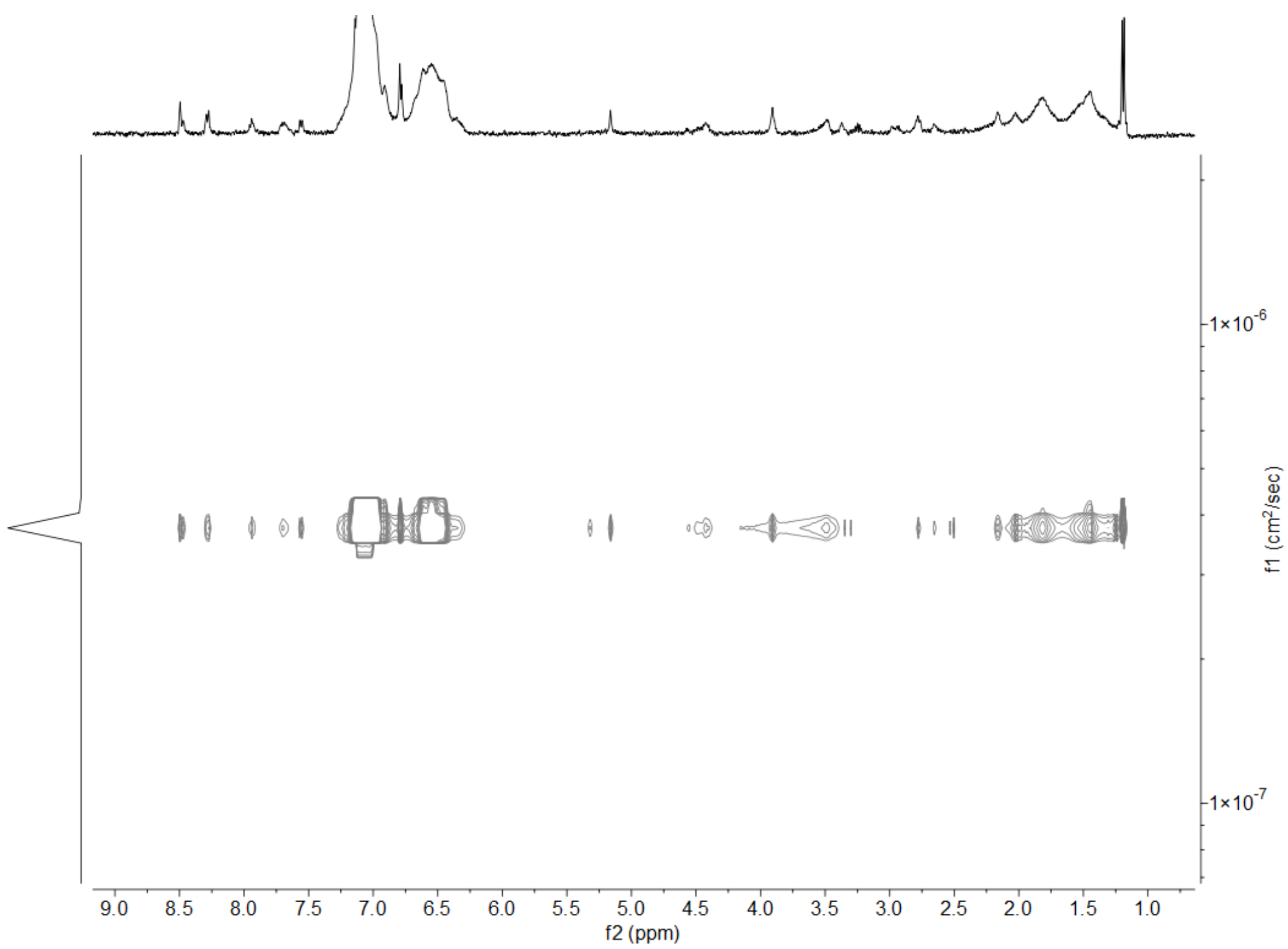

Figure 49. DOSY spectrum (500 MHz, DMSO-d_6, 298 K, gradient duration (δ) = 9 ms, diffusion time Δ = 55 ms) of **1**.

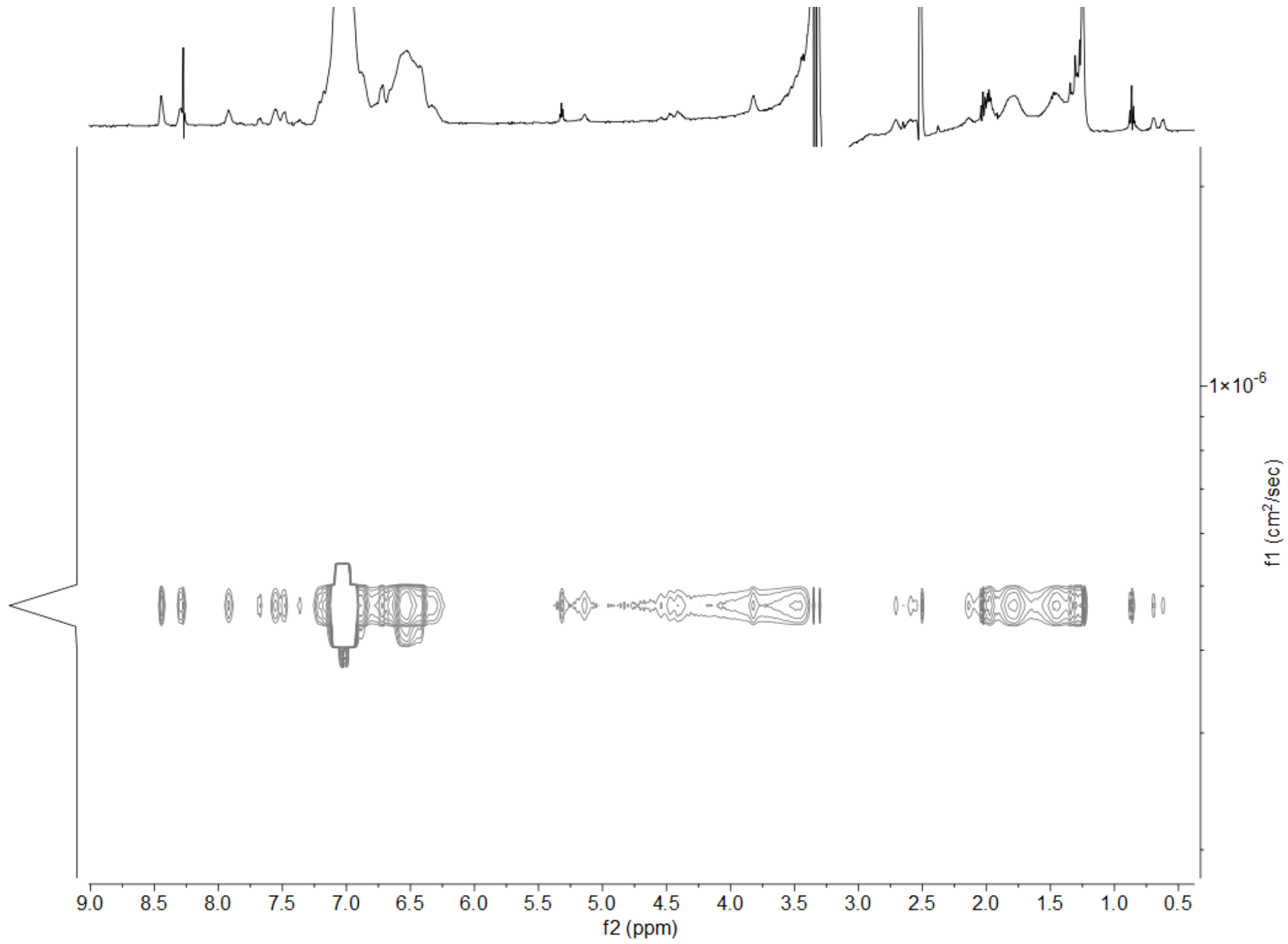

Figure 50. DOSY spectrum (500 MHz, DMSO-d_6, 298 K, gradient duration (δ) = 8.4 ms, diffusion time Δ = 55 ms) of **2**.

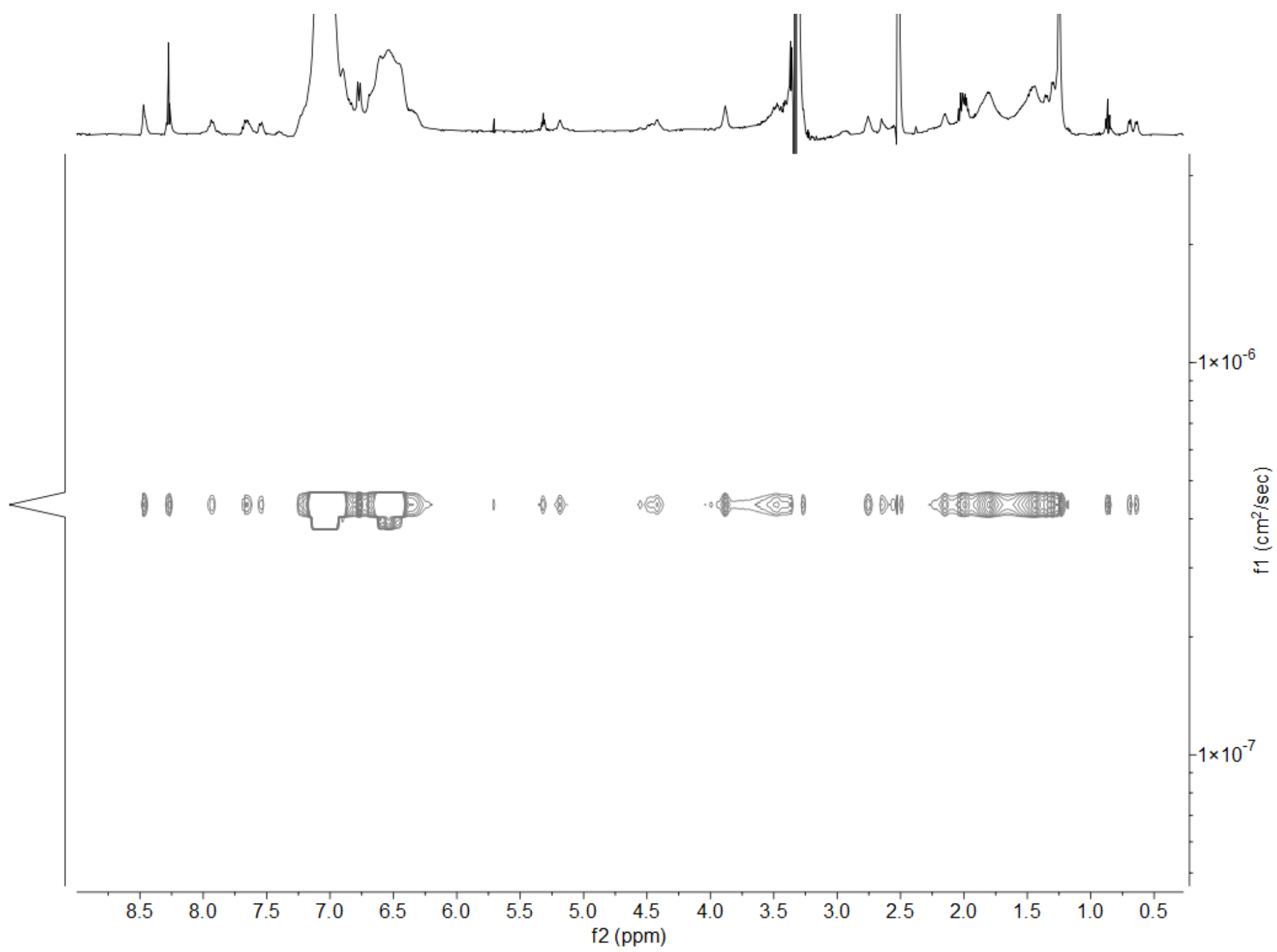

Figure 51. DOSY spectrum (500 MHz, DMSO-d_6, 298 K, gradient duration (δ) = 9 ms, diffusion time Δ = 55 ms) of **3**.

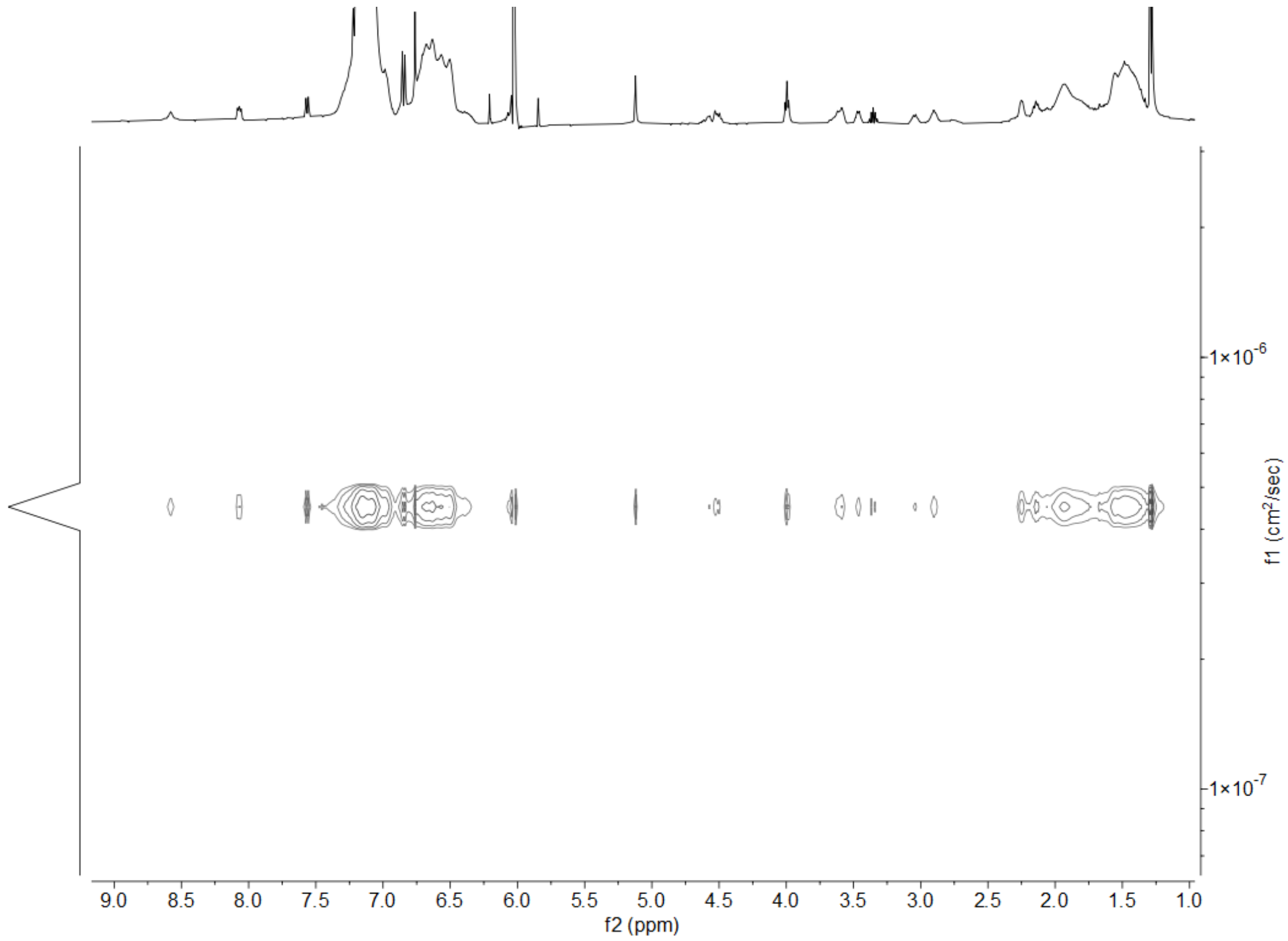

Figure 52. DOSY spectrum (500 MHz, TCE-d_2, 298 K, gradient duration (δ) = 9.6 ms, diffusion time Δ = 55 ms) of **1**.

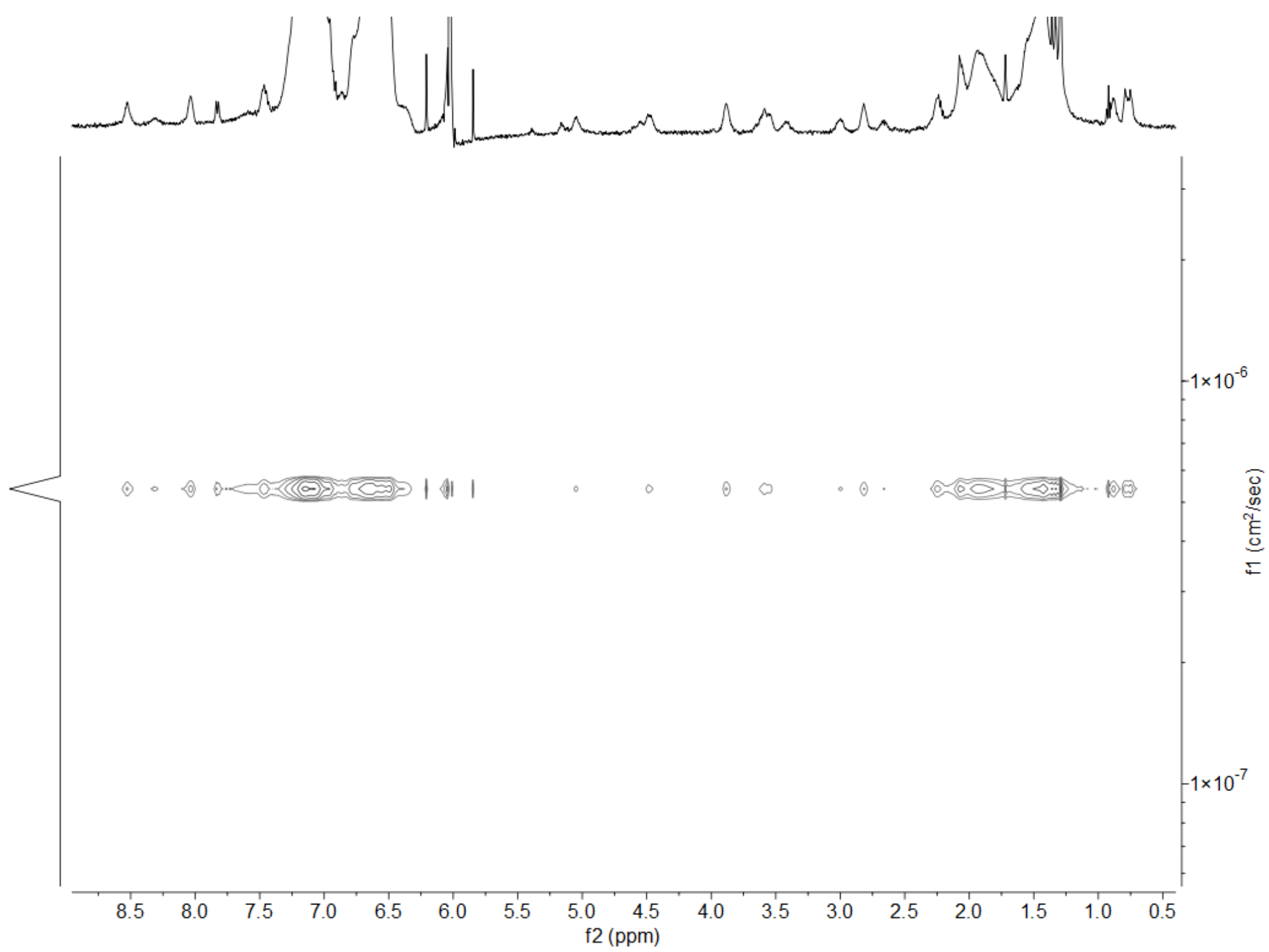

Figure 53. DOSY spectrum (500 MHz, TCE-d_2, 298 K, gradient duration (δ) = 8.4 ms, diffusion time Δ = 55 ms) of **2**.

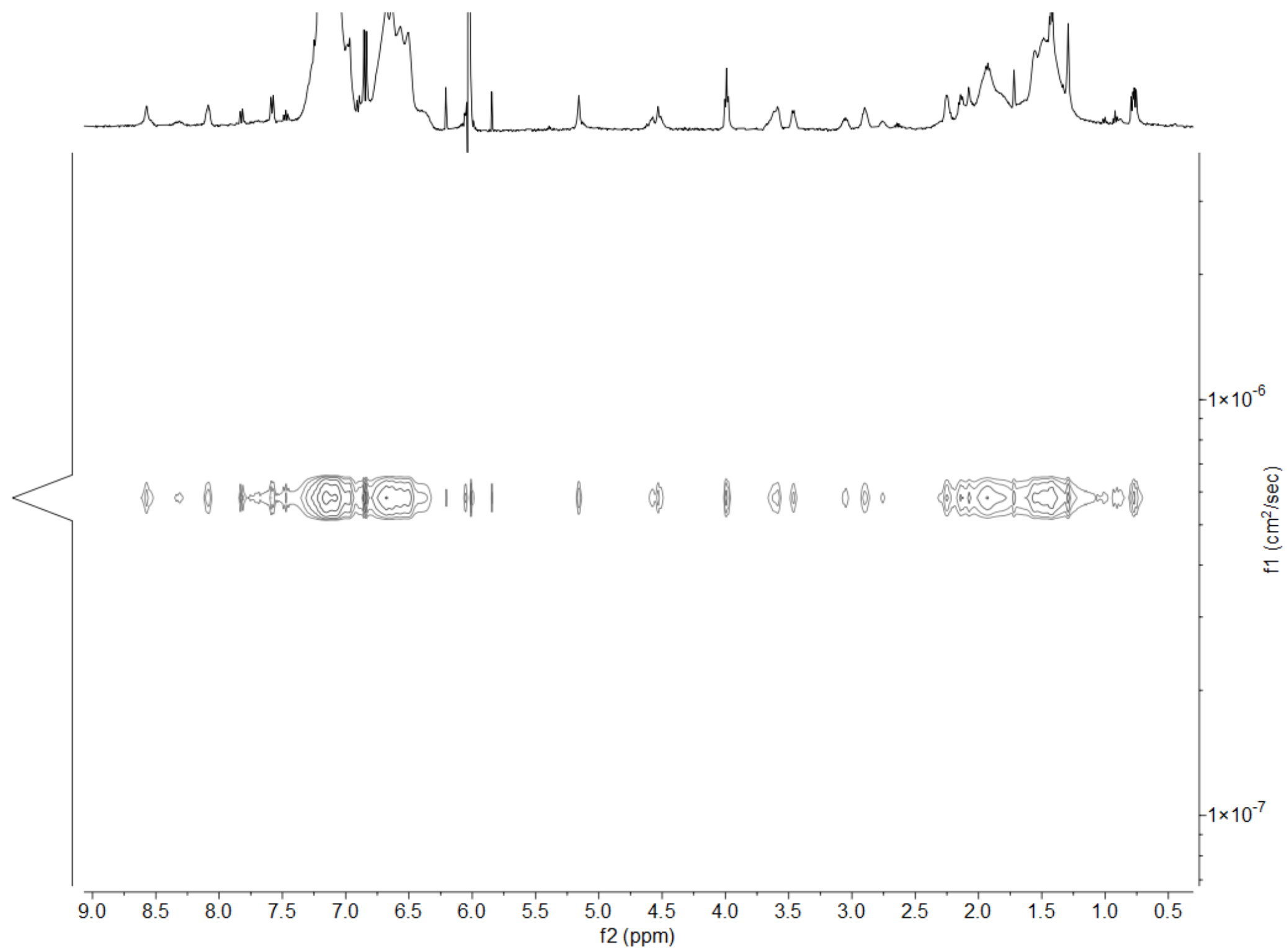

Figure 54. DOSY spectrum (500 MHz, TCE-d_2, 298 K, gradient duration (δ) = 8.4 ms, diffusion time Δ = 55 ms) of **3**.

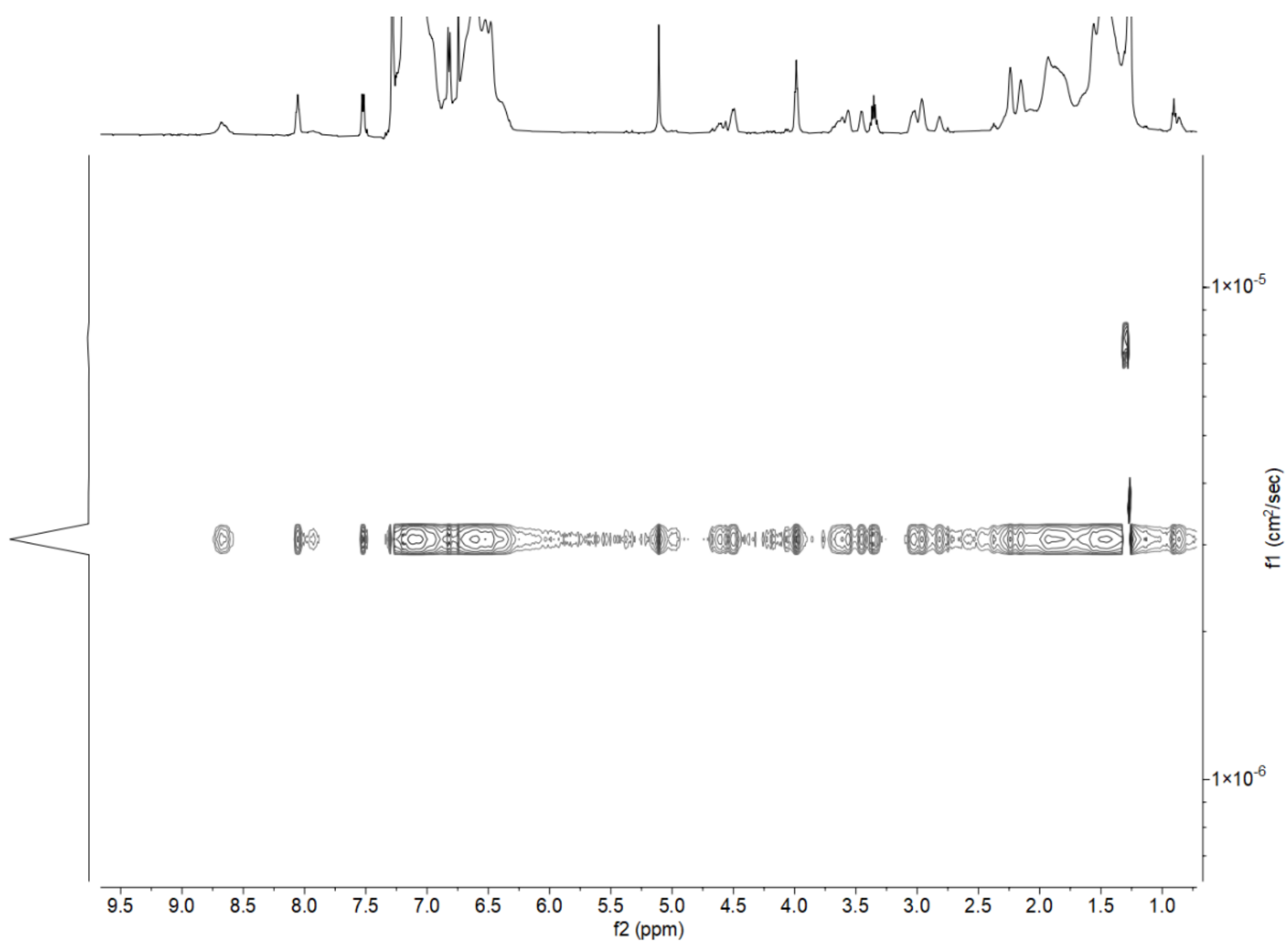

Figure 55. DOSY spectrum (500 MHz, $CDCl_3$, 298 K, gradient duration (δ) = 4.4 ms, diffusion time Δ = 60 ms) of **4**.

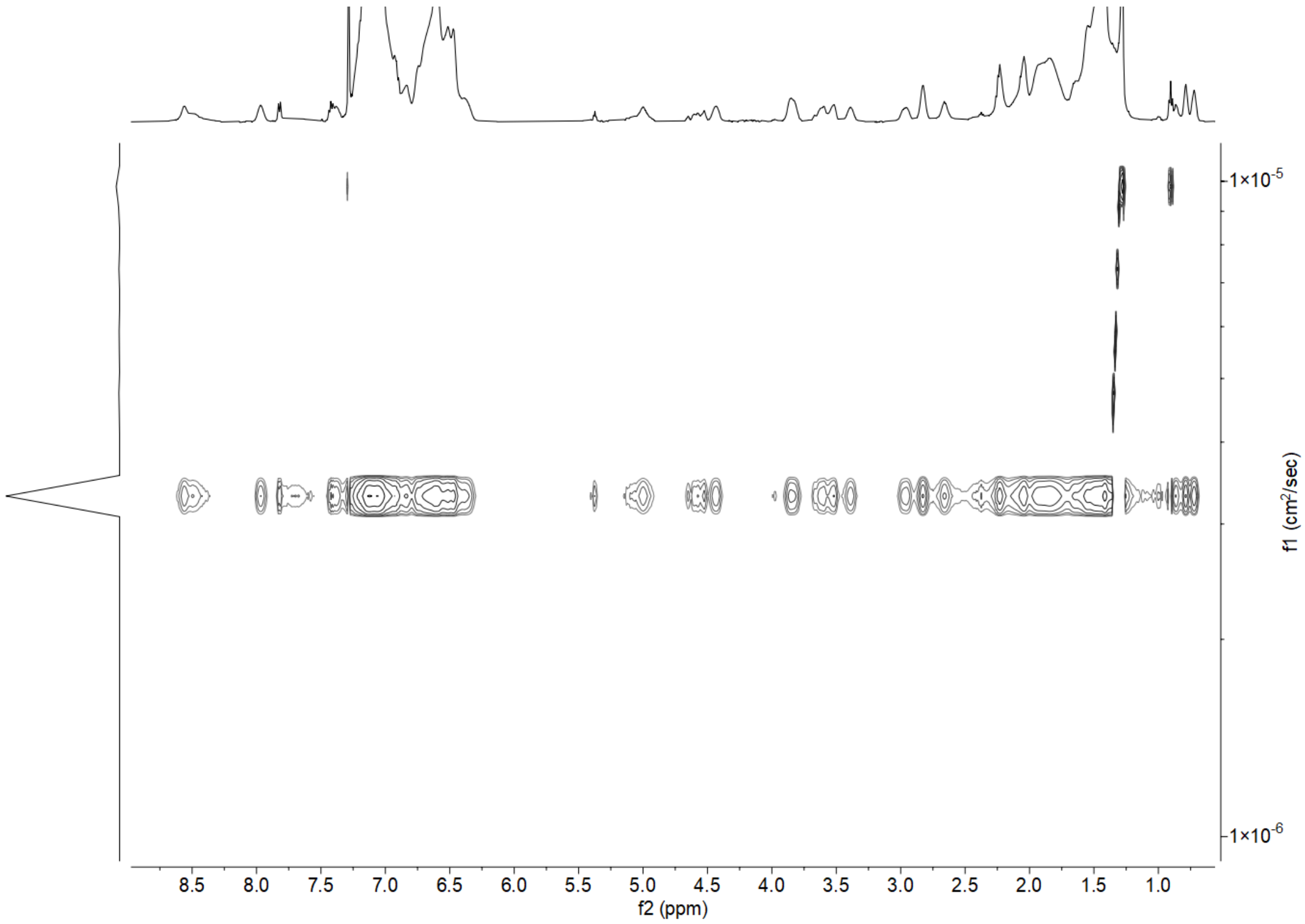

Figure 56. DOSY spectrum (500 MHz, $CDCl_3$, 298 K, gradient duration (δ) = 4 ms, diffusion time Δ = 60 ms) of **5**.

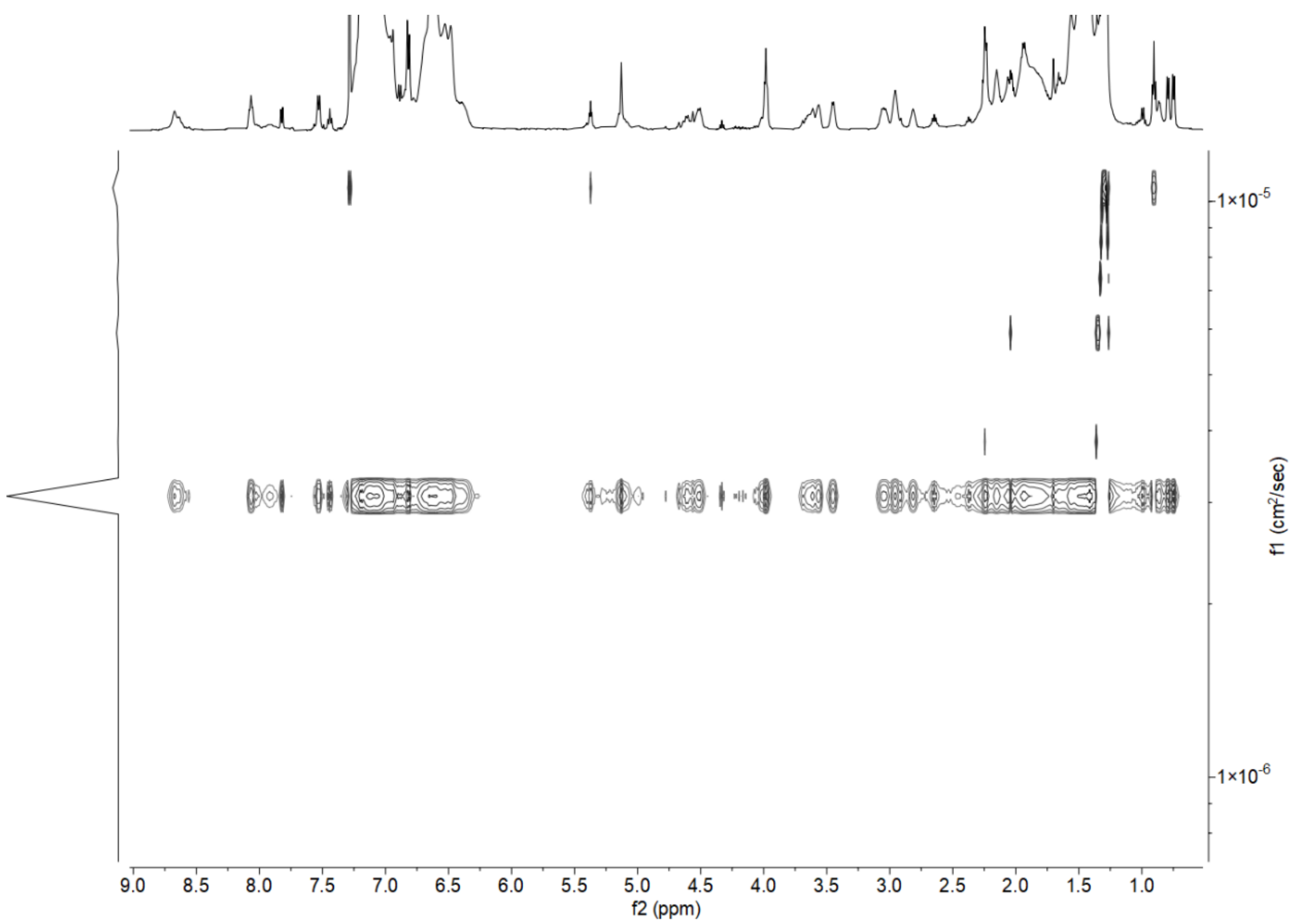

Figure 57. DOSY spectrum (500 MHz, $CDCl_3$, 298 K, gradient duration (δ) = 4.4 ms, diffusion time Δ = 60 ms) of **6**.

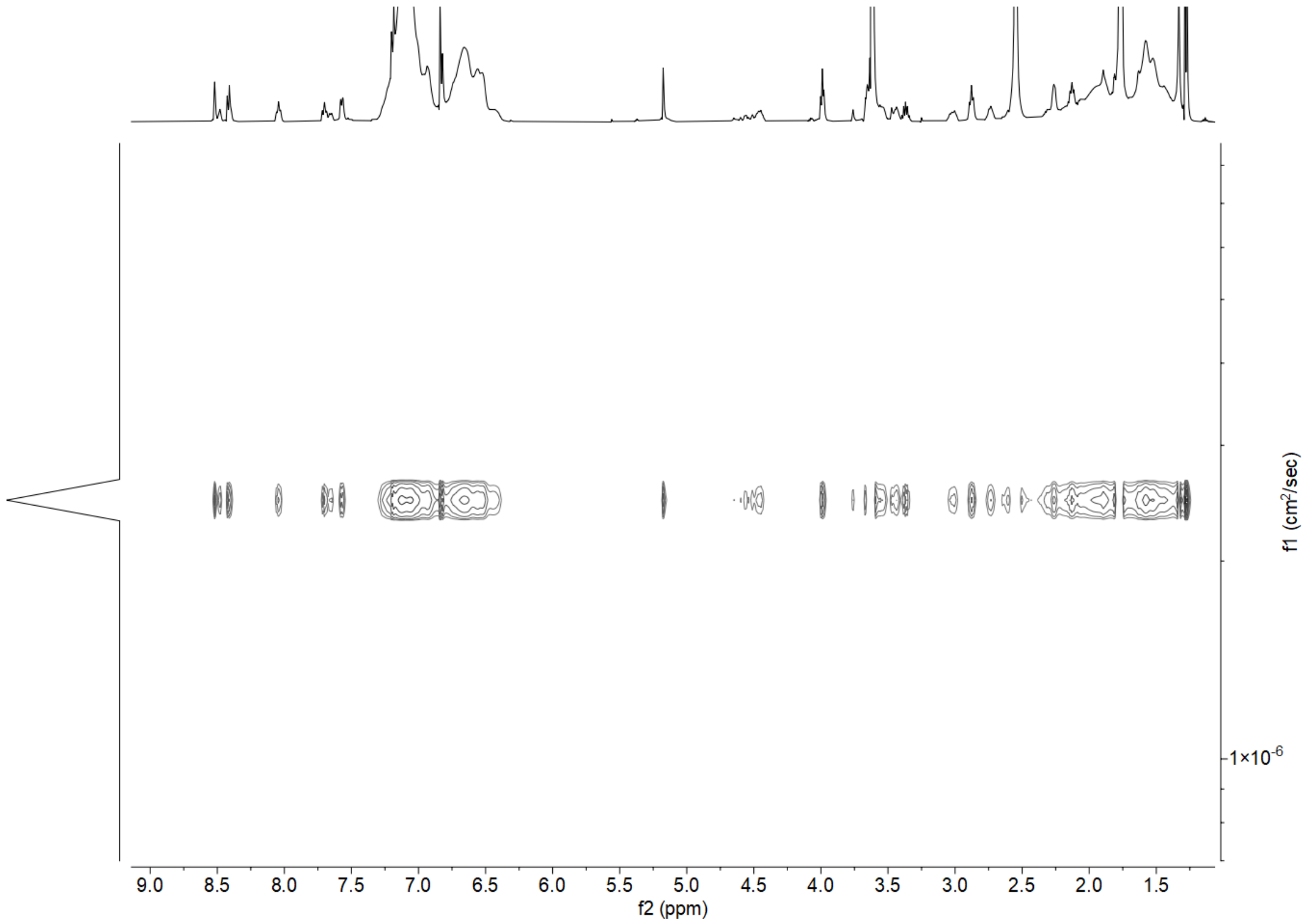

Figure 58. DOSY spectrum (500 MHz, THF-d_4, 298 K, gradient duration (δ) = 4.6 ms, diffusion time Δ = 60 ms) of **4**.

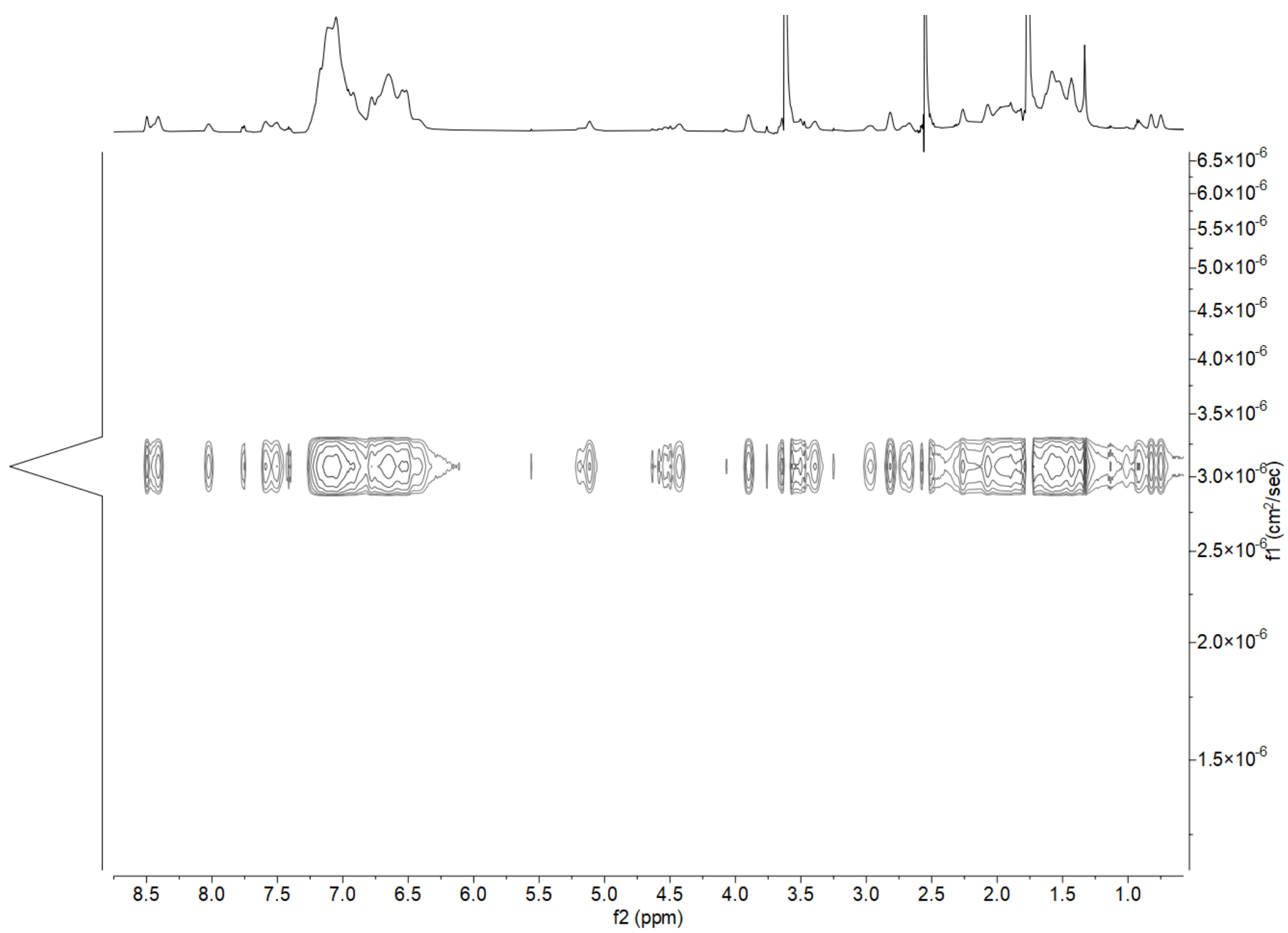

Figure 59. DOSY spectrum (500 MHz, THF-d_4, 298 K, gradient duration (δ) = 4 ms, diffusion time Δ = 60 ms) of **5**.

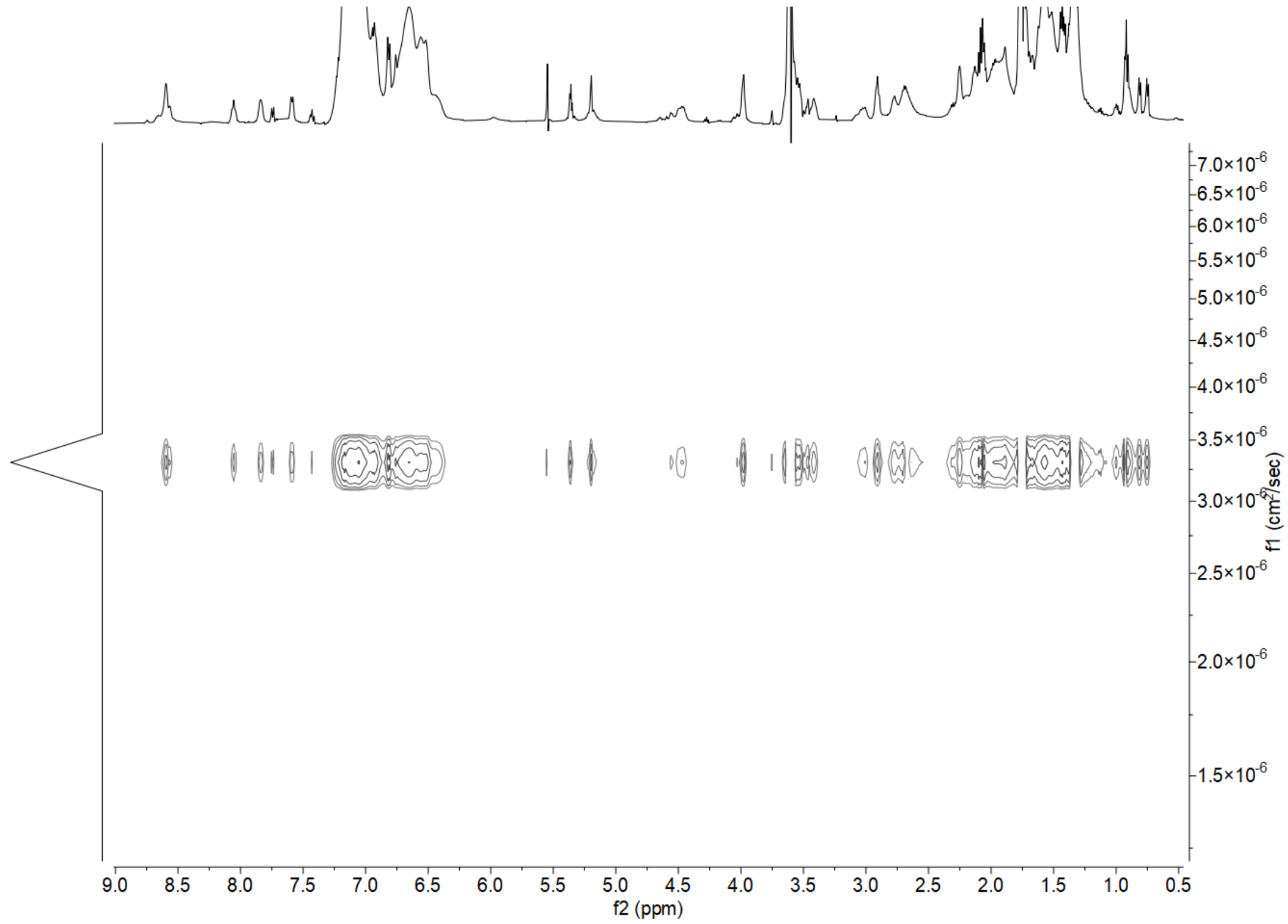

Figure 60. DOSY spectrum (500 MHz, THF-d_4, 298 K, gradient duration (δ) = 4 ms, diffusion time Δ = 60 ms) of **6**.

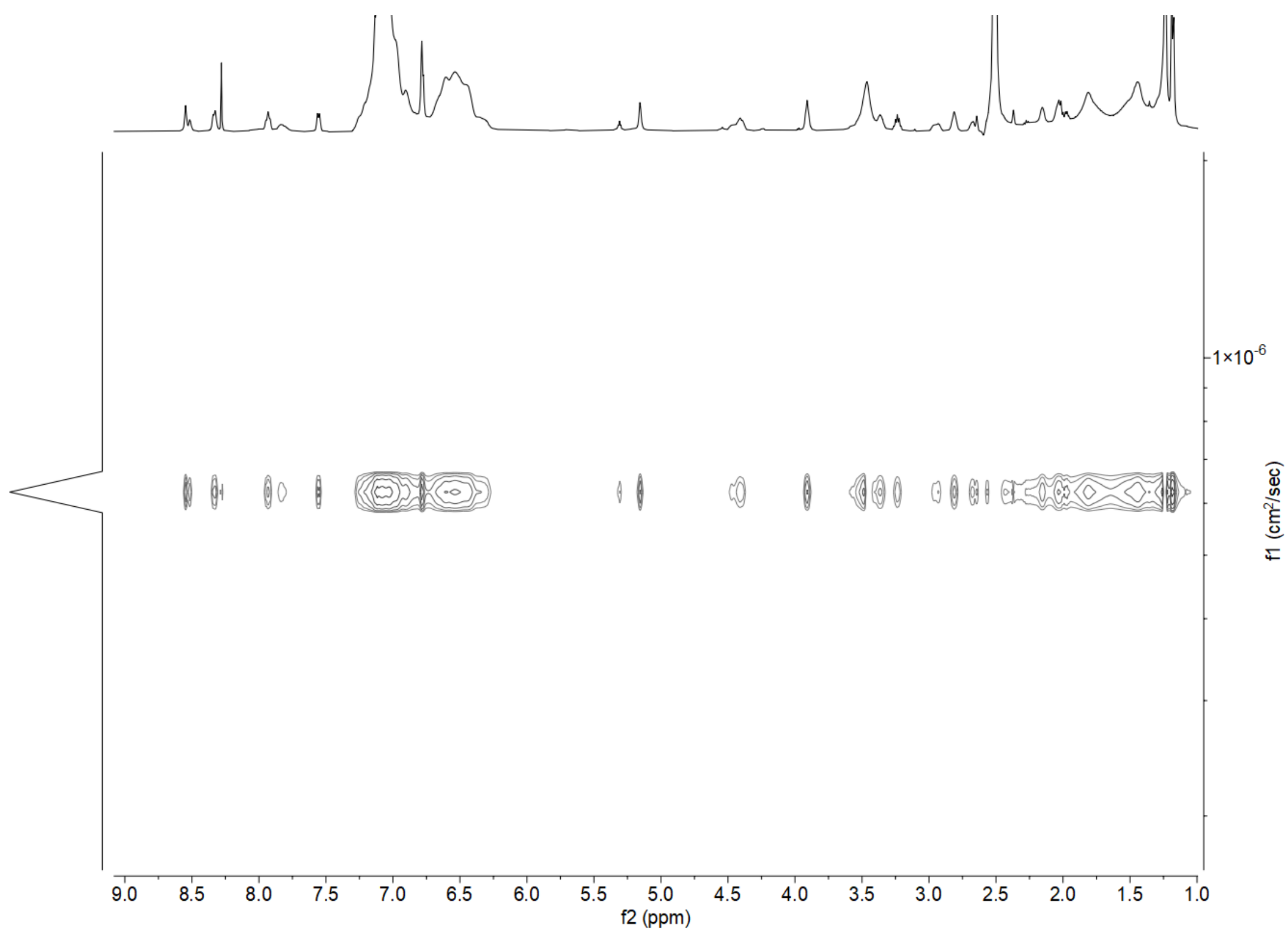

Figure 61. DOSY spectrum (500 MHz, DMSO-d_6, 298 K, gradient duration (δ) = 8 ms, diffusion time Δ = 60 ms) of **4**.

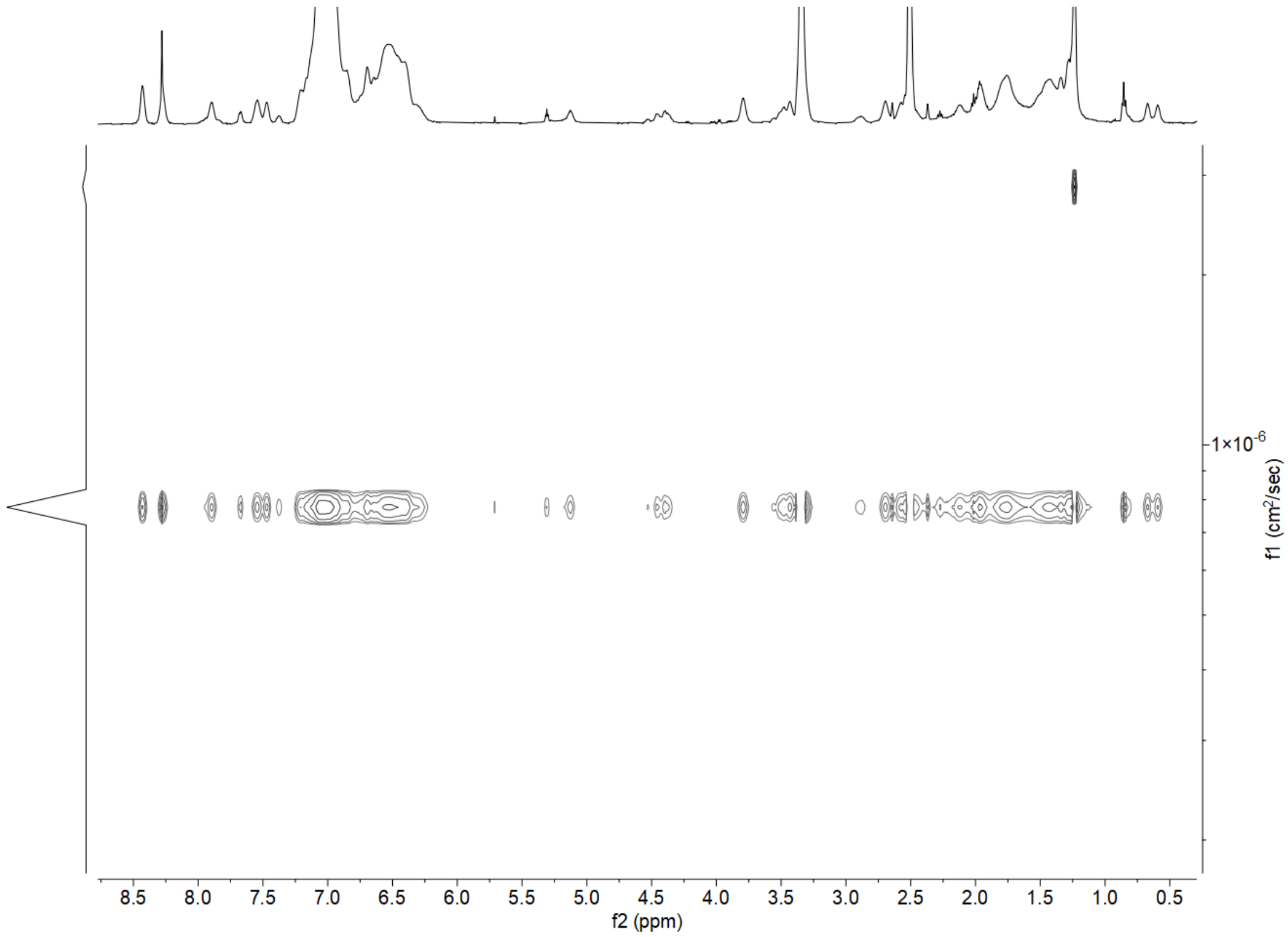

Figure 62. DOSY spectrum (500 MHz, DMSO-d_6, 298 K, gradient duration (δ) = 8 ms, diffusion time Δ = 60 ms) of **5**.

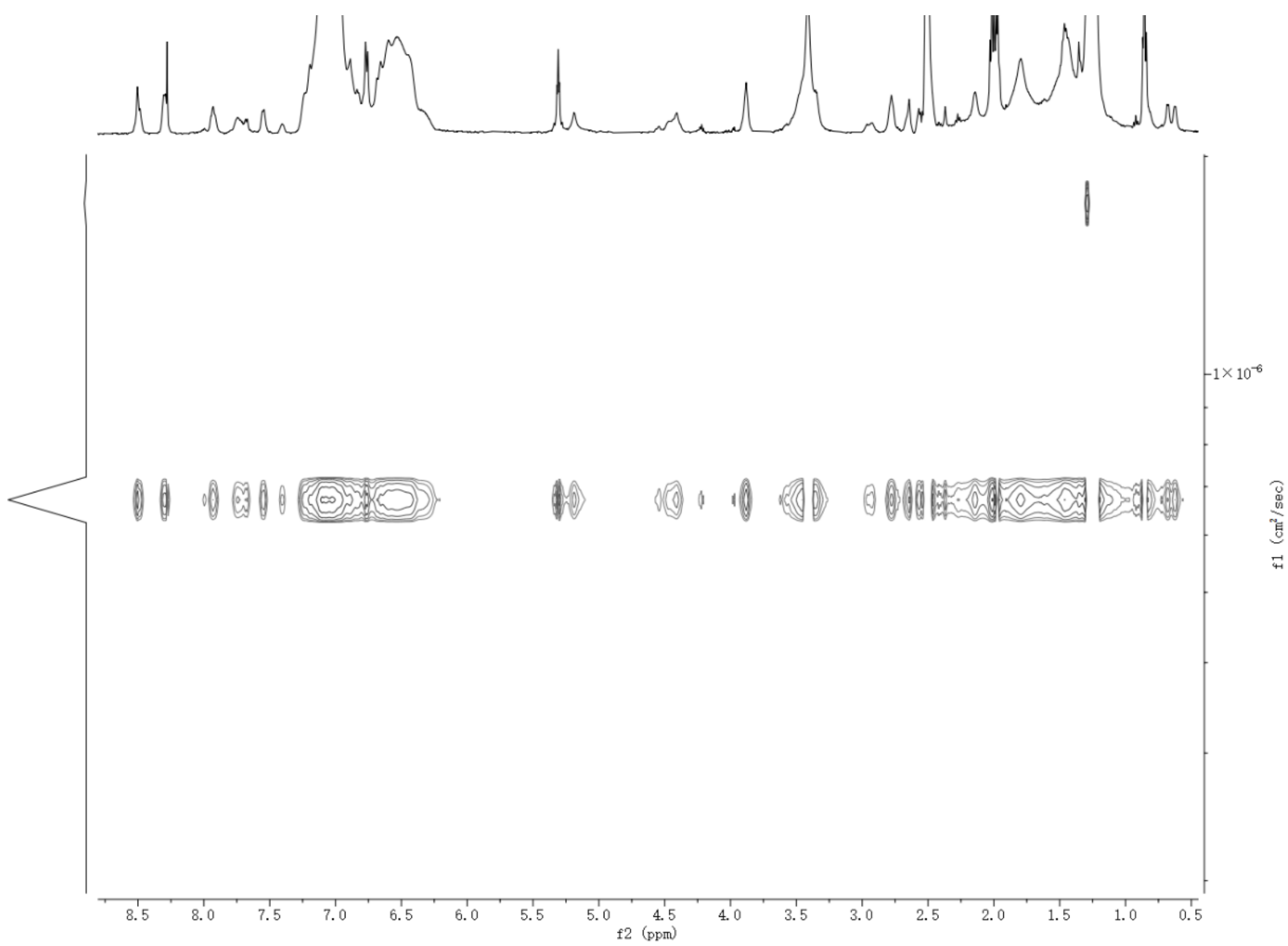

Figure 63. DOSY spectrum (500 MHz, DMSO-d_6, 298 K, gradient duration (δ) = 6.6 ms, diffusion time Δ = 80 ms) of **6**.

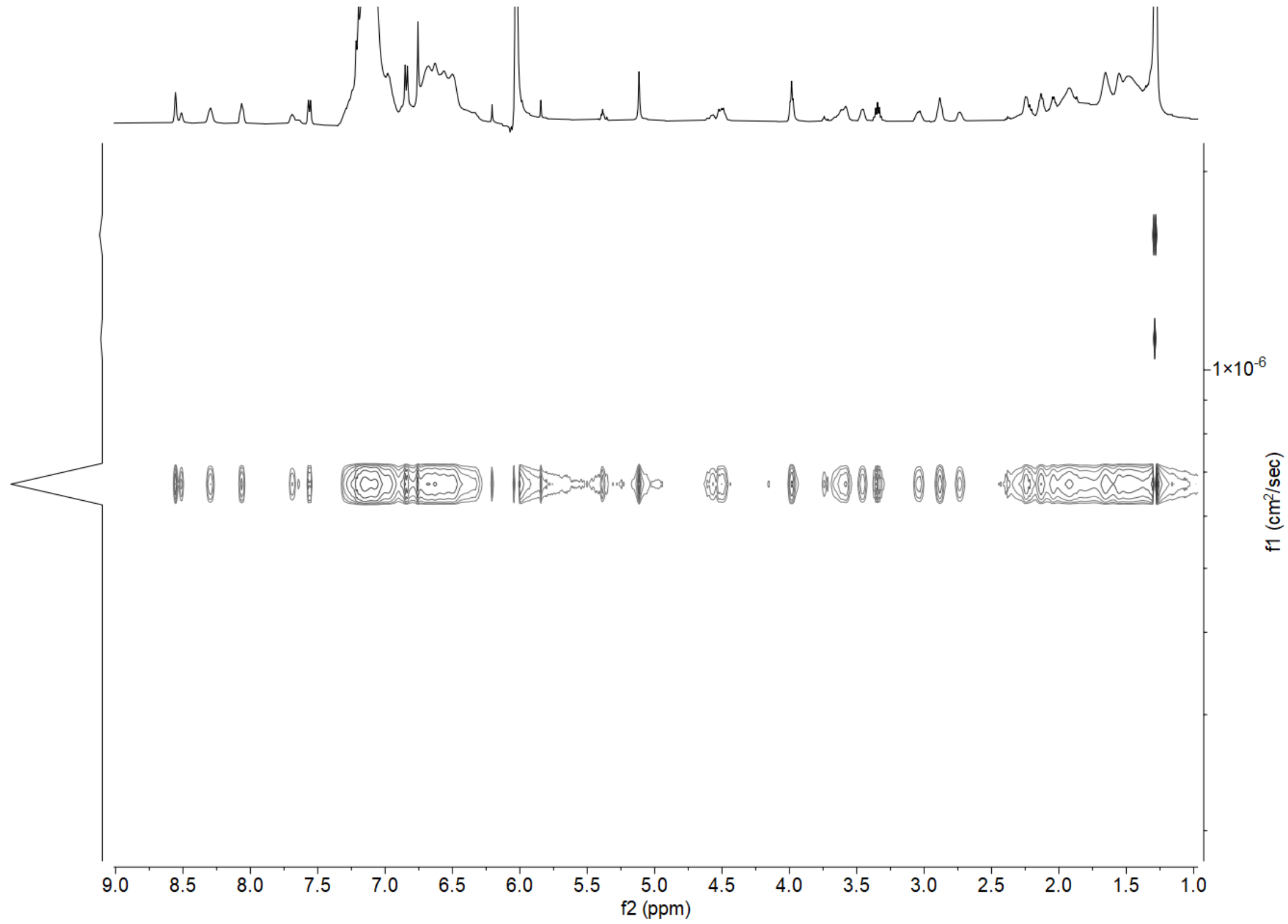

Figure 64. DOSY spectrum (500 MHz, TCE-d_2, 298 K, gradient duration (δ) = 8 ms, diffusion time Δ = 60 ms) of **4**.

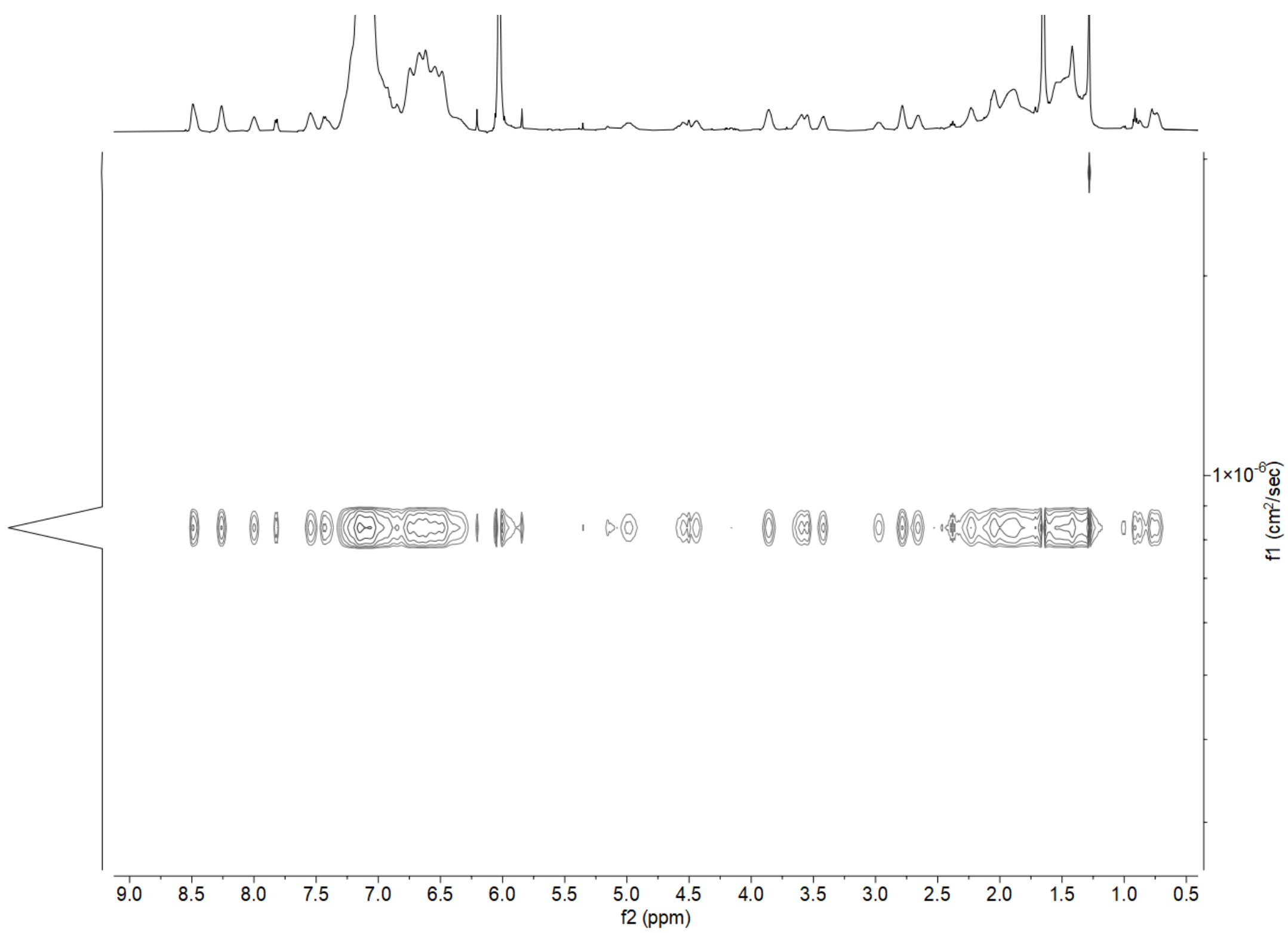

Figure 65. DOSY spectrum (500 MHz, TCE-d_2, 298 K, gradient duration (δ) = 7.6 ms, diffusion time Δ = 60 ms) of **5**.

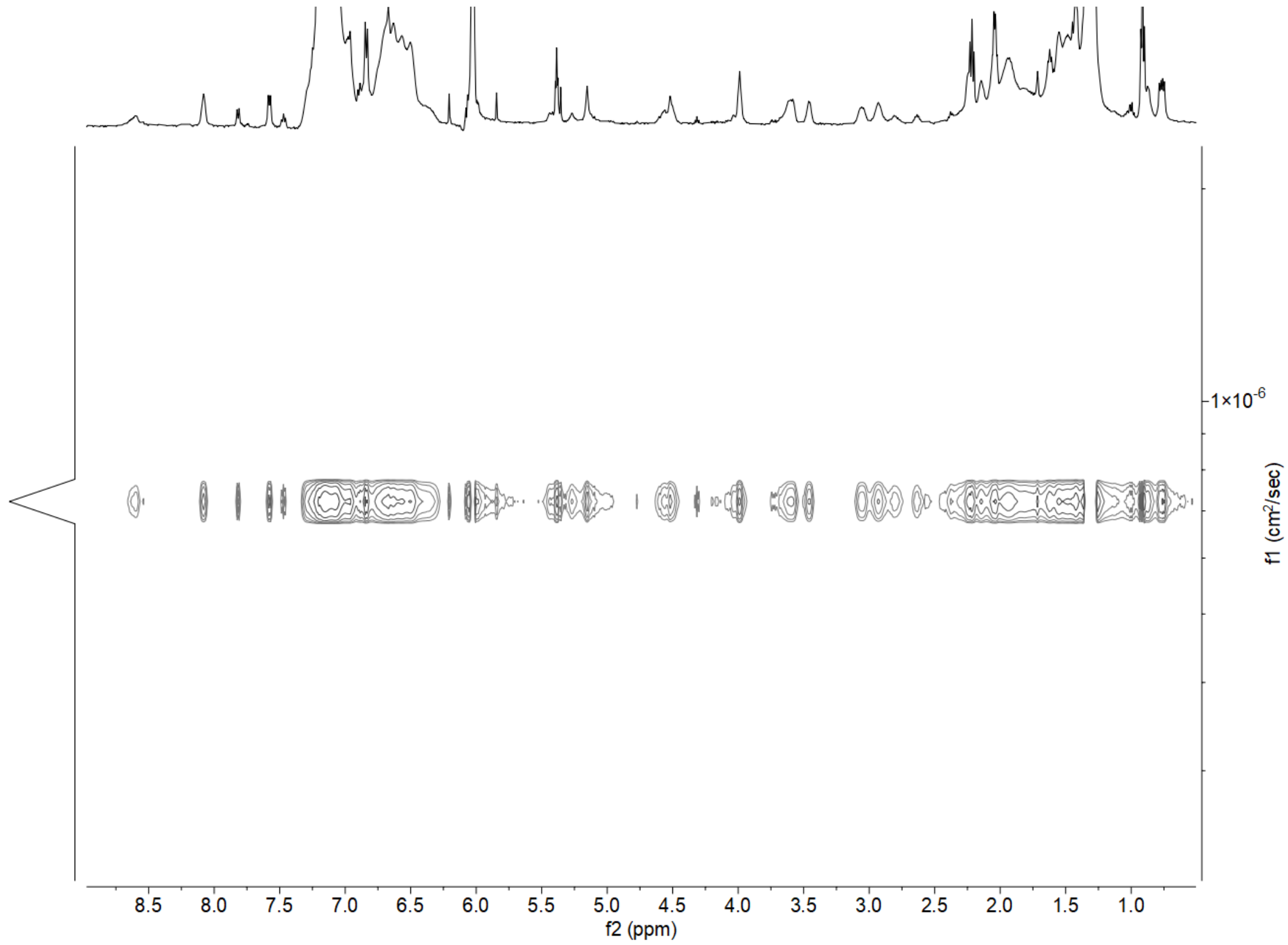

Figure 66. DOSY spectrum (500 MHz, TCE-d_2, 298 K, gradient duration (δ) = 7 ms, diffusion time Δ = 80 ms) of **6**.

9. AFM iamges of linear (1), trfoiled (2) and cyclic (3) polymers

Atomic force microscopy (AFM) images were obtained on a Bruker Dimension® Icon™ instrument under tipping mode in air. The samples were prepared by drop casting the chloroform solution of the samples (1.0×10^{-8} mol/L) onto freshly cleaved mica and then air-dry.

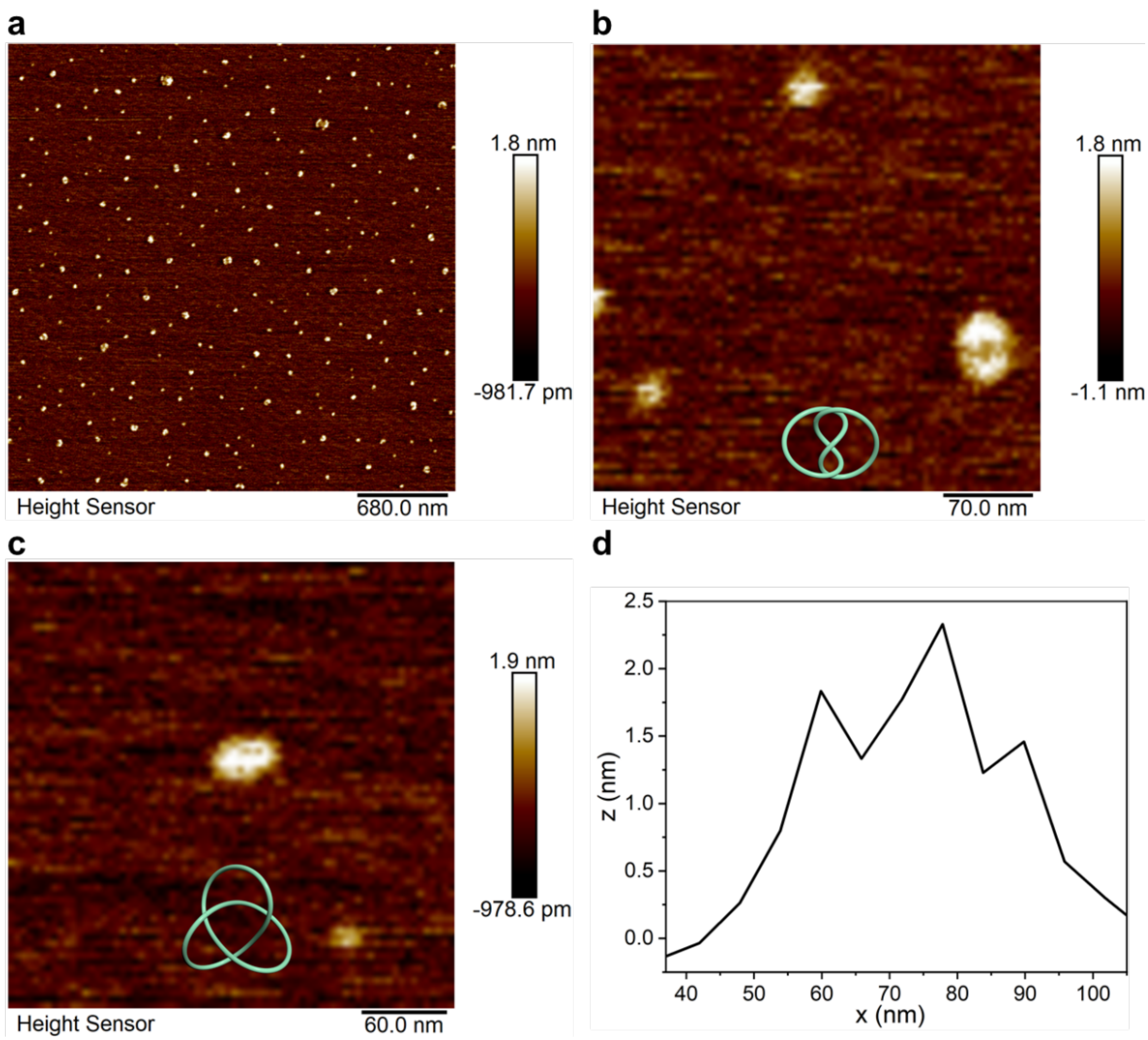

Figure 67. (a, b, c) AFM images and corresponding; (d) height profile (collected from c) of trefoiled polymer **2**.

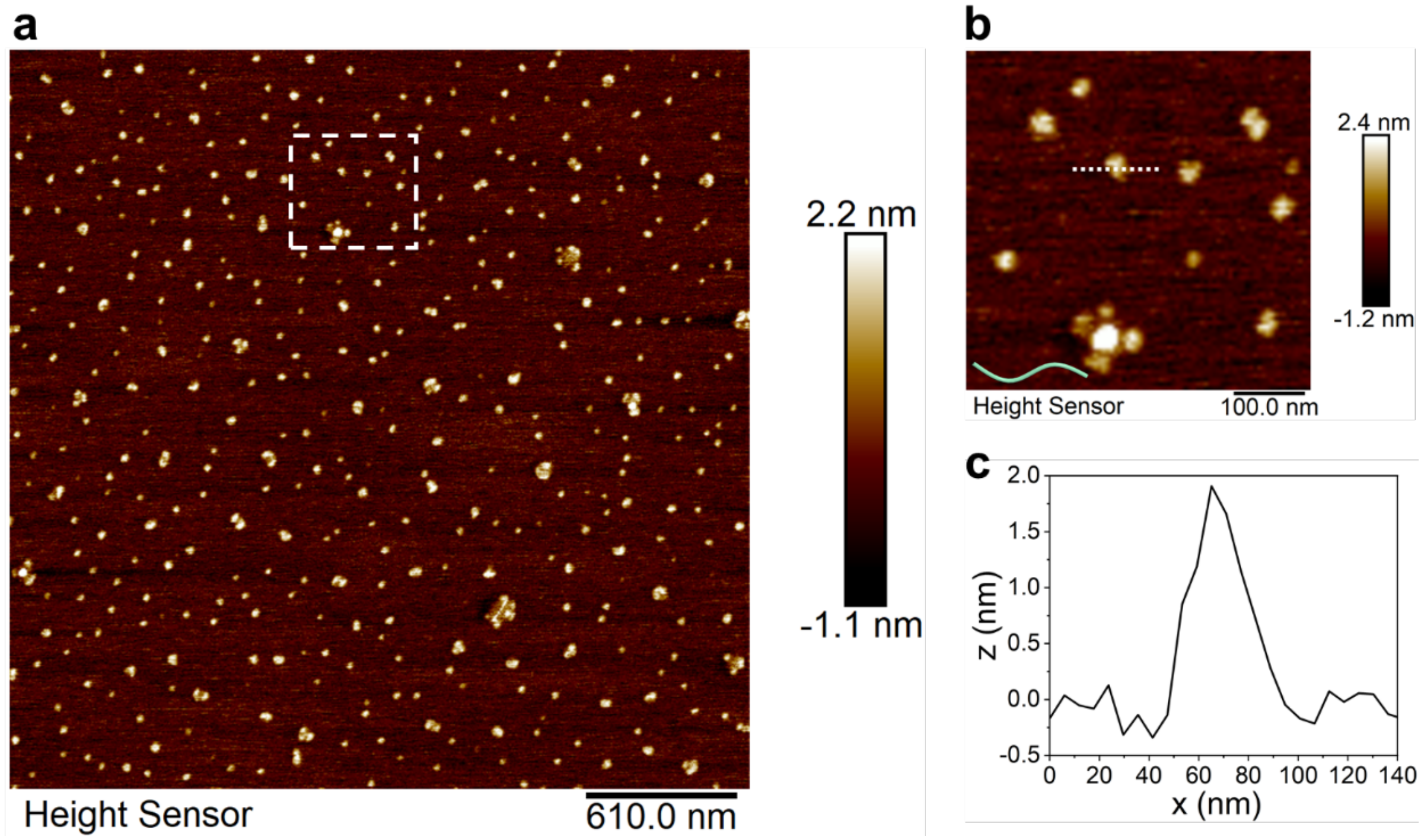

Figure 68. (a, b) AFM images and corresponding; (c) height profile of linear polymer **1.**

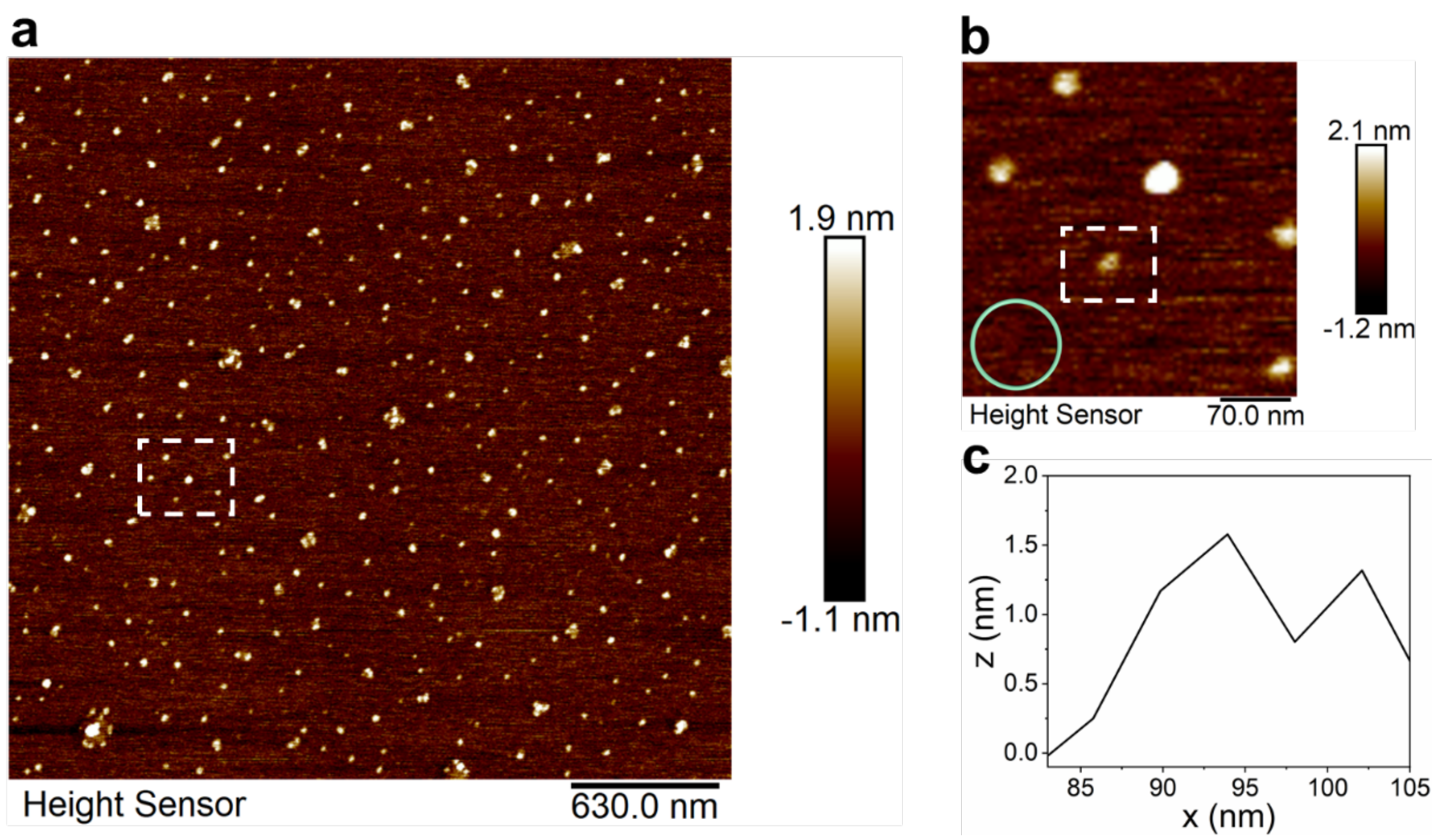

Figure 69. (a, b) AFM images and corresponding; (c) height profile of macrocycle **3**.

10. STM iamges of 1, 2, 3, 5 and [Fe2](PF_6)$_2$

Ultra-High-Vacuum Low-Temperature Scanning Tunneling Microscopy (UHV-LT-STM) images were conducted on a CreaTec Fischer & Co. GmbH LT-STM-AFM-BO scanning tunneling microscope at 77 K. The Ag(111) substrate was cleaned by repeated cycles of Ar^+ sputtering (1.5 kV) followed by annealing at 700 K. An electrochemically etched polycrystalline tungsten wire was used for the STM tip. **1**, **2**, **3**, **5**, [Fe**2**](PF_6)$_2$ were dissolved in DCM at a concentration of 5.0 × 10^{-7} M. **2** was also dissolved in the mixture of DCM and DMF (v: v = 10: 1) at a concentration of 5.0 × 10^{-7} M to research the influence of polarity on the morphology of knot. Onto the freshly annealed Ag(111) surface, 0.02 mL of the solution was deposited by drop casting. The obtained STM images were analyzed by WSxM5.0 software.

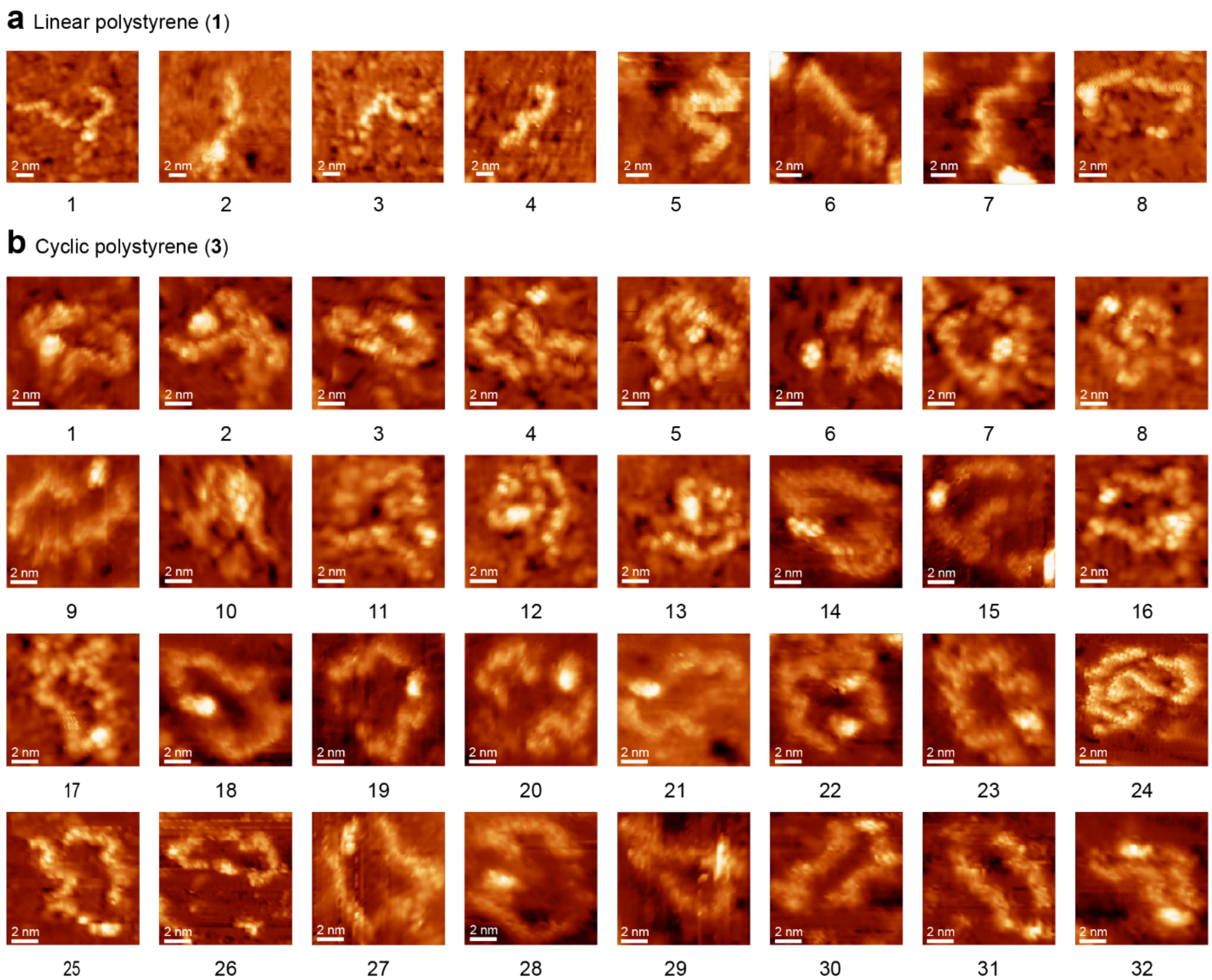

Figure 70. Single molecular STM images of (a) linear polystyrene **1** and (b) cyclic polystyrene **3** with DCM as solvent for preparing the samples. A bright lobe can be observed which is attributed to DIBOD moiety (Scanning parameters: U = 2.7 V, I_t = 20 pA).

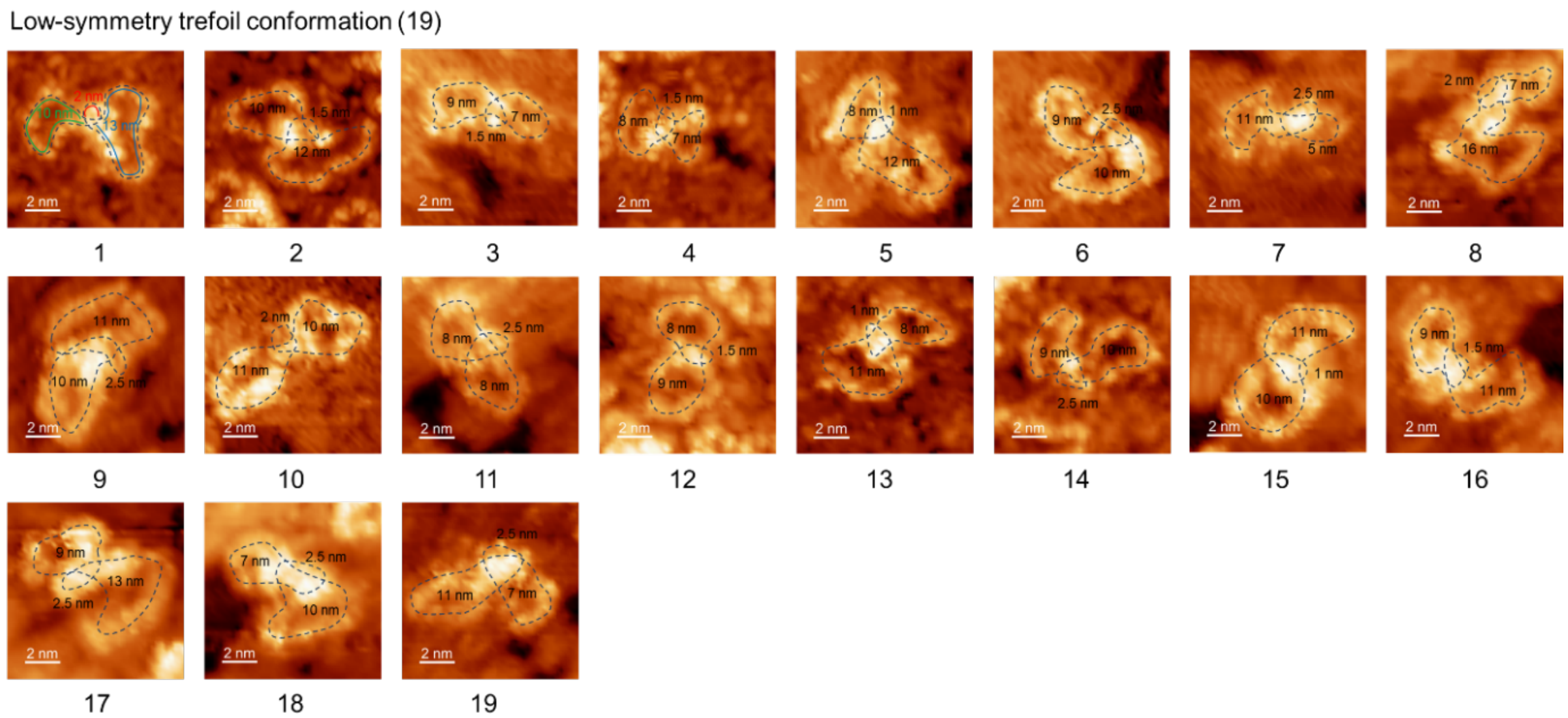

Figure 71. Single molecular STM images of low-symmetry trefoil **2** with DCM as solvent for preparing the samples. A black dashed line superimposed on each molecule outlines the skeletal structure of the low-symmetry trefoil, with the corresponding loop lengths indicated. The loop lengths are labelled in the loop. After analysing 69 individual images, it was found that 19 (28%) exhibited low-symmetry conformation (Scanning parameters: $U = 2.7$ V, $I_t = 20$ pA). If the contour length of the shortest loop is less than 2.8 nm, we assign the structure as a low-symmetry trefoil. In contrast, when all three loops of a three-loop knot exceed this threshold length, the conformation is accordingly classified as a high-symmetry trefoil. Furthermore, if a molecule exhibits a two-loop state with a shared elongated interior region, it is considered a medium-symmetry trefoil.

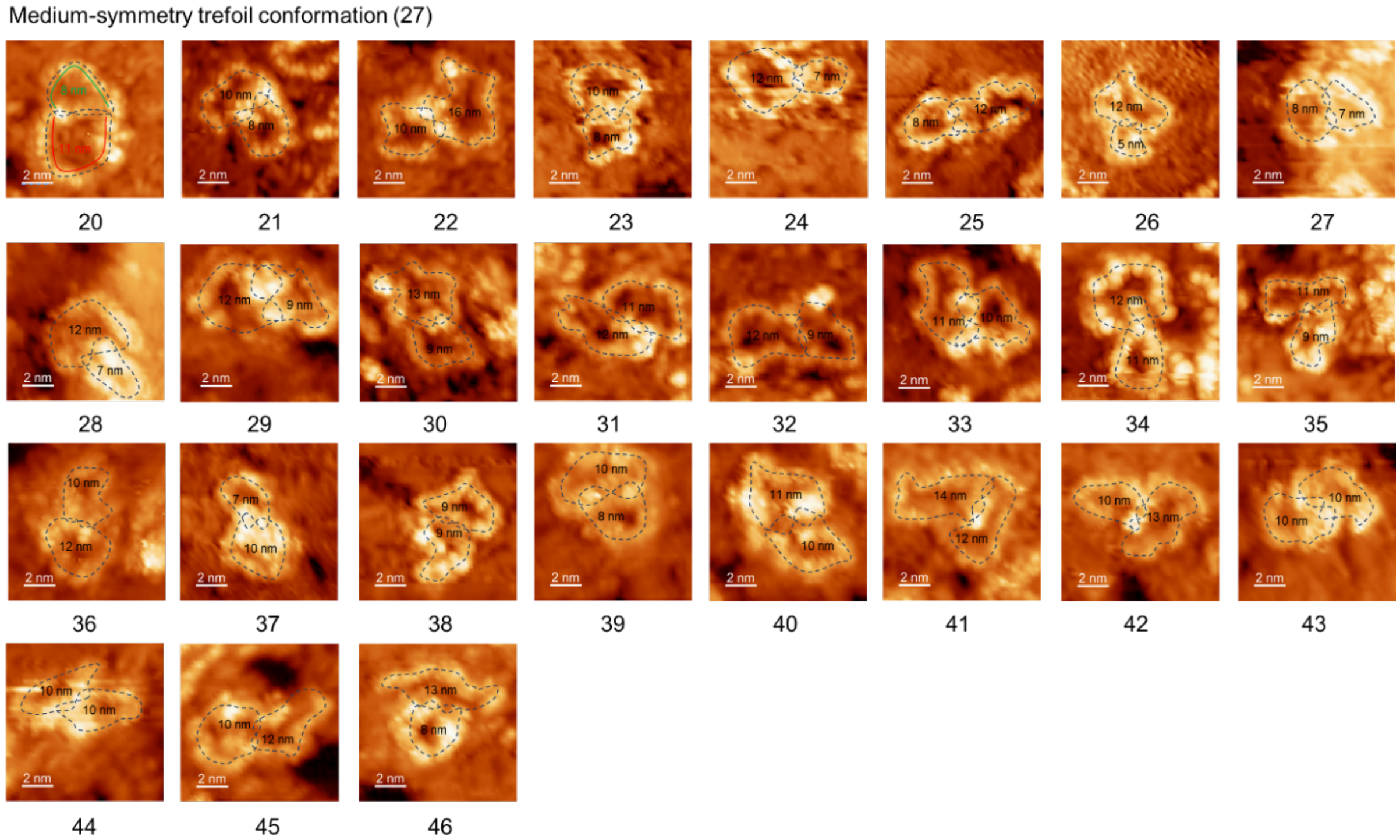

Figure 72. Single molecular STM images of medium-symmetry trefoil **2** with DCM as solvent for preparing the samples. A black dashed line superimposed on each molecule outlines the skeletal structure of the medium-symmetry trefoil, with the corresponding loop lengths indicated. After analysing 69 individual images, it was found that 27 (39%) exhibited medium-symmetry conformation (Scanning parameters: $U = 2.7$ V, $I_t = 20$ pA).

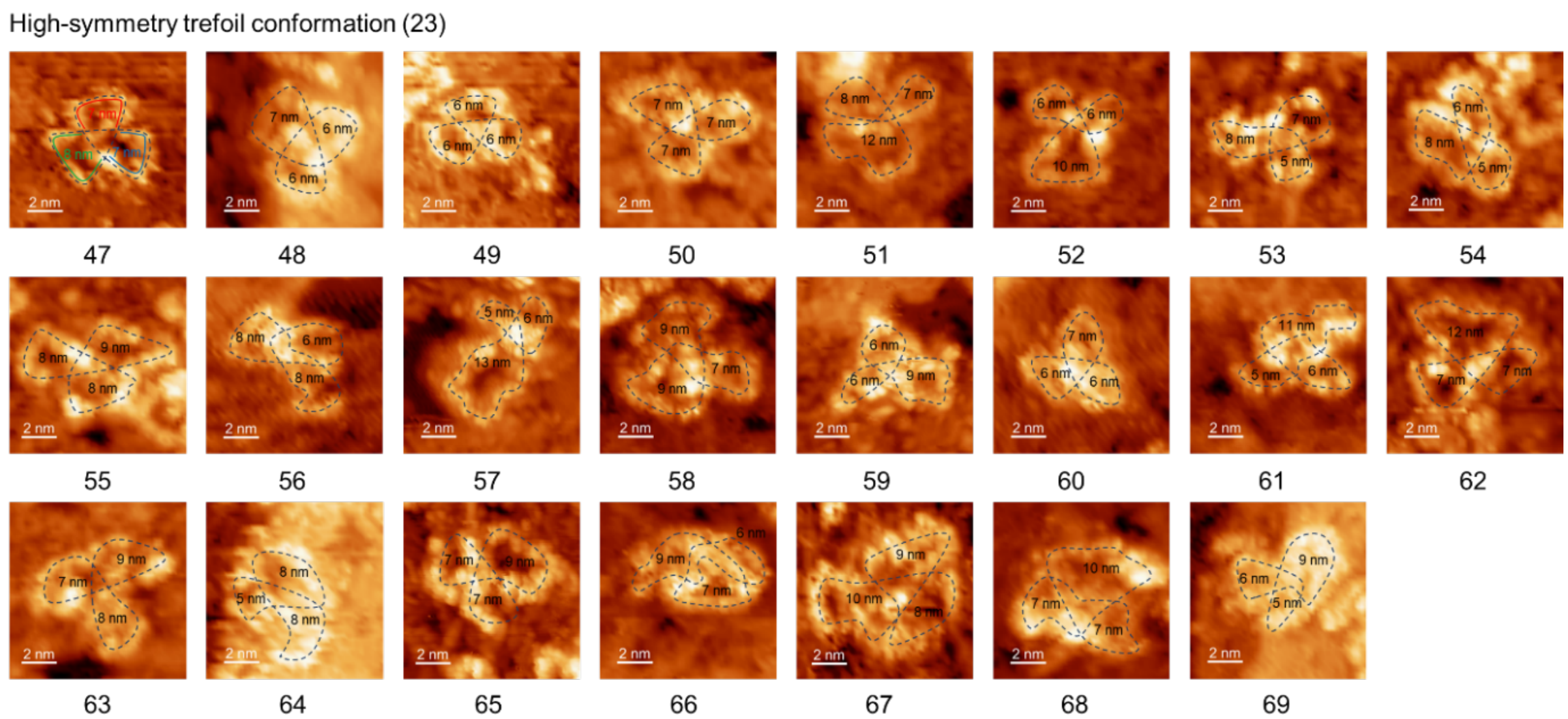

Figure 73. Single molecular STM images of high-symmetry trefoil **2** with DCM as solvent for preparing the samples. A black dashed line superimposed on each molecule outlines the skeletal structure of the low-symmetry trefoil, with the corresponding loop lengths indicated. The loop lengths are labelled in the loop. After analysing 69 individual images, it was found that 23 (33%) exhibited high-symmetry conformation (Scanning parameters: U = 2.7 V, I_t = 20 pA).

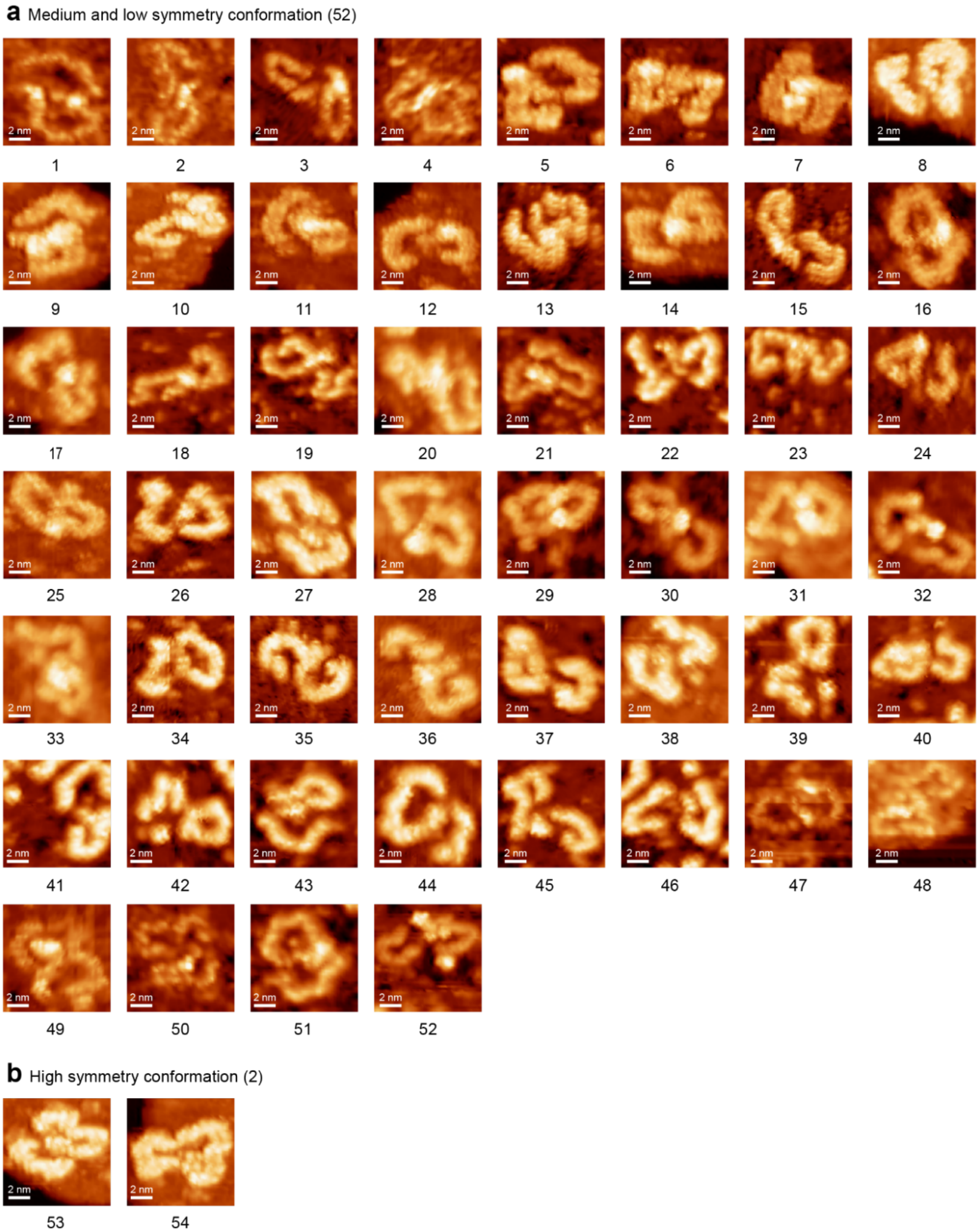

Figure 74. Single molecular STM images of **2** with the mixture of DCM and DMF as solvent (DCM: DMF, *v*: *v* = 10: 1) for preparing the samples. The STM images reveal three distinct conformations: high-, medium- and low-symmetry trefoil. For the purposes of statistical analysis, the medium- and low-symmetry trefoil conformations were considered the same isomers to avoid miscalculation. After analysing 54 individual images, it was found that only 2 (4%) exhibited the high-symmetry trefoil conformation (Scanning parameters: $U = 2.7$ V, $I_t = 20$ pA).

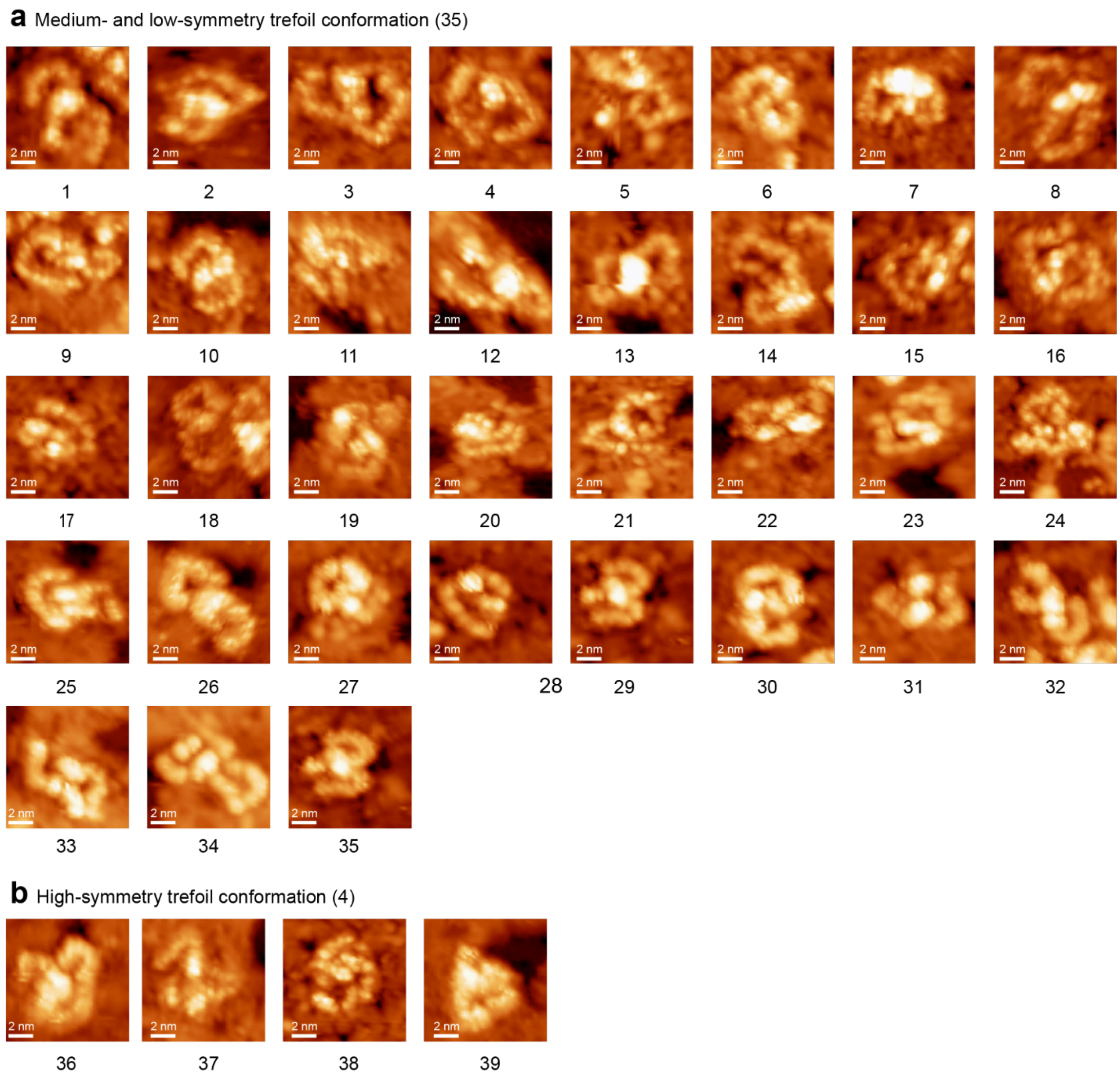

Figure 75. Single molecular STM images of **5** with DCM as solvent for preparing the samples. The STM images reveal three distinct conformations: high-, medium- and low-symmetry trefoil. For the purposes of statistical analysis, the medium- and low-symmetry trefoil conformations were considered the same isomers to avoid miscalculation. After analysing 39 individual images, it was found that only 4 (10%) exhibited the high-symmetry trefoil conformation (Scanning parameters: $U = 2.7$ V, $I_t = 20$ pA).

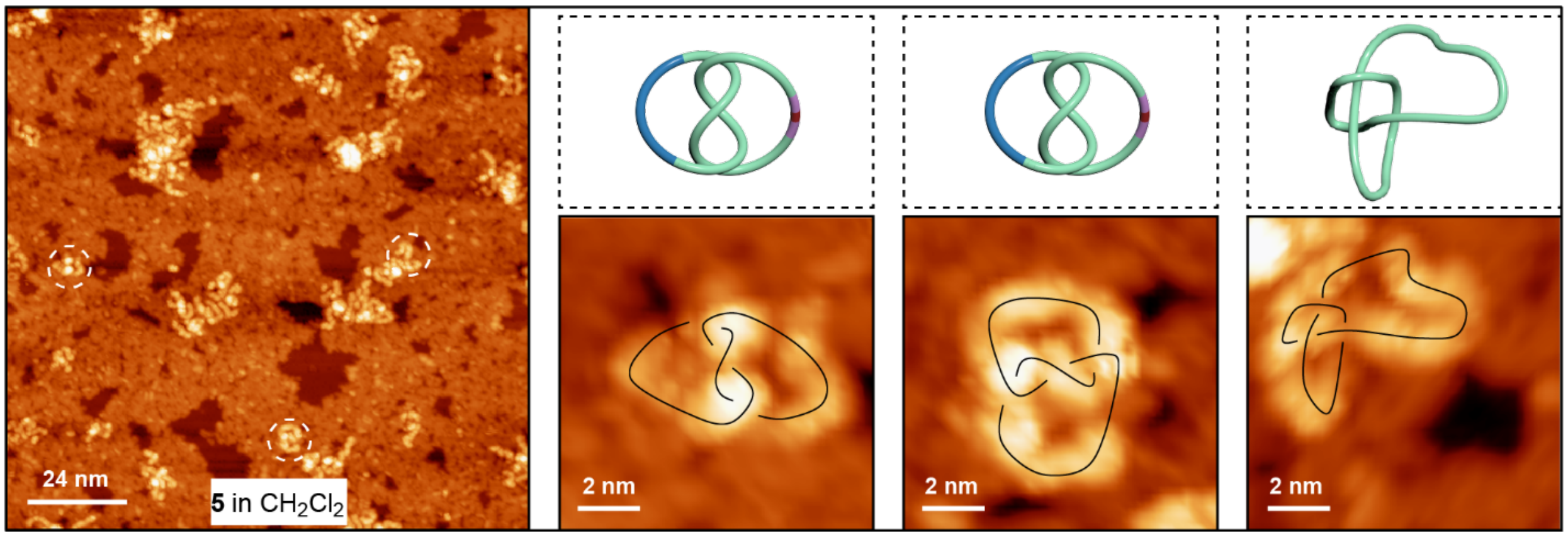

Figure 76. STM images of **5** clearly reveal medium- and low-symmetry trefoil conformations.

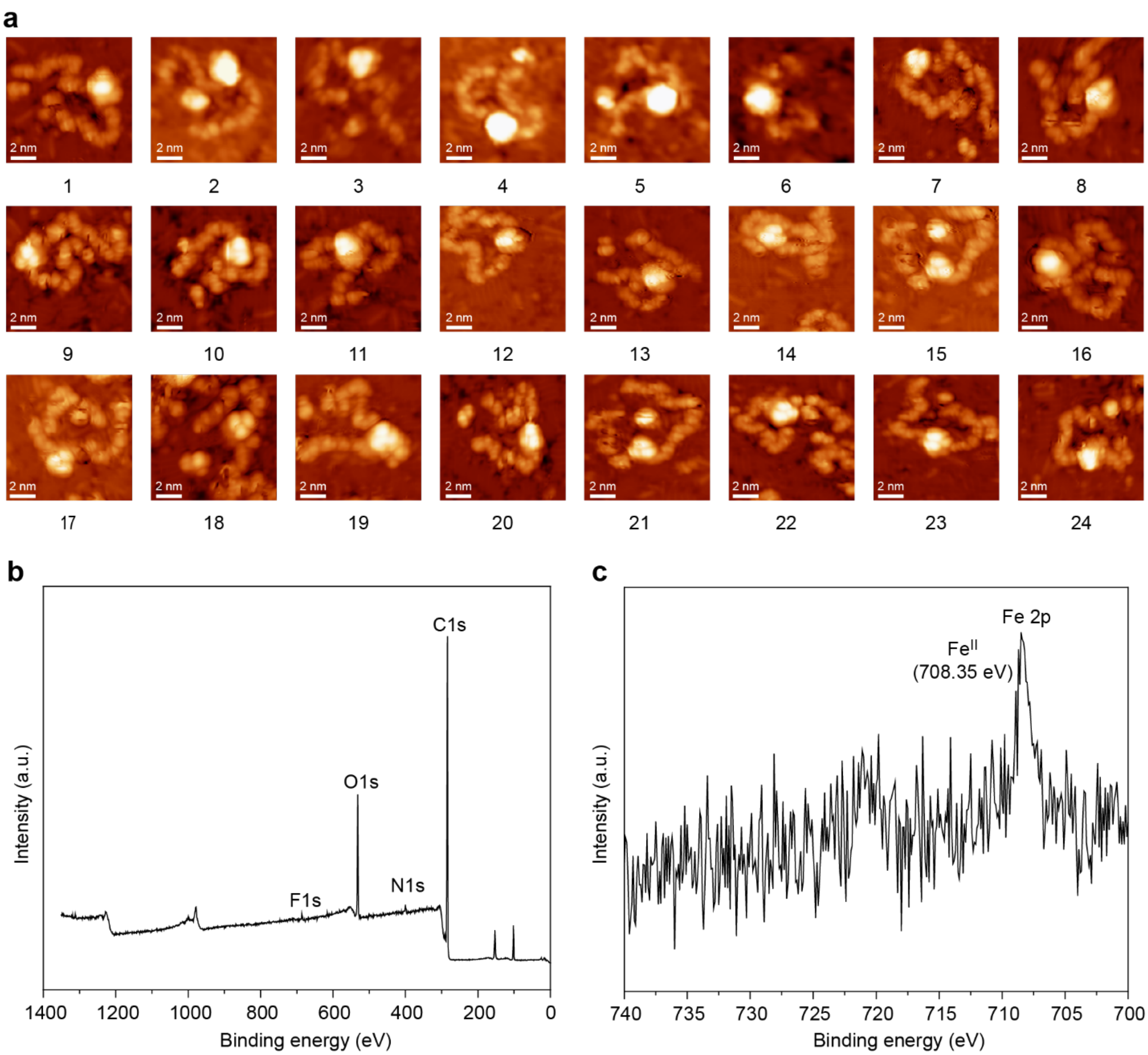

Figure 77. (a) Single molecular STM images of [Fe**2**]$(PF_6)_2$ with DCM as solvent for preparing

the samples. Two bright lobes can be observed which are attributed to metalated overhand knot (large lobe) and DIBOD moiety (small lobe) (Scanning parameters: $U = 2.7$ V, $I_t = 20$ pA). (b, c) XPS spectra of [Fe**2**]$(PF_6)_2$.

11. Coarse-graining (CG) simulation

To understand the conformational properties of a polymer knot on a surface, we carried out coarse-graining (CG) simulations of polymer knots using the LAMMPS program. The polymer chain was modelled as a string of beads, same as the previous studies.[3-5] In this model, adjacent beads are connected by bonds, described by the finite extensible nonlinear elastic (FENE) potential:

$$E_{bond} = -0.5\, KR_0^2 \ln\left[1 - \left(\frac{r}{R_0}\right)^2\right] + 4\varepsilon\left[\left(\frac{\sigma}{r}\right)^{12} - \left(\frac{\sigma}{r}\right)^6\right] + \varepsilon,$$

$$\text{with } K = 30,\ R_0 = 30,\ \varepsilon = 1,\ \sigma = 1.$$

The pairwise interaction between beads is described by the Weeks−Chandler−Andersen (WCA) potential, i.e. the Lennard-Jones (LJ) potential with the cutoff distance of $r_{\text{cut}} = 2^{1/6}a$, where a is the bead diameter and serves as the length unit.

$$E_{pair} = 4\varepsilon\left[\left(\frac{\sigma}{r}\right)^{12} - \left(\frac{\sigma}{r}\right)^6\right] + \varepsilon, \text{ with } \varepsilon = 10,\ \sigma = 1,\ r \le 2^{1/6}$$

The WCA potential is a hardcore repulsion, which is used to describe a polymer in good solvent. For each three adjacent beads, a bending potential is applied:

$$E_{\text{bend}} = (1/2)\left(L_p/a\right)\theta^2\, k_B T,$$

where θ is the bending angle, a is the hardcore diameter of bead, L_p is the persistence length to specify the polymer bending stiffness, and $k_B T$ is the Boltzmann energy and serves as the energy unit. We set $L_p = 2$ unless otherwise specified. The temperature and damping coefficient are set to 1. We started the simulation with a trefoil-knotted conformation. During the simulation, the knotted state is preserved because polymer segments cannot cross each other. We run simulations for 10^9 steps with time step of 0.01 and saved conformations every 10^5 for analysis.

— Simulation of a polymer knot confined between two plates

To mimic a polymer knot on a surface in experiments, we added a spatial confinement along the z direction such that all beads are only allowed to move between two plates $z = 0$ and $z = h$, where h is the height. The bead-wall interactions are also described by the WCA potential:

$$E_{wall} = 4\varepsilon\left[\left(\frac{\sigma}{r}\right)^{12} - \left(\frac{\sigma}{r}\right)^6\right] + \varepsilon, \text{ with } \varepsilon = 1,\ \sigma = 1,\ r \le 2^{1/6}$$

The slit height, i.e., the distance between two plates, is set to $h = 5$, unless otherwise specified. Note that due to the WCA potential for bead-wall interactions, each wall plate has a thickness of 0.5. The accessible height for the polymer chain is $5 - 2 \times 0.5 = 4$.

For a trefoil-knotted polymer on a surface or confined within a narrow slit, two distinct conformational states were identified: two-loop (two-loop) state and three-loop (three-loop) state (Fig. 78a and Fig. 79). A loop (loop) is defined as the fragment between two crossing points and locating at the exterior of conformation (not interior). The transition between these two states requires overcoming a high energy barrier associated with a flip movement and the stacking of

three segments (Fig. 78a). For h < 5, the barrier exceeds 10 k_BT, making switching infrequent or impossible. Therefore, we set h = 5 to observe at least tens of switching events over 10^9 simulation steps, enabling us to calculate the occurrence probabilities of the different states.

To facilitate a direct comparison with experimental observations, we further subdivide the three-loop (three-loop) state into high-symmetry ($L_{\text{short}} > 11$) and low-symmetry ($L_{\text{short}} \leq 11$) subclasses, where L_{short} denotes the contour length of the shortest loop. The two-loop (two-loop) state corresponds to the medium-symmetry state.

The cutoff value $L_{\text{short}} = 11$ is chosen based on the spatial resolution of the STM measurements. In our simulations, each bead represents approximately one styrene repeat unit with a backbone length of ~ 0.25 nm; thus, 11 beads correspond to a contour length of ~ 2.8 nm. When all three loops in a three-loop knot exceed this length, the conformation is unambiguously resolved as a three-loop state in STM images. In contrast, if one loop is shorter than 2.8 nm, the conformation might be misidentified as a two-loop state due to the finite STM resolution.

For this reason, in the main text we report the fraction of high-symmetry conformations rather than the total fraction of three-loop conformations. Identification of high-symmetry states, defined by all loop lengths exceeding 2.8 nm, is more reliable in STM images than identification of all three-loop states, which includes configurations with sub-resolution loops. Since the STM resolution is independent of polymer length, the same cutoff value $L_{\text{short}} = 11$ is used for both polymer lengths studied in the experiments.

To quantify the conformational fluctuations in high-, medium- and low- symmetry states, we calculated three conformational tubes in Fig. 78c. In each graph, the tube axis corresponds to the average conformation of a state and the tube diameter corresponds to the fluctuation of individual conformations around the average conformation, which is usually referred to as the root mean square displacement (RMSD). The high-symmetry state exhibits reduced entropy compared to the low- and medium-symmetry states, as shown by the RMSD values in Fig. 78d.

In most simulations, bead-bead interactions were described by WCA potentials, which corresponds to polymers in good solvent. To capture the slightly poor solvent quality in experiments, we used another bead-bead interaction: Lennard-Jones potential with the cutoff distance of $r_{\text{cut}} = 1.2a$.

$$E_{pair} = 4\varepsilon\left[\left(\frac{\sigma}{r}\right)^{12} - \left(\frac{\sigma}{r}\right)^{6}\right] + \varepsilon, \text{ with } \varepsilon = 10, \sigma = 1, r \leq 1.2.$$

It means the bead-bead interaction is attractive when the bead-bead distance is between $2^{1/6}a$ and $1.2a$. We found that the weak attraction decreased the probability of the high-symmetry trefoil conformation, in agreement with experimental results.

We also compared the bending curvature in two conformational regions: outer region of loops and interior (Fig. 78e). For each region, we calculated the average bending energy per monomer, using $E_{bend} = (1/2)L_p\theta^2(k_BT)$ with $L_p = 2$. The difference in average bending energy between the two regions was calculated. In all three conformers, the loop region exhibited higher bending curvature than the interior, suggesting that a soft segment prefers to stay at the outer loop regions (relatively more curvature) and a stiff segment prefers to stay at the interior regions (relatively less curvature). To validate this prediction, we performed additional simulations with a soft segment ($L_p = 0$) or a stiff segment ($L_p = 10$) in a knotted polymer ($L_p = 2$ at other monomers). The segment length is 9 and the polymer length is 80. As predicted, our simulations observed a higher occurrence probability of the soft segment at the outer loop regions than the case of average

distribution (88% *vs.* 79%), as shown in Fig. 78f. We also observed a higher occurrence probability of the stiff segment at the interior regions than the case of average distribution (29% *vs.* 21%), as shown in Fig. 78f.

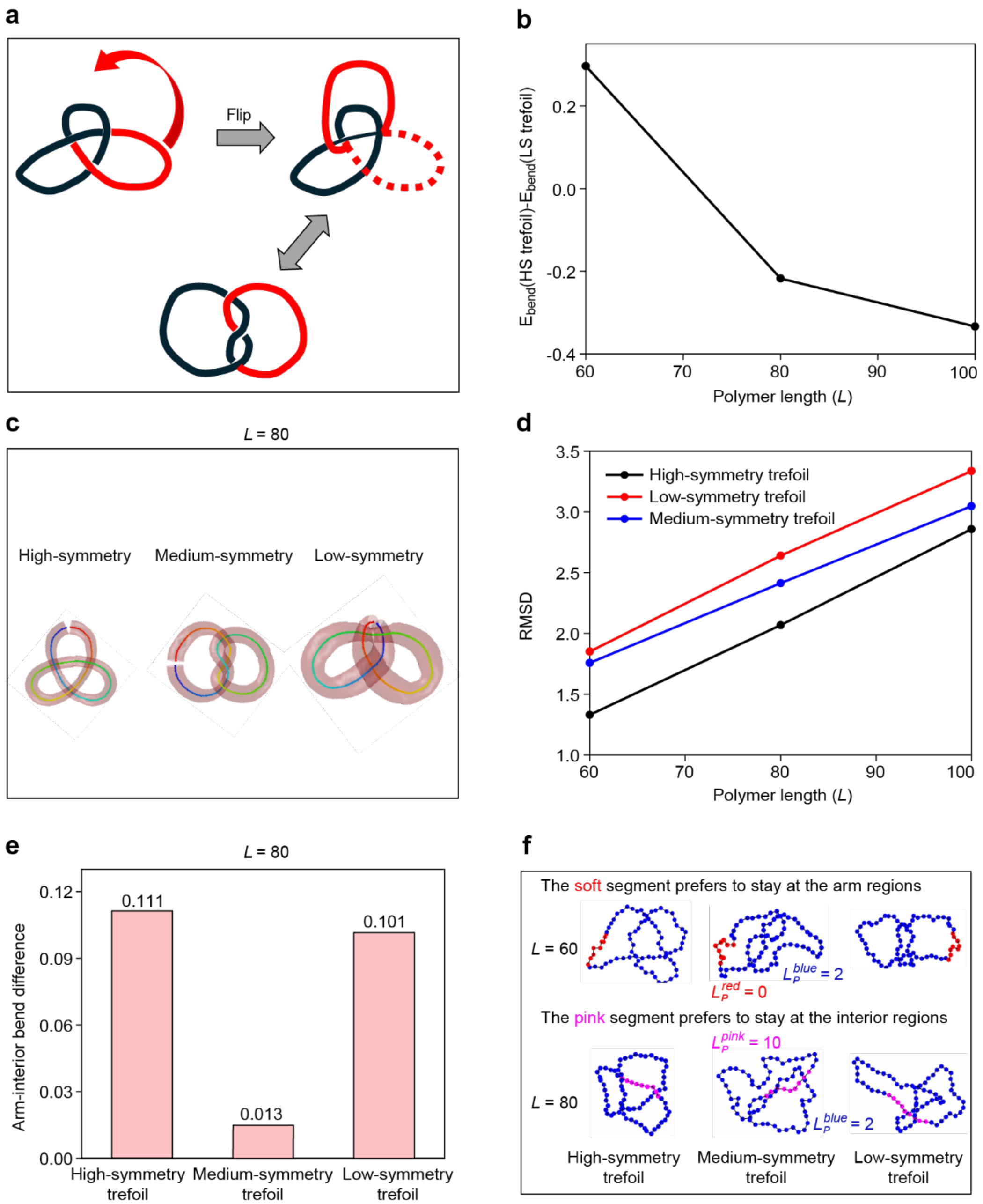

Figure 78. Conformational diversity and transitions in knotted polymers. (a) The transition between the trefoil and helical conformations requires a flipping operation. During the flipping, a segment slides over a crossing point, which produces the stacking of three segments and produces a high-energy barrier. (b) The difference in the average bending energy between the high-symmetry and low-symmetry trefoil conformations from simulations. The energy unit is k_BT. (c) Simulation results of the average conformations for the three states with L=80 and L_p=2. In each graph, the tube axis corresponds to the average conformation of a state and the tube diameter corresponds to

the fluctuation of individual conformations around the average conformation, or RMSD. (d) Comparison of RMSD values for three states. (e) The difference in the bending curvatures in the loop and interior regions of three states. (f) Simulation snapshots of a soft or stiff segment in the knot conformation.

— Algorithm for distinguishing two-loop and three-loop polymer conformations

We develop a geometric criterion to distinguish two-loop and three-loop polymer conformations on a surface. Here, the two-loop state corresponds to the medium-symmetry trefoil, and the three loop-state include high-symmetry and low-symmetry trefoils. As illustrated in Fig. 79a, a two-loop conformation contains three crossings, labeled A, B, and C. Two loops connect crossing points A and C and form a closed curve, indicated by the dashed line in Fig. 79a. The defining feature of a two-loop conformation is that the remaining crossing point, B, lies inside this closed curve. In contrast, a three-loop conformation does not satisfy this criterion. As shown in Fig. 79b, for a three-loop conformation, each crossing point lies outside the closed curve formed by the two loops connecting the other two crossings.

In a two-loop conformation, only one of the three crossings satisfies the "inside-the-curve" criterion. Therefore, we apply this test to all three crossings and identify the conformation as two-loop if exactly one crossing is found to be inside the corresponding closed curve. This algorithm applies to conformations containing exactly three crossings. In our simulations, only a small fraction (a few percent) of conformations contain more than three crossings, as additional crossings produce extra energetic costs. These rare conformations are excluded from the analysis.

To determine whether a point lies inside a closed curve in two dimensions, we employ the ray-casting algorithm. A point is inside the curve if a ray starting from the point intersects the curve an odd number of times. In practice, we choose a horizontal ray, as illustrated in Fig. 79a. In our simulations, the closed curve is represented by a sequence of beads and thus forms a polygon. To determine whether the ray intersects a given polygon segment, we use the criterion $(y_i - y_B)(y_{i+1} - y_B) < 0$ and $x_{i+1} > x_B$, where (x_B, y_B) are the coordinates of the crossing point B, and (x_i, y_i) and (x_{i+1}, y_{i+1}) are the coordinates of the two beads defining the segment.

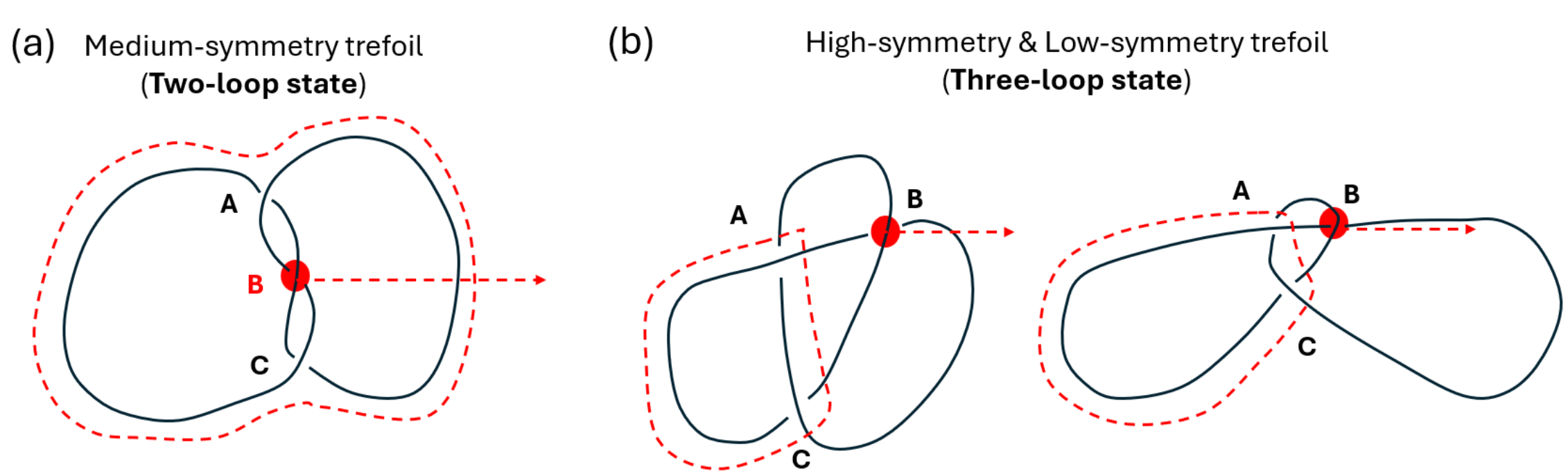

Figure 79. Algorithm for distinguishing two-loop and three-loop polymer conformations. (a) The two-loop state satisfies a criterion: a crossing (red dot) locates inside the closed curve (dashed curve) formed by the two loops connecting the remaining two crossings. When the red dot is in

the red dashed loop, the red arrow intersects the red dashed loop an odd number of times. (b) The three-loop state does not satisfy this criterion. When the red dot is not in the red dashed loop, the red arrow intersects the red dashed loop an even number of times.

— **Simulation of an adsorbed polymer knot**

We perform additional simulations of adsorbed polymer knots to mimic the polymer knots in experiments (Fig. 80). In these simulations, there is only one wall. A polymer conformation is adsorbed by an attractive LJ potential between the wall and beads:

$$E_{wall} = 4\varepsilon_{wall}\left[\left(\frac{\sigma}{r}\right)^{12} - \left(\frac{\sigma}{r}\right)^{6}\right] + \varepsilon_{wall} \text{ for } r < r^{*}_{wall}$$

The attraction strength and range are $\varepsilon_{wall} = 2$ and $r^{*}_{wall} = 3$ unless otherwise specified. The parameters of polymer interactions remain unchanged as described above. As shown in Fig. 80a, the high-symmetry probability of adsorbed knots increases with polymer length, exhibiting the same trend as observed in experiments and in confined-knot simulations. The high-symmetry probability of adsorbed knots decreases upon introducing weak bead–bead attraction (Fig. 81b), again consistent with both experimental observations and confined-knot results.

Notable differences between adsorbed and confined knots are observed in Fig. 81a). These differences are not surprising, as adsorbed polymers retain finite fluctuations in their conformational height, whereas confined polymers are strictly restricted by the slit geometry. To further elucidate the origin of these differences, we systematically vary the bead–wall attraction strength for adsorbed knots and the slit height for confined knots. As shown in Fig. 81c–d, the high-symmetry probability decreases with increasing bead–wall attraction strength, while it increases with increasing slit height. These two sets of results are mutually consistent: a stronger bead–wall attraction effectively corresponds to a smaller slit height, leading to enhanced confinement of polymer conformations.

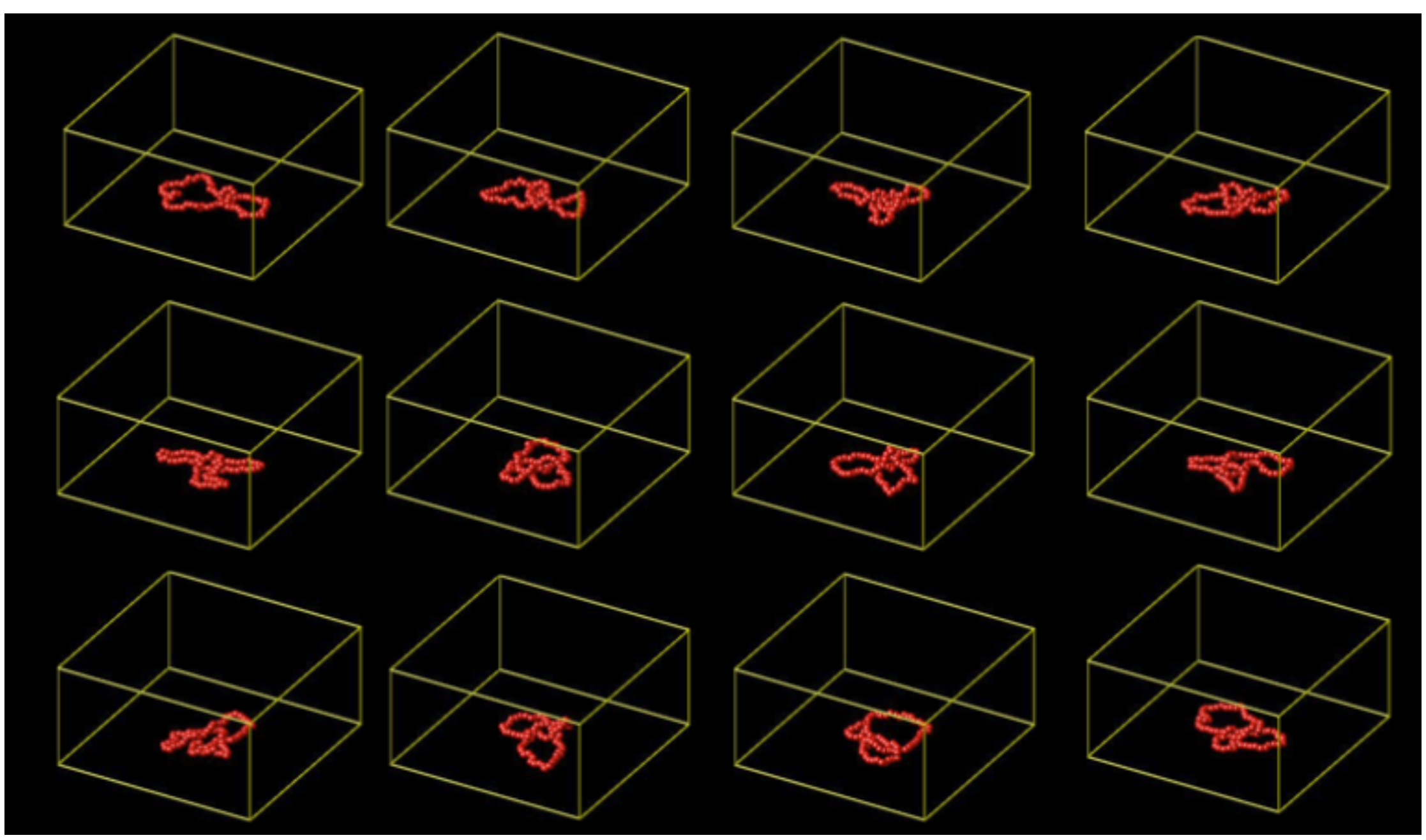

Figure 80. Simulation snapshots of an adsorbed polymer knot.

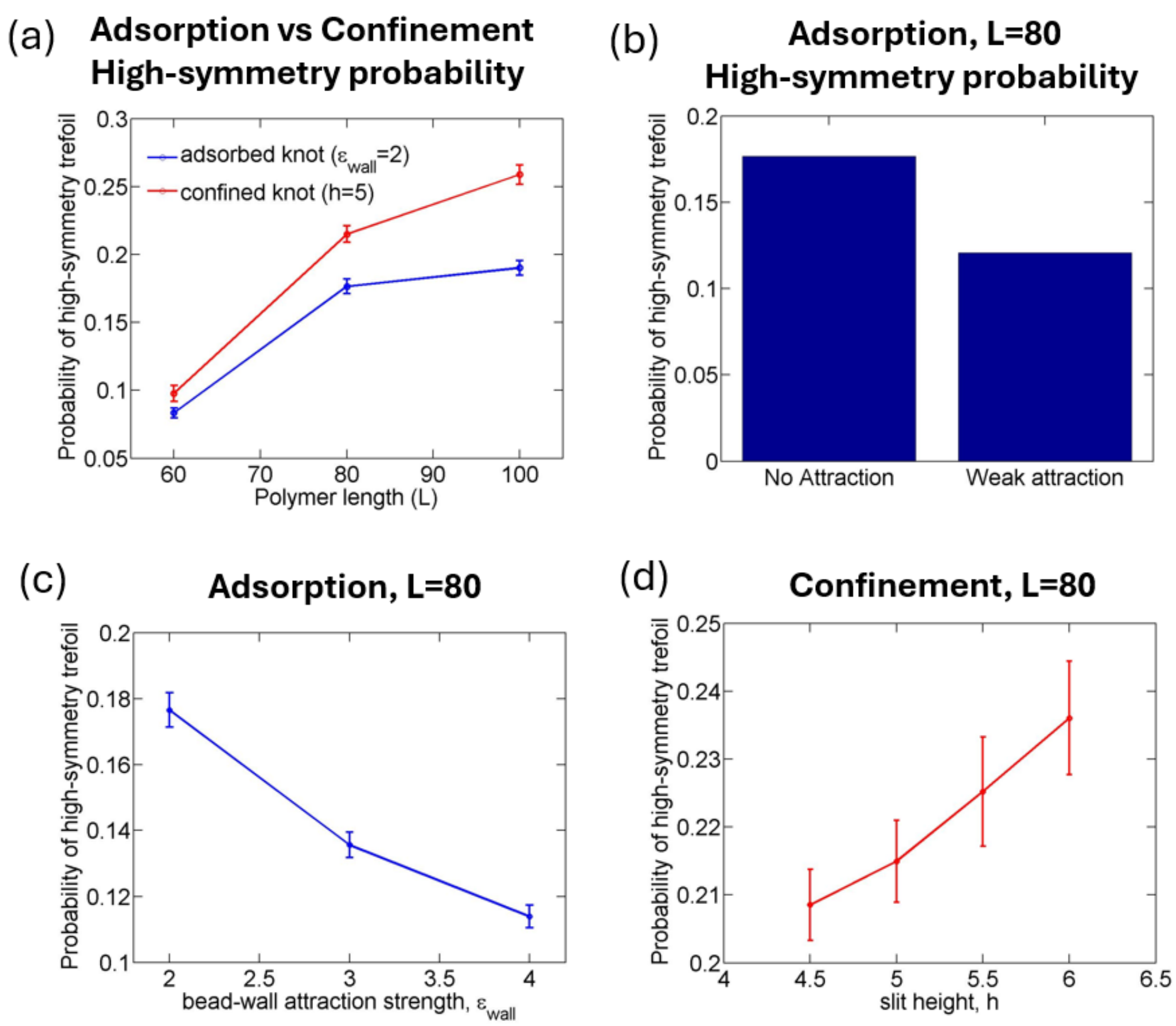

Figure 81. Simulation results of absorbed knots and comparison with confined knots. (a) High-symmetry probability as a function of polymer length for adsorbed and confined knots. (b) High-symmetry probability of adsorbed knots with and without weak bead–bead attraction. (c) High-symmetry probability as a function of bead–wall attraction strength. (d) High-symmetry probability as a function of slit height for confined knots.

— Discussion about the three-loop probability *vs.* high-symmetry probability

The tightest trefoil knots are expected to adopt the three-loop state. Accordingly, we calculate the three-loop probability as a function of polymer length. As shown in Fig. 82, the three-loop probability increases as the polymer becomes shorter for both adsorbed and confined knots, consistent with this expectation.

In the main text, however, we analysed the high-symmetry probability rather than the three-loop probability, because identification of high-symmetry states, defined as configurations in which all loop lengths exceed a threshold, is more reliable in STM images. In contrast, identification of all three-loop states includes configurations with one or more sub-resolution loops, which cannot be unambiguously resolved experimentally.

We note that for shorter polymers, the three-loop probability increases whereas the high-symmetry probability decreases. This opposite trend arises because, for shorter three-loop knots, the loop lengths are more likely to fall below the experimental resolution and therefore are not classified as

high-symmetry states.

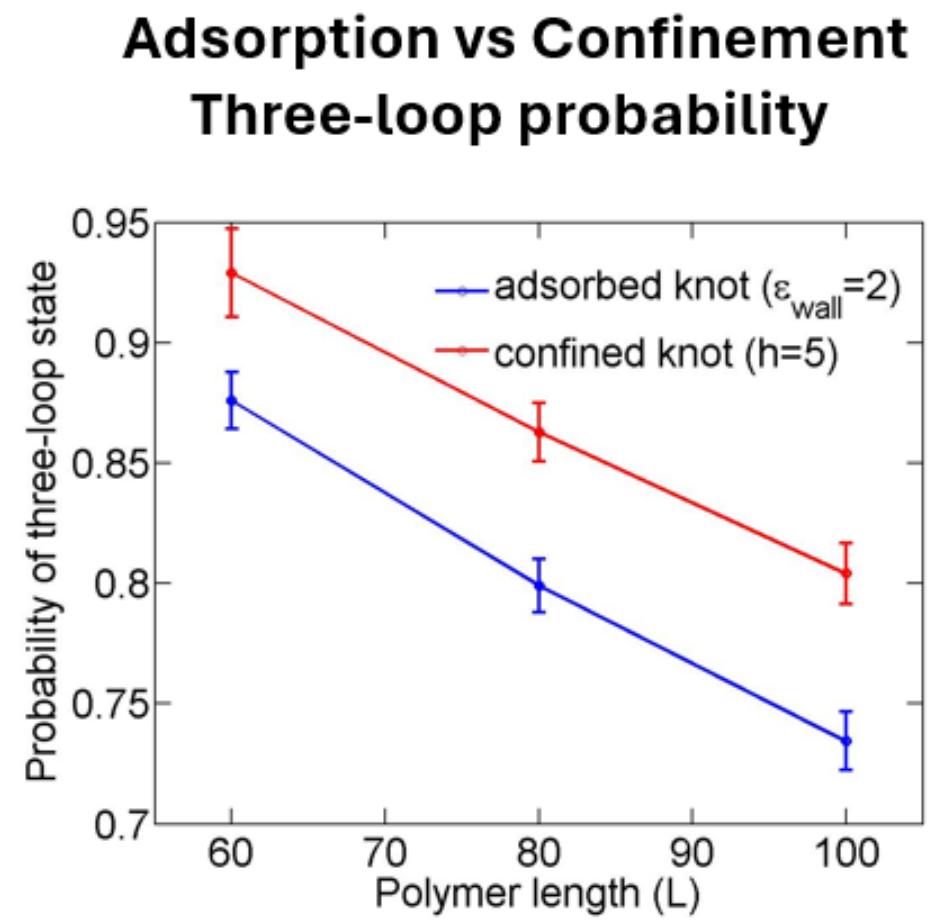

Figure 82. Three-loop probability as a function of polymer length for adsorbed and confined knots.

— Discussion about the knot localization in the long-chain limit

Many previous studies[6-11] have demonstrated knot localization in sufficiently long polymers, where the most probable knot-core length becomes insensitive to the total chain length. To assess this regime in our system, we performed additional simulations of relatively long polymers confined in a slit with height $h = 5$. As shown in Fig. 83, the most probable knot length (*i.e.*, the peak position of the distribution) increases from 35 to 45 as the chain length increases from 60 to 80. Upon further increasing the chain length beyond 80, the peak position remains fixed at 45, indicating the onset of knot localization.

The threshold chain length required to reach the long-chain limit depends on both the polymer persistence length and the slit height. For a persistence length of $L_p = 2$ and a slit height of $h = 5$, meaningful tests of knot localization require comparing knot-length distributions for chains substantially longer than the most probable knot-core length (~45), typically at least twice as long (*i.e.,* ~90). In contrast, the PS contour lengths used in the present study (62 and 88) lie below this threshold and therefore do not reach the long-chain limit necessary for probing knot localization.

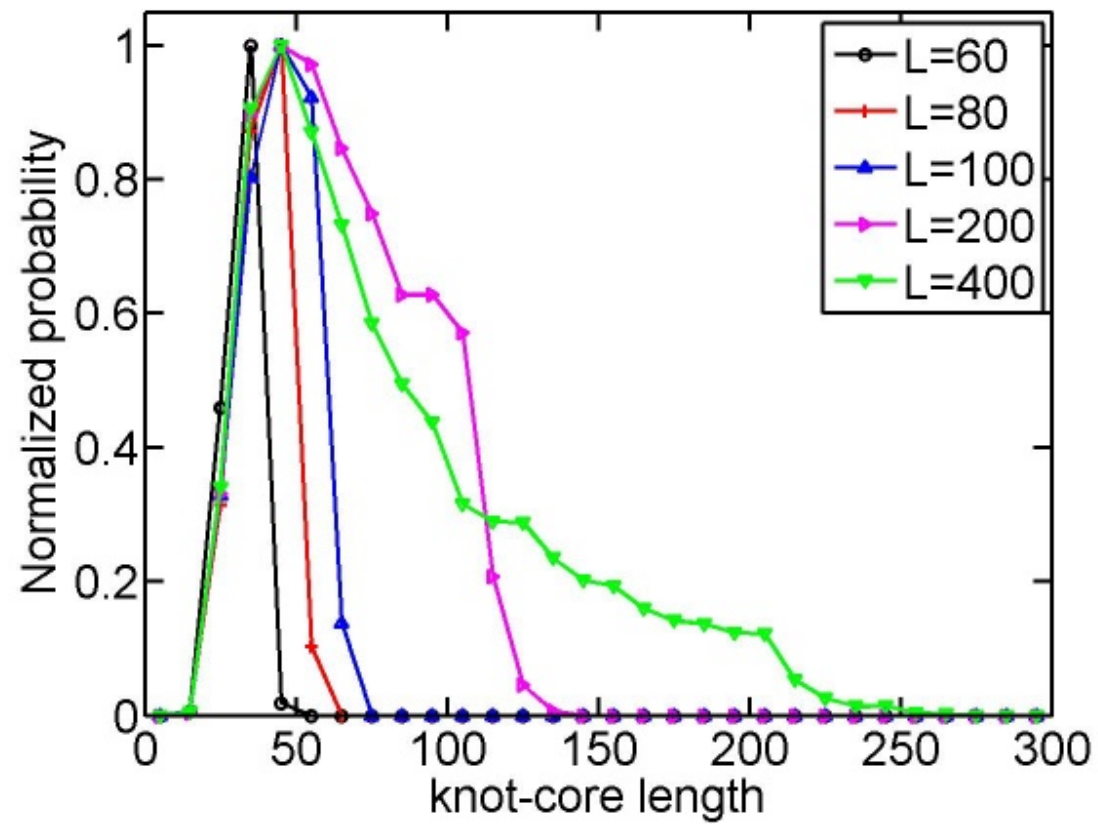

Figure 83. Simulation results of knot-length distribution for confined polymer knots in a slit. The slit height is $h = 5$.

12. Synthesis of linear (7, 9) and trefoiled (8, 10) polymers

12.1 Synthesis of S31 and S33

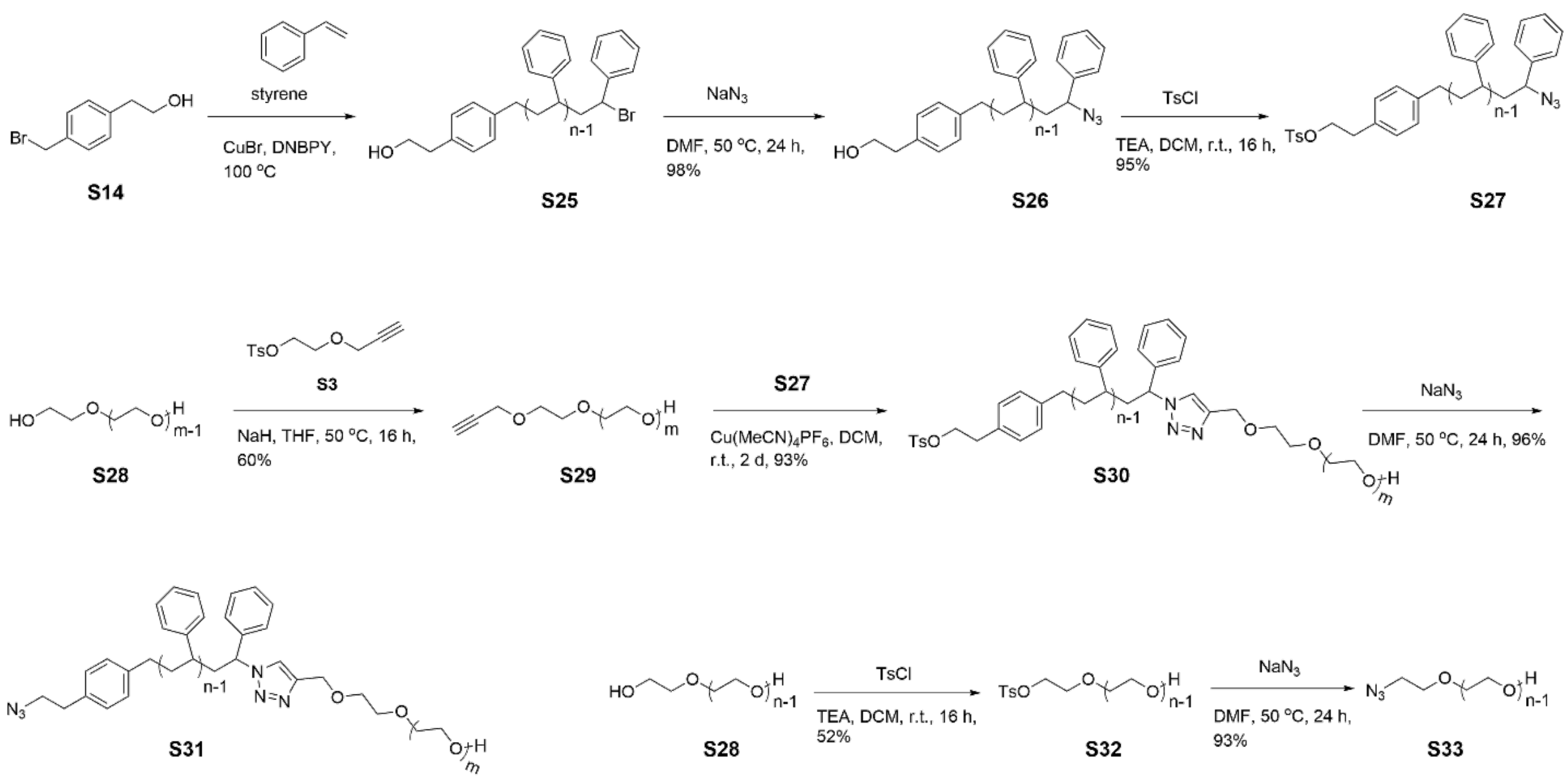

Figure 84. Synthesis of **S31** and **S33**.

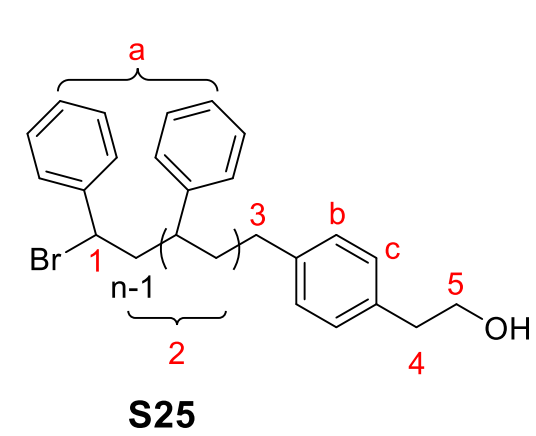

S14 (0.10 g ,0.47 mmol), styrene (7.29 g, 70.09 mmol) and 4,4'-dinonyl-2,2-bipyridine (0.38 g,

0.94 mmol) were added to a two-necked round bottom flash in N_2 atmosphere. The mixture was degassed by three freeze-pump-thaw cycles and backfilled with nitrogen. The mixture was added CuBr (145.8 mg, 1.03 mmol) in N_2 atmosphere and degassed again by freeze-pump-thaw cycle. The reaction mixture was stirred at 100 °C for 2 hours. After completion, the reaction mixture was wlooped to room temperature and diluted with THF. The copper complex was removed by neutral alumina column. The product was precipitated from MeOH for three times and dry at 60 °C for 6 hours to give **S25** as white solid (2.60 g, M_n = 2910 Da, $Đ$ = 1.11).

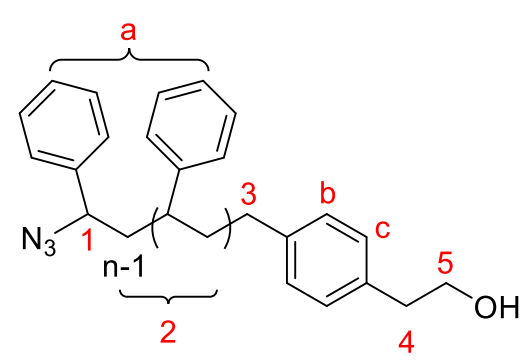

S26

To a stirred solution of **S25** (1.20 g) in DMF (20 mL) was added NaN_3 (0.20 g). The reaction mixture was stirred at 50 °C for 24 h. After completion, the reaction mixture was wlooped to room temperature and diluted with water. White solid precipitated from the mixture, filtration and wash the filter cake with water and MeOH. The solid was redissolved in DCM and concentrated under vacuum. The product was precipitated from MeOH and dry at 60 °C for 6 hours to give **S26** as white solid (1.20 g, yield 98%, M_n = 2937 Da, $Đ$ = 1.11).

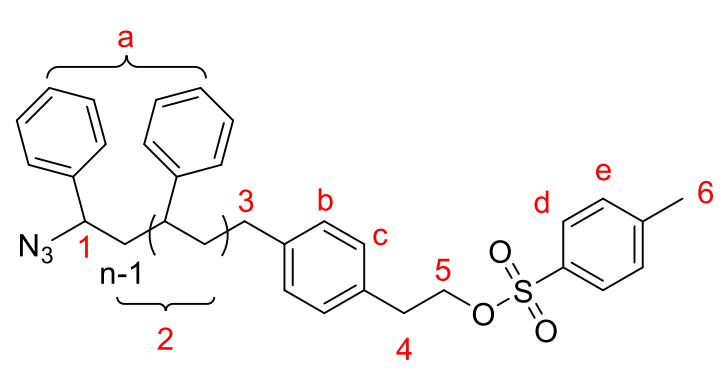

S27

To a stirred solution of **S26** (1.00 g, 0.33 mmol), TEA (0.10 g, 0.99 mmol) in dry DCM (20 mL) was slowly added the solution of TsCl (0.16 g, 0.83 mmol) in dry DCM (5 mL) at 0 °C. Then the mixture was wlooped to room temperature and stirred for 16 hours at room temperature. After completion, the reaction mixture was concentrated under vacuum, and the product was precipitated from MeOH. Filtration and the solid was dissolved in DCM. After concentrated, the product was precipitated from MeOH again, collection of the solid cake and dry at 60 °C for 6 hours to give **S27** as white solid (1.00 g, yield 95%, M_n = 3132 Da, $Đ$ = 1.11).

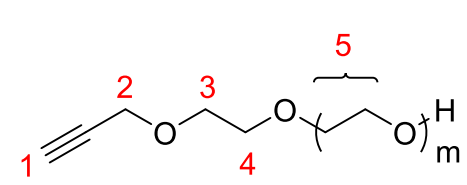

S29

S28 (8.00 g, 13.33 mmol) and NaH (0.13 g, 5.33 mmol) in 250 mL two-neck round bottom bottle was added dry THF (80 mL) in N_2 atmosphere. The reaction mixture was heated to 50 °C and then **S3** (0.68 g, 2.67 mmol) in dry THF (10 mL) was added dropwise to the mixture for 2 hours. The reaction mixture was stirred at 50 °C for 16 hours. After completion, the reaction mixture was quenched with water (10 mL) and extracted with dichloride methane. The organic phase was dry

with anhydrous sodium sulfate and evaporated to dryness. The crude product was purified by silica gel column using DCM and MeOH (DCM: MeOH = 20 :1) as eluent to give **S29** as colorless oil (1.10 g, yield 60%, M_n = 648 Da, $Đ$ = 1.11).

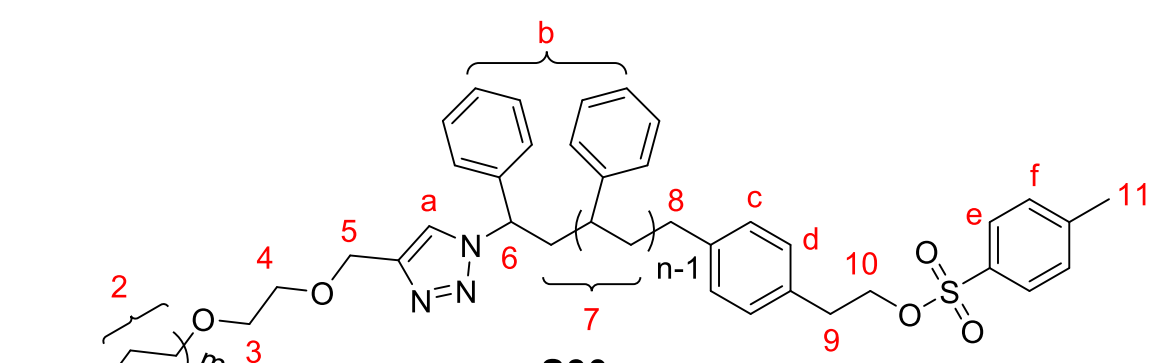

S30

S29 (0.15 g, 0.22 mmol), **S27** (0.45 g, 0.15 mmol), $Cu(MeCN)_4·PF_6$ (55.9 mg, 0.15 mmol) were dissolved in DCM (10 mL) in a sealed vial in N_2 atmosphere. The reaction mixture was stirred at room temperature for 48 hours. After completion, the crude product was purified by silica gel column using DCM and MeOH (DCM: MeOH = 20: 1) as eluent to give **S30** as white solid (0.51 g, yield 93%, M_n = 3895 Da, $Đ$ = 1.09).

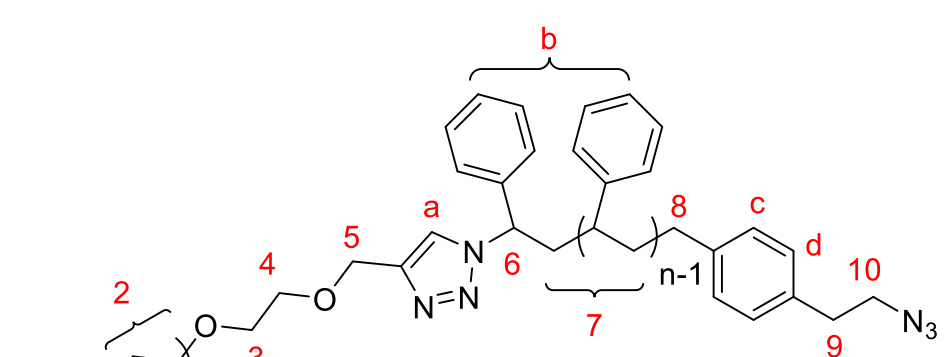

S31

To a stirred solution of **S30** (0.45 g) in DMF (20 mL) was added NaN_3 (12 mg). The reaction mixture was stirred at 50 °C for 24 h. After completion, the reaction mixture was wlooped to room temperature and diluted with water. White solid precipitated from the mixture, filtration and wash the filter cake with water and MeOH. The solid was redissolved in DCM and concentrated under vacuum. The product was precipitated from MeOH and dry at 60 °C for 6 hours to give **S31** as white solid (0.43 g, yield 96%, M_n = 2804 Da, $Đ$ = 1.07).

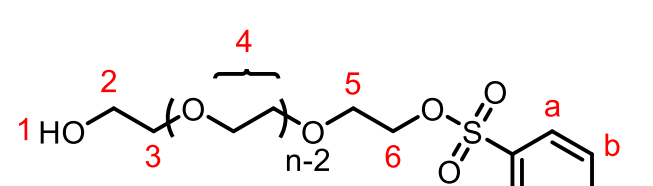

S32

To a stirred solution of **S28** (8.00 g, 13.33 mmol), TEA (1.41 g, 14.0 mmol) in dry DCM (60 mL) was slowly added the solution of TsCl (0.89 g, 4.67 mmol) in dry DCM (10 mL) at 0 °C. Then the mixture was wlooped to room temperature and stirred for 16 hours at room temperature. After completion, the reaction mixture was concentrated under vacuum. The crude product was purified by silica gel column using DCM and MeOH (DCM: MeOH = 20: 1) as eluent to give **S32** as colorless oil (1.83 g, yield 52%).

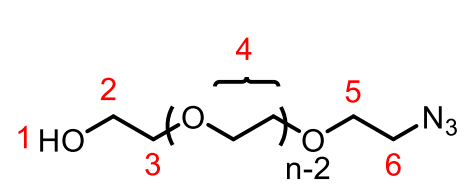

S33

To a stirred solution of **S32** (0.70 g) in DMF (15 mL) was added NaN_3 (78.5 mg). The reaction mixture was stirred at 50 °C for 24 h. After completion, the reaction mixture was wlooped to room temperature and remove the solvent under vacuum. The crude product was purified by silica gel column using DCM and MeOH (DCM: MeOH = 20: 1) as eluent to give **S33** as colorless oil (0.54 g, yield 93%, M_n = 539 Da, $Đ$ = 1.09).

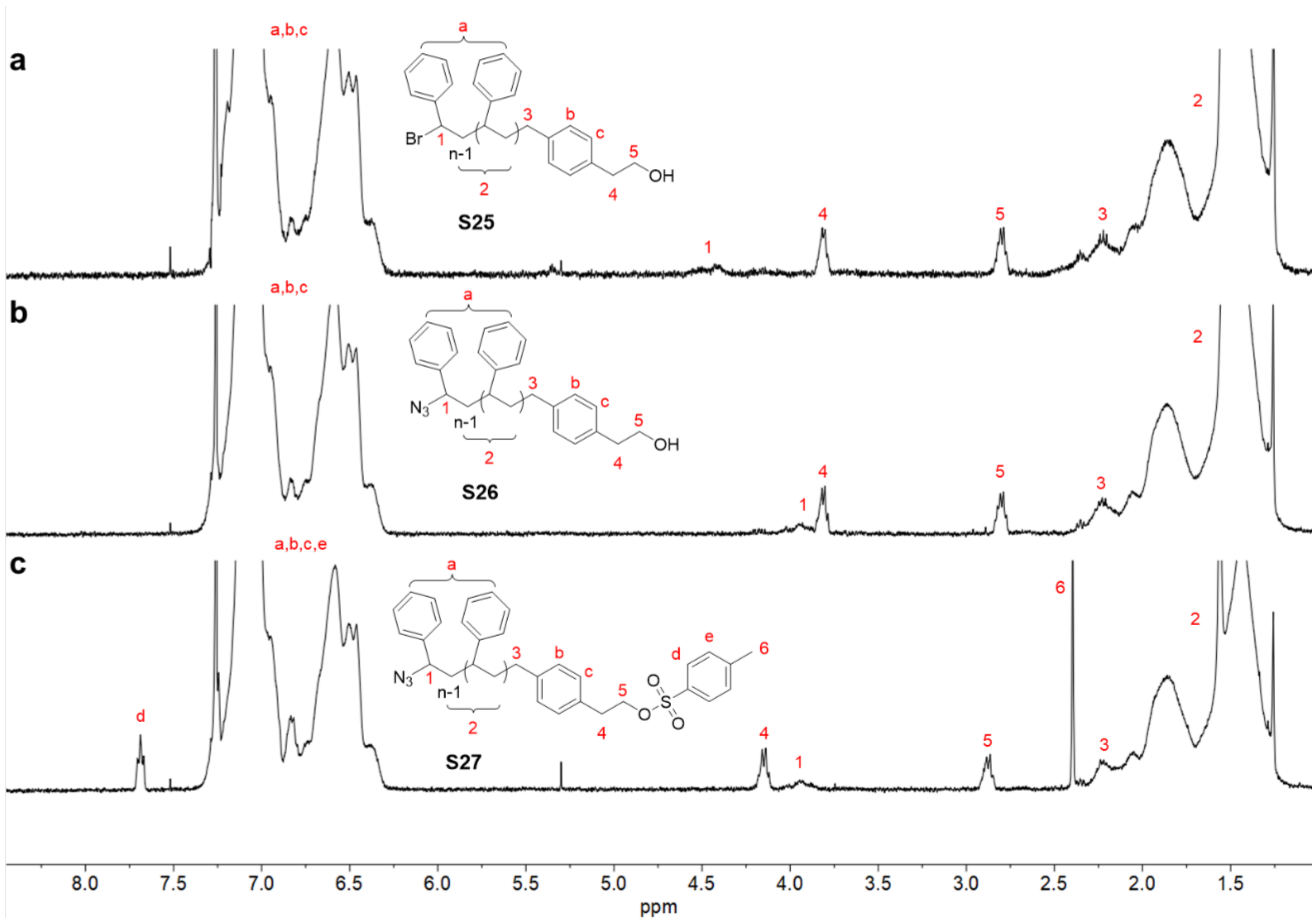

Figure 85. ^{1}H NMR spectra (400 MHz, $CDCl_3$, 298 K) of (a) **S25**; (b) **S26**; (c) **S27**.

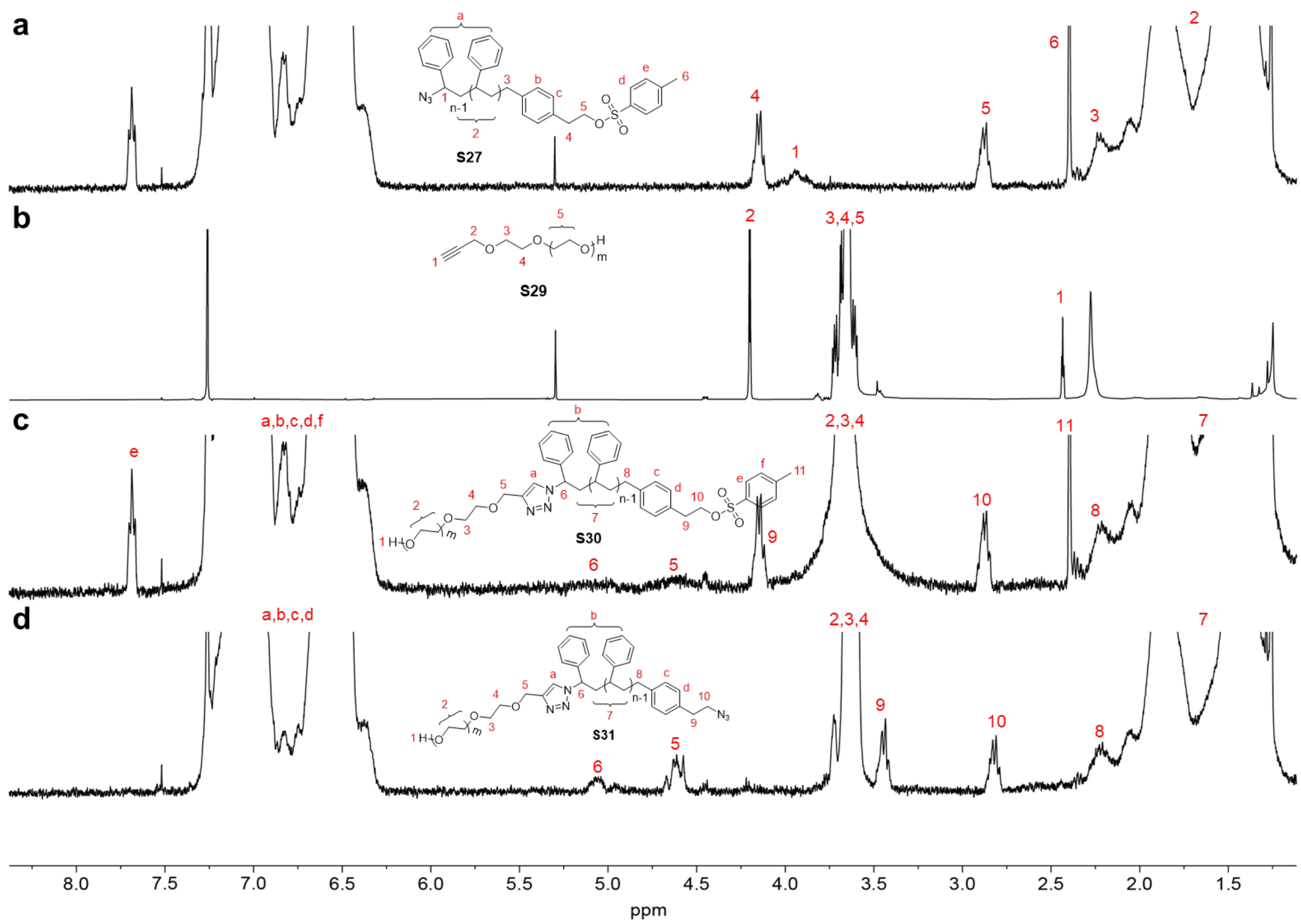

Figure 86. ^{1}H NMR spectra (400 MHz, $CDCl_3$, 298 K) of (a) **S27**; (b) **S29**; (c) **S30**; (d) **S31**.

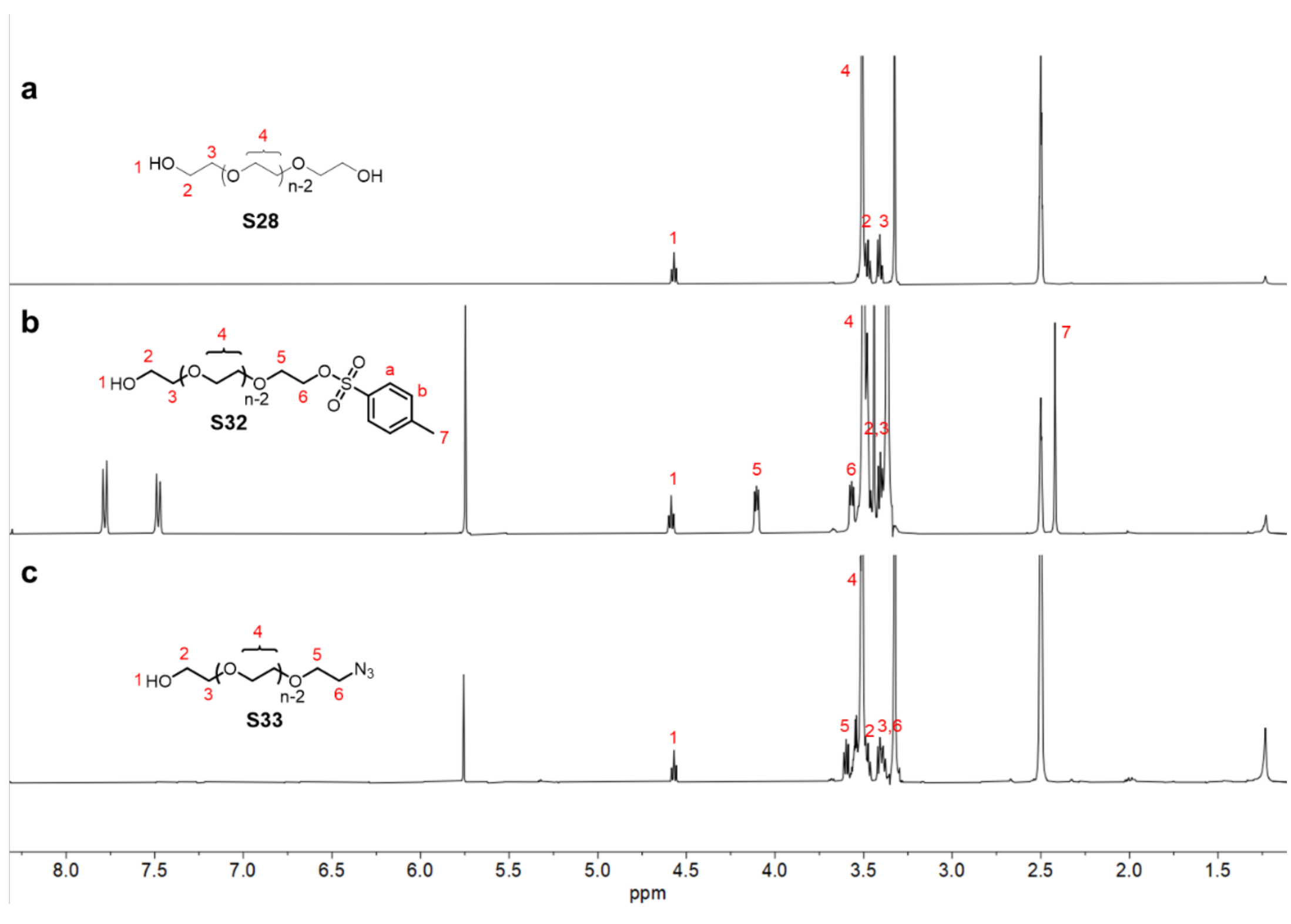

Figure 87. ^{1}H NMR spectra (400 MHz, $CDCl_3$, 298 K) of (a) **S28**; (b) **S32**; (c) **S33**.

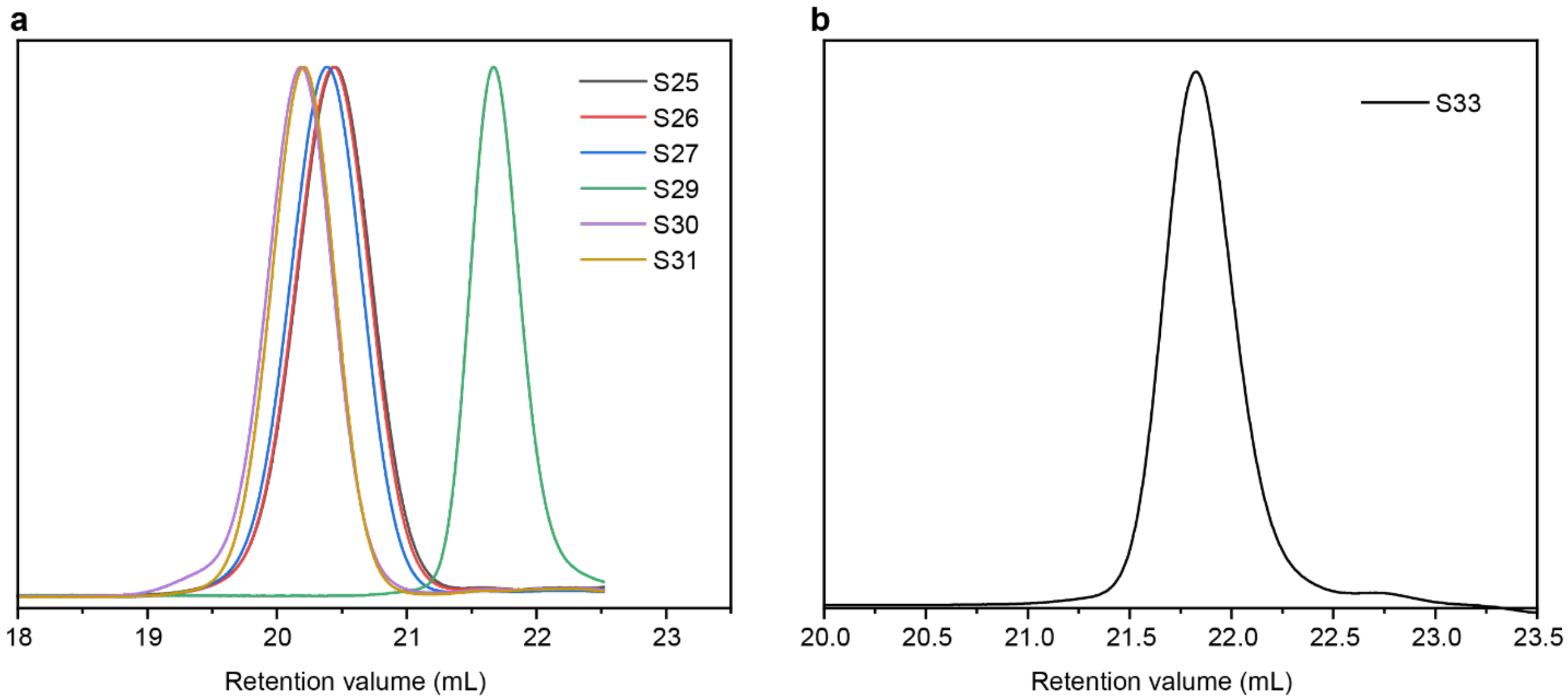

Figure 88. GPC curves of (a) **S25**, **S26**, **S27**, **S29**, **S30**, **S31** and (b) **S33**.

12.2 Synthesis of linear (7) and trefoiled (8) PEG polymers

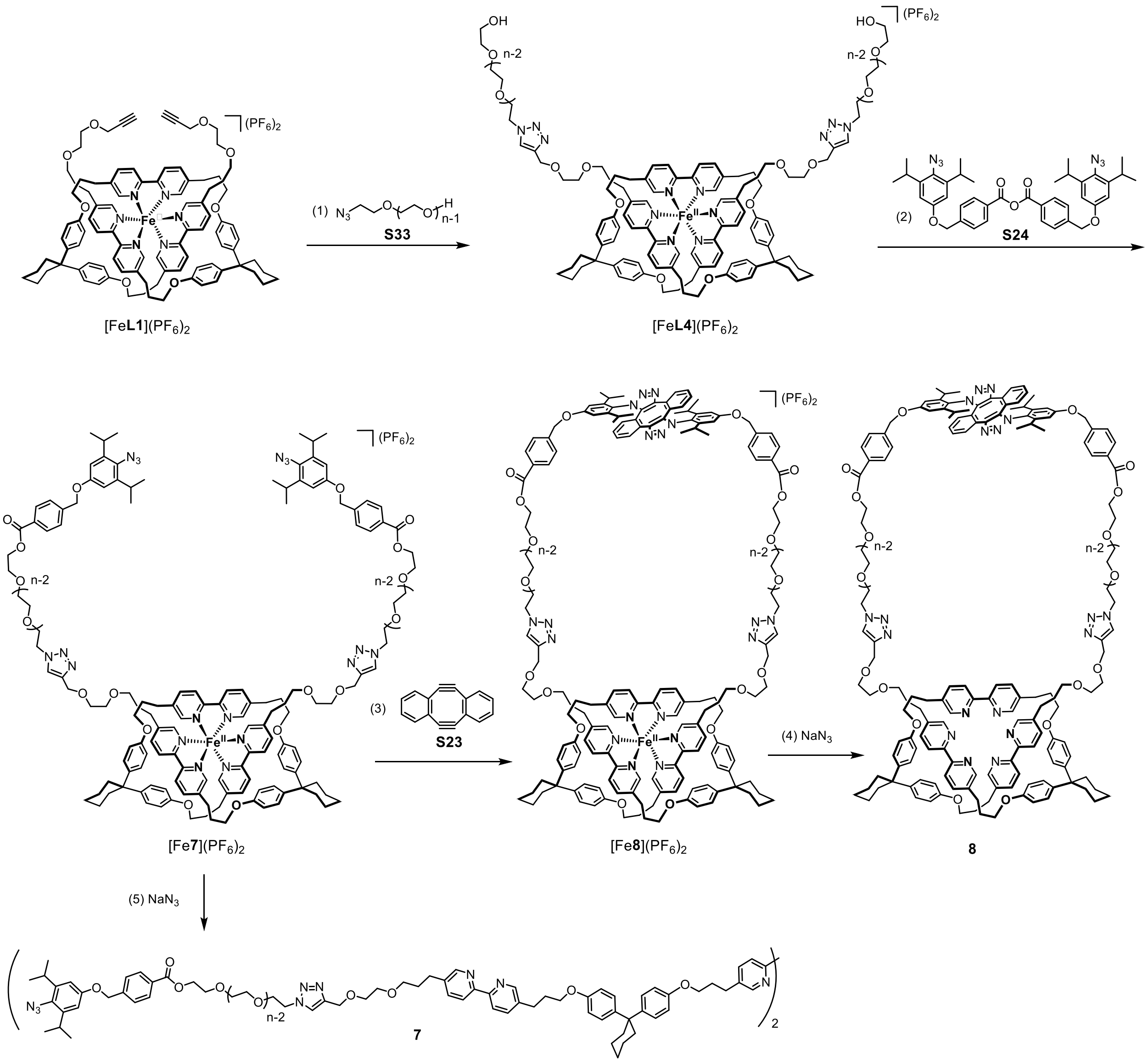

Figure 89. Synthesis of linear (**7**) and trefoiled (**8**) PEG polymers.

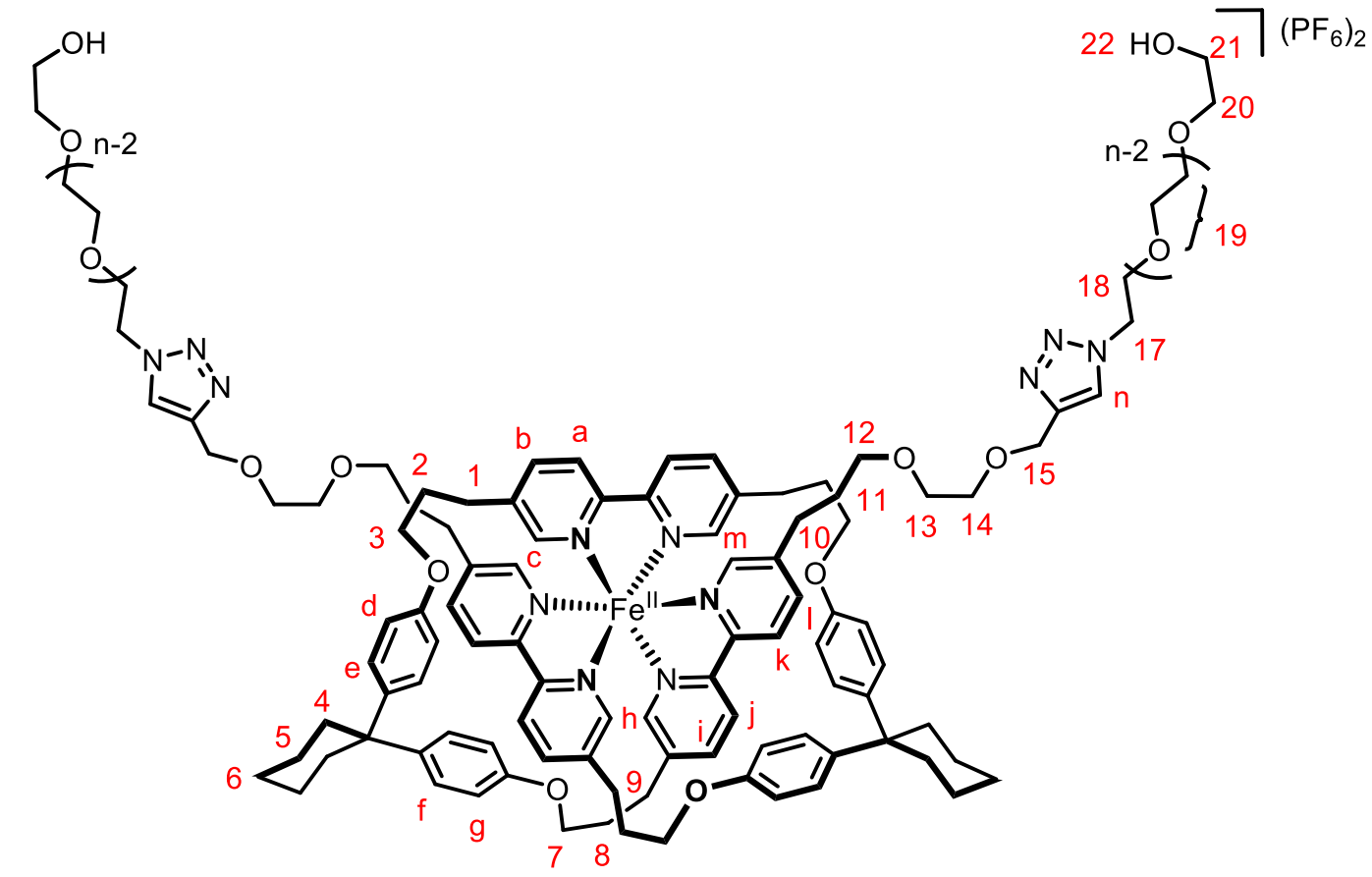

[Fe**L4**]$(PF_6)_2$

[Fe**L1**]$(PF_6)_2$ (130 mg, 73.7 µmol), **S33** (120 mg, 184.2 µmol), $Cu(MeCN)_4{\cdot}PF_6$ (68.5 mg, 184.2 µmol) and TBTA (98 mg, 184.2 µmol) were dissolved in the mixture solvent of MeCN (10 mL), MeOH (10 mL), THF (20 mL) in a sealed vial in N_2 atmosphere. The reaction mixture was stirred at 50 °C for 48 hours. After completion, the reaction mixture was cooled to room temperature and removed the solvent under vacuum. The crude product was purified by beads to give [Fe**L4**]$(PF_6)_2$ as red solid (217 mg, yield 96%).

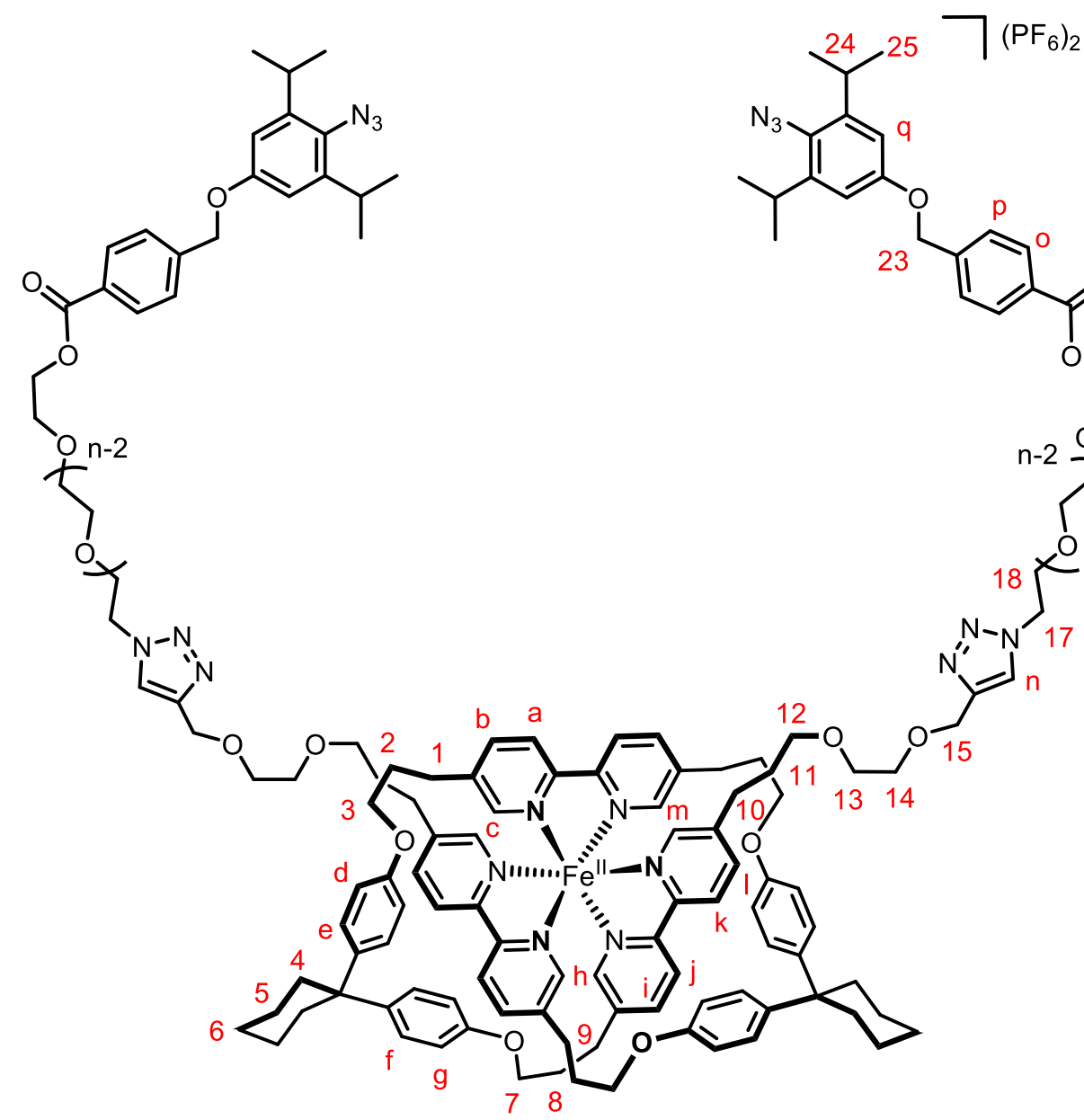

[Fe**7**]$(PF_6)_2$

[Fe**L4**]$(PF_6)_2$ (200 mg, 65.2 µmol), **S24** (270 mg, 390.9 µmol), and DMAP (23.9 mg, 195.4 µmol) were dissolved in anhydrous DCM (10 mL) in a 100 mL round bottom flask in N_2 atmosphere. The reaction mixture was stirred at room temperature for 4 days. After completion, the mixture was concentrated under vacuum and the crude product was purified by beads to give [Fe**7**]$(PF_6)_2$ as red solid (230 mg, yield 94%).

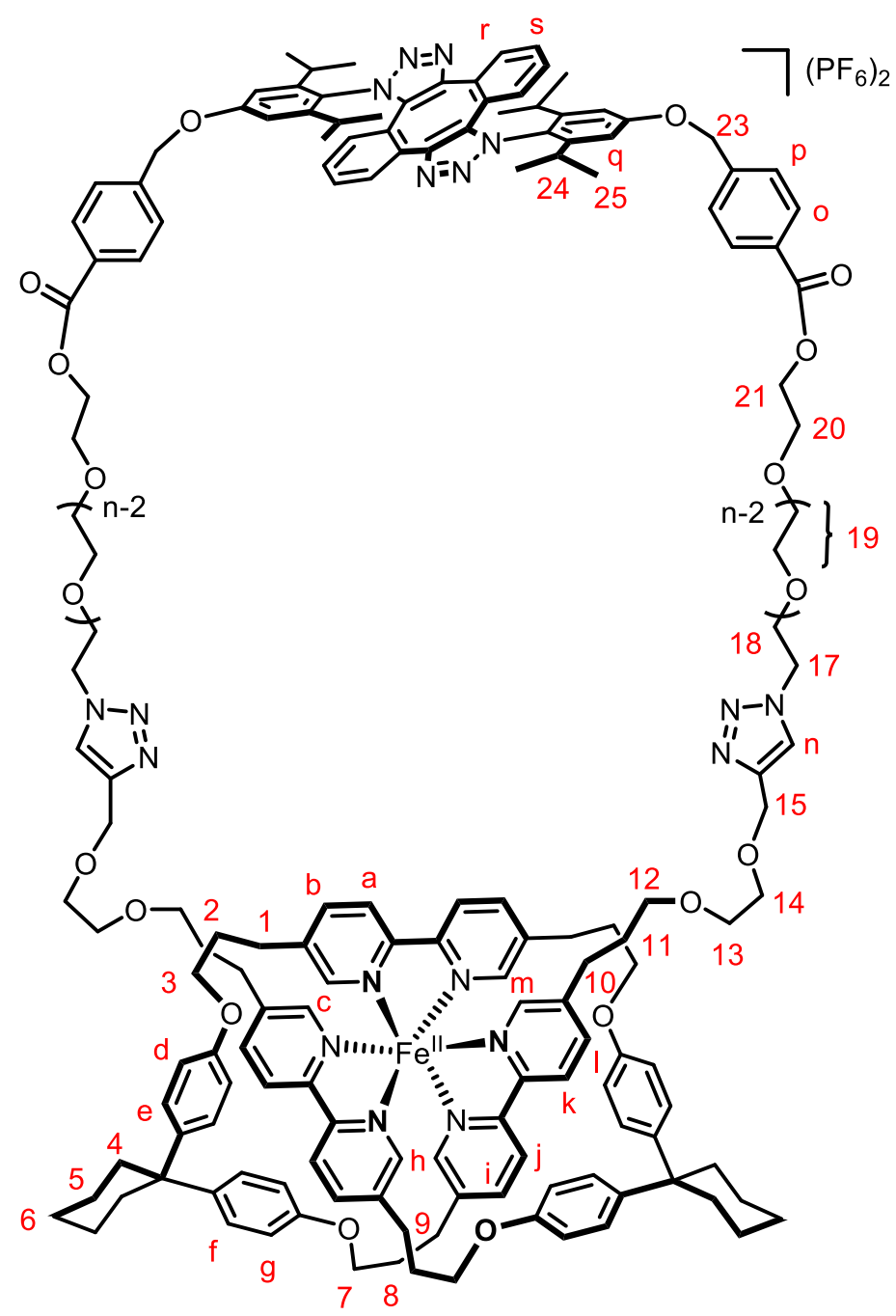

[Fe**8**]$(PF_6)_2$

To a stirred solution of **S23** (107 mg, 535.9 μmol) in THF (150 mL) was added the solution of [Fe**7**]$(PF_6)_2$ (100 mg, 26.8 μmol) in THF (50 mL) dropwise for 24 hours and then the reaction mixture was stirred for another 5 hours. After completion, the mixture was concentrated under vacuum and the crude product was purified by beads to give [Fe**8**]$(PF_6)_2$ as pink solid (103 mg, crude yield: 98%, contain some higher molecular weight by-products which formation by the oligomerization of [Fe**7**]$(PF_6)_2$ and **S23**).

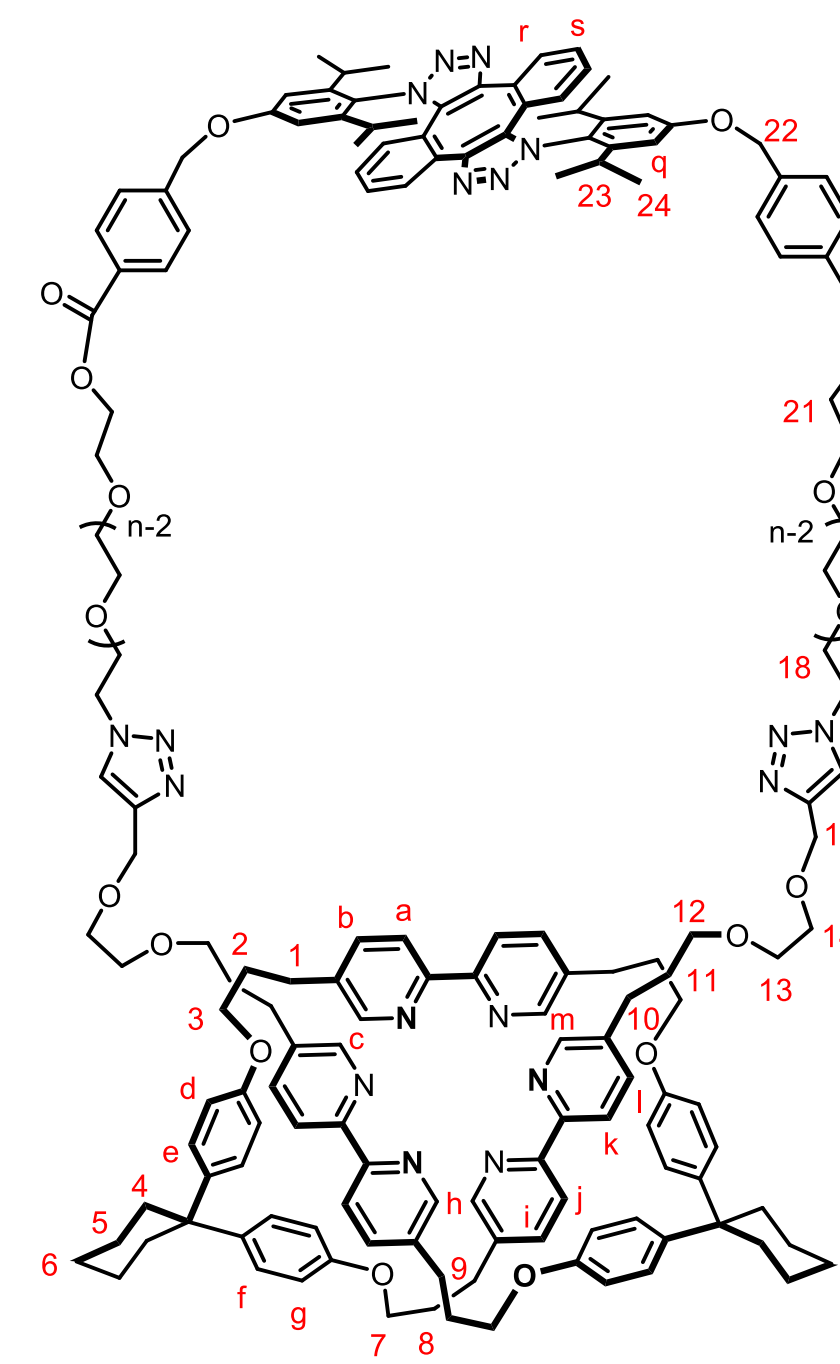

8

TBAF (153 µL, 153 µmol, 1 M in THF) was added to the solution of [Fe**8**]$(PF_6)_2$ (60 mg, 15.3 µmol) in THF (3 mL). The reaction mixture was stirred at room temperature for 5 mins. After completion, the crude product was firstly purified by beads, and then further purified by preparative GPC to give **8** as waxy solid (36 mg, yield 60%, $Đ$ = 1.06).

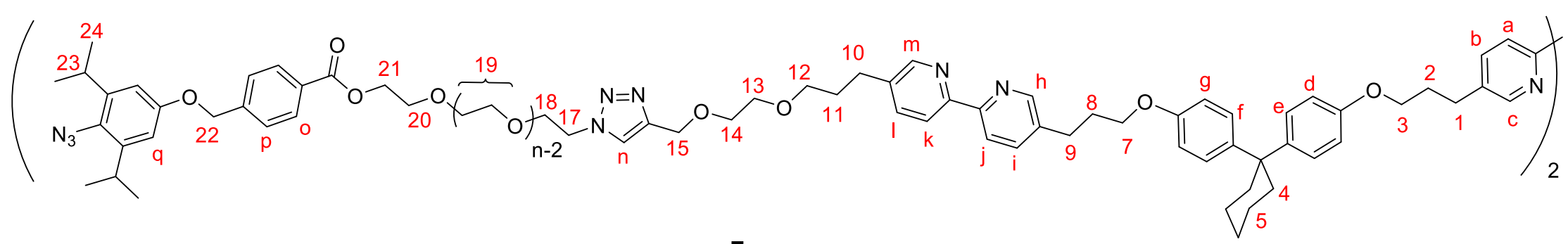

7

TBAF (107 µL, 107 µmol, 1 M in THF) was added to the solution of [Fe**7**]$(PF_6)_2$ (40 mg, 10.7 µmol) in THF (2 mL). The reaction mixture was stirred at room temperature for 5 mins. After completion, the crude product was firstly purified by beads, and then further purified by preparative GPC to give **7** as waxy solid (31 mg, yield 86%, M_n = 3763 Da, $Đ$ = 1.05).

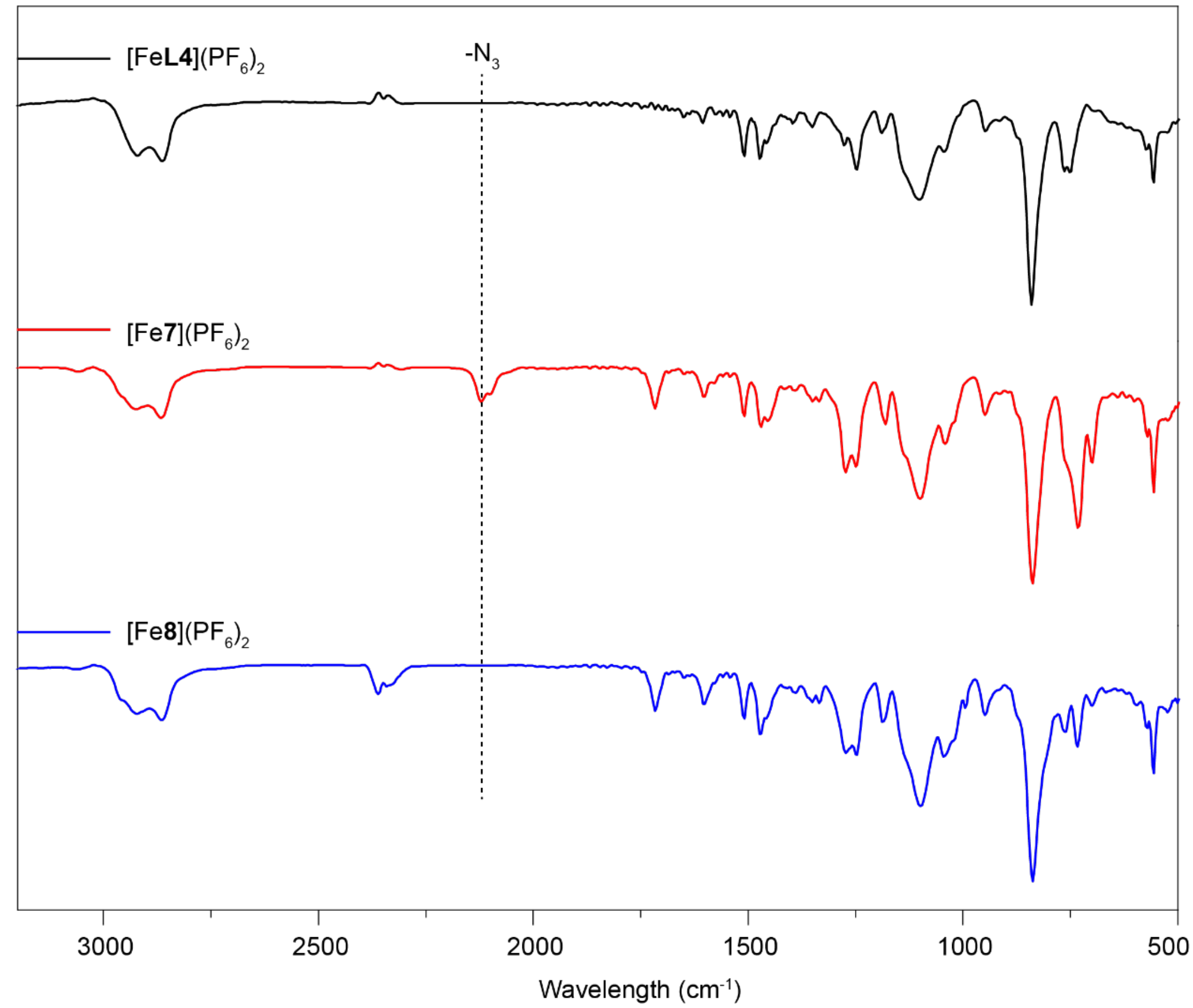

Figure 90. IR spectra of [Fe**L4**](PF_6)$_2$, [Fe**7**](PF_6)$_2$, and [Fe**8**](PF_6)$_2$. The appearance and disappearance of the characteristic peak of -N_3 at 2122 cm^{-1} prove the successful introduce of terminal -N_3 in [Fe**7**](PF_6)$_2$ and thoroughly consumed the -N_3 group when prepared [Fe**8**](PF_6)$_2$.

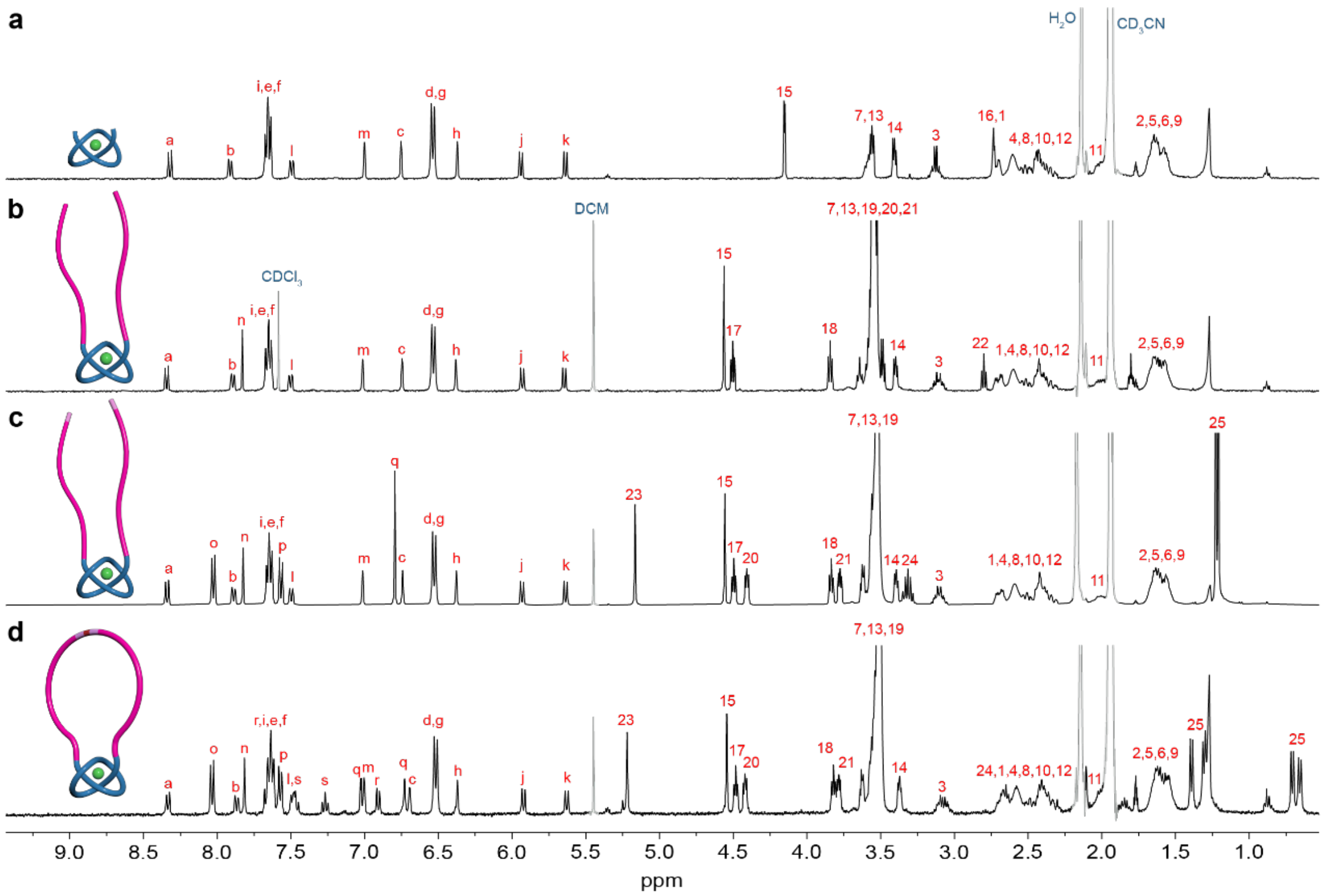

Figure 91. ^{1}H NMR spectra (400 MHz, MeCN-d_3 or MeCN-d_3: $CDCl_3$ = 5: 1, 298 K) of (a) [Fe**L1**]$(PF_6)_2$; (b) [Fe**L4**]$(PF_6)_2$; (c) [Fe**7**]$(PF_6)_2$; (d) [Fe**8**]$(PF_6)_2$. $CDCl_3$ was added to increase the solubility of the polymers.

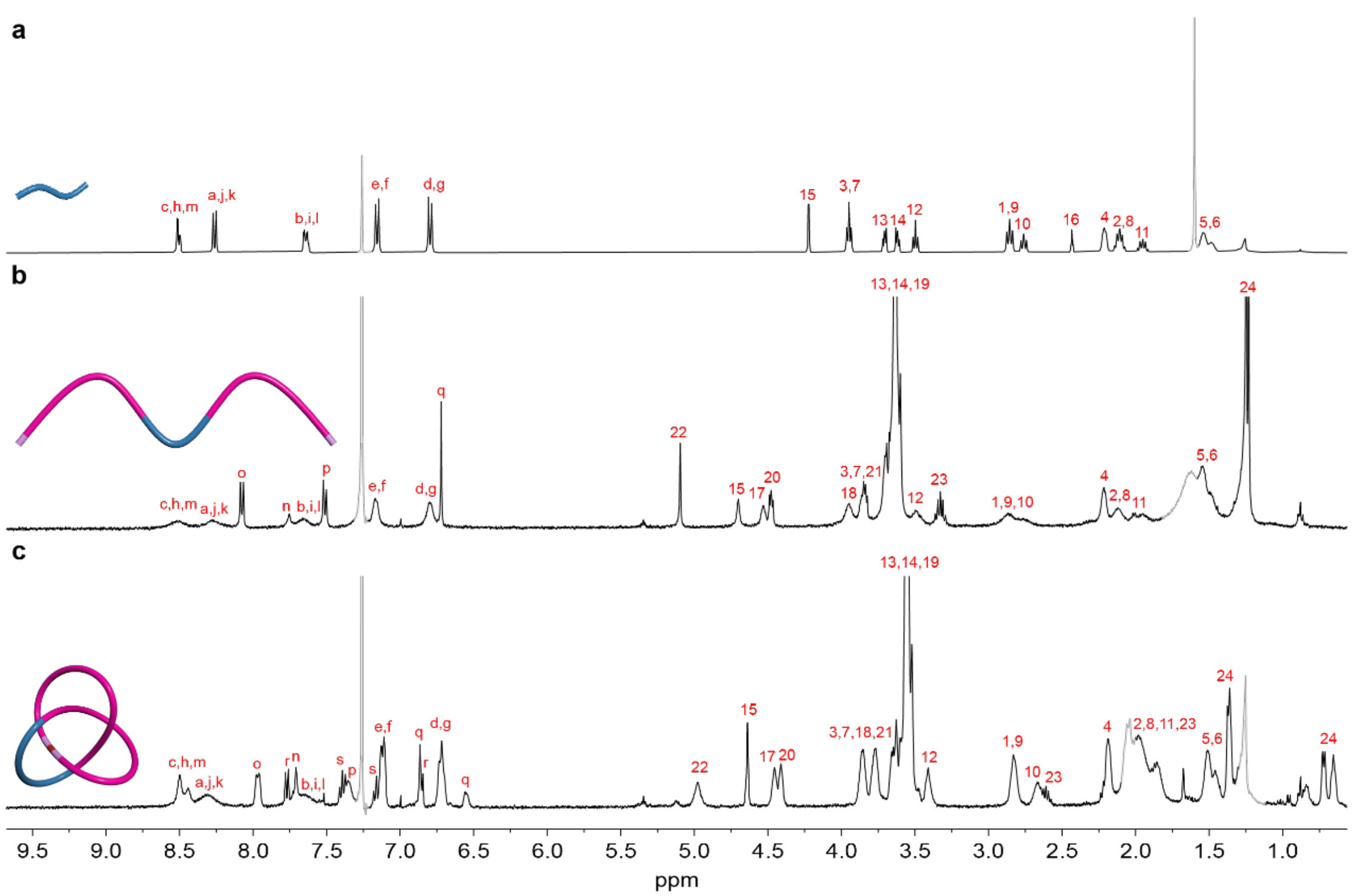

Figure 92. ^{1}H NMR spectra (400 MHz, $CDCl_3$, 298 K) of (a) **L1**; (b) **7**; (c) **8**.

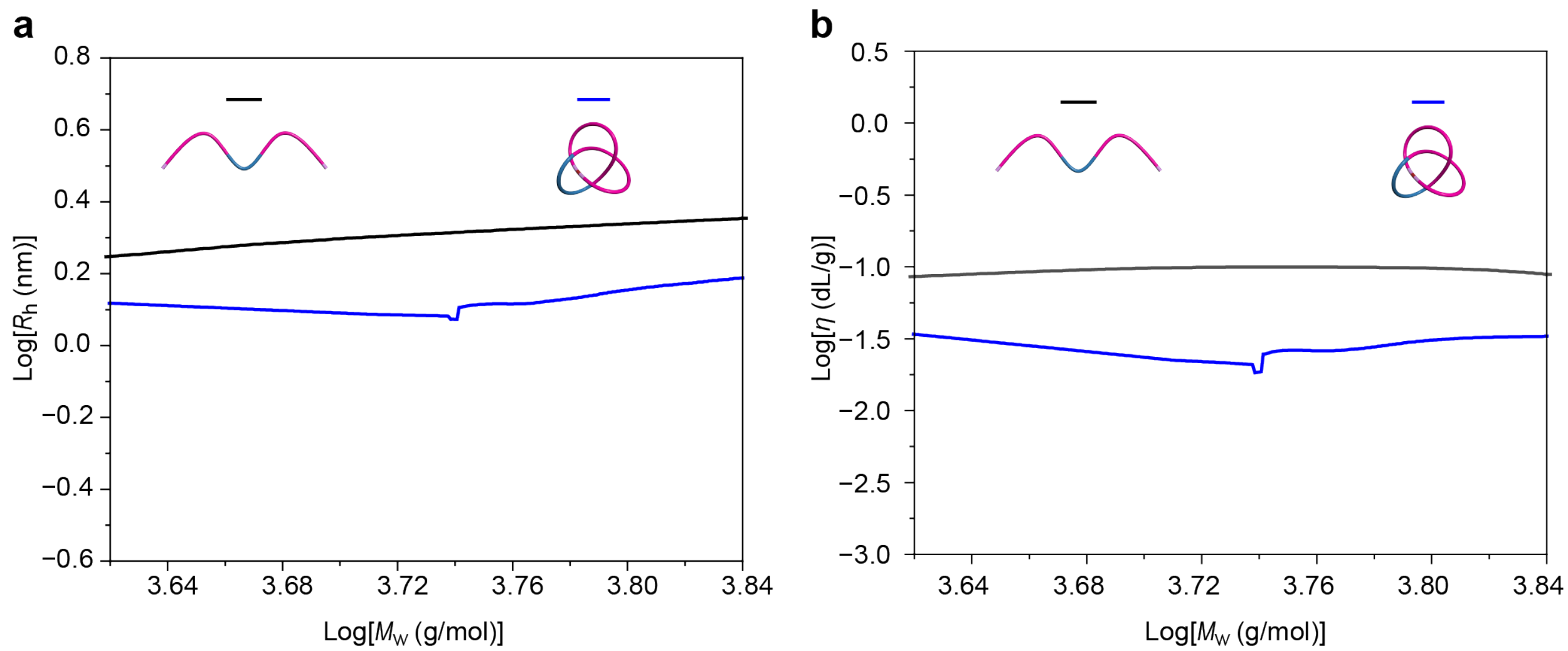

Figure 93. (a) Logarithm of [R_h] versus the logarithm of molar mass; (b) Logarithm of [η] versus the logarithm of molar mass of **7** and **8**.

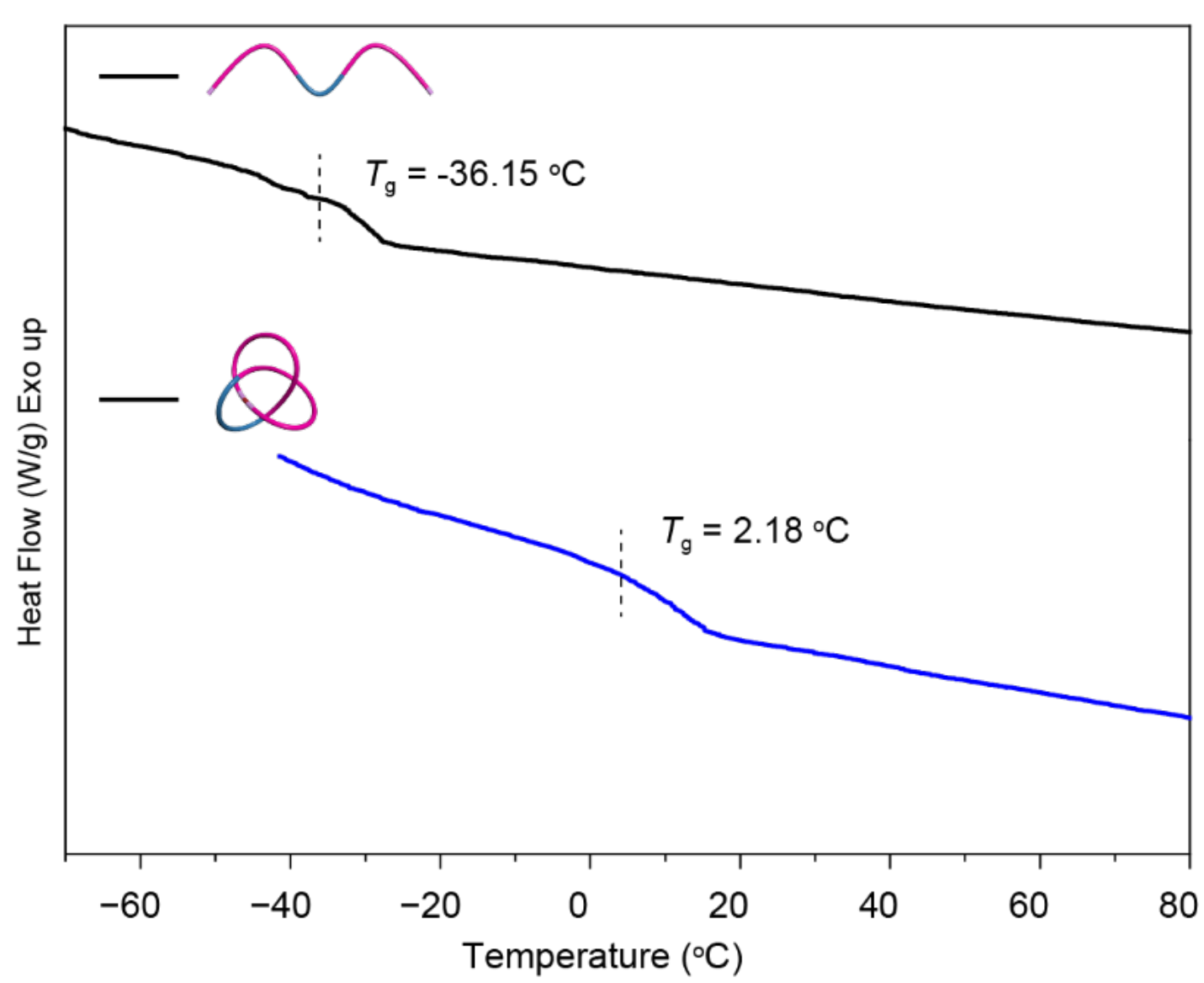

Figure 94. Differential scanning calorimetry (DSC) curves (T_g were determined by onset temperatures) of **7** and **8**.

12.3 Synthesis of linear (9) and trefoiled (10) diblock PS-PEG polymers

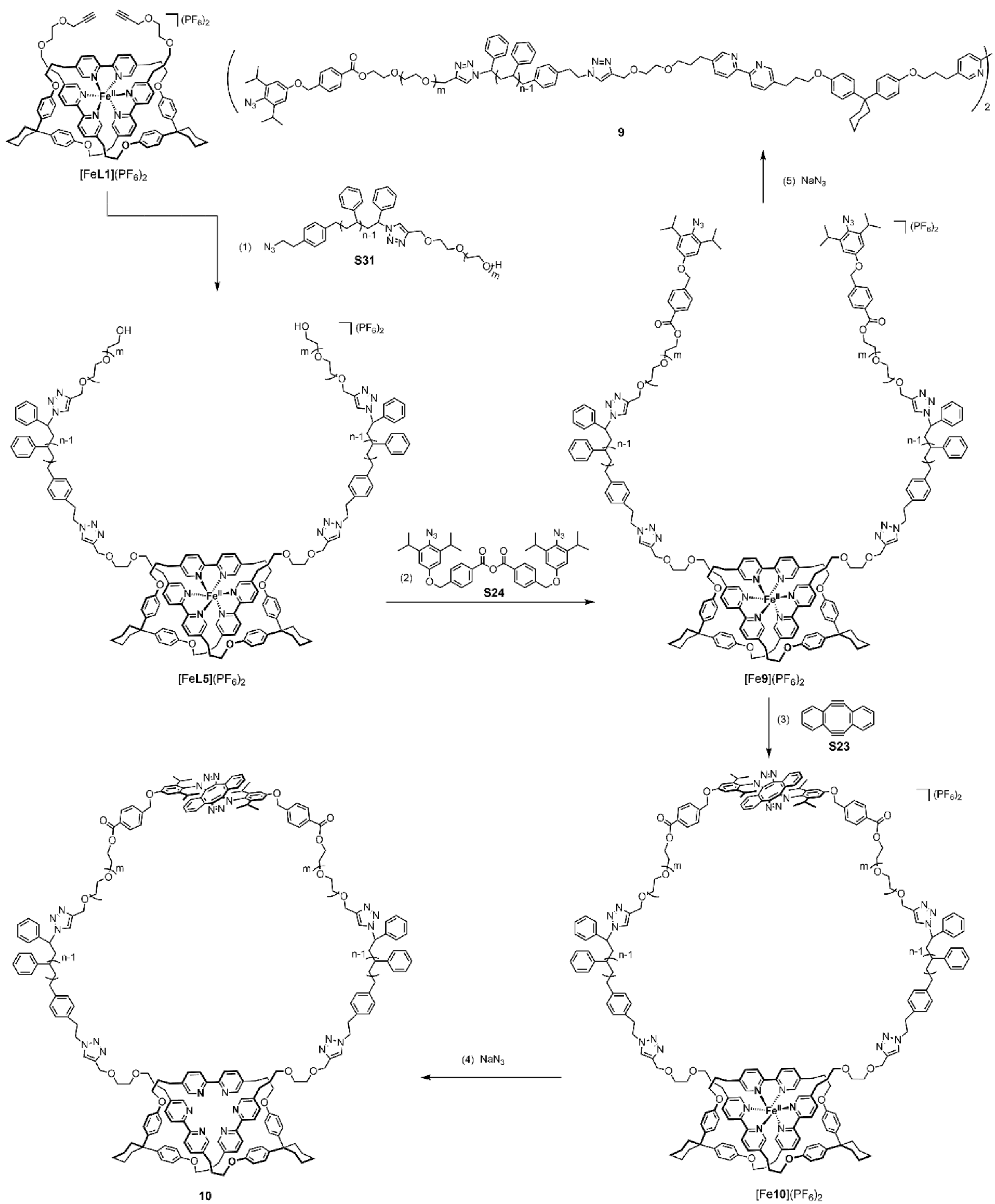

Figure 95. Synthesis of linear (**9**) and trefoiled (**10**) **PS-PEG** polymers.

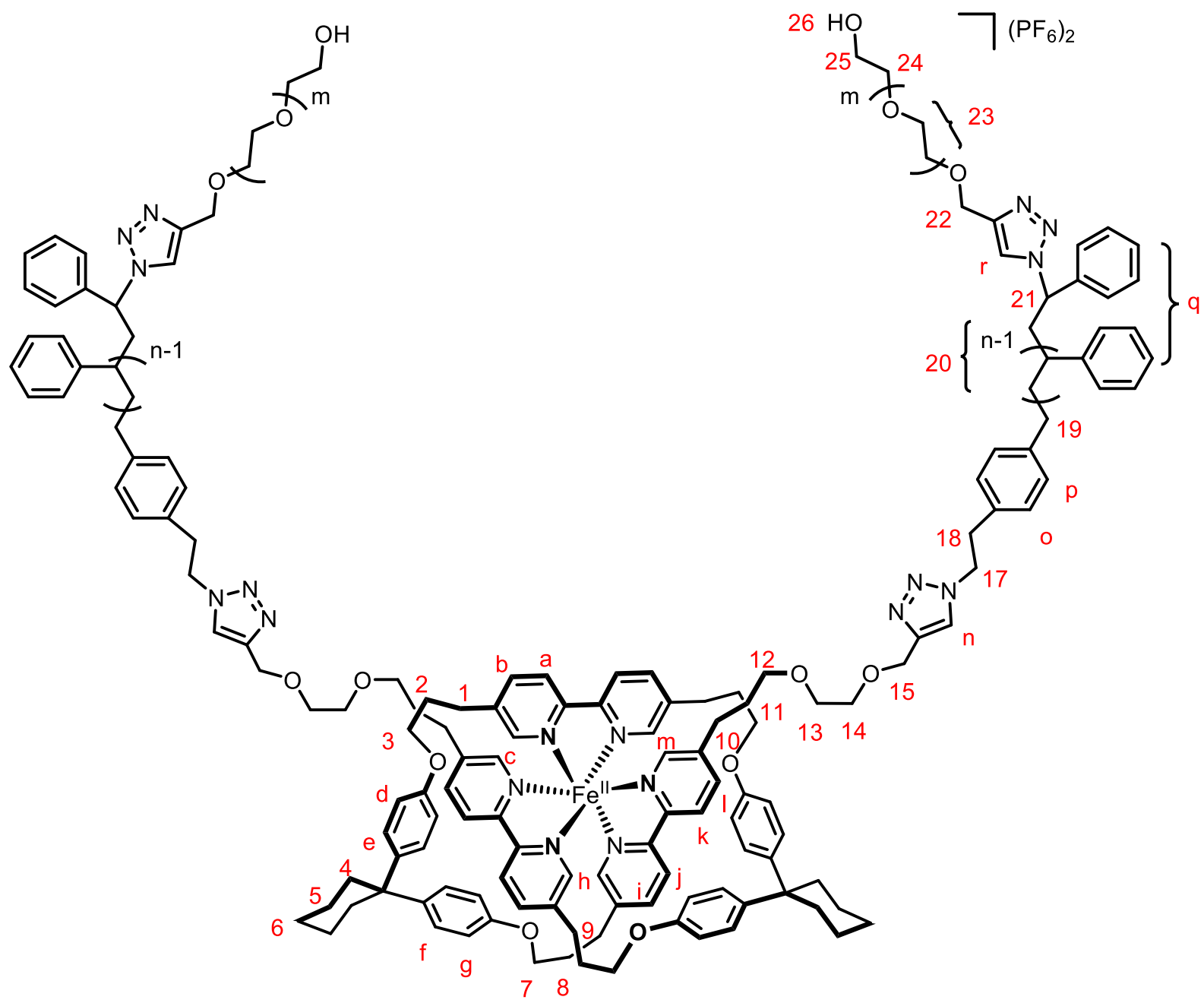

[Fe**L5**]$(PF_6)_2$

[Fe**L1**]$(PF_6)_2$ (42 mg, 23.8 µmol), **S31** (220 mg, 59.5 µmol), $Cu(MeCN)_4\cdot PF_6$ (22 mg, 59.5 µmol) and TBTA (32 mg, 59.5 µmol) were dissolved in the mixture solvent of MeCN (8 mL), MeOH (8 mL), THF (16 mL) in a sealed vial in N_2 atmosphere. The reaction mixture was stirred at 50 °C for 48 hours. After completion, the reaction mixture was cooled to room temperature and removed the solvent under vacuum. The crude product was purified by beads to give [Fe**L5**]$(PF_6)_2$ as red solid (207 mg, yield 95%).

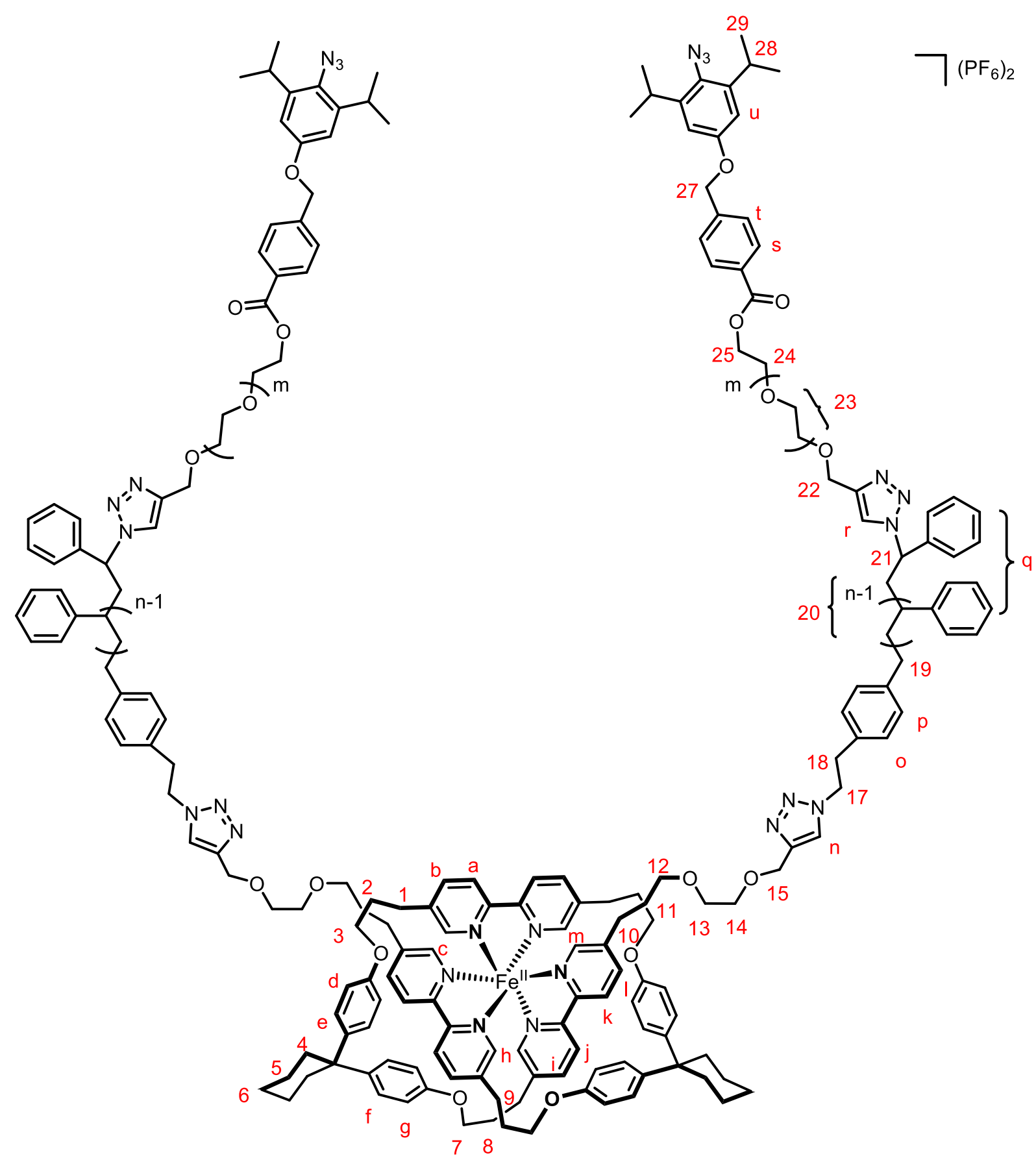

[Fe**9**]$(PF_6)_2$

[Fe**L5**]$(PF_6)_2$ (200 mg, 21.8 µmol), **S24** (90 mg, 130.6 µmol), and DMAP (8.0 mg, 65.3 µmol) were dissolved in anhydrous DCM (10 mL) in a 100 mL round bottom flask in N_2 atmosphere. The reaction mixture was stirred at room temperature for 4 days. After completion, the mixture was concentrated under vacuum and the crude product was purified by beads to give [Fe**9**]$(PF_6)_2$ as red solid (200 mg, yield 93%).

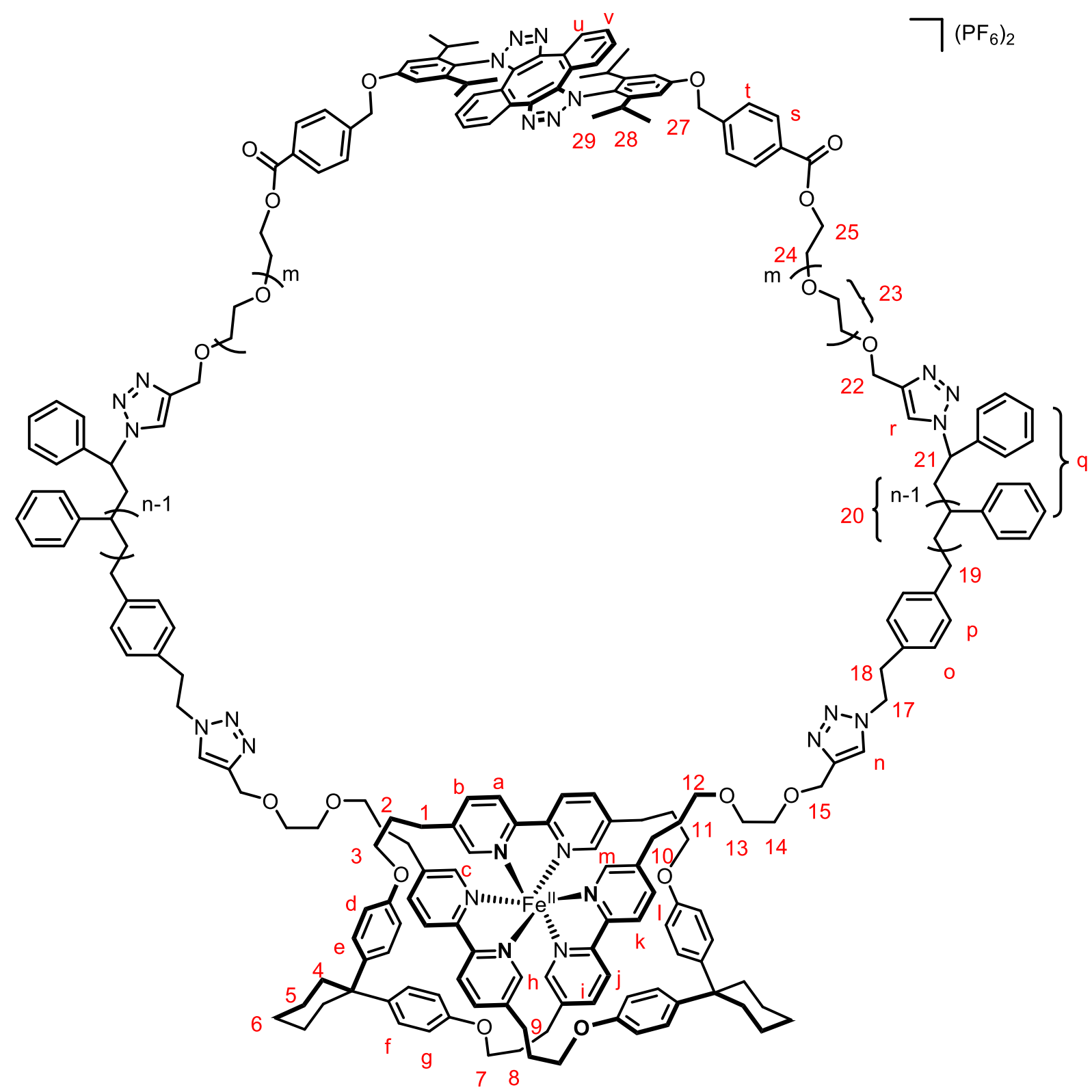

[Fe**10**](PF_6)$_2$

To a stirred solution of **S23** (41.5 mg, 20.7 μmol) in THF (60 mL) was added the solution of [Fe**9**](PF_6)$_2$ (100 mg, 10.4 μmol) in THF (20 mL) dropwise for 24 hours and then the reaction mixture was stirred for another 5 hours. After completion, the mixture was concentrated under vacuum and the crude product was purified by beads to give [Fe**10**](PF_6)$_2$ as pink solid (99 mg, crude yield: 97%, could contain some higher molecular weight by-product which formation by the oligomerization of [Fe**9**](PF_6)$_2$ and **S23**).

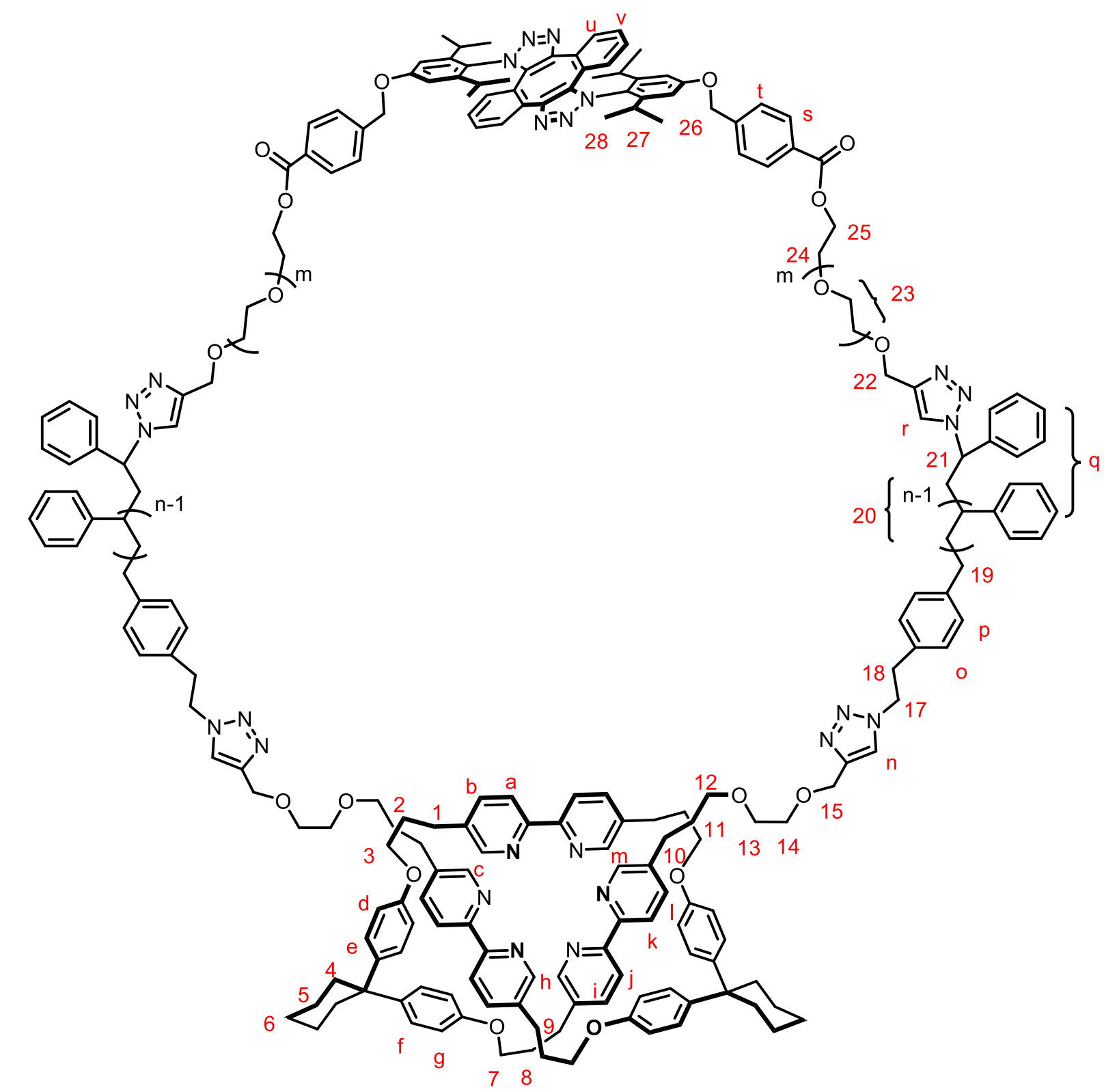

10

TBAF (81 µL, 81 µmol, 1 M in THF) was added to the solution of [Fe**10**](PF_6)$_2$ (80 mg, 8.1 µmol) in THF (3 mL). The reaction mixture was stirred at room temperature for 5 mins. After completion, the crude product was firstly purified by beads, and then further purified by preparative GPC to give **10** as white solid (55 mg, yield 69%, $Đ$ = 1.07).

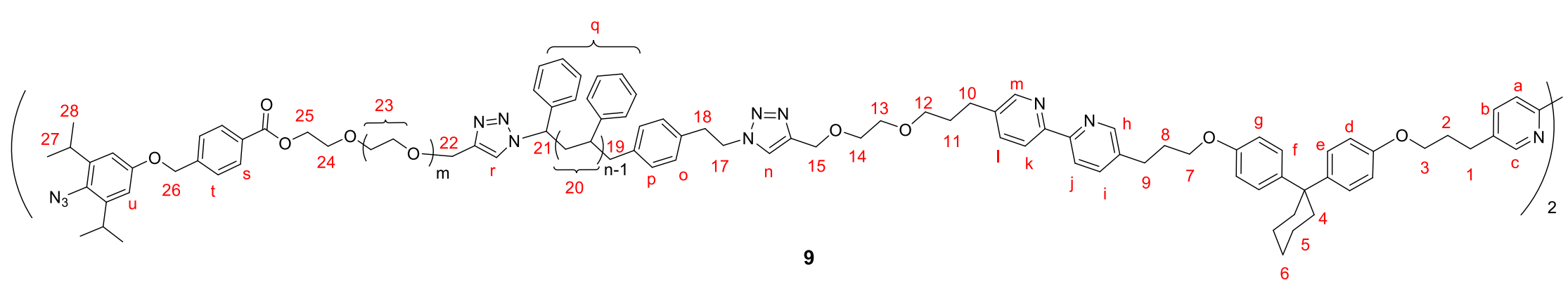

9

TBAF (31 µL, 31 µmol, 1 M in THF) was added to the solution of [Fe**9**](PF_6)$_2$ (30 mg, 3.1 µmol) in THF (2 mL). The reaction mixture was stirred at room temperature for 5 mins. After completion, the crude product was firstly purified by beads, and then further purified by preparative GPC to give **9** as white solid (24 mg, yield 82%, M_n = 10600 Da, $Đ$ = 1.05,).

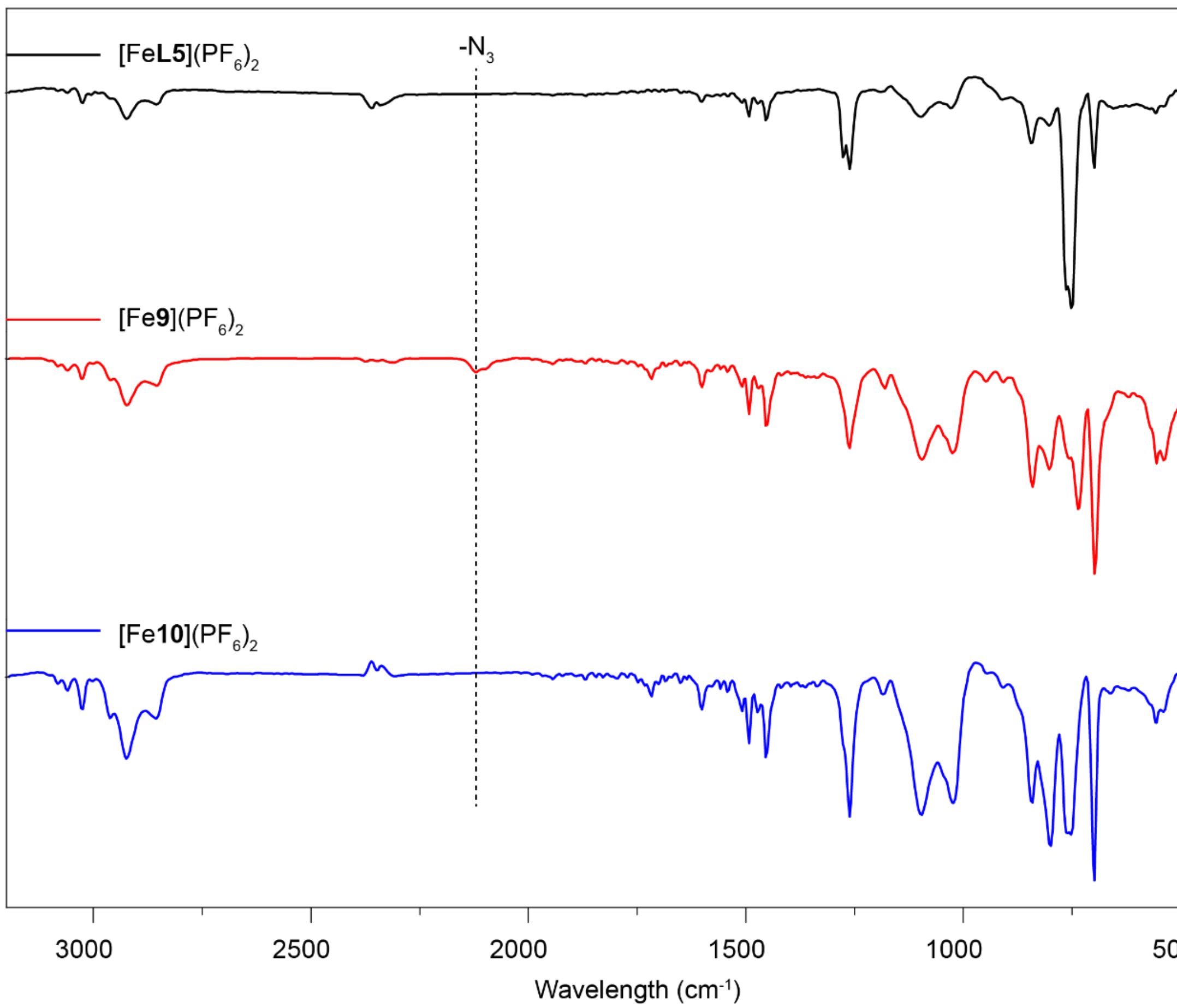

Figure 96. IR spectra of [Fe**L5**](PF_6)$_2$, [Fe**9**](PF_6)$_2$, and [Fe**10**](PF_6)$_2$. The appearance and disappearance of the characteristic peak of -N_3 at 2122 cm^{-1} prove the successful introduce of terminal -N_3 in [Fe**9**](PF_6)$_2$ and thoroughly consumed the -N_3 group when prepared [Fe**10**](PF_6)$_2$.

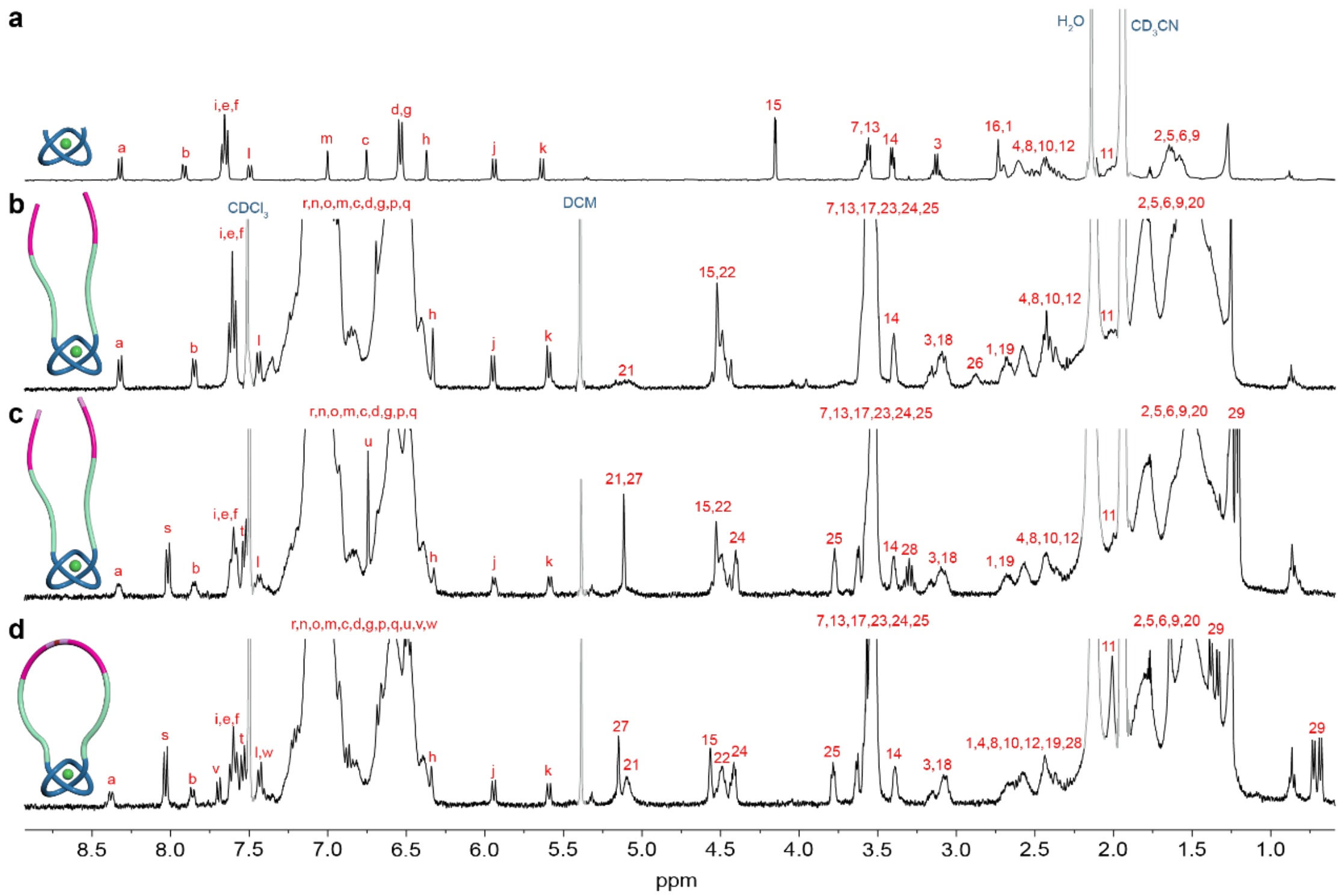

Figure 97. ^{1}H NMR spectra (400 MHz, MeCN-d_3 or MeCN-d_3: $CDCl_3$ = 5: 1, 298 K) of (a) [Fe**L1**]$(PF_6)_2$; (b) [Fe**L5**]$(PF_6)_2$; (c) [Fe**9**]$(PF_6)_2$; (d) [Fe**10**]$(PF_6)_2$. $CDCl_3$ was added to increase the solubility of the polymers.

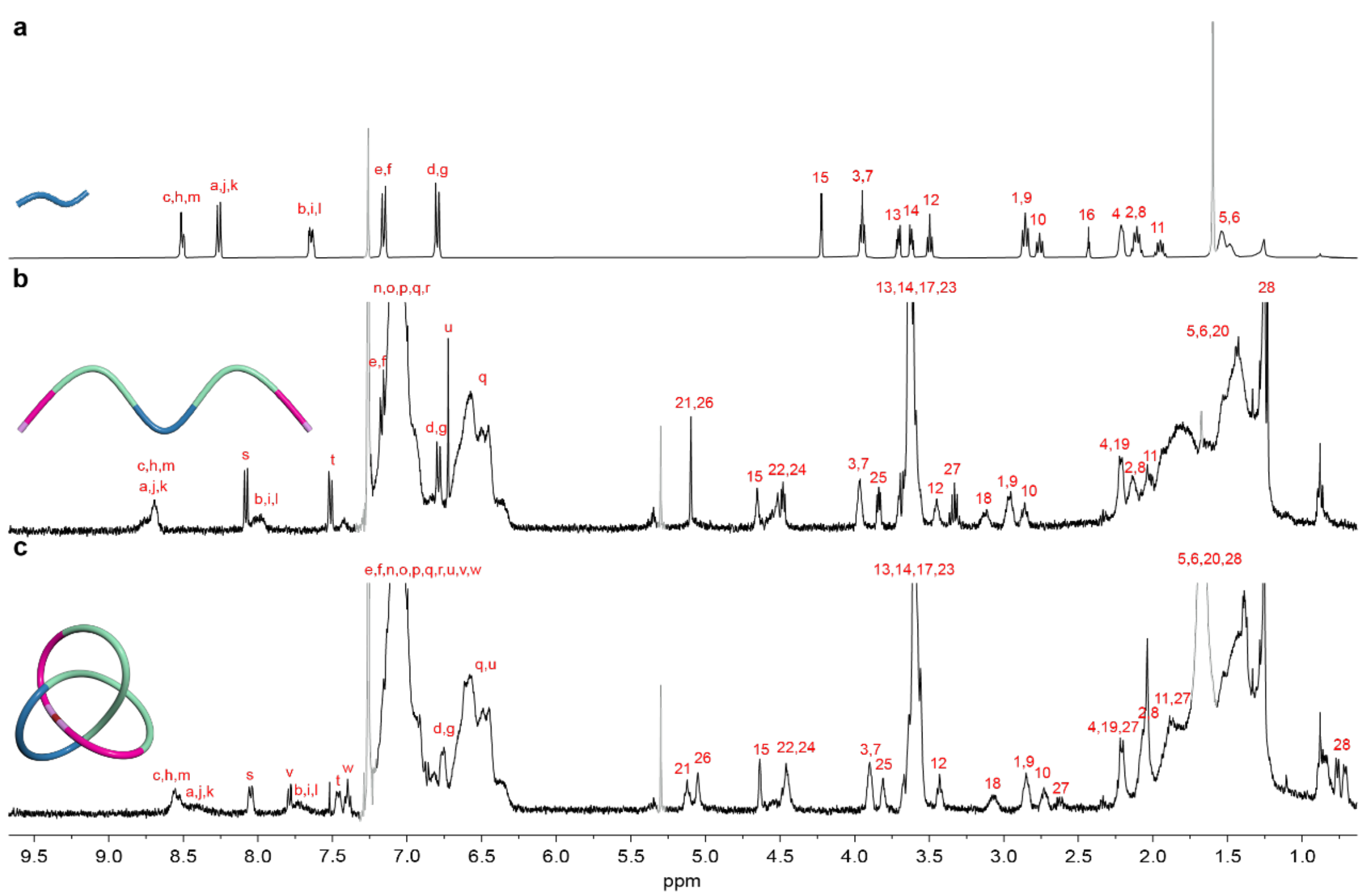

Figure 98. ^{1}H NMR spectra (400 MHz, $CDCl_3$, 298 K) of (a) **L1**; (b) **9**; (c) **10**.

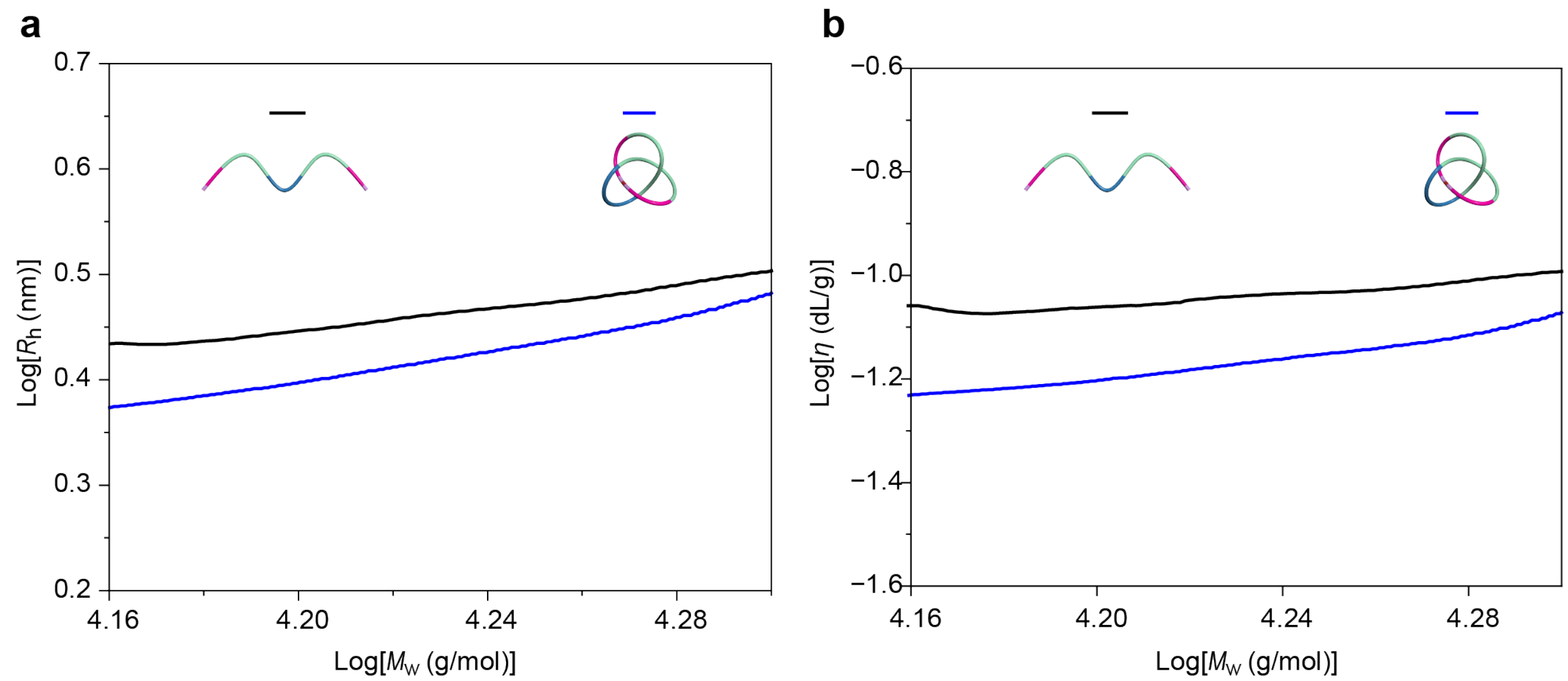

Figure 99. (a) Logarithm of [R_h] versus the logarithm of molar mass; (b) Logarithm of [η] versus the logarithm of molar mass of **9** and **10**.

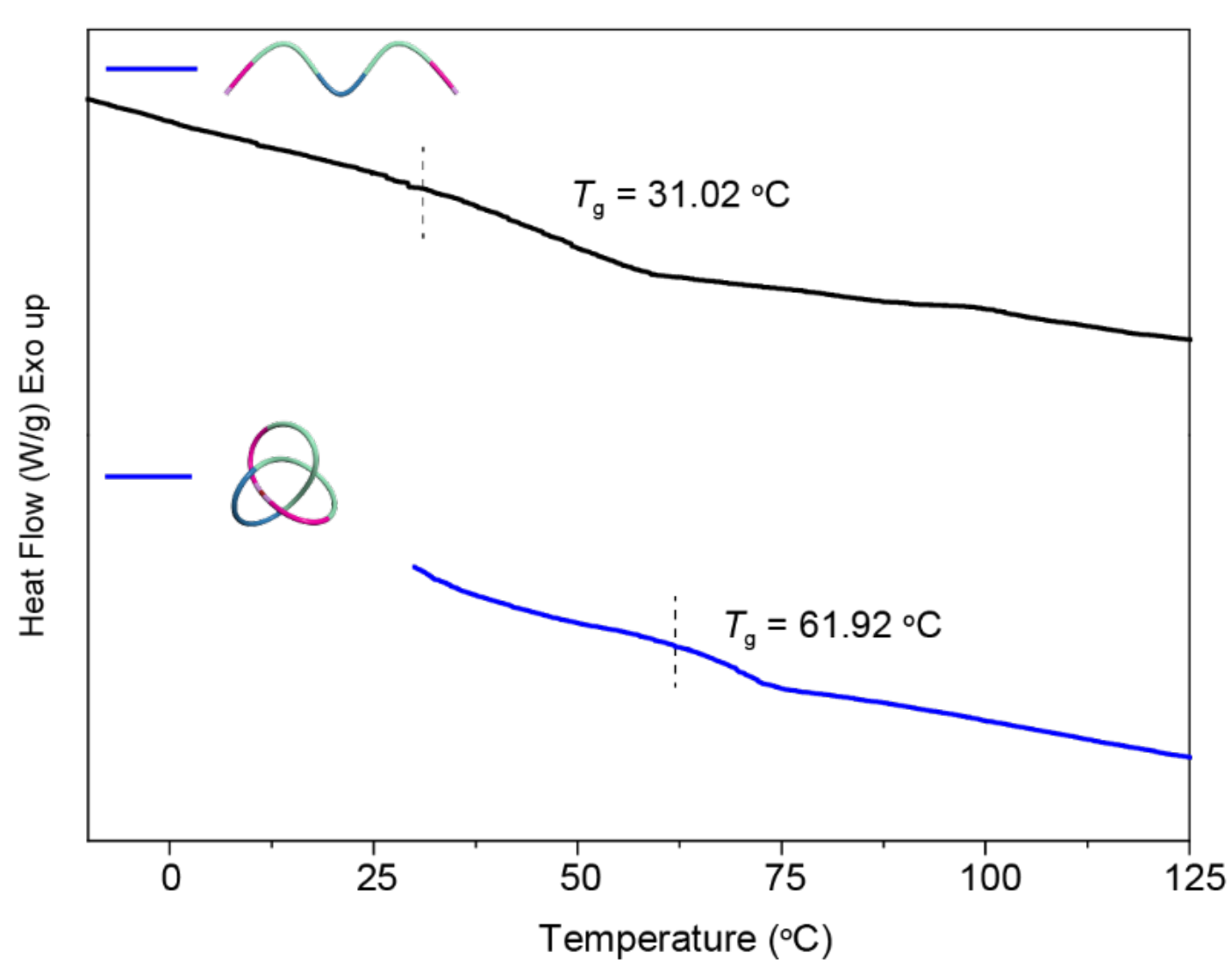

Figure 100. Differential scanning calorimetry (DSC) curves (T_g were determined by onset temperatures) of **9** and **10**.

13. Conceptual illustrations highlighting the potential of the topological knot synthon strategy to engineer topologically complex polymers

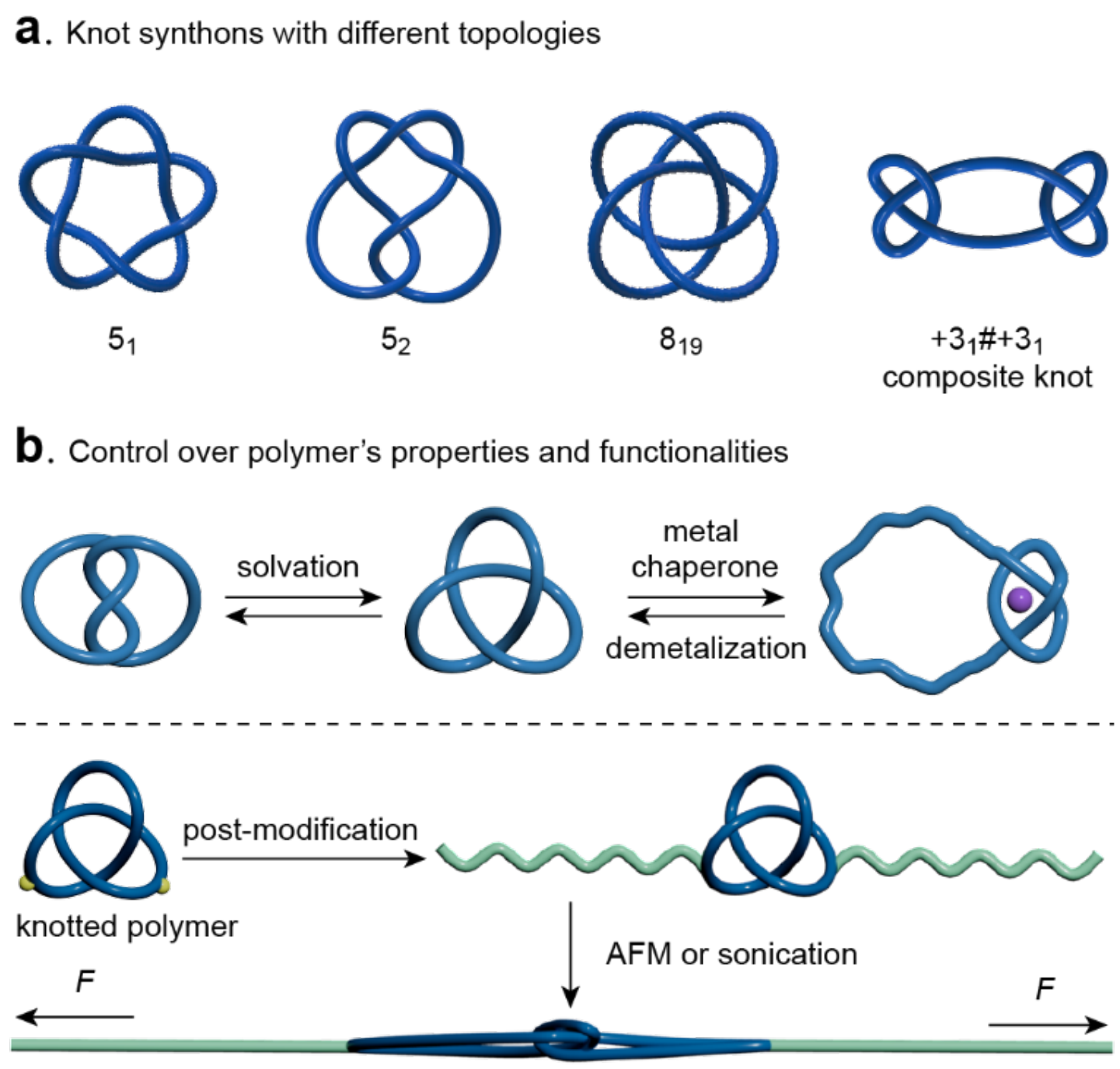

Figure 101. (a) Knot synthon strategy can serve as a transformative platform for constructing polymers with well-defined knotted topologies. (b) Use of metal chaperones allows precise initial or subsequent knot location within the polymer chain, enabling tuning of polymer properties and the introduction of other functionality. This could aid the investigation of the mechanical properties of polymeric knots, aiding understanding of how topological features influence a material's overall behavior and performance.[12,13]

14. References

1 Zhang, L., Wu, Y., Li, S., Zhang, Y. & Zhang, K. Scalable bimolecular ring-closure method for cyclic polymers. *Macromolecules* **53**, 8621–8630 (2020).

2 Sun, P., Chen, J., Liu, J. & Zhang, K. Self-accelerating click reaction for cyclic polymer. *Macromolecules* **50**, 1463–1472 (2017).

3 Dai, L. & Doyle, P. S. Universal knot spectra for confined polymers. *Macromolecules* **51**, 6327–6333 (2018).

4 Dai, L., Soh, B. W. & Doyle, P. S. Effects of side chains on polymer knots. *Macromolecules* **52**, 6792–6800 (2019).

5 Zhu, H., Tian, F., Sun, L., Zhu, Y., Qiu, Q. & Dai, L. Computational design of extraordinarily stable peptide structures through side-chain-locked knots. *J. Phys. Chem. Lett.* **13**, 7741–7748 (2022).

6 Metzler, R., Hanke, A., Dommersnes, P. G., Kantor Y. & Kardar, M. Equilibrium shapes of flat knots, *Phys. Rev. Lett.* **88**, 188101 (2002).

7 Marcone, B., Orlandini, E. & Stella, A. L. Knot localization in adsorbing polymer rings, *Phys. Rev. E* **76**, 051804 (2007).

8 Grosberg, A. Y. & Rabin, Y. Metastable tight knots in a wormlike polymer, *Phys. Rev. Lett.* **99**, 217801 (2007).

9 Tubiana, L. Computational study on the progressive factorization of composite polymer knots into separated prime components, *Phys. Rev. E* **89**, 052602 (2014).

10 Dai, L., Renner, B. & Doyle, P. S. Metastable tight knots in semiflexible chains, *Macromolecules* **47**, 6135−6140 (2014).

11 Dai, L., Renner, C. B. & Doyle, P. S. Origin of metastable knots in single flexible chains, *Phys. Rev. Lett.* **114**, 037801 (2015).

12 Marcos, V. et al. Allosteric initiation and regulation of catalysis with a molecular knot. *Science* **352**, 1555–1559 (2016).

13 Christian, T. et al. Methyl transfer by substrate signaling from a knotted protein fold. *Nat. Struct. Mol. Biol.* **23**, 941–948 (2016).
