# Supplementary material for: Trefoil polymers from a knotted synthon
Source: Nat Chem. 2026 Jun 29;18(10):1708–16. doi: 10.1038/s41557-026-02197-4 (PMC13623649; doi:10.1038/s41557-026-02197-4)
Supplement: Supplementary file 2 [file 41557_2026_2197_MOESM2_ESM.pdf]

nature portfolio

Peer Review File

Trefoil polymers from a knotted synthon

Corresponding Author: Professor David Leigh

This file contains all reviewer reports in order by version, followed by all author rebuttals in order by version.

Version 0:

Reviewer comments:

Reviewer #1

(Comments for the Author)
The manuscript by Dai, Li, Leigh, Zhang et al describes the synthesis of trefoil knots and their macrocyclic topological isomers, their visualisation on STM and incorporation in block copolymers. The experimental data is backed by high level characterisation of all the building blocks and coarse-grind simulation. The manuscript is well written and the experimental data matches conclusions drawn by the authors. I would recommend the following points to be addressed before publications:

1. There are a few typos that can be quickly ironed out by a spell check ("reptation", "iamges", etc)
2. The STM assignment of the metallated trefoil twist and the DIBOD is based on size only. Could the authors get XPS data to support their assignment, especially for the metallated trefoil?
3. The ESI is very well put together but for consistency the authors should check the numbering and proton assignment for S7 and the precision with which they report the diffusion coefficients determined by DOSY.

Reviewer #2

(Comments for the Author)
Summary
Du et al. present an extremely thorough study of the effects of topology on properties of polymers. They synthesize compare linear, cyclic, and trefoil-knotted polystyrene (PS) through many means, such as: MALDI-TOF mass spectrometry, hydrodynamic volume and viscosity, hydrodynamic radius, differential scanning calorimetry, and 1H NMR spectroscopy. They repeat these experiments on two different length polymers for all three topologies. Furthermore, they also prepare and test polyethylene glycol polymers (PEG) and mixed PS-PEG block copolymers. One of the most compelling aspects of the manuscript is the visualization of the PS polymers by ultrahigh vacuum scanning tunneling microscopy. Here they find the knotted structures fall into three conformations: high-, medium-, and low-symmetry. The solvent used to prepare the Ag(111) surface influences the distributions of these three conformers. The authors then model these using coarse-grained Langevin dynamics simulations. The modeling provided interesting insights and was consistent with the author’s interpretation of the forces involved in determining the relative ratio of the conformers.

Validity
Du et al. present exhaustive characterization of the synthetic intermediates and the polymers.

Significance
There have been other theoretical and experimental studies of knotted polymers, but none that are as thorough as this work. Here the chemical composition of the linear, cyclic, and trefoil-knotted polymers are so similar and this is extremely important when drawing conclusions on the effects of topology on the properties. This study is fundamental in so many ways. The molecular topology community has been speculating on many of the issues for decades and this work has now experimentally addressed and resolved them.

Data and methodology; analytical approach
No issues.

Suggested Improvements

The PEG and PS-PEG polymers should be discussed more. I thought I accidentally misplaced a page of the manuscript because the synthesis of these are discussed, but the properties and how they articulate / compare with the PS polymer results is lacking.

The macrocyclization step should be discussed more in the manuscript. These are done under high dilution conditions (dropwise addition), but I was surprised about how good the yields are. Do the authors have an idea of the effective concentration under their macrocyclization conditions and how fast the click reaction is under those conditions? The SI states the the product “could contain some higher molecular weight by-product which formation [sic] by the oligomerization of …” How did the authors evaluate how much of this by-product is present. I guess the disappearance of the azide signal in the IR supports that it is not much, but was this by-product detected by mass spec or other means?

At the end of the conclusion there is a one-sentence discussion of how this work might be extended to 5_2 or 8_19 knot synthons. The SI also mentions this, but also shows a 5_1 and composite knot. It would be nice if the composite knot is mentioned in the manuscript as well and a sentence or two of how the current synthesis might be adapted to make the granny/square knot composite polymer.

References
These are appropriate.

Clarity and context
The manuscript is well-written and appropriate context is given.

I recommend publishing once the issues discussed above are addressed.

Reviewer #3

(Comments for the Author)
Summary of key results
In their manuscript “Trefoil polymers from a knotted synthon” Du et al propose a new strategy to synthesize knotted trefoil polymers from various backbones with high yield and low polydispersity. The technique, based on a knotted synthon to which different linear polymers can be attached through click-chemistry and later circularized, allows one to control the contour length of the final knotted polymer as well as to knot different polymers, like polystyrene (PS) or PEG. The authors further investigate the effect of polymer length on the properties of knotted PS, finding that chain length increases the probability of highly-symmetrical conformations, something rather unintuitive from the point of view of the computational and theoretical studies of knotted homopolymers. The results are further reinforced by simulations of a coarse-grained model that mimics some properties of the real system.

Originality and significance
I found some minor issues with the manuscript, regarding some missing connections to the existing theory, the statement that the knot “translates away from the synthon”, and some aspects of the simulation part, which I believe could be extended and should be better explained. That notwithstanding, I think that this is a fascinating, seminal study that will help the community studying knotted polymers moving forward. In particular, this work paves the way for a more detailed characterization of knots in polymers far from their scaling limit and will be a huge help and motivator also for computational polymer physicists to study such systems. I highly recommend publication after the authors have tackled my, mostly minor, comments.

Comments:
This section covers Data & methodology, appropriate use of statistics, suggested improvements, references, following the order of the manuscript.

1. Fig 1. I guess that 2n indicates the number of monomers in both PS, but it should be specified.

2. Page 3 The authors write “While theory suggests that strand length31,32 should influence the degree that a knot is confined to a particular region of a polymer strand, this has not previously been explored experimentally”. Here it is not completely clear to me what the authors mean. If by “particular region” they mean a specific region with higher or lower flexibility than the rest of the chain, as the references suggest, I agree with them. But this needs to be specified. Related to this, the fact that the synthon is more rigid than the rest of the chain should be stated explicitly: while for most chemists this might be easily discerned, computational physicists will have difficulties understanding it from the chemical structure (I guess the bipyridines add enough rigidity compared to PS). It seems to me that up to page 3 of the manuscript this difference in flexibility can be understood only from the sentence “(LF, defined as LF =2LPS/LL1; LPS: length of PS chain; LL1: length of ligand L1) for the two trefoiled PS polymers of 3.9 (2) and 2.8 (5)”, which implicitly proposes L1 as the most rigid part of the ring.

3. Page 3 “We therefore prepared a second set of linear (4), trefoiled (5), and cyclic (6) PSs using a PS chain with a lower molecular weight [...] which should produce a tighter tangle in the trefoiled polymer.” I am not sure where this conclusion comes from in the literature. If we consider flexible homopolymers, the knot length distribution always has the same peak, indicating that knot length grows simply due to fluctuations that produce larger knots in proportion to the contour length of the rings (Tubiana, L. (2014). Physical Review E, 89(5), 052602; Rieger, F. C., & Virnau, P. (2016). PLOS Computational Biology, 12(9), e1005029), which results in the length of the knot growing sublinearly with that of the polymer. The tangle

however could very well remain of the same size as soon as the knot becomes relatively localized, i.e. as soon as the knot occupies half of the chain or less. Theoretical analyses on semiflexible homopolymers argue that very long chains bring knot localization (Grosberg, A. Y., & Rabin, Y. (2007). Physical review letters, 99(21), 217801, ref 17 in the manuscript). In fact, the results reported in the rest of the paper are surprising because computational studies report that the highly symmetrical configuration of the trefoil is more compatible with shorter chain lengths (e.g. Katritch, V., Bednar, J., Michoud, D., Scharein, R. G., Dubochet, J., & Stasiak, A. (1996). Nature, 384(6605), 142-145).

4. Pages 6-7 “the knot does not adopt a single well-defined conformation but rather undergoes rapid reptation of the different sections of the strand through the region of entanglement” (bold font is mine). I agree that the chain certainly moves through the knot. Only, without knowing the rapidity of variation of the temperature in the VT-NMR, I find it difficult to understand how rapid this reptation is. Would it be possible to compare it against other dynamical times of the system? On a more technical note, the authors use the term reptation, but it could also be that the knot breathes enough for it to “move along” the chain in a mechanism that is different from reptation.

5. Page 7 The authors write: “The diffusion coefficient (D) of cyclic PS3 was higher than that of the linear analogue 1 across a range of solvents [...] indicating a smaller hydrodynamic radius (Rh) [...] In contrast, trefoil PS 2, the topology with the smallest Rh, showed similar or smaller D values in these solvents compared to 3.” I am a bit confused. From the first sentence, it seems that Rh is estimated from D, while for the second sentence to hold Rh must be measured independently of D. Please clarify how Rh is measured.

6. Page 7 “Theory suggests there will be influenced [...]” (bold is mine). Typo: there -> these.

7. Page 7 “solvation conditions and the presence of external forces such as temperature or shear” (bold is mine). Temperature is not a force.

8. Page 7 “the STM images show the knotted region to be much looser and translated away from the original synthon into the PS chains”. I agree that the knot is much looser, but I am not sure whether the data from STM show that the knot has translated away from the synthon, and I would like the authors to clarify this. To establish the position of the knot with respect to the synthon it seems to me that one would first need to locate the synthon in the STM images of Fig 3, without the Fe2 ion. How is this done? I thought that one could try to locate the DIBOD spot, but looking at the knots in panel D, for example, this seems hard to achieve, as the knots have either a relatively uniform brightness or a higher brightness in correspondence with their crossings.
Aside from that, it is also unclear how to define “translated away” for a knot that takes up a very considerable portion of the chain. To me, the best way would be to show that the knot position has no correlation with the position of the synthon, which could then appear in one of the loops or in the knot crossing region without preference. However it seems to me that some of the results in Fig 2 and the computational insights presented later show instead a preference for the synthon to be located in the crossing region. In the case of Fig. 2, the slight upfield shift and broadening of the proton signals for the bipyridines in 2 compared to 3 could be compatible with the synthon portion of the chain being more often within the crossing region of the knot, since this encourages interactions with other parts of the knotted loop. The computational model instead shows that a more rigid portion of the chain tends to be localized in the crossing region.

9. Page 9 “For the shorter strand trefoil [...] the proportion of the high symmetry conformer [...] was substantially lower”. As pointed before, this is not an obvious result. If there is space it would be nice to have it stressed more in the conclusion.

Page 9 Mechanistic insights from coarse-grained simulations

I have a few comments on how the simulations have been performed and analyzed. I strongly encourage the authors to rewrite this section and S11 to improve clarity, and to perform some additional simulations to better mimic adsorption. These could be put in SI as well.

[confinement vs adsorption] First of all, the simulations are based on knotted polymers confined between two close walls. While this mimics the quasi-2d situation of adsorbed polymers, the way this is achieved is very different from the experimental situation. It would be important to at least report some result from a proper, albeit very simplified, adsorption simulation. Using a Lj potential to mimic adsorption would be enough to reproduce the fact that when the strands cross the one that is further away from the surface is less attracted to it.

[literature on adsorption] There are several studies on quasi-2D knots and adsorbed knotted polymers that I suggest checking. To the best of my knowledge they consider longer homopolymers so that the results presented here are completely original, but a comparison would be nice. Please see previous work from Orlandini, Van Rensburg, and their collaborators.

[Parameters] The length of the chains is specified only in Fig 4. The parameters for the langevin are not reported in the SI. In fact, the integration timestep, damping coefficient, etc. are missing and should be added to the SI.

[Identification of two-loop and three-loop configurations] How are they identified exactly? S11 does not help that much. It defines a loop as a fragment between two crossings located at the exterior of the configuration. How do the authors define the interior and exterior of a knotted configuration though?

[Identification of high and low symmetry configurations] It is quite unclear how the authors distinguish between high-,

medium- and low-symmetry configurations. The description provided at page 9 “[...] based on the shortest the length between crossings in the major loops (L_short > 11 or <= 11 respectively” is insufficient. The explanation in S11 does not improve the situation. Why was the number 11 chosen? Why doesn’t it change when going from chain of length 60 to chain of length 100? This seems wrong, and I suspect that the number in question applies to only one chain length.

[statistical uncertainties in Fig 4c,d] Please add error bars in Fig. 4c,d.

Conclusions

The following questions are curiosities of mine. I think touching upon them in the manuscript would make it even more interesting to a number of researchers, but I leave the decision to the authors. I am not asking for further experiments.

* How difficult would it be to use this strategy to produce even longer knotted polymers? Doing so would allow one to study the transition to scaling polymer theory.

* Would it be possible to use co-polymers instead of PS or PEG, so that part of them has the same stiffness as the synthon? In principle, this should completely decorrelate the position of the knot from that of the synthon.

* Would it be doable to use a similar approach with more than one synthon, resulting in composite knots?

Clarity and context

I found the manuscript to be overall well written, with some minor clarifications required, as written above. The abstract and introduction clearly communicate the scope and significance of the work (see however my minor comments about page 3). The conclusions, as discussed above, could perhaps touch some more future applications. These however might be speculative and I thus leave the choice to the authors.

Version 1:

Reviewer comments:

Reviewer #1

(Comments for the Author)
The authors have addressed my and the other reviewers' comments satisfactorily and have improved the manuscript and ESI accordingly.

Reviewer #2

(Comments for the Author)
The authors are to be commended for such a thorough and thoughtful revision that addressed all of the points raised in my review. I think the manuscript is much improved overall and this is a good example of peer review working well.

In my opinion the revised manuscript is now acceptable for publication.

Reviewer #3

(Comments for the Author)
The authors have answered all my comments, and the manuscript can be published basically as is.

My only minor suggestion is that the authors add a sentence in the main text explaining the reason behind the threshold length 11 in section "mechanistic insight from coarse-grained simulations". While it is true that the rationale is explained in detail in the SI, I believe it will help the reader to find a short explanation right in the main text.

Regarding the authors' question about possible avenues for an experimental study of composite knots, I believe that the main question here is under what conditions prime components can expected to be localized in different portions of the polymer or nested one inside the other, and what are the consequences on the properties of the polymer ring. A few things come to mind. First, computational and experimental studies on DNA have shown that knots tend to weekly attract due to the finite size of the chain, and that nested configurations are favored in semiflexible, stretched polymers. Does this expectation hold in a case like the one investigated here? Furthermore, is it possible to tune this co-localization somehow? Do composite knots properties (e.g. hydrodynamic radius) correspond to those of prime knots of comparable complexity? Do they scale linearly with the number of prime components (I don't think so, but that is just my expectation)?

Open Access This Peer Review File is licensed under a Creative Commons Attribution 4.0 International License, which permits use, sharing, adaptation, distribution and reproduction in any medium or format, as long as you give appropriate credit to the original author(s) and the source, provide a link to the Creative Commons license, and indicate if changes were made.
In cases where reviewers are anonymous, credit should be given to 'Anonymous Referee' and the source.
The images or other third party material in this Peer Review File are included in the article's Creative Commons license, unless indicated otherwise in a credit line to the material. If material is not included in the article's Creative Commons license and your intended use is not permitted by statutory regulation or exceeds the permitted use, you will need to obtain permission directly from the copyright holder.
To view a copy of this license, visit https://creativecommons.org/licenses/by/4.0/

Reviewer #1:

The manuscript by Dai, Li, Leigh, Zhang et al describes the synthesis of trefoil knots and their macrocyclic topological isomers, their visualisation on STM and incorporation in block copolymers. The experimental data is backed by high level characterisation of all the building blocks and coarse-grind simulation. The manuscript is well written and the experimental data matches conclusions drawn by the authors. I would recommend the following points to be addressed before publications.

***Q1:** 'There are a few typos that can be quickly ironed out by a spell check ("reptation", "iamges", etc).'*

We have rechecked the manuscript carefully and corrected a number of typographical errors.

***Q2:** 'The STM assignment of the metallated trefoil twist and the DIBOD is based on size only. Could the authors get XPS data to support their assignment, especially for the metallated trefoil?'*

We have collected X-ray photoelectron spectroscopy (XPS) data for the metallated trefoil polymer [Fe**2**]$(PF_6)_2$ as suggested. The spectrum shows the presence of Fe, O, N, and C elements, fully consistent with the composition of the trefoil knotted polymer (Fig. R1). These data have now been added to the revised *Supplementary Information* as Figs. S77b and S77c.

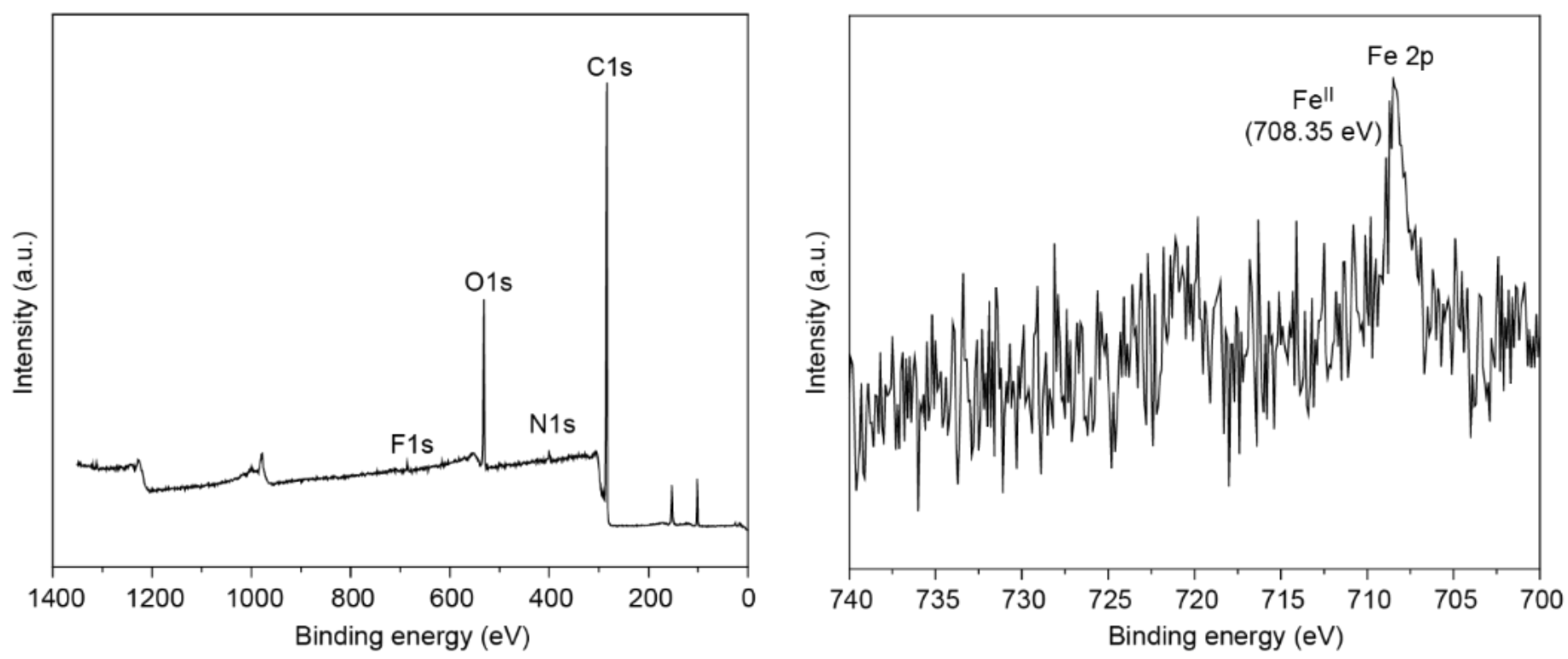

Fig. R1. XPS spectrum of the trefoil knotted polymer [Fe**2**]$(PF_6)_2$, showing the presence of Fe, O, N, and C elements, consistent with the assigned composition.

***Q3:** 'The ESI is very well put together but for consistency the authors should check the numbering and proton assignment for S7 and the precision with which they report the diffusion coefficients determined by DOSY.'*

We have corrected the proton assignments for compound S7 and revised all diffusion coefficients determined by DOSY to three significant figures. This level of precision is now used consistently throughout the manuscript and the Supplementary Information. We thank the reviewer for this

helpful suggestion, which has helped improve the overall quality and consistency of the manuscript.

Reviewer #2:

There have been other theoretical and experimental studies of knotted polymers, but none that are as thorough as this work. Here the chemical composition of the linear, cyclic, and trefoil-knotted polymers are so similar and this is extremely important when drawing conclusions on the effects of topology on the properties. This study is fundamental in so many ways. The molecular topology community has been speculating on many of the issues for decades and this work has now experimentally addressed and resolved them... I recommend publishing once the issues discussed above are addressed.

We sincerely thank the reviewer for these generous and encouraging comments. We are particularly pleased that the reviewer recognises the benefits of comparing linear, cyclic, and trefoil-knotted polymers with closely matched chemical compositions, and we share the reviewer's view that this platform provides an experimental foundation for resolving long-standing questions in molecular topology. We also appreciate the reviewer's perspective that this approach may help bridge experimental studies with theoretical and simulation efforts by enabling access to a broader range of well-defined topological polymers.

***Q1:** 'The PEG and PS-PEG polymers should be discussed more. I thought I accidentally misplaced a page of the manuscript because the synthesis of these are discussed, but the properties and how they articulate / compare with the PS polymer results is lacking.'*

Following the reviewer's suggestion, we have substantially expanded the characterisation and discussion of the PEG and PS–PEG systems. Specifically, we have collected gel permeation chromatography (GPC) and differential scanning calorimetry (DSC) data for both the PEG and PS-PEG polymers and added these results to the Supplementary Information (Section S12). GPC analysis shows that trefoiled PEG **8** possesses a smaller hydrodynamic volume and lower intrinsic viscosity than linear PEG **7**, consistent with a more compact conformation induced by knotting (Fig. R2). Differential scanning calorimetry further reveals a pronounced increase in glass-transition temperature (T_g) from −36.15 °C for linear PEG **7** to 2.18 °C for trefoiled PEG **8** (Fig. R3), attributable to reduced free volume and restricted chain mobility in the knotted architecture.

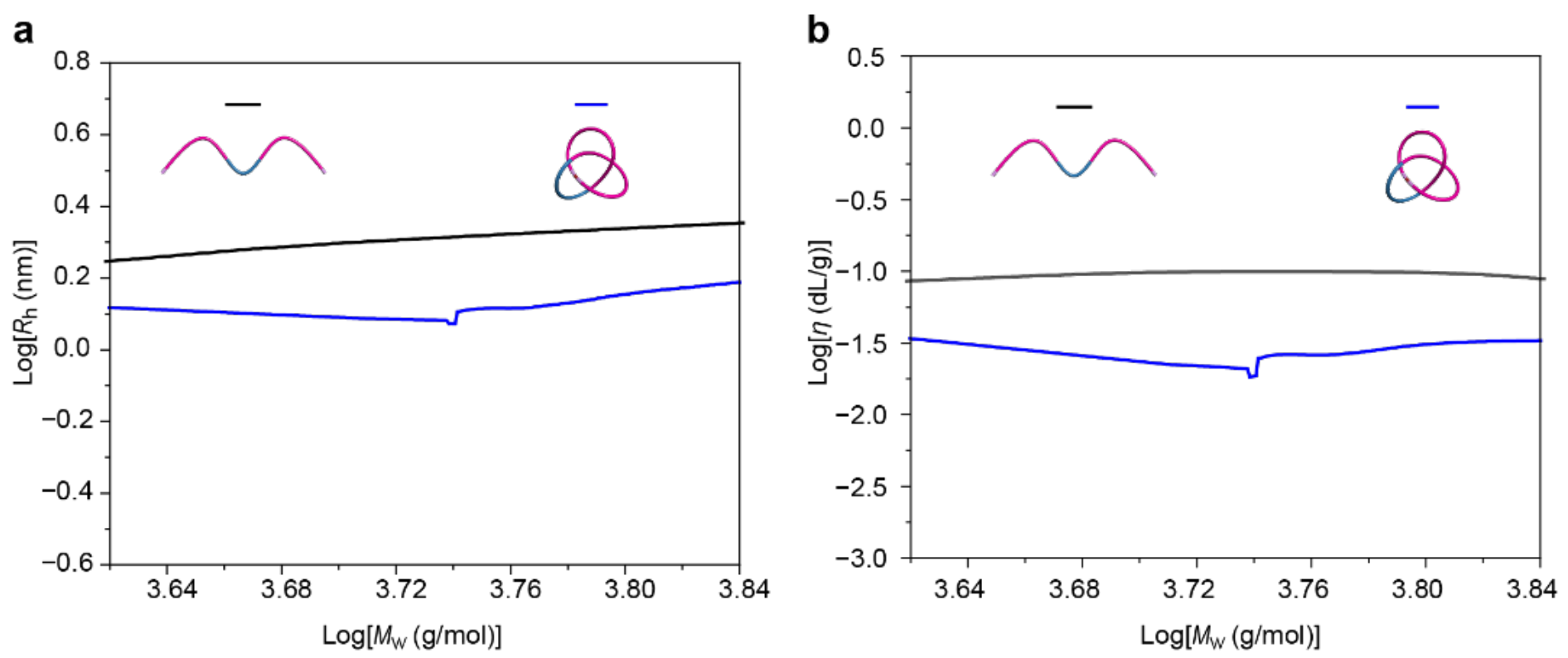

Fig. R2. (a) Logarithm of [R_h] versus the logarithm of molar mass; (b) Logarithm of [η] versus the logarithm of molar mass of **7** and **8**.

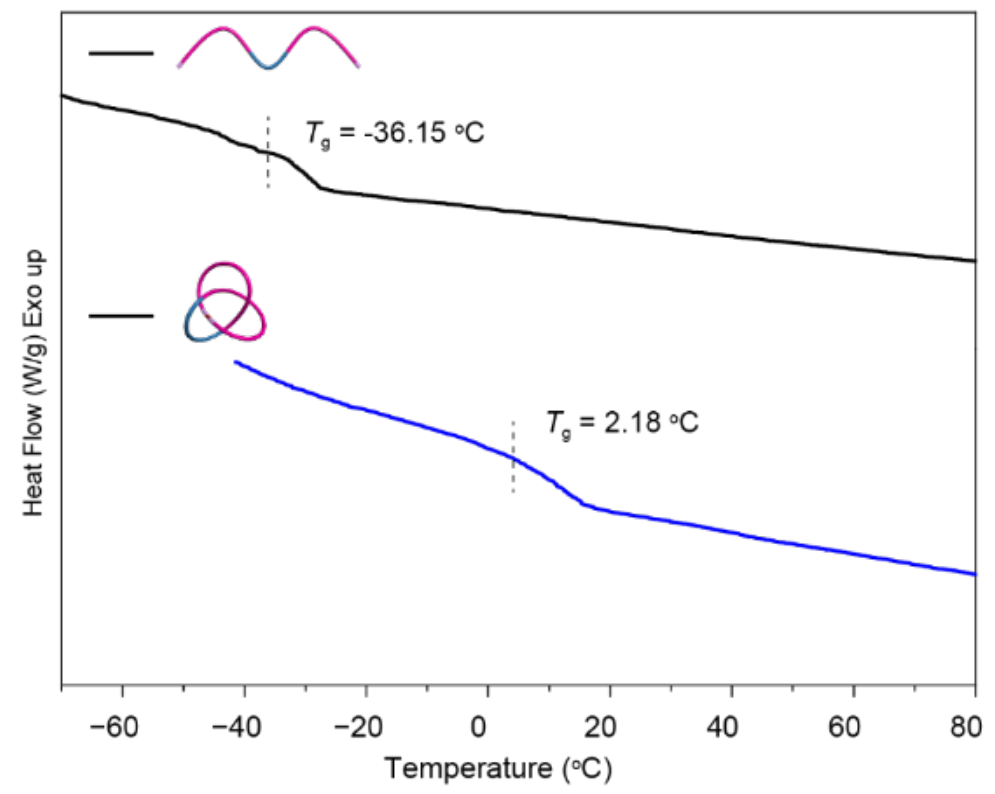

Fig R3. Differential scanning calorimetry (DSC) curves (T_g were determined by onset temperatures) of **7** and **8**.

Analogous trends are observed for the PS-PEG block copolymers. Trefoiled PS-PEG **10** exhibits a smaller hydrodynamic volume and lower intrinsic viscosity than its linear analogue **9**, and DSC analysis shows an increase in T_g from 31.02 °C (**9**) to 61.92 °C (**10**), again consistent with enhanced chain confinement upon knotting (Figs. R4 and R5).

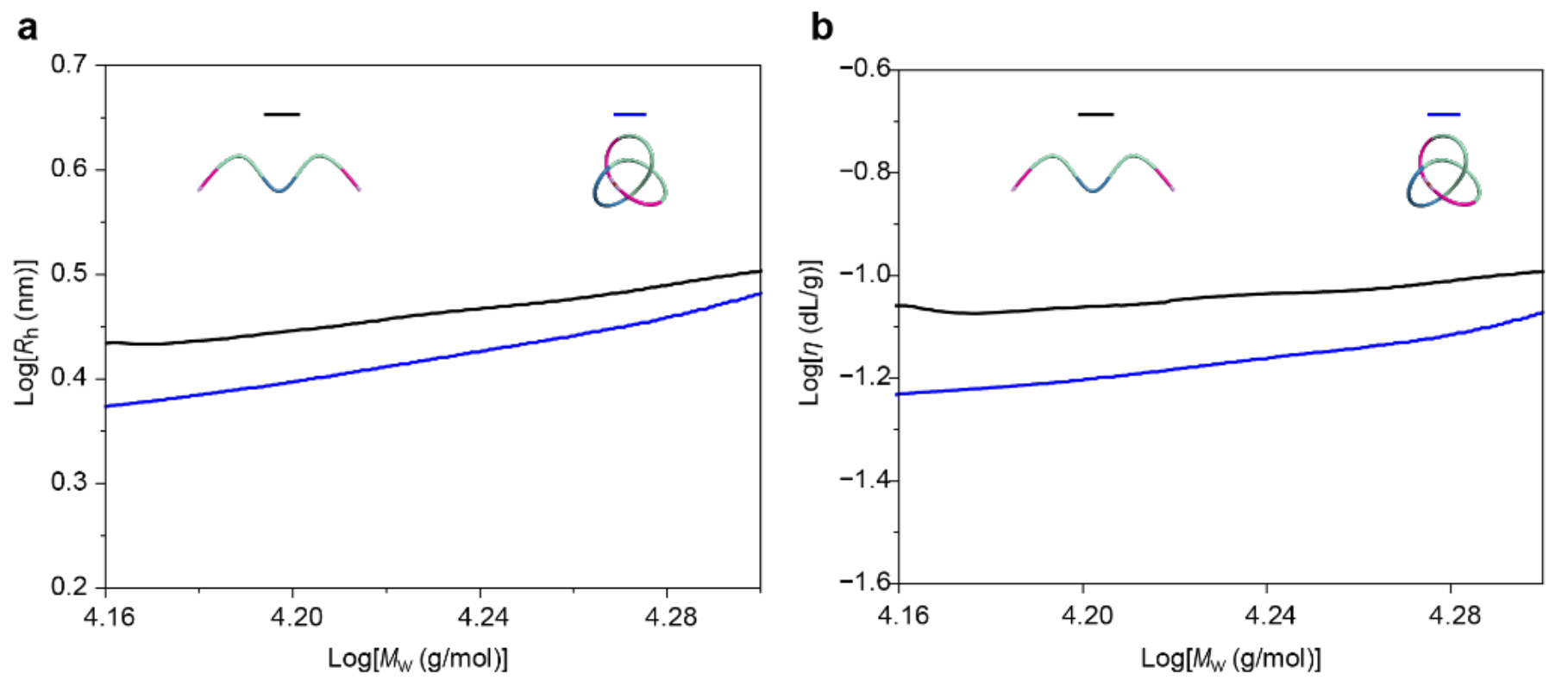

Fig. R4. (a) Logarithm of [R_h] versus the logarithm of molar mass; (b) Logarithm of [η] versus the logarithm of molar mass of **9** and **10**.

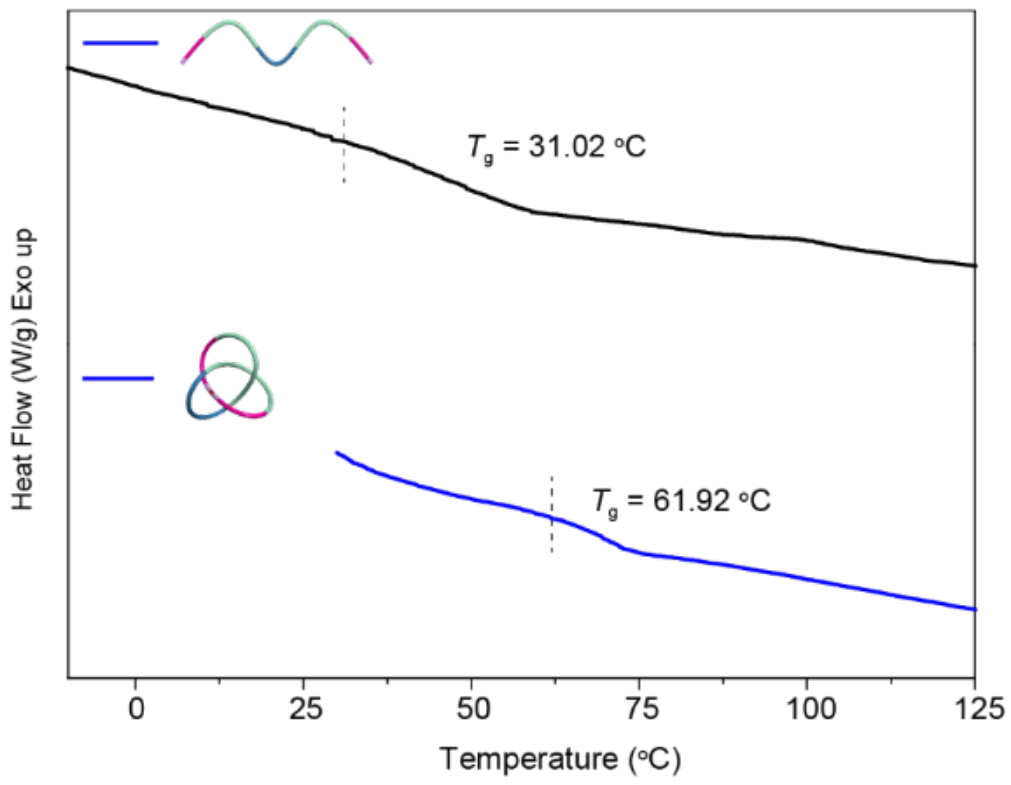

Fig. R5. Differential scanning calorimetry (DSC) curves (T_g were determined by onset temperatures) of **9** and **10**.

These results are now discussed in the revised manuscript, the sentence '*GPC indicates that trefoiled polymers exhibit systematically smaller hydrodynamic volumes and lower intrinsic viscosities than their linear analogues for both PEG and PS-PEG backbones (Figs. S93 and S99). DSC further shows pronounced increases in T_g upon trefoil formation (e.g., −36.15 °C to 2.18 °C for PEG and 31.02 °C to 61.92 °C for PS-PEG) (Figs. S94 and S100). These results demonstrate that topology alone, independent of chemical composition, has an important influence on bulk polymer properties.*' has been added. The corresponding data have been incorporated into the Supplementary Information as Figs. S93-S94 and S99-S100.

***Q2:** 'The macrocyclization step should be discussed more in the manuscript. These are done under high dilution conditions (dropwise addition), but I was surprised about how good the yields are. Do the authors have an idea of the effective concentration under their macrocyclization conditions and how fast the click reaction is under those conditions? The SI states the product "could contain some higher molecular weight by-product which formation by the oligomerization of …" How did the authors evaluate how much of this by-product is present. I guess the disappearance of the azide signal in the IR supports that it is not much, but was this by-product detected by mass spec or other means?'*

As suggested, we have expanded the discussion of the macrocyclization step in the main text. The macrocyclization follows the established slow-feeding/high-dilution logic used for bimolecular ring-closure reactions. In the precedent study we cite Ref. 1 (*Macromolecules* **2020**, *53*, 8621–8630, Supplementary Information). The ring-closing reactions are typically conducted at a nominal polymer concentration on the order of ~10^{-4}-10^{-5} M, and the slow feeding protocol further suppresses intermolecular coupling by keeping the instantaneous concentration of reactive chain ends low during addition. Kinetically, the double strain-promoted azide-alkyne cycloaddition (DSPAAC) between DIBOD and sterically hindered aryl azides displays a self-accelerating behaviour, with reported rate constants k_1 = 0.712 M^{-1} s^{-1} and k_2 = 128.16 M^{-1} s^{-1} ($k_2/k_1 \approx 180$). This

large k_2/k_1 ratio suggests that once the first cycloaddition occurs, the second (ring-closing) step becomes much faster, enabling efficient macrocyclisation even under very dilute conditions. We have incorporated a brief statement in the revised manuscript to rationalize the unexpectedly high macrocyclization efficiency in terms of this self-accelerating DSPAAC kinetics

'*The high macrocyclisation efficiency can be rationalised by the self-accelerating DSPAAC kinetics, in which the second cycloaddition is ~180-fold faster than the first, biasing the reaction toward intramolecular ring closure under high-dilution conditions.*'

As noted by the reviewer, a small amount of higher-molecular-weight material can arise from intermolecular coupling (oligomerisation) during macrocyclisation. Because direct determination of the macrocyclisation yield at the metallated intermediate stage is not straightforward, the efficiency of ring closure was instead evaluated after demetallation by size-exclusion chromatography (SEC), which clearly resolved the high-molecular-weight fraction from the target knotted polymer. Integration of the corresponding SEC peak areas indicates that the oligomeric by-products account for ~43% for the longer PS knot **2**, whereas the byproduct fraction decreases and the isolated yield increases to ~72% for the shorter PS knot **5** (Fig. R6), consistent with a chain-length-dependent decrease in macrocyclisation efficiency and an increased propensity for intermolecular coupling with longer polymers.

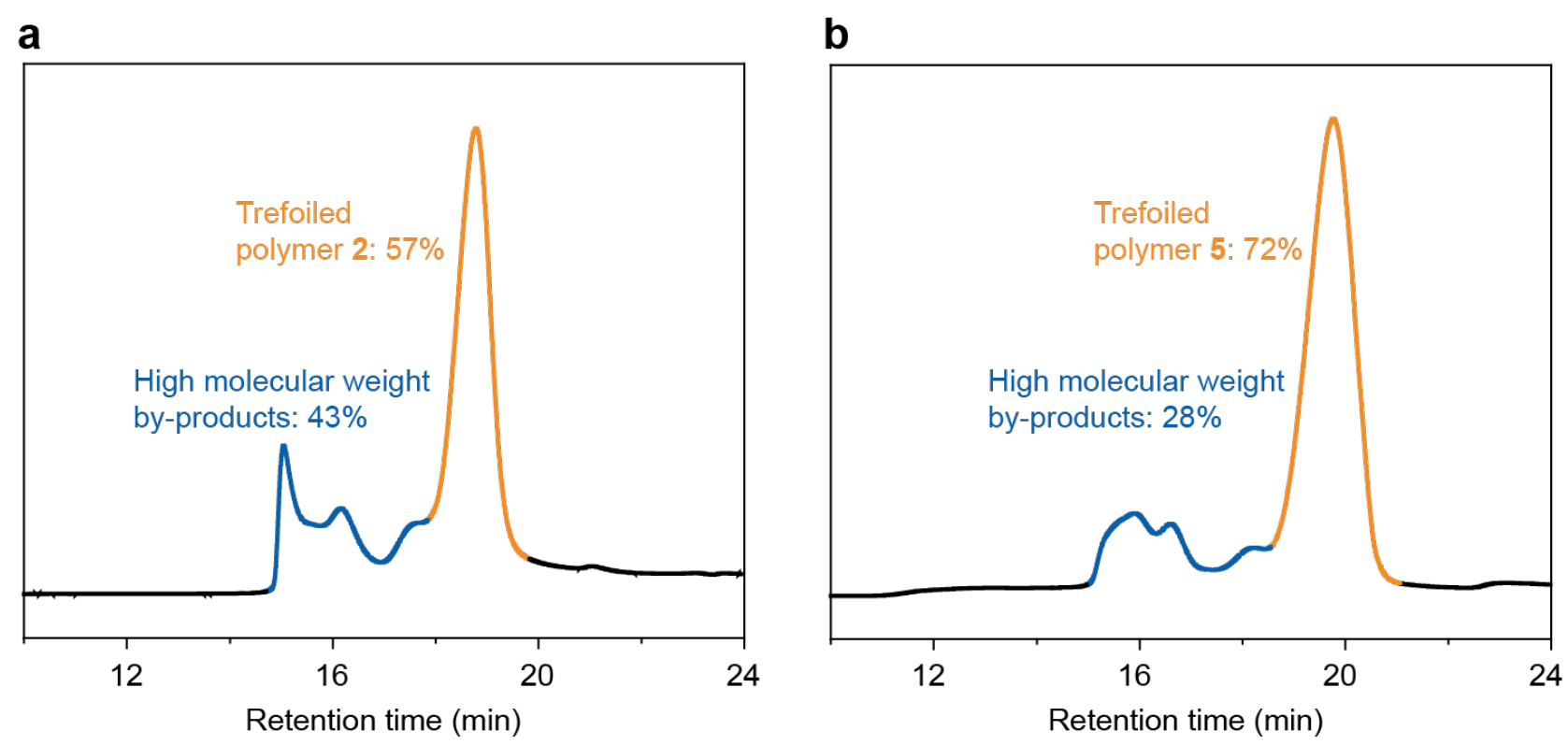

Fig. R6. SEC traces of crude trefoil PS **2** and **5**, showing that the fraction of knotted polymer increases for the shorter-chain sample, consistent with chain-length-dependent macrocyclisation efficiency.

The discussion of this data has been added to the revised Supplementary Information: '*Because direct determination of the macrocyclisation yield in the metallated intermediate is not straightforward, the efficiency of ring closure was evaluated after demetallation by size-exclusion chromatography (SEC). Preparative SEC of the crude organic knotted polymers reveals a distinct higher-molecular-weight fraction attributable to intermolecular oligomerization. Integration of SEC peak areas indicates that this fraction accounts for ~43% of the material for the longer PS knot* ***2****, whereas the proportion of oligomeric by-products decreases and the isolated yield increases to*

~72% for the shorter PS knot ***5****, consistent with chain-length-dependent macrocyclization efficiency. The disappearance of the azide stretching band in the IR spectra further supports high end-group conversion. Representative SEC traces and integrations are provided in Fig. S28.*'

Q3: '*At the end of the conclusion there is a one-sentence discussion of how this work might be extended to 5_2 or 8_19 knot synthons. The SI also mentions this, but also shows a 5_1 and composite knot. It would be nice if the composite knot is mentioned in the manuscript as well and a sentence or two of how the current synthesis might be adapted to make the granny/square knot composite polymer.'*

As suggested by reviewer, we have revised the sentence to '*We anticipate that combining together multiple trefoil synthons should generate composite knots (e.g. granny/square knots), and that other synthons (e.g. based on 5_1, 5_2 or 8_{19} small-molecule knots) should allow the precise synthesis of polymers with higher-order topologies.*[40,48,49]'

Reviewer #3:

I found some minor issues with the manuscript, regarding some missing connections to the existing theory, the statement that the knot "translates away from the synthon", and some aspects of the simulation part, which I believe could be extended and should be better explained. That notwithstanding, I think that this is a fascinating, seminal study that will help the community studying knotted polymers moving forward. In particular, this work paves the way for a more detailed characterization of knots in polymers far from their scaling limit and will be a huge help and motivator also for computational polymer physicists to study such systems. I highly recommend publication after the authors have tackled my, mostly minor, comments.

We agree that theoretical and computational studies in polymer physics have been a source of inspiration for this project, and cite it accordingly. We hope that the experimental platform established here, particularly the ability to access well-defined knotted polymers, will stimulate further collaborative efforts between experimentalists and computational polymer physicists.

Q1: '*Fig 1. I guess that 2n indicates the number of monomers in both PS, but it should be specified.'*

We have revised the figure caption accordingly, adding '*2n indicates the total number of PS monomers in polymer chains*'.

Q2: '*Page 3 The authors write "While theory suggests that strand length31,32 should influence the degree that a knot is confined to a particular region of a polymer strand, this has not previously been explored experimentally". Here it is not completely clear to me what the authors mean. If by "particular region" they mean a specific region with higher or lower flexibility than the rest of the chain, as the references suggest, I agree with them. But this needs to be specified. Related to this,*

the fact that the synthon is more rigid than the rest of the chain should be stated explicitly: while for most chemists this might be easily discerned, computational physicists will have difficulties understanding it from the chemical structure (I guess the bipyridines add enough rigidity compared to PS). It seems to me that up to page 3 of the manuscript this difference in flexibility can be understood only from the sentence "(LF, defined as LF =2LPS/LL1; LPS: length of PS chain; LL1: length of ligand L1) for the two trefoiled PS polymers of 3.9 (2) and 2.8 (5)", which implicitly proposes L1 as the most rigid part of the ring.'

We thank the reviewer for pointing out the ambiguity in this section. This paragraph was intended to describe the effect of the relative lengths of flexible and stiff segments on knot conformational properties. To avoid confusion, we now explicitly define the relevant parameter as a flexible–stiff length ratio and clarify the mechanical distinction between the polymer backbone and the knot synthon. The corresponding text in the main manuscript has been revised as follows.

'*While theory suggests that the lengths of flexible and stiff segments can influence knot conformational properties*[31,32]*, such effects have not previously been explored experimentally. In our system, polystyrene (PS) constitutes a relatively flexible segment, whereas the bipyridine units in the knot synthon form a comparatively stiffer region. We therefore define the flexible-stiff length ratio as $\chi_{FS} = (2L_{PS})/L_{L1}$, where L_{PS} is the length of PS chain and L_{L1} is the length of ligand* ***L1.*** *To probe the effect of χ_{FS}, we prepared a second set of linear (**4**), trefoiled (**5**), and cyclic (**6**) PSs using a PS chain with a lower molecular weight (**S18**: M_n = 2886 Da, Đ = 1.14; Supplementary Information, Section S5.5–S5.7), which should produce a tighter tangle in the trefoiled polymer. The contour lengths of the PS strands are 10.35 nm (**S17**) and 7.54 nm (**S18**) (Supplementary Information, Section S5.8), giving the corresponding ratios are χ_{FS} = 3.9 (**2**) and 2.8 (**5**), respectively. Although knot localisation occurs in the long-chain limit, where the most probable knot-core length becomes insensitive to the overall chain length,*[17,33,34] *the polymer lengths studied here do not reach this regime (Supplementary Information, Section 11).*'

The suggested references have been added to the revised manuscript as refs. 33 and 34:

33. Tubiana, L. Computational study on the progressive factorization of composite polymer knots into separated prime components. *Phys. Rev. E* **89**, 052602 (2014).

34. Rieger, F. C. & Virnau, P. A Monte Carlo study of knots in long double-stranded DNA chains. *PLOS Comput. Biol.* **12**, e1005029 (2016)

Q3: ***'****Page 3 "We therefore prepared a second set of linear (4), trefoiled (5), and cyclic (6) PSs using a PS chain with a lower molecular weight [...] which should produce a tighter tangle in the trefoiled polymer." I am not sure where this conclusion comes from in the literature. If we consider flexible homopolymers, the knot length distribution always has the same peak, indicating that knot length grows simply due to fluctuations that produce larger knots in proportion to the contour length of the rings (Tubiana, L. (2014). Physical Review E, 89(5), 052602; Rieger, F. C., & Virnau, P.*

(2016). PLOS Computational Biology, 12(9), e1005029), which results in the length of the knot growing sublinearly with that of the polymer. The tangle however could very well remain of the same size as soon as the knot becomes relatively localized, i.e. as soon as the knot occupies half of the chain or less. Theoretical analyses on semiflexible homopolymers argue that very long chains bring knot localization (Grosberg, A. Y., & Rabin, Y. (2007). Physical review letters, 99(21), 217801, ref 17 in the manuscript). In fact, the results reported in the rest of the paper are surprising because computational studies report that the highly symmetrical configuration of the trefoil is more compatible with shorter chain lengths (e.g. Katritch, V., Bednar, J., Michoud, D., Scharein, R. G., Dubochet, J., & Stasiak, A. (1996). Nature, 384(6605), 142-145).'

We agree that in the long-chain limit, knot localisation occurs, such that the knot-length distribution exhibits a fixed peak position. Beyond a threshold polymer length, increasing the chain length only extends the long-length tail of the distribution, while the peak location remains unchanged. However, the polymer lengths employed in the present study do not reach this threshold (~ 90). As a result, the knot-core length remains constrained by the total polymer length. We have added relevant discussion and simulation results in the main text and Supplementary Information Section 11.

'*Although knot localization occurs in the long-chain limit, where the most probable knot-core length becomes insensitive to the overall chain length, the polymer lengths studied here do not reach this regime (see Supplementary Information, Section S11 for details).*"

In Supplementary Information Section 11: '*Many previous studies have demonstrated knot localisation in sufficiently long polymers, where the most probable knot-core length becomes insensitive to the total chain length. To assess this regime in our system, we performed additional simulations of relatively long polymers confined in a slit with height h = 5. As shown in Fig. S83 (refer to Fig. R7 in this letter), the most probable knot length (i.e., the peak position of the distribution) increases from 35 to 45 as the chain length increases from 60 to 80. Upon further increasing the chain length beyond 80, the peak position remains fixed at 45, indicating the onset of knot localisation.*

The threshold chain length required to reach the long-chain limit depends on both the polymer persistence length and the slit height. For a persistence length of L_p = 2 and a slit height of h = 5, meaningful tests of knot localisation require comparing knot-length distributions for chains substantially longer than the most probable knot-core length (~ 45), typically at least twice as long (i.e., ~90). In contrast, the PS contour lengths used in the present study (62 and 88) lie below this threshold and therefore do not reach the long-chain limit necessary for probing knot localisation.'

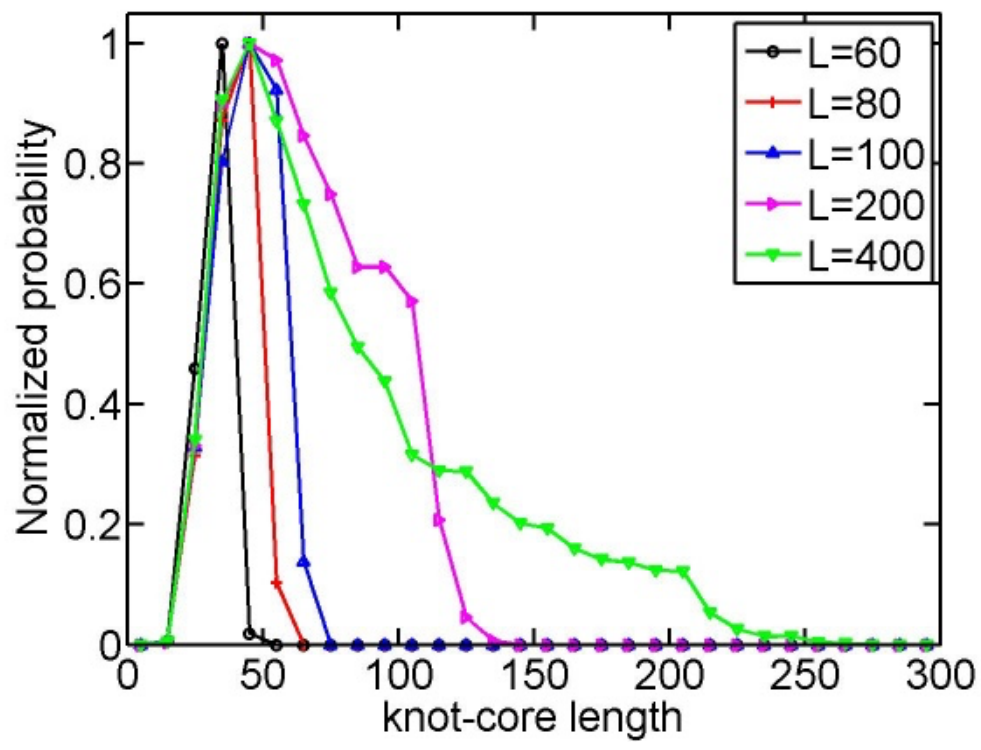

Fig. R7. Simulation results of knot-length distribution for confined polymer knots in a slit. The slit height is h = 5.

We agree that the 'three-loop' state probability increases for a short polymer, and we observe this trend in our simulation (see the figure below). However, to facilitate a direct comparison with experimental observations, we further divide the three-loop state into high-symmetry (L_{short} >11) and low-symmetry ($L_{short} \leqslant 11$) subclasses, where L_{short} denotes the contour length of the shortest loop. The two-loop state corresponds to the medium-symmetry state.

The cutoff value L_{short} = 11 is chosen based on the spatial resolution of the STM measurements. In our simulations, each bead represents approximately one styrene repeat unit with a backbone length of ~0.25 nm; thus, 11 beads correspond to a contour length of ~2.8 nm. When all three loops in a three-loop knot exceed this length, the conformation is unambiguously resolved as a three-loop state in STM images. In contrast, if one loop is shorter than 2.8 nm, the conformation might be misidentified as a two-loop state due to the finite STM resolution.

For this reason, in the main text we report the fraction of high-symmetry conformations rather than the total fraction of three-loop conformations. Identification of high-symmetry states, defined by all loop lengths exceeding 2.8 nm, is more reliable in STM images than identification of all three-loop states, which includes configurations with sub-resolution loops. Since the STM resolution is independent of polymer length, the same cutoff value L_{short} = 11 is used for both polymer lengths studied in the experiments.

We note that for shorter polymers, the three-loop probability increases whereas the high-symmetry probability decreases. This opposite trend arises because, for shorter three-loop knots, the loop lengths are more likely to fall below the experimental resolution and therefore are not classified as high-symmetry states (Fig. R8).

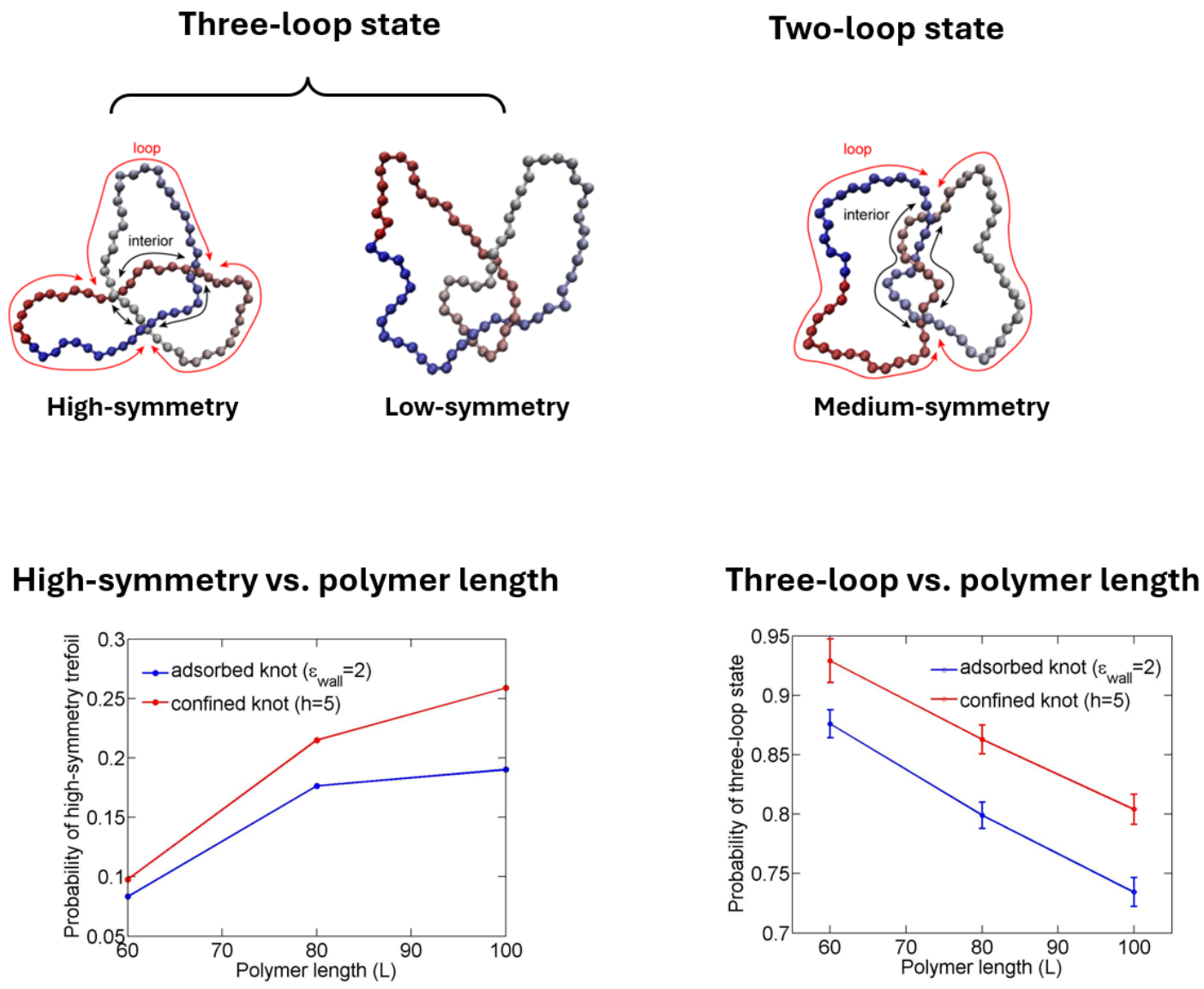

Fig. R8. The identification of distinct three-loop and two-loop conformational states in the trefoiled polymer, together with the chain-length dependence of their relative populations.

These simulations and discussion have been incorporated into the revised manuscript. The sentences '*The tightest trefoil knots are expected to adopt the three-loop state*[46]*, consistent with this expectation, our simulations show that the three-loop probability increases for shorter polymers (Supplementary Information, Section S11). Notably, however, for shorter polymers the three-loop probability increases while the high-symmetry probability decreases. This opposite trend arises because, for shorter three-loop knots, the loop lengths are more likely to fall below the experimental resolution and therefore are not classified as high-symmetry states.*' has been added. The corresponding reference has been added as ref. 47 in the revised manuscript.

47. Katritch, V. *et al*. Geometry and physics of knots. *Nature* **384**, 142-145 (1996).

Q4: *'Pages 6-7 "the knot does not adopt a single well-defined conformation but rather undergoes rapid reptation of the different sections of the strand through the region of entanglement" (bold font is mine). I agree that the chain certainly moves through the knot. Only, without knowing the rapidity of variation of the temperature in the VT-NMR, I find it difficult to understand how rapid this reptation is. Would it be possible to compare it against other dynamical times of the system? On a more technical note, the authors use the term reptation, but it could also be that the knot breathes enough for it to "move along" the chain in a mechanism that is different from reptation.'*

We agree that chain segments can move through the knot via multiple dynamic mechanisms, including both 'knot breathing' (fluctuations in knot-core length) and sliding of the knot along the polymer backbone, and that distinguishing the corresponding timescales is not possible with the techniques employed in this work. Accordingly, we have revised the sentence to '*…, indicating that the knot does not adopt a single well-defined conformation but rather undergoes knot 'breathing' (fluctuations in knot-core length) and/or sliding along the polymer backbone.*' to avoid implying a

specific microscopic mechanism.

***Q5:* ‘*Page 7 The authors write: “The diffusion coefficient (D) of cyclic PS3 was higher than that of the linear analogue 1 across a range of solvents [...] indicating a smaller hydrodynamic radius (Rh) [...] In contrast, trefoil PS 2, the topology with the smallest Rh, showed similar or smaller D values in these solvents compared to 3.” I am a bit confused. From the first sentence, it seems that Rh is estimated from D, while for the second sentence to hold Rh must be measured independently of D. Please clarify how Rh is measured.’*

In this work, hydrodynamic radius (R_h) was evaluated using two independent experimental approaches, diffusion-ordered NMR spectroscopy (DOSY) and size-exclusion chromatography (GPC), and the trends derived from both methods are consistent.

In DOSY experiments, diffusion coefficients (D) are directly measured and converted to apparent hydrodynamic radii using the Stokes-Einstein relationship. Thus, the statement that cyclic PS **3** exhibits higher D values than linear PS **1** corresponds to a smaller apparent R_h derived from DOSY. Independently, GPC provides relative hydrodynamic size information through elution volume, which reflects the polymer's solution hydrodynamic volume and can be correlated to R_h. Although absolute R_h values obtained from DOSY and GPC differ because of distinct models, calibration procedures, and assumptions, both techniques consistently indicate that the trefoil knot possesses the most compact conformation, followed by the cyclic polymer and then the linear analogue.

To avoid confusion, the corresponding sentences have been revised to ‘*The diffusion coefficient (D) of cyclic PS **3** was higher than that of the linear analogue **1** across a range of solvents with varying polarities ($CDCl_3$, tetrahydrofuran-d_8, dimethylsulfoxide-d_6, $Cl_2CDCDCl_2$), corresponding to a smaller effective hydrodynamic radius (R_h) as derived from DOSY measurements and independently supported by GPC analysis. In contrast, trefoil PS **2**, the topology with the smallest apparent R_h, showed similar or smaller D values in these solvents compared to **3**.*’

***Q6:* ‘*Page 7 “Theory suggests there will be influenced [...]” (bold is mine). Typo: there -> these.’*

We have corrected this typo.

***Q7:* ‘*Page 7 “solvation conditions and the presence of external forces such as temperature or shear” (bold is mine). Temperature is not a force.’*

We have changed the sentence to ‘*…, solvation conditions, temperature, and the presence of external forces such as shear,..*’

***Q8:* ‘*Page 7 “the STM images show the knotted region to be much looser and translated away*

from the original synthon into the PS chains". I agree that the knot is much looser, but I am not sure whether the data from STM show that the knot has translated away from the synthon, and I would like the authors to clarify this. To establish the position of the knot with respect to the synthon it seems to me that one would first need to locate the synthon in the STM images of Fig 3, without the Fe2 ion. How is this done? I thought that one could try to locate the DIBOD spot, but looking at the knots in panel D, for example, this seems hard to achieve, as the knots have either a relatively uniform brightness or a higher brightness in correspondence with their crossings.

Aside from that, it is also unclear how to define "translated away" for a knot that takes up a very considerable portion of the chain. To me, the best way would be to show that the knot position has no correlation with the position of the synthon, which could then appear in one of the loops or in the knot crossing region without preference. However it seems to me that some of the results in Fig 2 and the computational insights presented later show instead a preference for the synthon to be located in the crossing region. In the case of Fig. 2, the slight upfield shift and broadening of the proton signals for the bipyridines in 2 compared to 3 could be compatible with the synthon portion of the chain being more often within the crossing region of the knot, since this encourages interactions with other parts of the knotted loop. The computational model instead shows that a more rigid portion of the chain tends to be localized in the crossing region.'

We agree that our original wording could be interpreted as implying a level of positional assignment that is not directly supported by the STM data.

By '*translated away from the synthon*', we intended to convey that, upon removal of the metal chaperone, the knot is no longer fixed at the tris-bipyridine synthon in a well-defined, templated geometry, but instead becomes mobile along the polymer backbone and adopts a more delocalised, dynamic distribution. We did not mean to imply that the exact position of the knot relative to the synthon can be directly determined from the STM images.

At present, we cannot unambiguously locate the tris-bipyridine synthon or the DIBOD unit within the STM images of the demetallated trefoil polymer. The spatial resolution of STM under these conditions does not allow stereochemical resolution of trefoil crossings, and both internal knot crossings and bulky aromatic units can appear as bright features, making definitive assignment challenging whether the DIBOD unit lies within or near the crossing region.

We agree that the most appropriate description is not a literal 'translation away', but rather a change from a synthon-anchored knot to a dynamically distributed knot whose position is not uniquely correlated with a single structural motif. Consistent with the reviewer's analysis, our NMR data and computational results suggest that relatively rigid segments of the chain, such as the tris-bipyridine unit, may be statistically more likely to occupy the crossing region, although this preference is significantly weaker than in the metal-templated synthon [Fe**2**](PF_6)$_2$, where the knot

is tightly localised.

Accordingly, we have revised the manuscript to remove the phrase 'translated away from the synthon' and to state instead that removal of the metal chaperone leads to a looser, dynamic knot that is no longer rigidly anchored at the synthon, while allowing for a modest statistical preference of stiffer segments to reside within the crossing region. The corresponding sentence has been revised to '*In contrast, in metal-free trefoiled PS (**2**) the STM images show a much looser knotted region that is no longer rigidly anchored at the synthon but instead appears dynamically distributed along the PS chains (Fig. 3c and 3d and Figs. S71-S74).*'

***Q9:** 'Page 9 "For the shorter strand trefoil [...] the proportion of the high symmetry conformer [...] was substantially lower". As pointed before, this is not an obvious result. If there is space it would be nice to have it stressed more in the conclusion.'*

We thank the reviewer for highlighting this non-intuitive observation and agree that it merits additional emphasis. As discussed above (see **#Reviewer 3, Q3**), shortening the polymer chain leads to an increase in the population of three-loop conformers, while the proportion of high-symmetry conformers decreases. These opposing trends arise because, for shorter trefoiled polymers, the individual loop lengths are more likely to fall below the experimental resolution threshold and therefore are not classified as high-symmetry states, even though they still adopt a three-loop topology.

***Q10:** 'I have a few comments on how the simulations have been performed and analyzed. I strongly encourage the authors to rewrite this section and S11 to improve clarity, and to perform some additional simulations to better mimic adsorption. These could be put in SI as well.*

[confinement vs adsorption] First of all, the simulations are based on knotted polymers confined between two close walls. While this mimics the quasi-2d situation of adsorbed polymers, the way this is achieved is very different from the experimental situation. It would be important to at least report some result from a proper, albeit very simplified, adsorption simulation. Using a Lj potential to mimic adsorption would be enough to reproduce the fact that when the strands cross the one that is further away from the surface is less attracted to it.'

This is an excellent suggestion. We perform additional simulations of adsorbed polymer knots to mimic the polymer knots in experiments (Fig. R9). In these simulations, there is only one wall. A polymer conformation is adsorbed by an attractive LJ potential between the wall and beads: $E_{wall} = 4\varepsilon_{wall}\left[\left(\frac{\sigma}{r}\right)^{12} - \left(\frac{\sigma}{r}\right)^{6}\right] + \varepsilon_{wall}$. for $r < r^*_{wall}$. The attraction strength and range are $\varepsilon_{wall} = 2$ and $r^*_{wall} = 3$ unless otherwise specified.

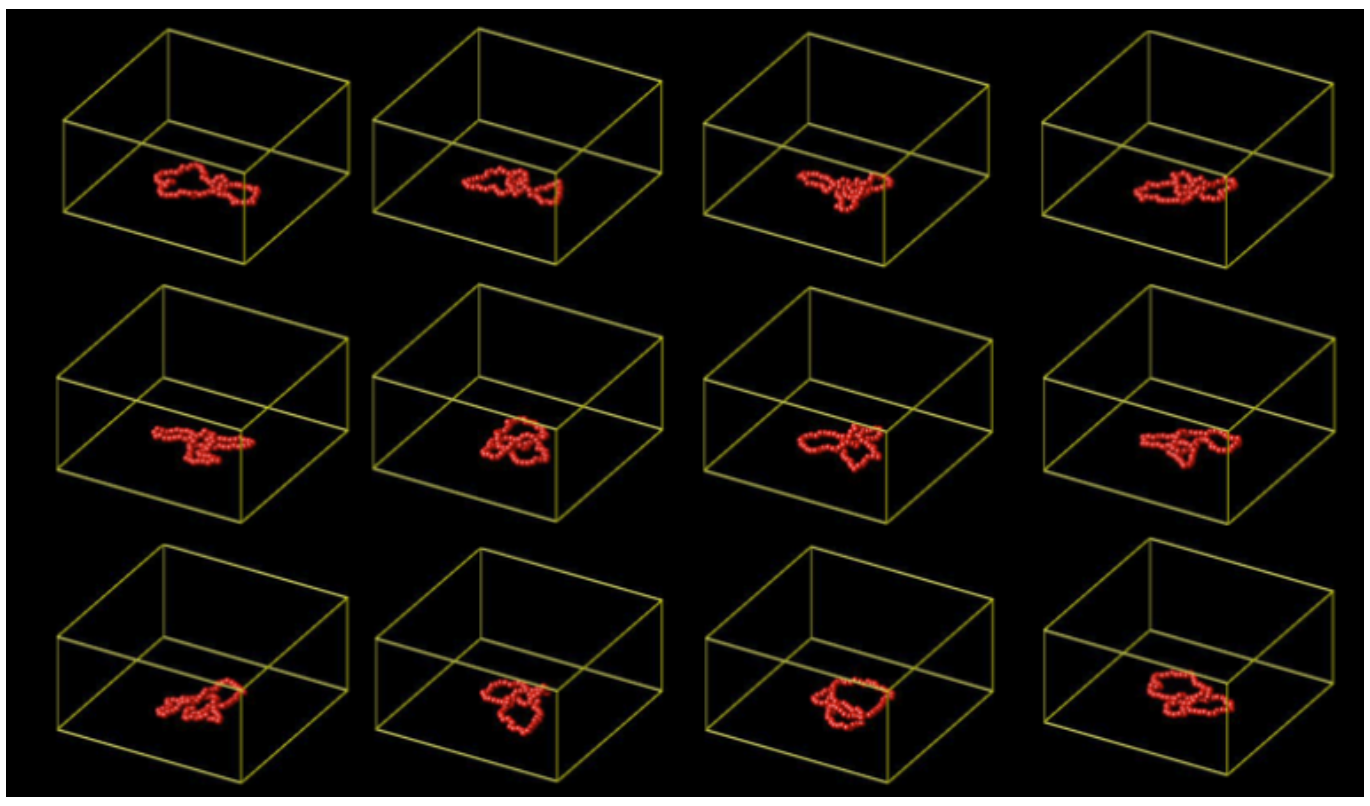

Fig. R9. Simulation snapshots of an adsorbed polymer knot.

The parameters of polymer interactions remain unchanged as described above. As shown in Fig. R10a, the high-symmetry probability of adsorbed knots increases with polymer length, exhibiting the same trend as observed in experiments and in confined-knot simulations. The high-symmetry probability of adsorbed knots decreases upon introducing weak bead–bead attraction (Fig. R10b), again consistent with both experimental observations and confined-knot results.

Notable differences between adsorbed and confined knots are observed in Fig. R10a. These differences are not surprising, as adsorbed polymers retain finite fluctuations in their conformational height, whereas confined polymers are strictly restricted by the slit geometry. To further elucidate the origin of these differences, we systematically vary the bead–wall attraction strength for adsorbed knots and the slit height for confined knots. As shown in Fig. R10c–d, the high-symmetry probability decreases with increasing bead–wall attraction strength, while it increases with increasing slit height. These two sets of results are mutually consistent, suggesting that a stronger bead–wall attraction effectively corresponds to a smaller slit height, leading to enhanced confinement of polymer conformations.

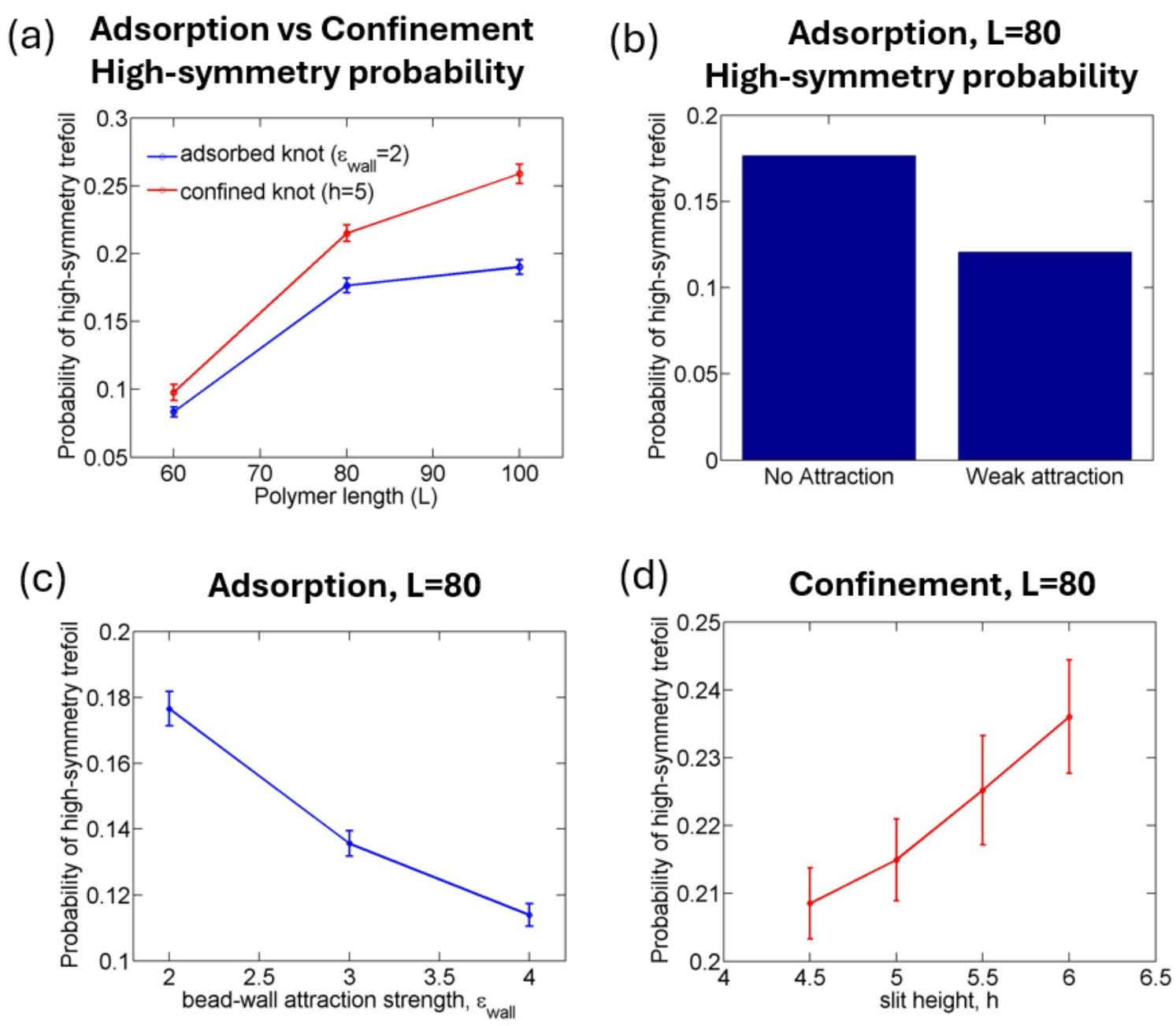

Fig. R10. Simulation results of absorbed knots and comparison with confined knots. (a) High-symmetry probability as a function of polymer length for adsorbed and confined knots. (b) High-symmetry probability of adsorbed knots with and without weak bead–bead attraction. (c) High-symmetry probability as a function of bead–wall attraction strength. (d) High-symmetry probability as a function of slit height for confined knots.

The sentence '*In addition to the confined knots, we also performed simulations of adsorbed knots, which exhibit similar results as the confined knots (Supplementary Information, Section S11).*' has been added, the corresponding reference has been added as ref. 45 and 46 in the revised manuscript. Additional simulation and discussion have also been added into the revise *Supplementary Information* Section 11.

45. van Rensburg, E. J. Squeezing knots. *J. Stat. Mech.* **2007**, P03001 (2007).
46. Marcone, B., Orlandini, E. & Stella, A. Knot localization in adsorbing polymer rings. *Phys. Rev. E* **76**, 051804 (2007).

Q11: *'[literature on adsorption] There are several studies on quasi-2D knots and adsorbed knotted polymers that I suggest checking. To the best of my knowledge they consider longer homopolymers so that the results presented here are completely original, but a comparison would be nice. Please see previous work from Orlandini, Van Rensburg, and their collaborators.'*

We have added the corresponding literatures about confined knots, adsorbed knots, and knot localisation, including: Micheletti, C. & Orlandini, E. Numerical study of linear and circular model DNA chains confined in a slit: metric and topological properties. *Macromolecules* **45**, 2113-2121 (2012).; van Rensburg, E. J. Squeezing knots. *J. Stat. Mech.* **2007**, P03001 (2007). In addition, we have added our simulation results of localisation of quasi-2D knots (see Fig. R7 above),

consistent with the results in *Phys. Rev. E* **76**, 051804 (2007).

Q12: *'[Parameters] The length of the chains is specified only in Fig 4. The parameters for the langevin are not reported in the SI. In fact, the integration timestep, damping coefficient, etc. are missing and should be added to the SI.'*

We have added all simulation parameters in the Supplementary Information Section 11. Below shows some of the parameter descriptions.

'To understand the conformational properties of a polymer knot on a surface, we carried out coarse-graining (CG) simulations of polymer knots using the LAMMPS program. The polymer chain was modelled as a string of beads, same as the previous studies.[3-5] In this model, adjacent beads are connected by bonds, described by the finite extensible nonlinear elastic (FENE) potential:

$$E_{bond} = -0.5\, K R_0^2 ln\left[1 - \left(\frac{r}{R_0}\right)^2\right] + 4\varepsilon\left[\left(\frac{\sigma}{r}\right)^{12} - \left(\frac{\sigma}{r}\right)^6\right] + \varepsilon,$$

$$\text{with } K = 30,\ R_0 = 30,\ \varepsilon = 1,\ \sigma = 1.$$

The pairwise interaction between beads is described by the Weeks−Chandler−Andersen (WCA) potential, i.e. the Lennard-Jones (LJ) potential with the cutoff distance of $r_{cut} = 2^{1/6}a$, where a is the bead diameter and serves as the length unit.

$$E_{pair} = 4\varepsilon\left[\left(\frac{\sigma}{r}\right)^{12} - \left(\frac{\sigma}{r}\right)^6\right] + \varepsilon, \text{ with } \varepsilon = 10,\ \sigma = 1,\ r \leq 2^{1/6}$$

The WCA potential is a hardcore repulsion, which is used to describe a polymer in good solvent. For each three adjacent beads, a bending potential is applied:

$$E_{bend} = (1/2)(L_p/a)\theta^2\, k_B T,$$

where θ is the bending angle, a is the hardcore diameter of bead, L_p is the persistence length to specify the polymer bending stiffness, and $k_B T$ is the Boltzmann energy and serves as the energy unit. We set $L_p = 2$ unless otherwise specified. The temperature and damping coefficient are set to 1. We started the simulation with a trefoil-knotted conformation. During the simulation, the knotted state is preserved because polymer segments cannot cross each other. We run simulations for 10^9 steps with time step of 0.01 and saved conformations every 10^5 for analysis.'

Q13: *'[Identification of two-loop and three-loop configurations] How are they identified exactly? S11 does not help that much. It defines a loop as a fragment between two crossings located at the exterior of the configuration. How do the authors define the interior and exterior of a knotted configuration though?'*

We have added the details of the algorithm for distinguishing two-loop and three-loop polymer conformations in the revised Supplementary Information.

'*We develop a geometric criterion to distinguish two-loop and three-loop polymer conformations*

on a surface. Here, the two-loop state corresponds to the medium-symmetry trefoil, and the three loop-state includes high-symmetry and low-symmetry trefoils. As illustrated in Fig. R11a, a two-loop conformation contains three crossings, labelled A, B, and C. Two loops connect crossing points A and C and form a closed curve, indicated by the dashed line in Fig. R11a. The defining feature of a two-loop conformation is that the remaining crossing point, B, lies inside this closed curve. In contrast, a three-loop conformation does not satisfy this criterion. As shown in Fig. R11b, for a three-loop conformation, each crossing point lies outside the closed curve formed by the two loops connecting the other two crossings.

In a two-loop conformation, only one of the three crossings satisfies the "inside-the-curve" criterion. Therefore, we apply this test to all three crossings and identify the conformation as two-loop if exactly one crossing is found to be inside the corresponding closed curve. This algorithm applies to conformations containing exactly three crossings. In our simulations, only a small fraction (a few percent) of conformations contain more than three crossings, as additional crossings produce extra energetic costs. These rare conformations are excluded from the analysis.

To determine whether a point lies inside a closed curve in two dimensions, we employ the ray-casting algorithm. A point is inside the curve if a ray starting from the point intersects the curve an odd number of times. In practice, we choose a horizontal ray, as illustrated in Fig. R11a. In our simulations, the closed curve is represented by a sequence of beads and thus forms a polygon. To determine whether the ray intersects a given polygon segment, we use the criterion $(y_i - y_B)(y_{i+1} - y_B) < 0$ *and* $x_{i+1} > x_B$*, where* (x_B, y_B) *are the coordinates of the crossing point B, and* (x_i, y_i) *and* (x_{i+1}, y_{i+1}) *are the coordinates of the two beads defining the segment.*'

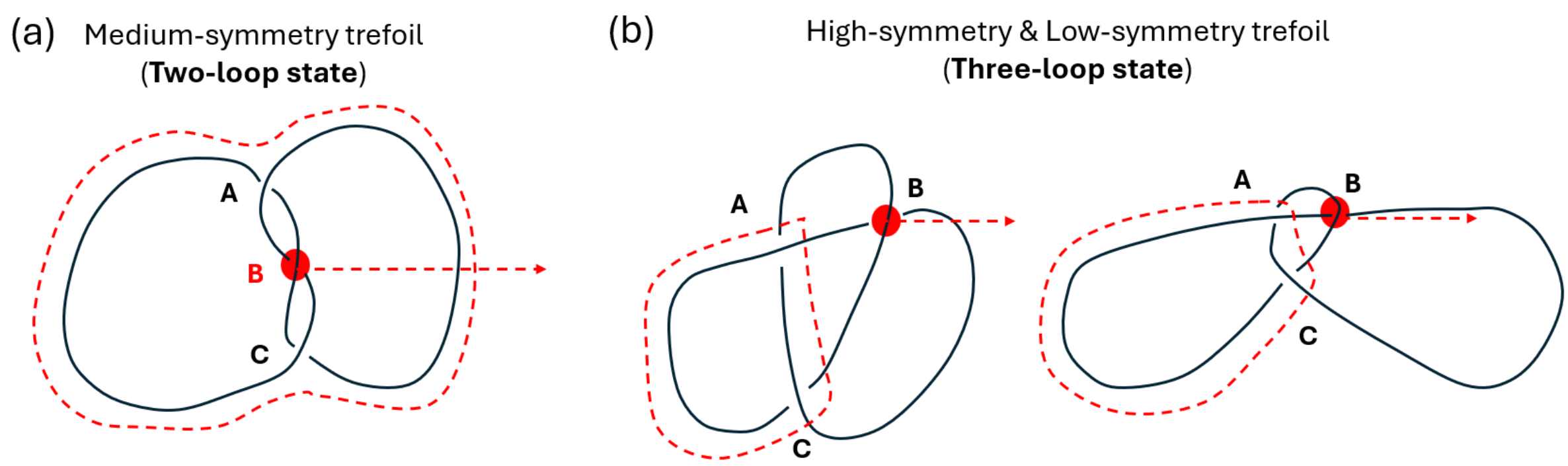

Fig. R11. Algorithm for distinguishing two-loop and three-loop polymer conformations. (a) The two-loop state satisfies a criterion: a crossing (red dot) locates inside the closed curve (dashed curve) formed by the two loops connecting the remaining two crossings. When the red dot is in the red dashed loop, the red arrow intersects the red dashed loop an odd number of times. (b) The three-loop state does not satisfy this criterion. When the red dot is not in the red dashed loop, the red arrow intersects the red dashed loop an even number of times.

The above content has been added into revised Supplementary Information Section 11.

Q14: *'[Identification of high and low symmetry configurations] It is quite unclear how the authors distinguish between high-, medium- and low-symmetry configurations. The description provided at page 9 "[...] based on the shortest the length between crossings in the major loops (L_short > 11 or <= 11 respectively" is insufficient. The explanation in S11 does not improve the situation. Why was the number 11 chosen? Why doesn't it change when going from chain of length 60 to chain of length 100? This seems wrong, and I suspect that the number in question applies to only one chain length.'*

In revised Section 11, we have added the reason for selecting the cutoff of 11 and using the same cutoff for different polymer lengths.

'The cutoff value L_{short} = 11 is chosen based on the spatial resolution of the STM measurements. In our simulations, each bead represents approximately one styrene repeat unit with a backbone length of ~ 0.25 nm; thus, 11 beads correspond to a contour length of ~ 2.8 nm. When all loops in a three-loop knot exceed this length, the conformation is unambiguously resolved as a three-loop state in STM images. In contrast, if one loop is shorter than ~ 2.8 nm, the conformation might be misidentified as a two-loop state due to the limits of STM resolution.

For this reason, in the main text we report the fraction of high-symmetry conformations rather than the total fraction of three-loop conformations. Identification of high-symmetry states, defined by all loop lengths exceeding ~ 2.8 nm, is more reliable in STM images than identification of all three-loop states, which includes configurations with sub-resolution loops. Since the STM resolution is independent of polymer length, the same cutoff value L_{short} = 11 is used for both polymer lengths studied in the experiments.'

We also classified the 69 trefoil **2** to high-, medium-, and low-symmetry configurations and measured the length of each loop as shown in Figs. R12-14. As shown in the Fig. R12, the length of each loop length of high-symmetry configurations are among 5-14 nm.

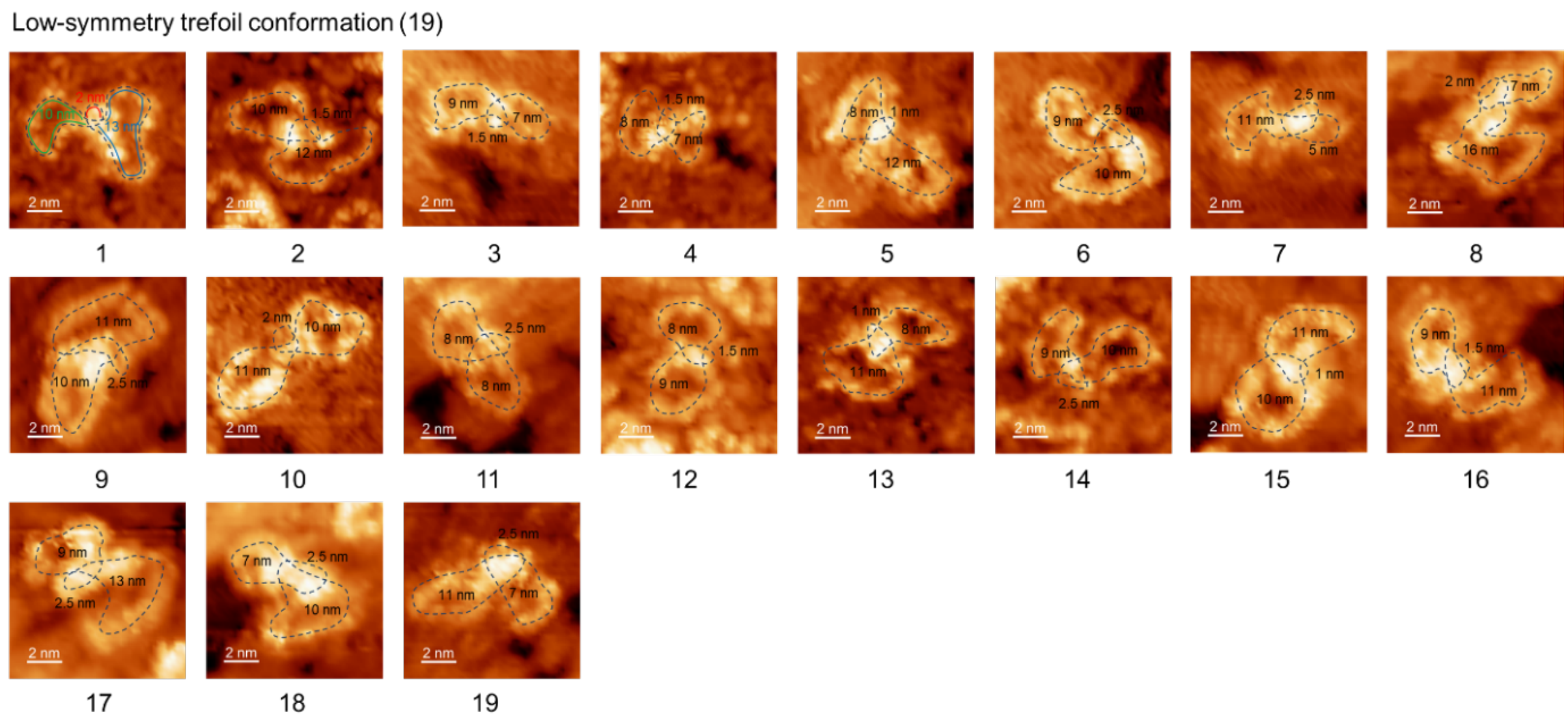

Fig. R12. Single molecular STM images of low-symmetry trefoil **2** with DCM as solvent for preparing the samples. A black dashed line superimposed on each molecule outlines the skeletal structure of the low-symmetry trefoil, with the corresponding loop lengths as indicated. The loop lengths are labelled in the loop. After analysing 69 individual images, it was found that 19 (28%) exhibited low-symmetry conformation (Scanning parameters: U = 2.7 V, I_t = 20 pA). If the contour length of the shortest loop is less than 2.8 nm, we assign the structure as a low-symmetry trefoil. In contrast, when all three loops of a three-loop knot exceed this threshold length, the conformation is accordingly classified as a high-symmetry trefoil. Furthermore, if a molecule exhibits a two-loop state with a shared elongated interior region, it is considered a medium-symmetry trefoil.

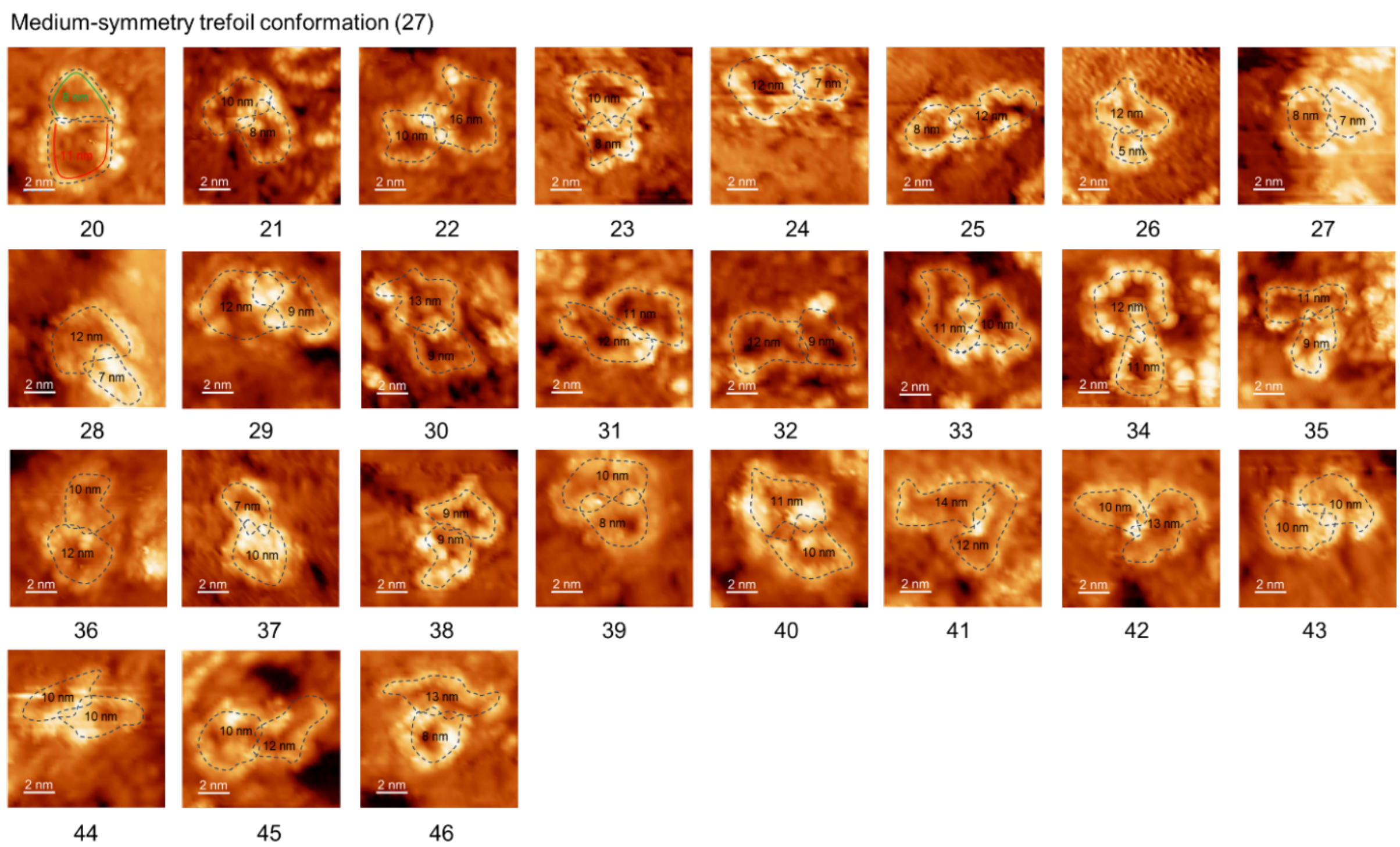

Fig. R13. Single molecular STM images of medium-symmetry trefoil **2** with DCM as solvent for preparing the samples. A black dashed line superimposed on each molecule outlines the skeletal

structure of the medium-symmetry trefoil, with the corresponding loop lengths as indicated. After analysing 69 individual images, it was found that 27 (39%) exhibited medium-symmetry conformation (Scanning parameters: U = 2.7 V, I_t = 20 pA).

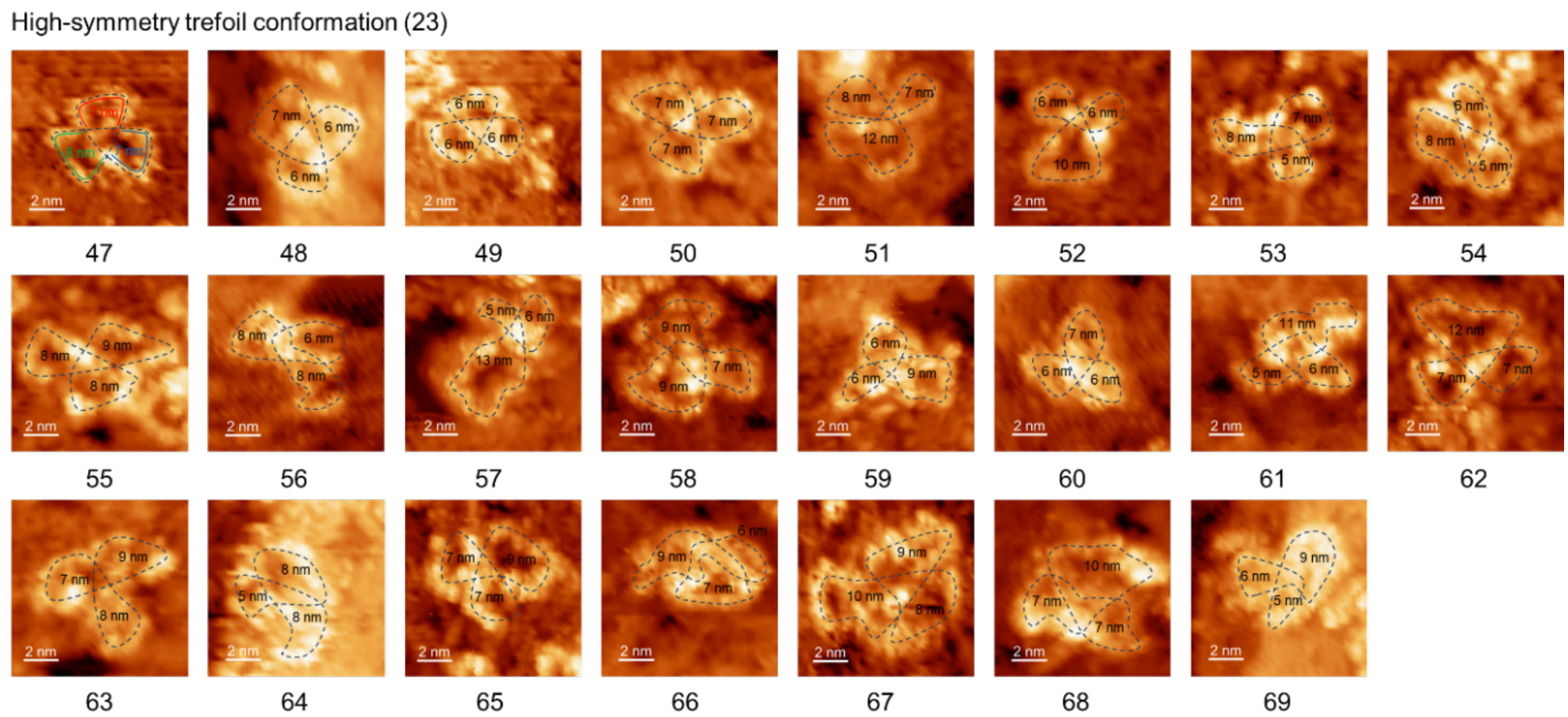

Fig. R14. Single molecular STM images of high-symmetry trefoil **2** with DCM as solvent for preparing the samples. A black dashed line superimposed on each molecule outlines the skeletal structure of the low-symmetry trefoil, with the corresponding loop lengths as indicated. The loop lengths are labelled in the loop. After analysing 69 individual images, it was found that 23 (33%) exhibited high-symmetry conformation (Scanning parameters: U = 2.7 V, I_t = 20 pA).

The above revision has been updated in the revised *Supplementary Information*.

Q15:* *'[statistical uncertainties in Fig 4c,d] Please add error bars in Fig. 4c,d.'

We have added error bars in the revised Fig. 4c, d.

Q16: *'The following questions are curiosities of mine. I think touching upon them in the manuscript would make it even more interesting to a number of researchers, but I leave the decision to the authors. I am not asking for further experiments.*

** How difficult would it be to use this strategy to produce even longer knotted polymers? Doing so would allow one to study the transition to scaling polymer theory.'*

In principle, the knot-synthon strategy is compatible with the synthesis of longer knotted polymers; however, we currently observe a gradual decrease in isolated yield with increasing chain length (SEC separation yield: 75% for **5** and 57% for **2**, see response to **Reviewer 2**, Q2), reflecting

growing challenges in ring-closure efficiency and product separation. The polymer length required to access long-chain and scaling behaviour depends on the specific knot property of interest. For example, our simulations indicate that chain lengths on the order of 100-300 repeat units are sufficient to probe knot localisation; whereas investigating asymptotic scaling of conformational size with polymer length typically requires chains comprising thousands of repeat units, and remains experimentally demanding. We are actively exploring alternative synthetic and processing methodologies aimed at extending the accessible chain-length regime.

Q17: *'Would it be possible to use co-polymers instead of PS or PEG, so that part of them has the same stiffness as the synthon? In principle, this should completely decorrelate the position of the knot from that of the synthon.'*

In theory, incorporating copolymers or stiffness-matched segments could further decorrelate the position of the knot from that of the synthon. However, the synthesis of well-defined poly(tris-bipyridine) segments with controlled length and uniform repeating units is currently very challenging, with only limited precedents reported. Short segments would effectively resemble combinations of a few synthons rather than true polymers, whereas longer segments often suffer from poor solubility and difficult purification. Encouraged by the reviewer's suggestion, we are exploring polymer-brush-based systems in which overall chain stiffness can be tuned through post-modification, and these studies are currently ongoing.

Q18: *'Would it be doable to use a similar approach with more than one synthon, resulting in composite knots?'*

The use of multiple synthons to access composite knots is conceptually feasible. As a proof of principle, we have connected two trefoil synthons prior to ring capture, yielding preliminary evidence for a $3_1\#3_1$ composite knot based on ^1H NMR and GPC analysis (Fig. R15). We are currently extending this approach to higher-order combinations (*e.g.*, $3_1\#3_1\#3_1$), and plan to employ STM and simulations to examine the relative diffusion and interaction of multiple knots along a single chain. We would welcome any suggestions from the reviewer regarding possible directions for this future work.

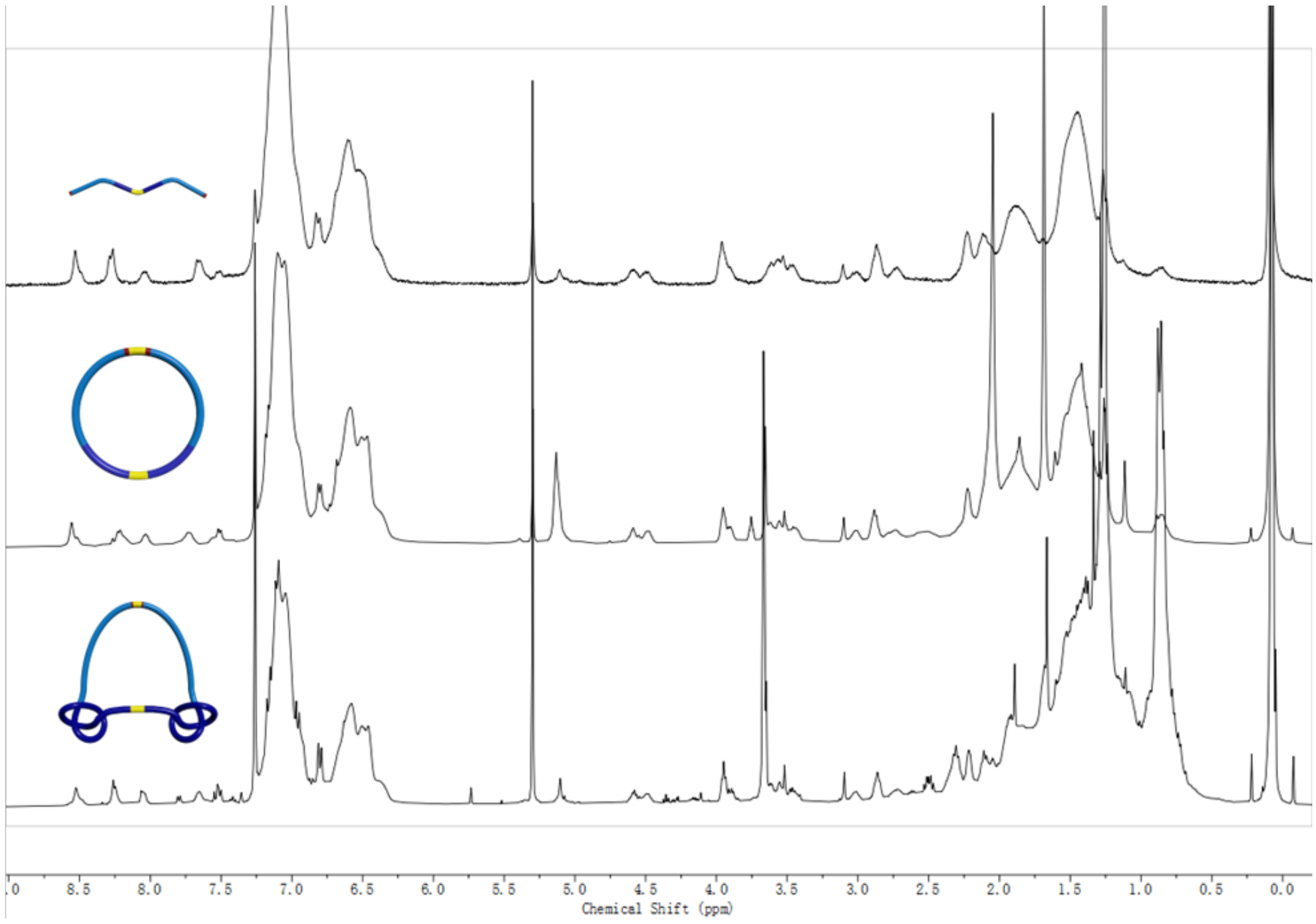

Fig. R15. Preliminary ^{1}H NMR results of $3_1\#3_1$ composite polymer knot made through the combination of two trefoil synthons.

Based on the reviewer's comments and suggestions, the relevant literature has been incorporated into the revised manuscript, as detailed below.

33. Tubiana, L. Computational study on the progressive factorization of composite polymer knots into separated prime components. *Phys. Rev. E* **89**, 052602 (2014).
34. Rieger, F. C. & Virnau, P. A Monte Carlo study of knots in long double-stranded DNA chains. *PLOS Comput. Biol.* **12**, e1005029 (2016).
44. Micheletti, C. & Orlandini, E. Numerical study of linear and circular model DNA chains confined in a slit: metric and topological properties. *Macromolecules* **45**, 2113–2121 (2012).
45. van Rensburg, E. J. Squeezing knots. *J. Stat. Mech.* **2007**, P03001 (2007).
46. Marcone, B., Orlandini, E. & Stella, A. Knot localization in adsorbing polymer rings. *Phys. Rev. E* **76**, 051804 (2007).
47. Katritch, V. et al. Geometry and physics of knots. *Nature* **384**, 142–145 (1996).
